# A Structure- and Ligand-Based Virtual Screening of a Database of “Small” Marine Natural Products for the Identification of “Blue” Sigma-2 Receptor Ligands

**DOI:** 10.3390/md16100384

**Published:** 2018-10-14

**Authors:** Giuseppe Floresta, Emanuele Amata, Carla Barbaraci, Davide Gentile, Rita Turnaturi, Agostino Marrazzo, Antonio Rescifina

**Affiliations:** 1 Department of Drug Sciences, University of Catania, V.le A. Doria, 95125 Catania, Italy; giuseppe.floresta@unict.it (G.F.); carlabarbaraci@rocketmail.com (C.B.); davide.gentile@studium.unict.it (D.G.); rita.turnaturi@unict.it (R.T.); marrazzo@unict.it (A.M.); 2 Department of Chemical Sciences, University of Catania, V.le A. Doria, 95125 Catania, Italy; eamata@unict.it

**Keywords:** virtual screening, database marine products, sigma-2 receptor, sigma-2 receptor ligands

## Abstract

Sigma receptors are a fascinating receptor protein class whose ligands are actually under clinical evaluation for the modulation of opioid analgesia and their use as positron emission tomography radiotracers. In particular, peculiar biological and therapeutic functions are associated with the sigma-2 (σ_2_) receptor. The *σ*_2_ receptor ligands determine tumor cell death through apoptotic and non-apoptotic pathways, and the overexpression of *σ*_2_ receptors in several tumor cell lines has been well documented, with significantly higher levels in proliferating tumor cells compared to quiescent ones. This acknowledged feature has found practical application in the development of cancer cell tracers and for ligand-targeting therapy. In this context, the development of new ligands that target the *σ*_2_ receptors is beneficial for those diseases in which this protein is involved. In this paper, we conducted a search of new potential *σ*_2_ receptor ligands among a database of 1517 “small” marine natural products constructed by the union of the Seaweed Metabolite and the Chemical Entities of Biological Interest (ChEBI) Databases. The structures were passed through two filters that were constituted by our developed two-dimensional (2D) and three-dimensional Quantitative Structure-Activity Relationship (3D-QSAR) statistical models, and successively docked upon a *σ*_2_ receptor homology model that we built according to the FASTA sequence of the *σ*_2_/TMEM97 (SGMR2_HUMAN) receptor.

## 1. Introduction

Sigma (*σ*) receptors include a particular pharmacologically defined family of membrane-bound receptors that bind compounds belonging to a variety of structural classes. Discovered in 1976 and recognized in two distinct subtypes in the early 1990s, they represent a potential target for the diagnosis and therapy of cancer and central nervous system (CNS) diseases [1,2]. Indeed, both *σ* receptor subtypes are highly expressed in several tissues and distinguished from each other through their affinity for different ligands and biological profiles.

The sigma-1 (*σ*_1_) receptor is a 25.3-kDa chaperone protein that resides in the endoplasmic reticulum–mitochondrion interface as well as in nuclear and plasma membranes [3,4,5], firstly cloned from Guinea pig liver cells [6] (Uni-ProtID Q60492, Gene names SIGMAR1, CHEMBL4153). The human *σ*_1_ receptor was characterized by X-ray crystallography and the crystal structures in complex with two ligands have been reported (PDB ID 5HK1 and 5HK2) [7]. Several synthetic small molecules with different structures bind with high affinity and selectivity to the *σ*_1_ receptor. The *σ*_1_ receptor agonists have shown neuroprotective, antiamnestic, and antidepressant effects; in contrast, the *σ*_1_ receptor antagonists possess modulatory effects on opioid analgesia, as well as antiproliferative and antiangiogenic activities [8,9,10,11,12,13,14].

The sigma-2 (*σ*_2_) receptor is an enigmatic protein that has attracted significant attention due to its involvement in several diseases, such as cancer and neurological disorders. However, despite the increasingly apparent medical importance of the *σ*_2_ receptor, its biological role has been stymied since the gene that encodes the receptor has never been identified, and a crystal structure of the receptor has never been released. The *σ*_2_ receptor has been reported to have a molecular weight between 18–21.5 kDa. For a long time, it has been contemplated as part of the progesterone receptor membrane component one (PGRMC-1), which is a heme-binding protein that is involved in cell survival and apoptosis [15]. Recently, it has been purified, revealing its identity as the Transmembrane Protein 97 (TMEM97), which is an endoplasmic reticulum-resident transmembrane protein that regulates the sterol transporter Niemann-Pick disease protein (NPC-1), which is involved in regulating intracellular Ca^2+^ concentration [2,11,16,17].

The *σ*_2_/Tmem97 ligands can produce a transient rise in intracellular Ca^2+^ levels [17] or overcome Ca^2+^ influx in the presence of an inducer [18]. The *σ*_2_ receptor ligands determine tumor cell death through apoptotic and non-apoptotic pathways [2,16]. The apoptotic processes mediated by the *σ*_2_ receptor include cell cycle arrest in the G1 phase, which seems to be induced by an expression of cyclinD and CDK2 proteins, as well as by a reduction in intracellular ATP levels [19]. Meanwhile, non-apoptotic mechanisms may include DNA fragmentation, lysosomal leakage, and oxidative stress [20,21,22]. Overexpressed in several tumor cell lines, the *σ*_2_ receptor ligands are actually under clinical evaluation as positron emission tomography (PET) radiotracers and indicated for the ligand-targeting therapy and as fluorescence imaging agents [23,24,25,26,27,28]. In this context, the development of new ligands that target the *σ*_2_ receptor may be particularly beneficial; on the other hand, few selective ligands have been found for the *σ*_2_ receptor, and in some cases, their finding occurred through an accidental discovery [26].

The spreading of computer science, and in particular the possibility of consulting hundreds of chemical databases, gives the opportunity to find novel compounds that are able to bind a specific receptor, enabling a rational investigation. This task may be carried out by means of virtual screening, which helps the end user in filtering many compounds based on virtual model specifications. In absence of virtual methods that are able to evenly handle an unlimited amount of compounds, this task must be manually accomplished by medicinal chemists, which has obvious limitations [29]. On these grounds and motivated by our ongoing interest in developing new compounds that selectively target the *σ*_2_ receptor, we have recently developed a two-dimensional (2D) [30,31] and a three-dimensional Quantitative Structure–Activity Relationship (3D-QSAR) model [32], which was built using the whole set of selective *σ*_2_ receptor ligands as retrieved from the *σ*_2_ receptor selective ligand database (S2RSLDB) (http://www.researchdsf.unict.it/S2RSLDB) [33].

Since structure and ligand-based computer-aided drug design are nowadays effective and useful tools in rational drug design [34,35,36,37,38,39], we used them to allow the identification of new virtually potent and selective molecules that are able to interact with the *σ*_2_ receptor. Therefore, herein, we report an investigation of new potentially *σ*_2_/TMEM97 receptor ligands among a database of 1517 “small” marine natural products, here named the Blue DataBase (BDB, Table S1), which was composed by the merging of the Seaweed Metabolite (http://www.swmd.co.in/) [40], the Chemical Entities of Biological Interest (ChEBI, http://www.ebi.ac.uk/chebi/) [41] databases, and from the reference [42]. The chemical structures were passed through two filters constituted by our developed 2D and 3D-QSAR statistical models that showed high statistical quality and robust predictive potential capability, and successively docked upon the *σ*_2_/TMEM97 receptor homology model. To the best of our knowledge, this is the first report on the build of a 3D structure of the *σ*_2_/TMEM97, by mixing the classic homology modeling approach with the evolutionary coupling analysis. Moreover, the robustness of this model has been confirmed by docking on it 200 *σ*_2_-ligands that were randomly selected from the S2RSLDB. Finally, the results of the two filters and docking have been merged, ordering them by the mean of the obtained p*K*_i_ (−log_10_*K*_i_) to draw up a list of the 15 best candidates (mean of 2D, 3D-QSAR, and docking p*K*_i_ results) as possible powerful *σ*_2_ receptor marine ligands. Four of them are already known in the literature for their antiproliferative and cytotoxic effects against A549 and HT29 cancer cell lines, which are two typical cancer cell lines characterized by *σ*_2_ receptor overexpression. In particular, compound 848 resembles progesterone, which in itself is a potent *σ*_2_ receptor ligand.

## 2. Results and Discussion

### 2.1. 2D Ligand-Based Filter

The BDB was filtered through an in-house hybrid *σ*_2_ receptor affinity filter [30], returning for each chemical entity a predicted σ_2_ receptor p*K*_i_. This 2D-QSAR model [30,31] has been built by using a Monte Carlo-based QSAR analysis employing the software CORAL (version 2016, http://www.insilico.eu/coral/index.html) [43,44]. CORAL allows for a hybrid representation of molecular structures that includes a simplified molecular input line entry system (SMILES) and a molecular graph. Hybrid representation using SMILES together with the molecular graph may give better models with higher statistical quality with respect to those models, and with a unique representation of the molecular structure [45,46]. The here-used 2D-QSAR model, being constructed with a large and structurally diverse set of 548 compounds, allows for a prediction of different populations of chemical compounds endpoints (*σ*_2_ receptor p*K*_i_).

The chemical structures of the 1517 “blue” compounds were transformed into SMILES and converted into freebase, while those compounds presenting a carboxylate were left as is. Eventually, the aromatic rings were converted into the kekulé forms. These conversions have been performed in order to generate SMILES with the same depiction as those used for building the hybrid *σ*_2_ receptor affinity filter, since this model works by chopping the SMILES or the molecular graphs into small fragments, and iteratively overmatching them with the SMILES and molecular graphs fragments that compose the training set [30,31].

Among 1517 compounds, 1313 have been defined by the model to be outliers, which means that their chemical structures were expressed as SMILES or a molecular graph that was not described by the model, entirely or for a significant part. Indeed, for these compounds, the type of SMILES or molecular graphs fragments generated do not overmatch, entirely or for a significant part, with the SMILES or molecular graphs fragments that compose the training set. For these molecules, the software still predicts an endpoint, but it highlights them for not falling in the field of applicability, and thus being outliers. The remaining 204 “blue” compounds were returned with a predicted endpoint, and were indicated as falling within the domain of applicability. From this subset, 42 compounds have been predicted to possess a *σ*_2_ receptor, *K*_i_, which was higher or equal to 100 nM (p*K*_i_ ≥ 7), and is empirically considered an appropriate value for processing a compound into more complex phenotypic assays. These compounds are reported in Table S2.

### 2.2. 3D Ligand-Based Filter

The same dataset of compounds was also evaluated using a second ligand-based filter. All of the 3D structures of the compounds were aligned to our previously published 3D-QSAR model for the *σ*_2_ receptor. The alignment of the molecules in the 3D-QSAR pharmacophore model was performed with Forge (v10.4.2, Cresset, New Cambridge House, Hertfordshire, UK) [47]. Once aligned, the compounds were scored assuming that if the fields (the local extrema of the electrostatic, van der Waals, and hydrophobic potentials of the molecule) of the newly evaluated molecules were very similar to those of the original compounds, the resulting compounds will have similar biological properties [47]. The 15 most potent compounds resulted from the 3D-ligand based filter are reported in Table 1, while the full set of compounds is present in Table S2. The selected compounds resulted in an excellent distance to the model (i.e., description by the model), which means that all or most of the features in the molecules were present in the training set of the 3D-QSAR model, and hence that the predicted activity is reliable. In the full set of evaluated compounds, there are also several compounds that are not well described by the QSAR equation; the external user should pay attention to the “3D Applicability” column in the supplementary material (Table S2). Values of “Excellent” or “OK” indicate the predictive reliability. Worse values (i.e., “Bad” or “Poor”) indicate that the molecule has field points in places that are not described by the equation, resulting in unreliable predicted activities.

### 2.3. Homology Model and Molecular Docking

To further reinforce the results obtained through the 2D and 3D filters, we decided to add a third filter based on molecular docking. Exploiting the recent identification of the *σ*_2_ receptor as the TMEM97, we built its 3D molecular structure by the homology modeling approach, starting from the Q5BJF2 (SGMR2_HUMAN) sequence deposited in the UniProt Knowledgebase (https://www.uniprot.org/uniprot/). To pursue this challenging task, due to the scarcity of crystallized structures possessing the sufficient sequence identity (>30%), we have chosen to employ an approach based on two strategies used in parallel. Indeed, this task has been developed by mixing the classical first three steps (i. finding the homologous template proteins of the known structure, ii. selecting the best template or set of templates, and iii. optimizing the multiple sequence alignment between the query and template protein sequences) [48] with the evolutionary coupling analysis [49]. 

Eventually, we performed the typical fourth step, which consisted of the build of the homology model for the query sequence that resembles the structures of the templates as closely as possible, accommodating for the deletions and insertions of query residues with respect to the template structures, in order to obtain a series of hybrid models that have been ordered by their overall *z*-score [50].

**Figure 1:**
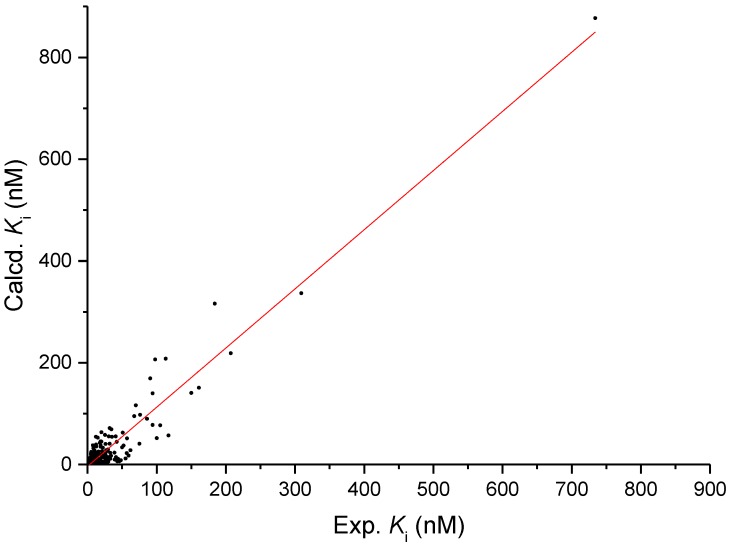
Plot of experimental *K*_i_ vs. calculated ones for 200 *σ*_2_-ligands randomly chosen from the set of selective *σ*_2_ receptor ligands as retrieved from the *σ*_2_ receptor selective ligand database (S2RSLDB). In red, the straight line corresponding to the linear regression analysis.

The obtained 3D model was docked with PB-28, which is a known *σ*_2_ receptor ligand (exp. *K*_i_ = 2.0 nM [51]); the best-obtained complex was then immersed in a simulated endoplasmic reticulum membrane, in physiological environment conditions, and subjected to a molecular dynamics (MD) simulation of 10 ns to accommodate the ligand, verified by the Root-mean-square deviation (RMSD) of the ligand. After the minimization of the frame with the best binding energy (belonging to the last 3 ns of MD simulation, where the ligand RMSD is constant), the re-docking of the ligand gave a calculated *K*_i_ of 1.5 nM, which was in excellent agreement with the experimental value. To additionally validate the 3D model, we docked 200 *σ*_2_ receptor ligands that were randomly selected from the S2RSLDB among those that possess a *K*_i_ in the range 0.01–1000 nM. The results (Table S3) are reported in Figure 1 as a plot of the experimental versus calculated *K*_i_, and highlight a very good prediction power with an *R*^2^ of 0.91.

On this model, we docked the best-predicted *σ*_2_ receptor ligands returned from the two 2D and 3D QSAR filters (524 compounds, being 13 in common). The 15 most potent compounds resulted from the docking analysis are reported in Table 2, while the full set of compounds is reported in Table S2.

**Figure 2:**
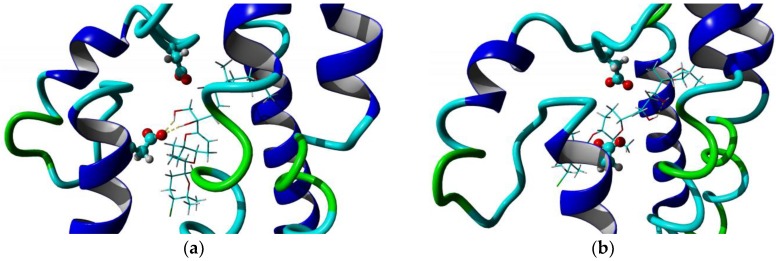
3D structures of the complex 1169/*σ*_2_ receptor (**a**) and 1421/*σ*_2_ receptor (**b**).

The 3D structures of the complex with the two best-docked molecules have been represented in Figure 2. Both molecules reside in the pocket constituted by the two ASP29 and ASP56 residues; in particular, compound 1169 shows two hydrogen bonds with ASP29.

### 2.4. The Ultimate Filter

Finally, to account for all of the typology of obtained results, we sorted the BDB library according to the mean of the predicted p*K*_i_ by 2D-QSAR, 3D-QSAR, and docking methodologies. The structures of the best 15 compounds have been reported in Table 3.

An accurate literature search for the 15 most promising hits highlighted that four of them (848, 984, 1169, and 1172) were reported to show clearly antiproliferative and cytotoxic effects against typical cancer cell lines such as A549 and HT29 (Table 4) [52,53,54,55,56,57,58]. These biological data were in agreement with specific *σ*_2_ receptors overexpression that was related to the same cell lines [59,60,61,62].

In particular, compound 848 possess a steroidal structure resembling that of progesterone; this compound has been demonstrated to act as a potent *σ*_2_ receptor ligand with a *K*_i_ of 441 nM [63]. To further validate our model and the goodness of the predicted data, we docked progesterone, obtaining a calculated *K*_i_ of 749 nM that became 369 nM after allowing a best accommodation by 100 ps step-10 annealing and 100 ps steepest descent minimization, followed by a local re-docking, which is in good agreement with the experimental *K*_i_.

## 3. Materials and Methods

### 3.1. Dataset of Marine Compounds

**Figure 3:**
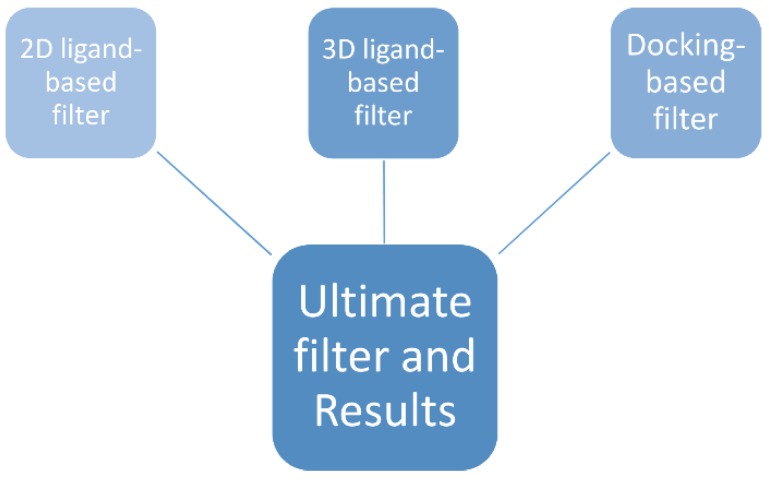
Workflow of used filters.

The chemical structures of the marine dataset were retrieved from the Seaweed Metabolite (http://www.swmd.co.in/), the Chemical Entities of Biological Interest (http://www.ebi.ac.uk/chebi/) databases, and from reference [42]. All of the molecules were manually checked, and the duplicates were removed to achieve a final number of 1517 compounds. The full list of molecules is available as SMILES for external users in the supplementary material (Table S1). In Figure 3, the workflow that was used has been schematized.

### 3.2. Structures 2D to 3D Building and Minimization

The structures of the marine products were built using Marvin Sketch (v. 18.24, ChemAxon Ltd., Budapest, Hungary) [64]. The 2D structures were subjected to molecular mechanics energy minimization by Merck molecular force field (MMFF94) using the Marvin Sketch geometrical descriptors plugin [64]. The protonation states of the molecules were calculated assuming a pH of 7.0. Before the alignment for the 3D-QSAR filter, the geometry of the obtained 3D structures was further optimized at the semi-empirical level using the parameterized model number 3 (PM3) Hamiltonian as implemented in the MOPAC package (MOPAC2016 v. 18.151, Stewart Computational Chemistry, Colorado Springs, CO, USA) [65,66,67].

### 3.3. 2D-QSAR

The software CORAL (CORrelation And Logic, version 2016, Istituto di Ricerche Farmacologiche Mario Negri, Milano, Italy) was used for building the 2D-QSAR model using 548 *σ*_2_ receptor selective ligands over the *σ*_1_ receptor, as previously reported [33,34]. The unique SMILES composing the blue dataset were converted in order to have a SMILES depiction that was equal to that used for generating the model (vide supra). To each SMILES, a random endpoint value was associated in order for the software to compare this value with the predicted one. The following regression was used for predicting the endpoints:

$$\;pKi_{\sigma 2}=3.5937472\left(\pm \;0.0139734\right)+\;0.0352642\left(\pm \;0.0001213\right)*\mathrm{DCW}\left(0,16\right)\;$$

where DCW is defined as the “descriptor of correlation weights”. The regression for the *σ*_2_ receptor p*K*_i_ has been developed in a previously published 2D regression model [30].

### 3.4. Compound Alignment for the 3D-Ligand Based Filter

All of the optimized structures were imported into the software Forge (v10.4.2, Cresset, New Cambridge House, Hertfordshire, UK) for the evaluation of the dataset in the field-based 3D-QSAR model that was previously published [32]. All of the molecules were aligned with the training set of the 3D-QSAR model. The negative, positive, shape, and hydrophobic field points of each molecule were generated using the extended electron distribution (XED) force field in Forge. The molecules were then aligned with the training set of the 3D-QSAR model by a maximum common substructure algorithm using a customized set-up. The software’s parameters that were used for the conformation hunt and alignment are presented in the supplementary material (Figures S1 and S2). The maximum number of conformations that was generated for each molecule was set to 500, as suggested by the software. The root mean square deviation of atomic positions’ cutoff for duplicate conformers was set to 0.5 Å (the similarity threshold below which two conformers are assumed to be identical). The gradient cutoff for conformer minimization was set to 0.1 kcal/mol. The energy window was set to 2.5 kcal/mol. Conformers with a minimized energy outside the energy window were discarded.

### 3.5. Homology Model and Docking

All of the simulations and molecular modeling experiments have been conducted with YASARA software (v. 18.4.24, YASARA Biosciences GmbH, Vienna, Austria). The homology model was built starting from the Q5BJF2 (SGMR2_HUMAN) sequence deposited in the UniProt Knowledgebase (https://www.uniprot.org/uniprot/) and using the crystallographic structures corresponding to the following PDB IDs as templates: 4LGC, 1VT4, 4M58, 2PFF, 2MGY, AND 1T33. To these structures have been added the best two structures obtained by the evolutionary coupling analysis, which were executed with the EVfold web-server (http://evfold.org/evfold-web/newprediction.do), and the ensemble has been processed with the hm_build macro of YASARA. In the end, an optimized hybrid model was built through iteratively replacing bad regions in the top scoring model with the corresponding fragments from the other models.

This model was docked with the *σ*_2_ receptor ligand PB-28 (see below for details), and the best pose ligand/receptor complex structure was then immersed in a simulated endoplasmic reticulum membrane [68], in physiological environment conditions, and subjected to a molecular dynamics (MD) simulation of 10 ns to accommodate the ligand. The simulation was set up automatically by first scanning the protein for exposed transmembrane helices [i.e., helices longer than 16 residues, with more than seven hydrophobic residues and more than three exposed ones (accessible side-chain surface area >30% of maximum)]. The major axis vectors of these helices (i.e., the direction vectors of the least-squares lines through the C_alpha_ atoms) were summed up to obtain the major axis of the protein, which was then oriented along the Y-axis, normally with respect to the plane of the membrane and the X–Z plane. The best shift of the membrane along this major axis was obtained by scanning the protein for the region with the largest number of exposed hydrophobic residues (see definition above) and a width of 28 Å (corresponding to the membrane core).

Having placed an equilibrated membrane structure (consisting of 55% of phosphatidylcholine, 30% of phosphatidylethanolamine, 10% of phosphatidylserine, and 5% of phosphatidylinositol molecules [68]) at this location named ‘MemCenterY’, the system was enclosed in a simulation cell of size [X*Y*Z] Å, the protein was temporarily scaled by 0.9 along the X–Z axes, and then, strongly clashing membrane lipids were deleted (lipids with an atom closer than 0.75 Å to a protein atom).

The temporary protein scaling, which was needed to avoid the deletion of too many lipids around the protein, was then slowly removed during a short simulation at 298 K in vacuum. The protein (with all of the atoms kept fixed) was scaled by 1.02 along the X–Z axes every 200 femtoseconds, while the membrane was allowed to move, but was restrained to ideal geometry (by pulling lipid residues with an atom further than 21.5 Å away from MemCenterY back into the membrane, and by pushing phosphorus atoms closer than 14 Å to MemCenterY back outwards). The force field was AMBER14, with Lipid17/GAFF2/AM1BCC parameters for non-standard residues. As soon as the protein had reached its original size again, the protein side-chain p*K*_a_s were predicted, protonation states were assigned according to pH 7.4, and the simulation cell was filled with water, 0.9% NaCl, and counter ions (proteins 57, 678–683). The main simulation was then run with PME, and an 8.0 Å cutoff for non-bonded real space forces, a four fs time-step, constrained hydrogen atoms, and at constant pressure and temperature (NPT ensemble), as described in detail previously. During the initial 250 picoseconds, the membrane was restrained to avoid distortions while the simulation cell adapted to the pressure exerted by the membrane (see above, additionally water molecules that got closer than 14 Å to MemCenterY were pushed outside). The source code of this simulation protocol and visualizations of the individual steps can be found at www.yasara.org/membranemd.

Docking experiments were effectuated, employing the AutoDock (4.2.5.1, The Scripps Research Institute, San Diego, California Jupiter, Florida, US) software implemented in YASARA. The maps were generated by the program AutoGrid (4.2.5.1, The Scripps Research Institute, San Diego, California Jupiter, Florida, US) with a spacing of 0.375 Å, and dimensions that encompass all of the atoms extending 5 Å from the surface of the PM3 minimized structure of PB-28. All of the parameters were inserted at their default settings. In the docking tab, the macromolecule and ligand are selected, and Lamarckian Genetic Algorithm (LGA) parameters are set as ga_runs = 100, ga_pop_size = 150, ga_num_evals = 20,000,000, ga_num_generations = 27,000, ga_elitism = 1, ga_mutation_rate = 0.02, ga_crossover_rate = 0.8, ga_crossover_mode = two points, ga_cauchy_alpha = 0.0, ga_cauchy_beta = 1.0, number of generations for picking worst individual = 10.

## 4. Conclusions

In this study, we describe the screening of new potentially *σ*_2_/TMEM97 receptor ligands that were reported in a database of 1517 “small” marine natural products, and formulated by the union of the Seaweed Metabolite (http://www.swmd.co.in/) and the Chemical Entities of Biological Interest (ChEBI, http://www.ebi.ac.uk/chebi/). The structures were selected by our developed 2D and 3D-QSAR statistical models, and successively verified by a robust *σ*_2_/TMEM97 receptor homology model appropriately built by us. This work provided 15 best candidates as powerful *σ*_2_ receptor marine ligands; four of them are clearly reported in the literature as antiproliferative and cytotoxic compounds against typical cancer cell lines such as A549 and HT29, and are in agreement with the specific *σ_2_* receptor overexpression that is related to the same cell lines. In particular, compound 848 resembles progesterone, which in itself is a potent *σ*_2_ receptor ligand. These findings will ensure prospectively advantageous applications to speed up the design and identification of new natural hit compounds as potent and selective *σ*_2_ receptor ligands. In vitro and biological screenings of the most promising compounds are in due course.

## Figures and Tables

**Table 1:** Structure and predicted p*K*_i_ values of the 15 most potent marine products resulted from the three-dimensional Quantitative Structure-Activity Relationship (3D-QSAR) filter. BDB: Blue DataBase.

BDB ID	Structure	Predicted p*K*_i_
621	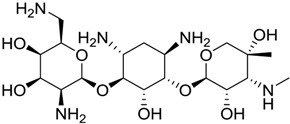	10.9
1612	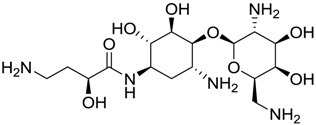	9
848	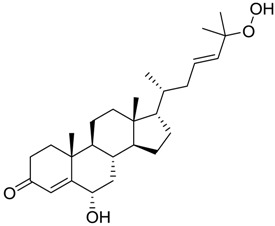	8.6
670	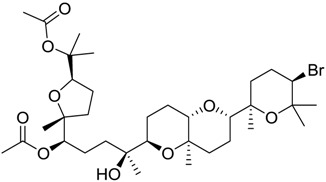	8.3
1703	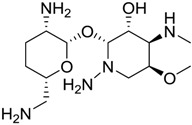	8.3
1144	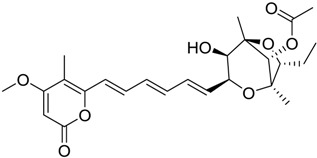	8.3
1342	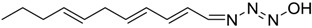	8.3
55	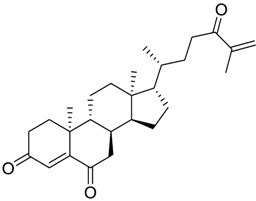	8.2
972	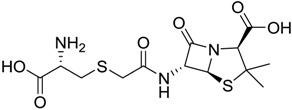	8.2
933	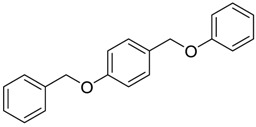	8.1
1426	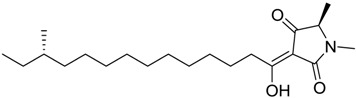	8.1
120	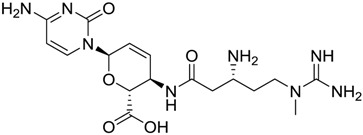	8.1
414	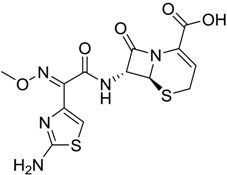	8.1
1453	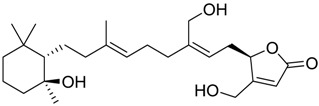	8
84	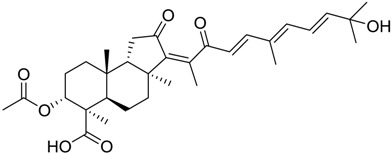	8

**Table 2:** Structure and calculated p*K*_i_ values of the 15 most potent marine products resulted from docking.

BDB ID	Structure	Calcd. p*K*_i_
1169	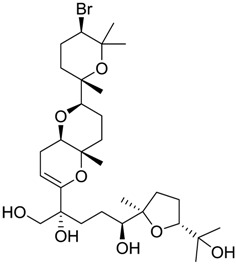	10.26
1421	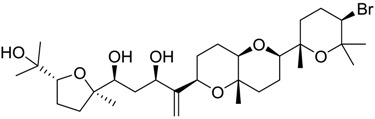	9.79
984	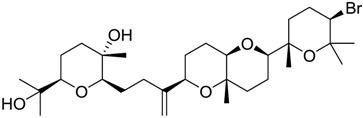	9.77
28	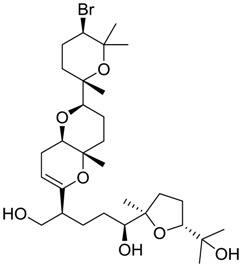	9.64
306	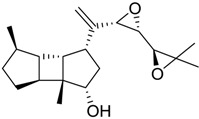	9.60
1333	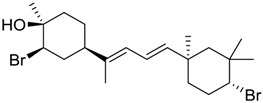	9.45
45	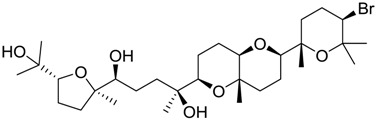	9.42
524	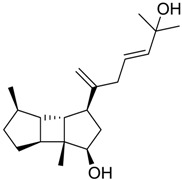	9.31
1172	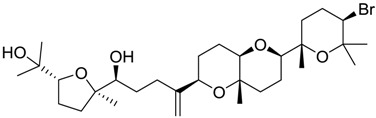	9.31
1123	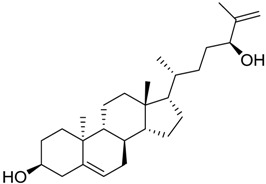	9.23
279	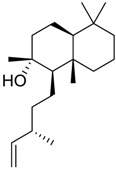	9.18
1581	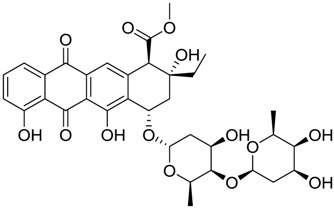	9.14
798	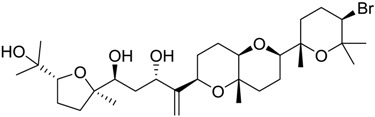	9.05
84	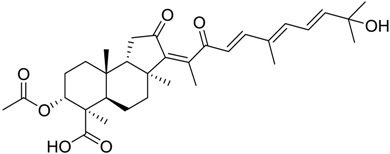	9.03
529	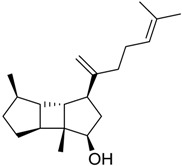	8.95

**Table 3:** Structure, calculated mean p*K*_i_, and corresponding *K*_i_ (nM) values of the 15 most potent marine products resulted from the mean of the three combined filters.

BDB ID	Structure	Mean p*K*_i_	Mean *K*_i_
1169	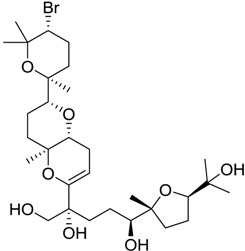	9.24	0.6
28	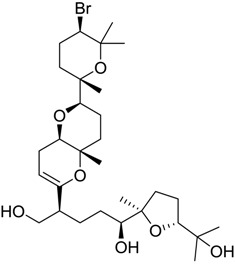	8.91	1.2
45	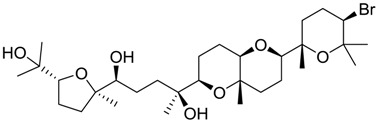	8.89	1.3
1172	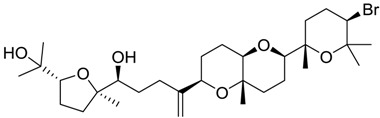	8.89	1.3
1421	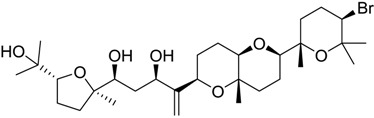	8.70	2.0
246	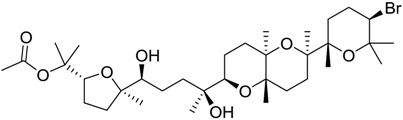	8.60	2.5
14	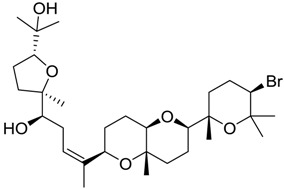	8.57	2.7
298	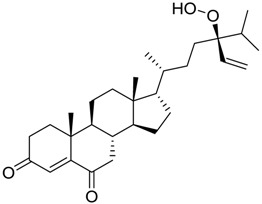	8.55	2.8
798	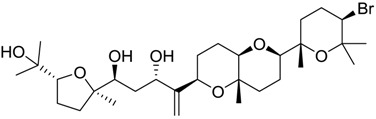	8.54	2.9
984	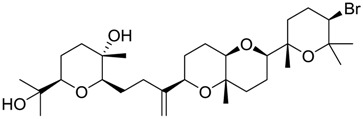	8.44	3.6
1179	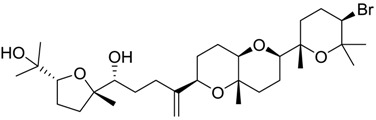	8.44	3.6
848	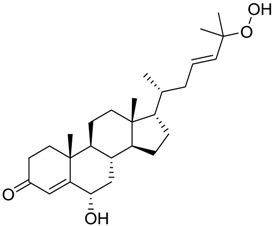	8.43	3.7
1333	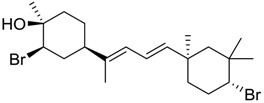	8.40	4.0
420	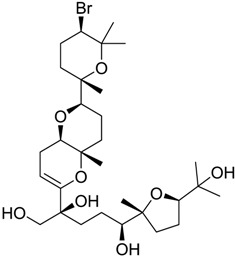	8.33	4.6
272	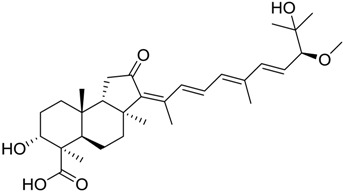	8.27	5.3

**Table 4:** ED_50_ (μg/mL) of four BDB compounds on A549 and HT29 cell lines that are known to overexpress the *σ*_2_ receptor.

BDB ID	A-549	HT-29
848	1.00	0.63
984	2.5	2.5
1169	10	10
1172	2.5	2.5

## Supplementary Materials

The following are available online at http://www.mdpi.com/1660-3397/16/10/384/s1, Figure S1: Forge’s parameters used for the conformation hunt, Figure S2: Forge’s parameters used for the alignment, Figure S3: Homology model of the *σ*_2_-receptor immersed in the endoplasmic reticulum membrane, Table S1: Dataset of marine products, Table S2: Complete results of the three filters by color code, Table S3: Experimental and calculated *K*_i_ by docking.
