# Supplementary material for: A Structure- and Ligand-Based Virtual Screening of a Database of “Small” Marine Natural Products for the Identification of “Blue” Sigma-2 Receptor Ligands
Source: Mar Drugs. 2018 Oct 14;16(10):384. doi: 10.3390/md16100384 (PMC6212963; doi:10.3390/md16100384)
Supplement: Supplementary file 1 [file marinedrugs-16-00384-s001.pdf]

marine drugs

Supplementary material

A structure and ligand-based virtual screening of a database of "small" marine natural products for the identification of "blue" sigma-2 receptor ligands

Giuseppe Floresta [1,2], Emanuele Amata [2], Carla Barbaraci [1], Davide Gentile [1], Rita Turnaturi [1], Agostino Marrazzo [1] and Antonio Rescifina [1,*]

[1] Department of Drug Sciences, University of Catania, V.le A. Doria, 95125 Catania, Italy
[2] Department of Chemical Sciences, University of Catania, V.le A. Doria, 95125 Catania, Italy
* Correspondence: arescifina@unict.it; Tel.: +39-095-738-5017

Table of content

Fig. S1. Forge's parameters used for the conformation hunt S2
Fig. S2. Forge's parameters used for the alignment S2
Table S1. Dataset of marine products S3
Table S2. Complete results of the three filters by color code S51
Table S3. Experimental and calculated K_i by docking S87
Fig. S3. Homology model of the σ_2-receptor immersed in the endoplasmic reticulum membrane S89

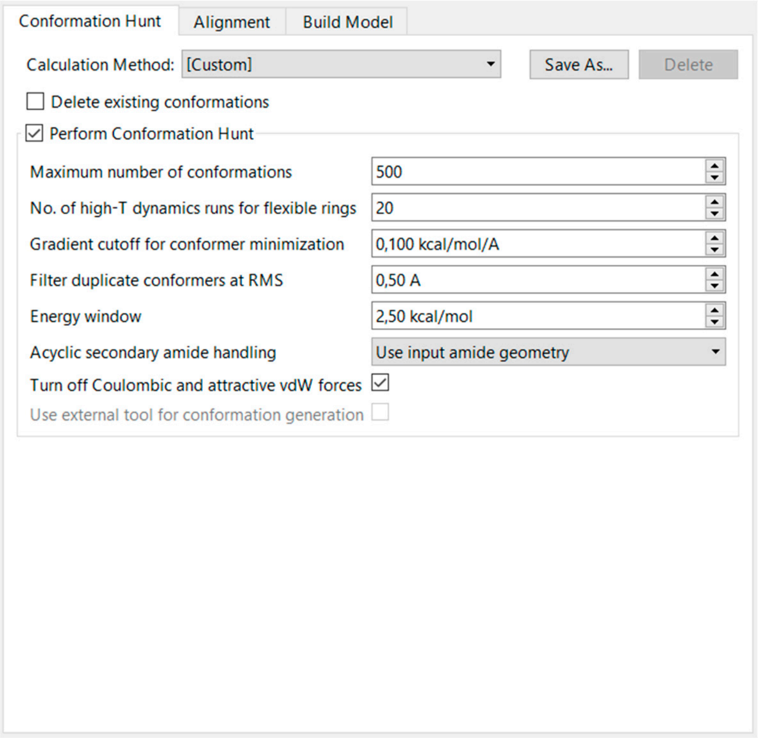

Fig. S1. Forge's parameters used for the conformation hunt.

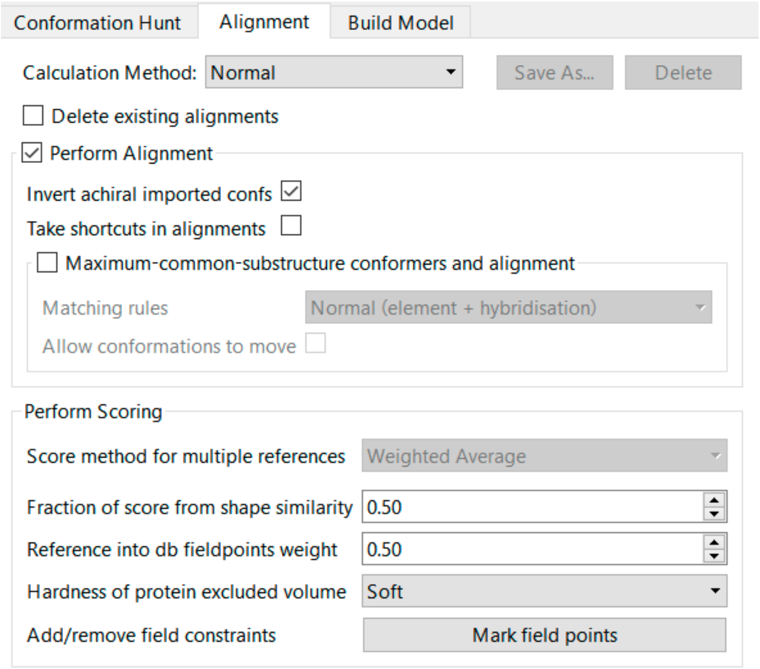

Fig. S2. Forge's parameters used for the alignment.

Table S1. Dataset of marine products.

Entry	BDB ID	SMILES
1	1169	CC(C)(O)[C@H]1CC[C@@](C)(O1)[C@@H](O)CC[C@@](O)(CO)C1=CC[C@H]2O[C@H](CC[C@]2(C)O1)[C@]1(C)CC[C@@H](Br)C(C)(C)O1
2	28	CC(C)(O)[C@H]1CC[C@@](C)(O1)[C@@H](O)CC[C@@H](CO)C1=CC[C@H]2O[C@H](CC[C@]2(C)O1)[C@]1(C)CC[C@@H](Br)C(C)(C)O1
3	45	CC(C)(O)[C@H]1CC[C@@](C)(O1)[C@@H](O)CC[C@](C)(O)[C@H]1CC[C@H]2O[C@H](CC[C@]2(C)O1)[C@]1(C)CC[C@@H](Br)C(C)(C)O1
4	1172	CC(C)(O)[C@H]1CC[C@@](C)(O1)[C@@H](O)CCC(=C)[C@H]1CC[C@H]2O[C@H](CC[C@]2(C)O1)[C@]1(C)CC[C@@H](Br)C(C)(C)O1
5	1421	CC(C)(O)[C@H]1CC[C@@](C)(O1)[C@@H](O)C[C@@H](O)C(=C)[C@H]1CC[C@H]2O[C@H](CC[C@]2(C)O1)[C@]1(C)CC[C@@H](Br)C(C)(C)O1
6	246	CC(=O)OC(C)(C)[C@H]1CC[C@@](C)(O1)[C@@H](O)CC[C@](C)(O)[C@H]1CC[C@@]2(C)O[C@](C)(CC[C@]2(C)O1)[C@]1(C)CC[C@@H](Br)C(C)(C)O1
7	14	C\C(=C\C[C@@H](O)[C@@]1(C)CC[C@@H](O1)C(C)(C)O)[C@H]1CC[C@H]2O[C@H](CC[C@]2(C)O1)[C@]1(C)CC[C@@H](Br)C(C)(C)O1
8	298	CC(C)[C@](CC[C@@H](C)[C@@H]1CC[C@H]2[C@@H]3CC(=O)C4=CC(=O)CC[C@]4(C)[C@H]3CC[C@]12C)(OO)C=C
9	798	CC(C)(O)[C@H]1CC[C@@](C)(O1)[C@@H](O)C[C@H](O)C(=C)[C@H]1CC[C@H]2O[C@H](CC[C@]2(C)O1)[C@]1(C)CC[C@@H](Br)C(C)(C)O1
10	984	CC(C)(O)[C@H]1CC[C@](C)(O)[C@@H](CCC(=C)[C@H]2CC[C@H]3O[C@H](CC[C@]3(C)O2)[C@]2(C)CC[C@@H](Br)C(C)(C)O2)O1
11	1179	CC(C)(O)[C@H]1CC[C@](C)(O1)[C@H](O)CCC(=C)[C@H]1CC[C@H]2O[C@H](CC[C@]2(C)O1)[C@]1(C)CC[C@@H](Br)C(C)(C)O1
12	848	C[C@H](C\C=C\C(C)(C)OO)[C@@H]1CC[C@H]2[C@@H]3C[C@H](O)C4=CC(=O)CC[C@]4(C)[C@H]3CC[C@]12C
13	1333	C\C(=C/C=C/[C@]1(C)CC[C@@H](Br)C(C)(C)C1)[C@@H]1CC[C@](C)(O)[C@H](Br)C1
14	420	CC(C)(O)[C@H]1CC[C@@](C)(O1)[C@@H](O)CC[C@](O)(CO)C1=CC[C@H]2O[C@H](CC[C@]2(C)O1)[C@]1(C)CC[C@@H](Br)C(C)(C)O1
15	272	CO[C@@H](\C=C\C(\C)=C\C=C\C(\C)=C1/C(=O)C[C@@H]2[C@]1(C)CC[C@@H]1[C@]2(C)CC[C@@H](O)[C@]1(C)C([O-])=O)C(C)(C)O
16	84	CC(=O)O[C@@H]1CC[C@]2(C)[C@@H]3CC(=O)\C(=C(\C)C(=O)\C=C\C(\C)=C\C=C\C(C)(C)O)[C@@]3(C)CC[C@H]2[C@@]1(C)C([O-])=O
17	1048	C[C@H](C\C=C\C(C)(C)OO)[C@@H]1CC[C@H]2[C@H]3CC=C4C[C@H](O)CC[C@@]4(C)[C@@H]3CC[C@]12C
18	914	CC(C)[C@@](CC[C@@H](C)[C@H]1CC[C@H]2[C@H]3CC=C4C[C@@H](O)CC[C@]4(C)[C@H]3CC[C@]12C)(OO)C=C
19	1232	C[C@H](C\C=C\C(C)(C)OO)[C@@H]1CC[C@H]2[C@@H]3CC(=O)C4=CC(=O)CC[C@@]4(C)[C@@H]3CC[C@@]12C
20	1223	C[C@H](CC[C@H](O)C(C)=C)[C@@H]1CC[C@@H]2[C@H]3CC=C4C[C@@H](O)CC[C@@]4(C)[C@@H]3CC[C@]12C
21	302	C\C=C(/CC[C@@H](C)[C@@H]1CC[C@H]2[C@@H]3CC=C4C[C@@H](O)CC[C@]4(C)[C@H]3CC[C@]12C)C(C)C

22	640	CO[C@H](\C=C\C(\C)=C\C=C\C(\C)=C1/C(=O)C[C@@H]2[C@]1(C)CC[C@@H]1[C@]2(C)CC[C@@H](OC(C)=O)[C@]1(C)C([O-])=O)C(C)(C)O
23	981	CC[C@H]1O[C@@H]2C[C@@H](O[C@@H]2C\C=C/C[C@@H]1Br)C=C=CBr
24	1273	CC(C)[C@](CC[C@@H](C)[C@@H]1CC[C@H]2[C@H]3C[C@@H](O)C4=CC(=O)CC[C@]4(C)[C@H]3CC[C@]12C)(OO)C=C
25	258	CC[C@H]1O[C@H]2C\C(O[C@H]2C[C@@H]1Br)=C(\Br)C\C=C\C#C
26	1190	CC(C)(O)[C@H]1CC[C@](C)(O1)[C@H]1CC[C@](C)(O1)[C@H]1CC[C@H]2O[C@H](CC[C@]2(C)O1)[C@@]1(C)OC(C)(C)[C@H](Br)C[C@@H]1O
27	670	CC(=O)O[C@H](CC[C@](C)(O)[C@H]1CC[C@@H]2O[C@@H](CC[C@@]2(C)O1)[C@@]1(C)CC[C@@H](Br)C(C)(C)O1)[C@@]1(C)CC[C@@H](O1)C(C)(C)OC(C)=O
28	456	CC[C@H]1O[C@H]2C\C(O[C@H]2C[C@@H]1Br)=C(\Br)C\C=C/C#C
29	279	C[C@@H](CC[C@H]1[C@](C)(O)CC[C@H]2C(C)(C)CCC[C@]12C)C=C
30	841	C[C@H](C\C=C\C(C)(C)O)[C@@H]1CC[C@@H]2[C@H]3CC=C4C[C@H](O)CC[C@@]4(C)[C@@H]3CC[C@]12C
31	903	CC(C)[C@](O)(CC[C@@H](C)[C@H]1CC[C@H]2[C@@H]3CC=C4CC(=O)CC[C@]4(C)[C@H]3CC[C@]12C)C=C
32	1158	CC[C@@H]1O[C@@H]2C[C@H](Cl)[C@H](C\C=C\C#C)O[C@@H]2C[C@H]1Br
33	1197	C[C@@H]1C[C@@H](C[C@@H]2CCCCN2)[C@H]2CCCN[C@@H]2C1
34	1087	CC(C)C(=C\C[C@@H](C)[C@H]1CC[C@H]2[C@@H]3CC=C4C[C@@H](O)CC[C@]4(C)[C@H]3CC[C@]12C)\[C@@H](C)O
35	434	CC(C)(O)[C@@H](O)CC[C@](C)(O)[C@@H]1CC[C@@](C)(O1)[C@H]1CC[C@H]2O[C@H](CC[C@]2(C)O1)[C@]1(C)CC[C@@H](Br)C(C)(C)O1
36	99	CC(C)[C@@](CC[C@@H](C)[C@@H]1CC[C@H]2[C@@H]3CC=C4C[C@@H](O)CC[C@]4(C)[C@H]3CC[C@]12C)(OO)C=C
37	1051	C[C@@H](CC[C@H]1[C@](C)(O)CC[C@H]2C(C)(C)[C@@H](O)CC[C@]12C)C=C
38	933	C(OC1=CC=C(COC2=CC=CC=C2)C=C1)C1=CC=CC=C1
39	245	C[C@H](C\C=C\C(C)(C)O)[C@@H]1CC[C@@H]2[C@H]3C[C@@H](O)C4=CC(=O)CC[C@]4(C)[C@H]3CC[C@]12C
40	524	C[C@@H]1CC[C@H]2[C@@H]1[C@@H]1[C@H](C[C@@H](O)[C@]21C)C(=C)C\C=C\C(C)(C)O
41	122	C\C=C(/CC[C@@H](C)[C@@H]1CC[C@H]2[C@@H]3CCC4=CC(=O)CC[C@]4(C)[C@H]3CC[C@]12C)C(C)C
42	827	CC[C@H]1O[C@@H]2C[C@H](O[C@@H]2C\C=C/C[C@@H]1Br)C=C=CBr
43	306	C[C@@H]1CC[C@H]2[C@@H]1[C@@H]1[C@@H](C[C@H](O)[C@]21C)C(=C)[C@@H]1O[C@@H]1[C@@H]1OC1(C)C
44	1114	C[C@H]1CC[C@@H]2C(=CC[C@@]2(C)[C@]1(O)CC[C@@](C)(O)C=C)C(C)(C)OO
45	95	C\C=C(/CC[C@@H](C)[C@@H]1CC[C@H]2[C@H]3C[C@@H](O)C4=CC(=O)CC[C@]4(C)[C@H]3CC[C@]12C)C(C)C
46	1049	C\C(=C/C=C/C(C)(C)O)[C@@H]1CC[C@@]2(C)[C@H](Br)CCC(=C)[C@]2(O)C1
47	1235	C\C(CCC1=C(C)CCCC1(C)C)=C/CCC1=CC[C@@H](O[C@H]1O)C1=CC(=O)O[C@H]1O
48	170	C[C@H](CCC=C(C)C)[C@@H]1CC[C@@H](C)[C@@H]2C[C@@H]2C2=C1C(=O)OC2
49	257	C[C@H](CC[C@@H]1OC1(C)C)[C@@H]1CC[C@H]2[C@H]3CC=C4C[C@H](O)CC[C@@]4(C)[C@@H]3CC[C@@]12C
50	820	CC(C)CCC[C@@H](C)[C@H]1CC[C@H]2[C@@H]3CC=C4C[C@@H](O)CC[C@]4(C)[C@H]3CC[C@]12C

51	1429	C[C@H](CC[C@@H]1[C@@H](C)[C@]2(C)CC[C@@]1(C)O2)[C@H]1CCC(C)=C[C@H]1O
52	1228	CC(C)(O)[C@H](O)CC[C@@]1(C)O[C@@](C)(CC=C1)[C@H]1CC[C@H]2OC(=CC[C@]2(C)O1)[C@]1(C)CC[C@@H](O)C(C)(C)O1
53	151	CC[C@H]1O[C@@H]2C[C@@H](O[C@H]2C\C=C\C#C)[C@H](O)C[C@@H]1Br
54	1474	CC1(C)CCC[C@@]2(C)[C@H]1CC[C@]1(C)[C@@H]2C[C@@H](O)[C@]2(C)[C@H]3OC(=O)C=C3[C@@H](O)C[C@@H]12
55	1299	CC(C)[C@@](CC[C@@H](C)[C@@H]1CC[C@H]2[C@H]3CCC4=CC(=O)CC[C@]4(C)[C@H]3CC[C@]12C)(OO)C=C
56	242	CC[C@H](Br)[C@H]1C[C@@H]2O\C(=C/C\C=C/C#C)[C@@H](Br)[C@@H]2O1
57	348	CC[C@@H](Br)[C@H]1C[C@@H](Cl)[C@@H](C\C=C/C\C=C\C#C)O1
58	696	CC(C)=CCC\C(=C\CC\C(C)=C\CC[C@@]1(C)OC2=C(C)C=C(O)C=C2C=C1)C([O-])=O
59	1123	CC(=C)[C@H]1CC[C@]2(C)C[C@@H]3[C@@](C)(O)CC[C@H](O)[C@@]3(C)CC[C@@H]12
60	734	CC[C@H]1O[C@H]2C[C@@H](OO)[C@@H](C[C@@H]1Br)O[C@@H]2C\C=C/C#C
61	850	CC[C@@H]1O[C@@H](C[C@@H]1O)[C@@H]1C[C@H](O)[C@H](C\C=C\C#C)O1
62	625	C[C@H](CC[C@@H]1OC1(C)C)[C@H]1CC[C@H]2[C@@H]3C[C@@H](O)C4=CC(=O)CC[C@]4(C)[C@H]3CC[C@@]12C
63	761	CC(C)=CCC[C@](C)(O)[C@@H]1[C@@H](O)CC(=C)[C@@H]2CC=C(C)[C@H]2[C@@H]1O
64	671	C[C@]12C[C@](C)(CCCCCC\C=C\C=C\C3=CC=CC=C3)OO[C@H]1CC(=O)O2
65	1386	C[C@H]1C[C@@H]1\C=C\CC\C=C/C=C\CC\C=C/C(=O)NC(CO)CO
66	1400	CC[C@@H](C)C[C@@H](C)\C=C\CC[C@@H](O)[C@@H](N)CC1=CC=C(O)C=C1
67	1009	CC1(C)C=CC[C@@]2(C)[C@H]1CC[C@@]1(C)C[C@H](O)C(=C)CC[C@H]21
68	570	CC[C@H]1O[C@@H]2C[C@@H](O[C@H]2C\C=C\C#C)[C@@H](O)\C=C/1
69	55	C[C@H](CCC(=O)C(C)=C)[C@@H]1CC[C@H]2[C@H]3CC(=O)C4=CC(=O)CC[C@@]4(C)[C@@H]3CC[C@@]12C
70	1471	CC(C)C(\CC[C@@H](C)[C@@H]1CC[C@H]2[C@H]3CC=C4C[C@@H](O)CC[C@]4(C)[C@H]3CC[C@]12C)=C\COO
71	1546	CO[C@@H]1C[C@@]2(OC1(C)C)OC(C\C(C)=C\CC1=C(O)C(C)=CC(OC)=C1)=C[C@]1(C)CCC[C@]21C
72	1036	C[C@H](CC[C@H](OO)C(C)=C)[C@@H]1CC[C@@H]2[C@H]3CC=C4C[C@@H](O)CC[C@]4(C)[C@@H]3CC[C@]12C
73	684	CC[C@H]1CCC[C@@](C)(CO)[C@H]2C=C[C@H](C)[C@@H]12
74	1094	CC(C)[C@@](O)(CC[C@@H](C)[C@H]1CC[C@H]2[C@@H]3CC=C4C[C@H](O)CC[C@]4(C)[C@H]3CC[C@]12C)C=C
75	975	CC(=C)\C=C\C\C(C)=C\CC\C(C)=C\CC\C(C)=C\CO
76	1034	CC(C)CCC[C@@H](C)[C@H]1[C@@H](O)C[C@H]2[C@H]3CC(=O)[C@H]4CC(=O)CC[C@]4(C)[C@H]3CC[C@]12C
77	1496	CC[C@@H](Br)[C@@H]1C\C=C/C[C@H](Cl)[C@H](C\C=C/C#C)O1
78	1262	C[C@@H]1CC[C@H]2C(C)(C)CCC[C@]2(C)[C@H]1CCC(=C)C=C
79	916	C[C@]1(CC(=C)[C@H]2CC[C@](C)(O)[C@@H](O)C[C@@H]12)[C@@H]1CC[C@](C)(O)[C@H](Br)C1
80	793	CC(C)(O)[C@H]1CC[C@@](C)(O1)[C@@H](O)CC[C@@](O)(CO)C(=O)[C@H](O)C[C@H]1O[C@H](CC[C@]1(C)O)[C@]1(C)CC[C@@H](Br)C(C)(C)O1
81	854	CC1=CC(O)=C(C=C1)[C@]1(C)CC[C@@H]2C[C@]12C
82	1112	C\C=C(/CC[C@H](C)[C@@H]1CC[C@H]2[C@@H]3CC[C@@]4(O)OC(=O)C[C@]4(C)[C@H]3CC[C@]12C)C(C)C

83	1215	C\C(CCC1=C(C)CC[C@H]2C(C)(C)CCC[C@]12C)=C/CC1=C(O)C=CC(=C1)C([O-])=O
84	1300	COC1=CC(C)=C2O[C@@]3(C[C@]2(OO)C1=O)[C@@H](C)CC[C@@H]1[C@@]2(C)CC[C@H](O)C(C)(C)[C@@H]2CC[C@@]31C
85	1329	CC[C@@H](Br)[C@H]1C[C@H](O)[C@H](O)C[C@H]2O[C@@H](C[C@H]2O1)C=C=CBr
86	1225	C[C@H]1CC[C@]2(O)[C@H]1CC[C@H](C)[C@@H]2C=C(C)C
87	1436	CC\C(Br)=C/C=C/[C@@H]1C[C@H]2O[C@@H](C[C@H]2O1)C=C=CBr
88	85	C[C@@H]1CC[C@H]2C(C)(C)C(=O)CC[C@]2(C)[C@H]1CCC(=C)C=C
89	594	C[C@]12COC3=C1[C@](C)([C@H](O)CC2)[C@@]1(O)CC[C@](C)(C=C)C=C1C3=O
90	1037	CC(C)(O)[C@H]1CC[C@@](C)(O1)[C@@H](O)CCC(=C)[C@H]1CC[C@H]2O[C@]3(C)CC[C@@H](Br)C(C)(C)O[C@H]3CC[C@]2(C)O1
91	1245	CC1=CC(O)=C(C=C1)[C@]1(C)CC[C@H](Br)C1(C)C
92	1265	C\C(CCC1=C(C)CCCC1(C)C)=C/CC\C(CO)=C/C[C@@H](O)C1=CC(=O)O[C@@H]1O
93	1581	CC[C@@]1(O)C[C@H](O[C@@H]2C[C@@H](O)[C@@H](O[C@@H]3C[C@H](O)[C@H](O)[C@H](C)O3)[C@@H](C)O2)C2=C(O)C3=C(C=C2[C@H]1C(=O)OC)C(=O)C1=CC=CC(O)=C1C3=O
94	1133	CC[C@H]1O[C@H]2C[C@H](O[C@@H]2C\C=C/C#C)[C@@H](Cl)C[C@@H]1Br
95	1428	CC[C@H]1O[C@H]2C[C@](OC)(O[C@H]2C[C@@H]1Br)[C@H](Br)C\C=C\C#C
96	939	CC1(C)[C@H](Br)CCC(=C)[C@@]11CC\C(=C\Br)C=C1
97	1455	COC(=O)CCCNC(=O)N(CC1=C(Br)C(Br)=C(O)C(O)=C1)CC1=C(CC2=C(Br)C(Br)=C(O)C(O)=C2)C(Br)=C(O)C(O)=C1
98	653	C[C@H](CC[C@@H](OO)C(C)=C)[C@@H]1CC[C@H]2[C@H]3CC(=O)C4=CC(=O)CC[C@]4(C)[C@@H]3CC[C@@]12C
99	352	CC1(C)CCC[C@@]2(C)[C@H]1CC[C@@]1(C)OC3=CC=C(O)C=C3C[C@H]21
100	353	COC(=O)[C@H]1C2=CC3=C(C(O)=C2[C@H](C[C@]1(C)O)O[C@@H]1C[C@@H]([C@@H](O[C@H]2C[C@@H](O)[C@H](O)[C@@H](C)O2)[C@H](C)O1)N(C)C)C(=O)C1=C(O)C=CC=C1C3=O
101	538	CC(=O)OC[C@]12CC[C@](OO1)(C=C2)[C@@H]1CC[C@@H](Br)C(C)(C)C1
102	369	C[C@@H](CCC=C(C)C)[C@@H]1CC[C@](C)(O)[C@@H]2CC=C(C)[C@H]2[C@@H]1O
103	796	COC1=C(N2[C@@H](SC1)[C@H](NC(=O)[C@H](N)C1=CCC=CC1)C2=O)C([O-])=O
104	962	C[C@H](CC[C@H](O)C(C)=C)[C@@H]1CC[C@@]2(C)[C@H](O)CC=C(C=O)[C@@]12C=O
105	1121	C\C(CC\C=C(/C)CC[C@@H](O)C(C)(C)O)=C/CC[C@]1(C)OC2=C(C)C=C(O)C=C2C=C1
106	1565	C[C@H]1CC[C@]2(O)[C@@H]1CC[C@H](C)[C@@H]2C=C(C)C
107	846	CC1=CC(O)=C(C=C1)[C@]1(C)CC[C@](C)(O)C1=C
108	529	C[C@@H]1CC[C@H]2[C@@H]1[C@@H]1[C@H](C[C@@H](O)[C@]21C)C(=C)CCC=C(C)C
109	1437	C\C(=C/[C@@H](O)[C@H]1C(=C)CC=CC1(C)C)[C@@H]1CC[C@](C)(O)[C@@H](Br)C1
110	1569	CC[C@@]1(Br)[C@H](\C=C\C#C)[C@H]2[C@H]3C[C@H]4O[C@]1(Br)[C@@H]2[C@H]4O3
111	1144	CC[C@H]1O[C@@]2(C)[C@H](OC(C)=O)[C@]1(C)O[C@@H](\C=C\C=C\C=C\C1=C(C)C(OC)=CC(=O)O1)[C@H]2O
112	20	CC[C@H](C)[C@@H](O)[C@@](O)(\C=C\CC(=C)CCC(=O)C1=C(O)C[C@@H](C)OC1=O)C([O-])=O
113	355	CC1(C)[C@H](Br)CCC(=C)[C@@]11CC\C(=C/Br)C=C1
114	293	C[C@H]1C\C(C)=C\C=C\C(=O)O[C@@H](CC2=NC(=CS2)[C@@H](C)C[C@@H](N)CC(=O)O1)\C=C(/C)\C=C\C(\C)=C\CN(C)C
115	1152	CC1=CC[C@]2(CC1)C(=C)CC[C@H](Br)C2(C)C
116	621	CN[C@@H]1[C@H](O)[C@@H](O[C@H]2[C@H](N)C[C@@H](N)[C@H](O[C@@H]3O[C@H](CN)[C@H](O)[C@H](O)[C@@H]3N)[C@H]2O)OC[C@]1(C)O

117	508	C[C@@H]1CC[C@H]2[C@@H]1[C@@H]1[C@H](C[C@H](O)[C@]21C)C(=C)C[C@@H](O)[C@@H]1OC1(C)C
118	488	C[C@@]12C[C@](C)(CCCCCCCCCCCCC3=CC=CC=C3)OO[C@@H]1CC(=O)O2
119	1154	CC(=C)[C@H](O)[C@@H]1CC[C@@]2(C)[C@@H]1[C@](C)(O)CC[C@@H]2Br
120	710	CC(C)C1=C2CC[C@@H](C)[C@@H]2CC(C)(C)[C@@H]1O
121	175	C[C@@H]1CC[C@@]2(O)[C@H]1[C@H]1[C@@H](CCC2=C)C1(C)C
122	929	CC(=C)[C@H]1CC[C@]2(C)C[C@@H]3C(C)=CC[C@H](O)[C@@]3(C)CC[C@@H]12
123	133	CC[C@H]1O[C@H]2C[C@H](O[C@@H]2C\C=C\C#C)[C@@H](Cl)C[C@@H]1Br
124	1549	CC[C@]1(O)C[C@H](O[C@@H]2C[C@@H](O)[C@H](O[C@@H]3C[C@@H](O)[C@H](O[C@@H]4CC[C@H](O)[C@H](C)O4)[C@H](C)O3)[C@@H](C)O2)C2=C(O)C3=C(C=C2[C@H]1C(=O)OC)C(=O)C1=CC=CC(O)=C1C3=O
125	1058	BrC1=CC=C2C(NC=C2[C@@H]2CNC(=O)C(=N2)C2=CNC3=CC(Br)=CC=C23)=C1
126	265	CC(C)=CCC\C(C)=C\CC1=CC(O)=C(Br)C=C1O
127	354	CC(C)[C@H]1[C@@H]2CC(C)=C3CC[C@](C)(O)[C@@H]3[C@H]12
128	1137	C[C@@]12CC[C@H]3[C@@](C)(CC[C@H]4C(C)(C)[C@@H](Br)CC[C@]34C)OC[C@@H]1O2
129	1018	COC1=CC=C2C3=C(C(=O)OC2=C1)[C@@](C)(CC\C=C(/C)CC1=CC(C)=CO1)[C@H](C)O3
130	1318	C\C(CC\C=C(/C)CC[C@@H](O)C(C)(C)O)=C/CC\C(C)=C\CC1=CC(=O)C=C(C)C1=O
131	1118	CC(=O)OC(C)(C)[C@H]1CC[C@](C)(O1)[C@@H]1CCC(=C)[C@@H]2CC=C(C)[C@H]2[C@@H]1O
132	688	CC1=CC[C@H](Br)C(C)(C)[C@@H]1CC[C@](C)(O)C=C
133	495	C[C@@]12C[C@](C)(CCCCCCCCCCC3=CC=C(O)C=C3)OO[C@@H]1CC(=O)O2
134	580	CC(C)=CC[C@@H](O)C(\C)=C\CC[C@](C)(O)CCC\C(C)=C\CO
135	853	C[C@@H]1CC(=C)[C@@]2(CC\C(=C\Br)C=C2)C(C)(C)[C@@H]1Br
136	790	CC(C)=CCC\C(C)=C\CC[C@@]1(C)O[C@@H]1CC1=CNC(=C1)[N+]([O-])=O
137	164	CC[C@H]1O[C@@H]2C[C@@H](O[C@H]2C\C=C\C#C)[C@@H](C[C@@H]1Br)OC(C)=O
138	1380	C[C@H]1[C@H]2[C@H](CC3=CC=CC=C3)NC(=O)[C@]22OC(=O)O\C=C\[C@@](C)(O)C(=O)[C@@H](C)C\C=C\[C@H]2[C@@H]2O[C@]12C
139	956	C[C@@H]1CC=C2[C@H]1[C@H]1[C@@H](CC[C@]2(C)O)C1(C)C
140	374	CC\C(Br)=C\C=C/C1=C(Br)[C@H]2O[C@@H](C[C@H]2O1)C=C=CBr
141	1242	COC1=C(OC)C(C)=C(C\C=C(/C)CCCCCCC2=CC=CC=C2)OC1=O
142	643	C\C=C\[C@@H]1C\C=C/C[C@H](Cl)[C@H](C\C=C/C#C)O1
143	1645	CNCC1=CC[C@H](N)[C@H](O[C@H]2[C@@H](N)C[C@H](N)[C@H](O[C@@H]3OC[C@@](C)(O)[C@H](N)[C@H]3O)[C@@H]2O)O1
144	1478	C[C@@H]1CC[C@H]2[C@@H]1[C@@H]1[C@@H](C[C@@H](O)[C@]21C)C(=C)C[C@H](O)C=C(C)C
145	1534	CC[C@@H](Br)[C@H]1C[C@H](Br)[C@@H]2C[C@H](O1)[C@H](C\C=C/C#C)O2
146	722	CC(C)=C[C@@H]1CC[C@@]2(C)[C@@H]1[C@](C)(O)CC[C@@H]2Br
147	234	CC[C@@H](Br)[C@H]1C[C@H](Br)[C@H](O)C[C@H]2C[C@@H](C[C@H]2O1)C=C=CBr
148	167	C[C@H](CCC=C(C)C)[C@@H]1CCC(=C)[C@H]2CC=C(C)[C@@H]2[C@@H]1O
149	79	CC1=CC(O)=C(C=C1Br)[C@]1(C)CC[C@](C)(O)C1=C
150	1521	CC1=CC[C@H]2C(C)(C)CCC[C@]2(C)[C@H]1CC1=C(O)C=CC(O)=C1
151	1453	C\C(CC[C@H]1C(C)(C)CCC[C@]1(C)O)=C/CC\C(CO)=C\C[C@H]1OC(=O)C=C1CO
152	1259	CC(C)[C@H]1CCC[C@@](C)(O)[C@H]2C=C[C@H](C)[C@@H]12
153	783	CO[C@@H]1C[C@@]2(OC1(C)C)OC(C[C@]1(C)CCC3=CC(OC)=CC(C)=C3O1)=C[C@]1(C)CCC[C@]21C
154	395	OC1=CC(CN2[C@@H](CCC2=O)C([O-])=O)=C(Br)C(Br)=C1O

155	1514	C[C@H](CCC=C(C)C)[C@@H]1CC[C@@H](C)C2=C1C=C(C)C=C2
156	557	CC(C)[C@@H]1CC(C)(C)C[C@]2(O)[C@](C)(O)[C@H](Cl)C[C@@]12O
157	639	CC1(C)[C@@H](Br)CC[C@]2(C)O[C@H]3COC(=C)C3=CC[C@@H]12
158	1612	NCC[C@H](O)C(=O)N[C@@H]1C[C@@H](N)[C@H](O[C@@H]2O[C@H](CN)[C@H](O)[C@H](O)[C@H]2N)[C@H](O)[C@H]1O
159	1350	COC1=C(C\C=C(/C)[C@H]2C(=O)C[C@]3(C)CCC[C@]3(C)[C@]22OC(C)(C)C=C2)C=C(O)C=C1C
160	1267	CC1CCC2(CC1)[C@@H](C)CCCC2(C)C
161	1364	CC1(C)CC(=O)C=C2[C@H](O)[C@]3(Cl)C[C@]12CC[C@@]3(C)O
162	682	C[C@@H]1CC[C@]23[C@H]1CC[C@@]2(C)C[C@H](C)[C@@]3(C)O
163	1449	CC1(C)[C@H](Br)[C@H](O)CC(=C)[C@@]11CC\C(=C\Br)C=C1
164	598	CC(=C)[C@@H](O)CCC(=C)[C@]1(O)CC[C@](C)(Br)[C@@H](Cl)C1
165	1439	OCC1=C(CC2=CC(Br)=C(O)C=C2)C(O)=C(O)C(Br)=C1
166	292	C[C@](O)(CC[C@H]1C(=C)CC[C@H](Br)C1(C)C)C=C
167	558	CC(C)C1=C2CC[C@@]3(C)[C@@H](O)CCC(=C)[C@]3(O)C[C@]2(C)CC1
168	875	COC(=O)CCCN(CC1=C(Br)C(Br)=C(O)C(O)=C1)C(=O)NCC1=C(Br)C(Br)=C(O)C(O)=C1
169	995	C\C(=C\C[C@@H]1C(=C)CC=CC1(C)C)[C@H](O)CBr
170	1170	C[C@@]12CC[C@@H](O)[C@@]3(C)C1=C(O[C@@H]2O)C(=O)C1=C[C@](C)(CC[C@]31O)C=C
171	1039	C[C@](O)(CCCCCCCCCCCCC1=CC=CC=C1)C[C@]1(C)OC(=O)C[C@@H]1O
172	1289	CC(C)[C@@H]1N(C)C2=C3C(C[C@H](CO)NC1=O)=CNC3=C(CC1=C3NC=C4C[C@@H](CO)NC(=O)[C@H](C(C)C)N(C)C(C=C1)=C34)C=C2
173	1464	OCC1=CC(O)=C(O)C(Br)=C1CC1=CC(Br)=C(O)C(O)=C1
174	1467	CC(C)=CCC\C(C)=C\[C@H](O)[C@H](O)C(\C)=C\CC[C@@]1(C)OC2=C(C)C=C(O)C=C2C=C1
175	839	CC1=CC[C@H](Br)C(C)(C)[C@@H]1CC[C@@](C)(O)C=C
176	1098	C\C(CC\C=C(/C)CCC(=O)C(C)(C)O)=C/CC\C(C)=C\CC1=CC(=O)C=C(C)C1=O
177	1370	C[C@H](CCC=C(C)C)[C@@H]1CC[C@@]2(C)CCC=C(C=O)[C@@]12C=O
178	1542	C[C@@H]1CC=C(C)[C@@H]2CC[C@](C)(O[C@@]12C)C=C
179	996	CC(C)[C@H]1[C@@H]2C[C@@H](C)[C@]3(O)[C@@H](Br)CC(C)=C3[C@H]12
180	1174	CC(C)=CC[C@@H](O)C(\C)=C\CC\C(C)=C\CC\C(C)=C\CO
181	1410	CC1(C)C[C@H](CC[C@@H]1Br)[C@@]12CC[C@@](O)(CO1)[C@@H](Cl)[C@H]2O
182	515	CC1(C)[C@H](Br)CC[C@@]2(C)[C@@H]3CC[C@@](C)(O[C@@]3(C)C[C@H](O)[C@H]12)C=C
183	651	C[C@@]1(CC=C2[C@@H](C1)[C@H](O)C[C@@H]1[C@@]3(CO)C[C@H]3[C@H](O)C[C@@]21C)[C@H]1CO1
184	390	CC1(C)CC(=O)C=C2C(=O)[C@]3(Cl)C[C@]12CC[C@]3(C)Cl
185	235	C[C@@H]1CCC(=O)[C@@H]2C=C[C@@H]3[C@@H](C3(C)C)[C@@]12C
186	1201	C[C@H]1\C(CC[C@@]1(C)C1=C(O)C=C(C)C=C1)=C\Br
187	1490	CC(C)[C@@H]1CC(C)(C)[C@@H](O)C2=C1CC[C@H]2C
188	1414	C[C@@](O)(CC[C@H]1C(=C)[C@@H](Br)C[C@@H](Br)C1(C)C)C=C
189	1473	CC1(C)[C@@H](Br)[C@@H](O)C=C2C[C@]3(C)CC[C@]12C[C@@H]3Cl
190	957	C[C@H]1CC=C(C)[C@@]23CC[C@@](C)(O[C@]12C)[C@@H](Br)C3
191	190	C[C@H](CC[C@@H](O)C(C)=C)[C@@H]1CC[C@@]2(C)[C@@H](O)CC=C(C=O)[C@@]12C=O
192	1678	CO[C@H]1C[C@@H](N)[C@H](O[C@@H]2O[C@@H](CC[C@@H]2N)[C@@H](C)N)[C@@H](O)[C@H]1N
193	794	CC1=CC[C@]2(CC1)C(=C)CC[C@@H](Br)C2(C)C
194	889	C[C@@H]1CC[C@]23[C@H]1CC[C@@]2(C)C[C@H](C)[C@]3(C)O

195	917	CN1CN([C@H]2C[C@@H](O)[C@@H](CO)O2)C(=O)NC1=O
196	511	C[C@H]1CC[C@@H]2[C@@]3(C)CC[C@H](O)C(C)(C)[C@@H]3CC[C@@]2(C)[C@]11CC2=C(O1)C(C)=CC(O)=C2O
197	1266	OC1=CC=C(CN2CC3=C(C[C@H]2C([O-])=O)C2=CC=CC=C2N3)C=C1
198	567	CC(C)=CC[C@H](O)C(\C)=C\CC\C(C)=C\CC\C(C)=C/C=O
199	923	C[C@H]1CC[C@](C)(C1=C)C1=C(O)C=C(C)C(Br)=C1
200	885	CC(C)[C@H]1CC(C)(C)[C@@H](O)C2=C1CC[C@H]2C
201	802	CC1=CC=C(C=C1)[C@]1(C)CC[C@@H](Br)C1(C)C
202	1064	OC1=CC(COCC2=CC(O)=C(O)C(Br)=C2Br)=C(Br)C(Br)=C1O
203	763	CC[C@H]1O[C@H](C[C@@H](O)[C@H](O)\C=C/1)[C@H](Cl)C\C=C/C#C
204	496	CC(=O)O[C@H]1CC[C@@]2(C)[C@@H](CC[C@@]3(C)[C@H]2CC[C@]2(C)OC4=C(C)C=C(OC(C)=O)C=C4C[C@@H]32)C1(C)C
205	950	CC1=CC[C@]2(CC1)C(C)=CC[C@H](Br)C2(C)C
206	1544	CC(C)=CC[C@H](O)C(\C)=C\CC\C(C)=C\CC\C(C)=C\C=O
207	955	CC[C@@H](C)[C@H]1[C@](C)(O)[C@]2(O)[C@@H]3C[C@](C)(O)C[C@@H](C)[C@@H]3[C@@]1(C)C(=O)\C2=C/O
208	977	C[C@@H]1CCC(=C)[C@H]2CC[C@](C)(O[C@@]12C)C=C
209	1145	CC[C@H]1O[C@H]2C\C=C/C[C@@H](O[C@H]2C[C@@H]1Br)C=C=CBr
210	423	BrC1=CNC2=CC(Br)=C(Br)C=C12
211	1001	C[C@@H]1CC[C@@]23[C@H](C)[C@](C)(O)C[C@]2(C)CC[C@@H]13
212	393	CN[C@@H]1[C@@H](O)[C@@H](O[C@@H]2O[C@H](CN)CC[C@@H]2N)[C@H](N)[C@@H](O)[C@@H]1OC
213	1089	CC(C)[C@H]1CC(C)(C)C[C@]23O[C@]12C[C@H](Br)[C@@]3(C)O
214	810	C[C@@H]1CC[C@@](C)(C1=C)C1=C(O)C=C(C)C=C1
215	1584	CO[C@H](C)[C@@H]1[C@H](C)C[C@@H]2[C@H]3C[C@H]4\C=C\C(\O)=C5\C(=O)N[C@@H]([C@H](O)CCNC(=O)\C=C/C[C@H]4[C@H]3[C@@H]3O[C@@H]3[C@@H]12)C5=O
216	1361	CC1=C[C@H]2O[C@@]2(C)[C@]2(C[C@H](Br)[C@@](C)(Cl)C[C@H]2O)C1(C)C
217	1135	CC(=C)[C@H]1CC[C@]2(C)CC3=C(C)CC[C@H](O)[C@@]3(C)CC[C@@H]12
218	1006	C[C@]12CC[C@H](Br)C(C)(C)[C@@H]1C[C@H](O2)C1=C(O)C(=O)OC1
219	1399	C[C@@H]1CCCC2=CC(=O)[C@@H]3[C@@H](C3(C)C)[C@@]12C
220	1684	CN[C@H]1[C@@H](O)[C@H](O[C@H]2[C@H](N)C[C@@H](N)[C@H](O[C@@H]3OC(CN)=CC[C@H]3NC=O)[C@H]2O)OC[C@@]1(C)O
221	1426	CC[C@H](C)CCCCCCCCCC\C(O)=C1/C(=O)[C@@H](C)N(C)C1=O
222	294	CC(=O)O[C@H]1CC[C@@]2(C)[C@@H](CC[C@@]3(C)[C@H]2CC[C@]2(C)OC4=C(C)C=C(O)C=C4C[C@@H]32)C1(C)C
223	1309	CC1(C)S[C@@H]2[C@H](NC(=O)CC3=CC=C(O)C=C3)C(=O)N2[C@@H]1C([O-])=O
224	1003	C\C(=C/C[C@H]1C(=C)CC[C@@H](Br)C1(C)C)[C@H](O)CBr
225	1384	CC1=CC[C@H](Br)C(C)(C)[C@@]11CC[C@@](C)(Cl)[C@H](Br)C1
226	539	CC1CC[C@H]2C(C)(C)CCC[C@]2(C)[C@H]1CC1=CC(=O)C=CC1=O
227	318	CC(=O)N(CCC1=CNC2=CC=CC=C12)C(C)=O
228	603	CC(C)=CCC\C(C)=C\CC[C@](C)(Cl)[C@H](O)CC1=CNC(=C1)[N+]([O-])=O
229	211	CC1=CC(=O)CC(C)(C)[C@]11CC\C(=C\Br)C=C1
230	50	CCCCCCCCC[C@@H](C)CCC\C(O)=C1/C(=O)[C@@H](C)N(C)C1=O

231	4	CC1(C)[C@@H](Br)[C@@H](O)CC(=C)[C@@]11CC\C(=C/Br)C=C1
232	490	COC(=O)[C@@H]1CCC(=O)N1CC1=C(CC2=C(Br)C(Br)=C(O)C(O)=C2)C(Br)=C(O)C(O)=C1
233	609	CN1C=C(Br)C2=CC(Br)=C(Br)C=C12
234	787	C\C(=C/C[C@H]1C(=C)CC=CC1(C)C)[C@H](O)CBr
235	157	CC1(C)[C@H](Br)[C@H](O)CC(=C)[C@@]11CCC(=O)C=C1
236	1382	O[C@@H]([C@@H]1O[C@H]1[C@@H]1O[C@H]1C=C=CC#CC#C)[C@@H]1CCC(=O)O1
237	1457	CC1(C)CCC[C@]2(C)OC3=CC(Br)=C(O)C=C3C[C@@H]12
238	895	CC[C@H]1O[C@H](C\C=C/C[C@@H]1Br)[C@@H](C\C=C\C#C)OC(C)=O
239	657	C[C@@H](CC[C@@]1(O)[C@](C)(O)CC[C@H]2C(C)(C)CCC[C@]12C)C=C
240	210	CC1=CC=C(C=C1)[C@]1(C)CC[C@H](Br)C1(C)C
241	1045	CC1(C)C[C@H](CC[C@@H]1Br)[C@]12CC[C@@](Cl)(CO)[C@H](O)[C@H]1O2
242	991	C[C@@H]1CCC(=O)C2=CC[C@@H]3[C@@H](C3(C)C)[C@@]12C
243	554	C[C@H]1[C@@H](Br)[C@H](O)C(C)=C2CC[C@](C)(Cl)[C@@H](Br)C[C@]12C
244	620	C[C@]12CC[C@H](Br)C(C)(C)[C@@H]1C[C@H](O2)C1=COC=C1
245	553	C[C@H]1C(=C)CC[C@@]1(C)C1=C(O)C=C(C)C(Br)=C1
246	149	CC1CC[C@H]2C(C)(C)CCC[C@]2(C)[C@H]1CC1=C(O)C=CC(O)=C1
247	65	COC1=C(OC)C(=O)OC(CCCCCCCCCC[S@@](C)=O)=C1C
248	756	C[C@H]1C(=C)CC[C@@]1(C)C1=CC=C(C)C=C1
249	945	C[C@@]1(O)CC[C@]2(C=C1)C(=C)C[C@H](O)[C@@H](Br)C2(C)C
250	1703	CN[C@@H]1[C@@H](O)[C@@H](O[C@@H]2O[C@H](CN)CC[C@@H]2N)[C@H](N)C[C@@H]1OC
251	1557	C\C(CC[C@@H](O)C(C)(C)O)=C/CC[C@@](C)(Cl)[C@H](O)CC1=CNC(=C1)[N+]([O-])=O
252	1517	C[C@H](CCC=C(C)C)[C@H]1CC\C(C)=C/CCC2=C1C(=O)OC2
253	601	CC1(C)[C@@H](Br)[C@@H](O)CC(=C)[C@]11CCC(=O)C=C1
254	858	C[C@@]1(CC=C2[C@@H](C1)[C@H](O)C[C@@H]1[C@@]3(CO)C[C@H]3[C@H](O)C[C@@]21C)[C@@H](Br)CO
255	1452	CC1=C(C)[C@@](C)(CC1)C1=C(O)C=C(C)C=C1
256	1230	C[C@H]1C(=O)C[C@@H](Br)C(C)(C)\C1=C\C[C@](C)(O)C=C
257	1186	CC(C)(O)\C=C\C[C@]1(C)[C@@H](Br)CC[C@](C)(O)[C@H]1CC[C@](C)(O)C=C
258	727	CC(C)[C@@H](Br)[C@@H]1C[C@@H](C)C2=CC=C(C)C2=C1
259	445	C[C@@H]1CCC=C2[C@@H](O)C[C@@H]3[C@@H](C3(C)C)[C@@]12C
260	582	CC(=C)[C@H]1CC[C@]2(C)O[C@@H]2[C@H]1CC(=C)[C@@H]1CC[C@](C)(O)[C@H](Br)C1
261	78	OCS\C=C1\C=C(O)C(=O)C(Br)=C1Br
262	1697	CN[C@H]1[C@H](O)[C@@H](O[C@H]2[C@@H](O)C[C@@H](N)[C@@H](O[C@@H]3O[C@H](CN)[C@@H](O)C[C@@H]3N)[C@H]2O)OC[C@]1(C)O
263	159	CC1=CC[C@H]2C(C)(C)CCC[C@]2(C)[C@H]1CC1=CC(=O)C=CC1=O
264	823	BrC1=C(C2=CC(Br)=C(Br)C=C2N1)C1=C(Br)NC2=CC(Br)=C(Br)C=C12
265	441	C[C@H]1CC[C@](C)(C1=C)C1=C(O)C=C(C)C=C1
266	818	C[C@H]1[C@@H](Br)[C@H](O)C(C)=C2CCC(C)=CC[C@]12C
267	972	CC1(C)S[C@H]2[C@H](NC(=O)CSC[C@@H](N)C([O-])=O)C(=O)N2[C@H]1C([O-])=O
268	454	NC1=N[C@H](O)[C@H]2[C@H]3O[C@]4(O)O[C@@H]([C@H](O)[C@@]2(N1)[C@@H]4O)[C@]3(O)CO
269	1371	CC1(C)CC(=O)C=C2C(=O)[C@]3(Cl)C[C@]12CC[C@@]3(C)Cl
270	630	CC1=C(C)[C@](C)(CC1)C1=CC(O)=C(C)C=C1
271	532	CC(C)[C@@H]1CCC(C)=C2CCC(C)=C[C@@H]12

272	751	CC(C)[C@@]1(O)CC[C@@]2(C)C[C@H]3C(C)=CC=C[C@@]3(C)C(=O)C[C@H]12
273	656	COC1=CC=C2C(NC(=O)[C@@]22C=C3N([C@H]2C=C(C)C)C(=O)[C@@H]2CCCN2C3=O)=C1
274	1351	CCCCCCCC[C@@H]1OC(=O)[C@@H]2OC(=O)C(=C)[C@H]12
275	847	CCC1=C(C2=C\C(C(=O)N2)=C2\C=C(NC2=O)C2=C(CC)C(C)=C3C([C@H](C)C[C@]3(C)O)=C2C)C(C)=C2[C@@H](C)C[C@@](C)(O)C2=C1C
276	1605	CN[C@H]1[C@@H](O)[C@H](O[C@H]2[C@H](N)C[C@@H](N)[C@H](O[C@@H]3OC(CN)=CC[C@@H]3O)[C@@H]2O)O[C@@H](CO)[C@@]1(C)O
277	1111	C\C(\C=C\[C@]1(O)C(=C)C[C@@H](O)CC1(C)C)=C/C([O-])=O
278	1209	C[C@@H]1CCC=C2CC[C@@H]3[C@@H](C3(C)C)[C@@]12C
279	998	C[C@@H]1CC[C@](C)(C1=C)C1=CC=C(C)C=C1
280	1334	CC(C)[C@@]1(O)CC[C@@]2(C)C[C@@H]3C(C)=CC=C[C@]3(C)C(=O)C[C@H]12
281	1188	C[C@H]1CC=C(C)[C@@]23CC[C@@](C)(O[C@]12C)[C@H](O)C3
282	855	CC(C)CCCCCCCCCCC\C(O)=C1/C(=O)[C@@H](C)N(C)C1=O
283	47	CCC[C@@H](O)C1=C(Br)[C@](CI)(OC)OC1=O
284	1014	C[C@@]12O[C@@H]1C[C@H]1C(C)(C)[C@@H](Br)CC[C@]1(C)O[C@H]2CBr
285	1081	CC(C)[C@H]1CC(C)(C)C[C@]23O[C@]12C[C@H](Cl)[C@@]3(C)O
286	51	CCCCC1=C(Br)[C@@](OC)(OC1=O)C(Br)Br
287	1017	C[C@@H]1CC=CC2=CC[C@@H]3[C@@H](C3(C)C)[C@@]12C
288	1284	C[C@@](Br)(CCl)[C@@H]1C[C@H](O)[C@@](C)(O1)C(\Cl)=C/Br
289	1035	CC(=O)O[C@](C)(C=C)\C=C\[C@@]12O[C@]1(C)CCCC2(C)C
290	194	C\C(CC1=C[C@@]2(C)CCC[C@@]2(C)[C@]2(C[C@H](O)C(C)(C)O2)O1)=C/CC1=C(O)C(C)=CC(O)=C1
291	317	C[C@]12C[C@@H](O)CC(C)(C)[C@]1(O)CC(=O)O2
292	310	C[C@@H]1CC[C@H]2[C@@H]1[C@@H]1[C@H](C[C@H](O)[C@]21C)C(=C)C[C@@H](O)C=C(C)C
293	1303	CC1(C)[C@@H](Br)[C@H](O)CC(=C)[C@@]11CCC(=O)C=C1
294	440	CCC[C@@H](O)C1=C(Br)[C@@](CI)(OC)OC1=O
295	976	NC(=N)NCCC[C@H](NC(=O)N[C@@H](CC1=CC=CC=C1)C([O-])=O)C([O-])=O
296	296	CC(C)=C[C@@H](O)C\C(C)=C1/CC[C@](C)(Cl)[C@@H](Br)C1
297	593	CC(C)CCC[C@@H](C)CCC[C@H](C)CCC\C(C)=C\CO
298	577	CC1=CC=C(C)C(CC[C@@](C)(O)C=C)=C1C
299	1221	C[S@](=O)C1=C(Br)NC2=CC(Br)=CC(Br)=C12
300	223	NC(=O)OC[C@H]1C2=C(O)C=C(C=O)C=C2N2C[C@@H]3N[C@H]3[C@@]1(O)O2
301	394	CC(=C)[C@H]1C[C@@]2(C)C[C@H](O2)[C@H]1CC(=C)[C@@H]1CC[C@](C)(O)[C@H](Br)C1
302	1342	CCC\C=C\C\C=C\C=C\C=N/N=N/O
303	1604	CN[C@H]1[C@H](C[C@@H](N)[C@H](O[C@@H]2O[C@@H](CC[C@@H]2N)[C@@H](C)N)[C@H]1O)OC
304	1271	C[C@H]1[C@@H](O)C(=O)C(C)=C2[C@H](O)CC(C)=CC[C@]12C
305	121	CC(C)=CCC\C(C)=C\C\C=C(\C)[C@H](O)CBr
306	1277	CC1(C)[C@@H](Br)C[C@H](Cl)\C(=C\CCl)[C@@H]1Cl
307	331	CC\C(Br)=C1/O[C@H]2C[C@@H]3O[C@H]2[C@H]1[C@@H]3[C@@H](Cl)\C=C/C#C
308	564	CC(C)=CC(=O)C\C(C)=C\CC\C(C)=C\CC\C(C)=C/C=O
309	673	COC(=O)[C@H](CC1=C(Br)C(Br)=C(O)C(O)=C1)C1=CC(Br)=C(OC)C(O)=C1
310	1275	C[C@@H]1CC[C@]23[C@H]1CC[C@@]2(C)CC(C)=C3C
311	738	COC(=O)CC[C@@H]1C(CC[C@@H]2[C@@](C)(CC3=C(O)C(C)=CC(O)=C3)[C@@H](C)CC[C@@]12C)=C(C)C

312	566	C[C@H]1C(=O)[C@H](Br)[C@H](C)[C@@]2(C)C[C@H](Br)[C@]3(C)CC[C@]123
313	1338	CO[C@H]1O[C@H]2OC[C@H](CCC=C(C)C)[C@@H]3CC\C(C)=C/C\C=C1/[C@@H]23
314	91	CCCCC[C@@H](O)CCCCCCCCCCCCCC(=O)C1=C(O)C=C(O)C=C1O
315	297	C[C@H]1CC(=O)O[C@H]1[C@@]1(C)CC(=O)C2=C(O)C(=CC=C2O1)C1=C(C)C=C2O[C@@](C)(CC(=O)C2=C1O)[C@@H]1OC(=O)C[C@H]1O
316	1088	C[C@@]1(C[C@](Cl)(CBr)[C@@H](Br)C[C@H]1Cl)\C=C\Cl
317	270	COC(=O)CCCNC(=O)NCC1=C(C[C@H]2CC(O)=C(O)C(Br)=C2Br)C(Br)=C(O)C(O)=C1
318	326	N[C@H](C(=O)N[C@H]1[C@H]2SCC(Cl)=C(N2C1=O)C([O-])=O)C1=CC=CC=C1
319	6	CC1=CC=C2C(O[C@H]3CC[C@]2(C)C3(C)C)=C1
320	1409	CCC[C@]1(CO1)C1=C(Br)\C(OC1=O)=C\Br
321	1653	CNC[C@H]1CC[C@H](N)[C@H](O[C@H]2[C@@H](N)C[C@H](N)[C@@H](O[C@@H]3O[C@H](CO)[C@@](C)(O)[C@H](NC)[C@H]3O)[C@@H]2O)O1
322	468	COC(=O)CCCNC(=O)NCC1=C(Br)C(Br)=C(O)C(O)=C1
323	1353	C[C@H]1C(=C)CC[C@@]1(C)C1=CCC(C)=CC1
324	568	C[C@H]1C[C@](C)(C[C@H]([C@@H](O)CC2CC(=O)NC(=O)C2)C1=O)OC(C)=O
325	1099	CC1(C)[C@@H](Br)C[C@H](Cl)\C(=C/CBr)[C@@H]1Cl
326	866	CN1C(=O)N(C)[C@]2(OC3=C(O)C=C4C(CC[N+]4(C)C)=C3C3=C2C(=O)C2=C(NC=C2CCN)C3=O)C1=O
327	74	COC(OC)[C@H](C)CC1=CC(O)=C(O)C(Br)=C1Br
328	383	C[C@H]1CC(=O)[C@@H](C)[C@]23CC[C@@]2(C)[C@@H](Br)C[C@]13C
329	1349	CC1(C)[C@@]2(Br)O[C@@H]3C[C@](C)(Cl)[C@@H](Br)C[C@@]13[C@](C)(O)C=C2
330	719	C[C@@H](CO)CC1=CC(O)=C(O)C(Br)=C1Br
331	687	C[C@]1(Cl)C[C@@](C)(\C=C\Cl)[C@H](Br)C[C@@H]1Cl
332	1194	CC1=C(N2[C@H](SC1)[C@@H](NC(=O)[C@@H](N)C1=CC(NS(C)(=O)=O)=CC=C1)C2=O)C([O-])=O
333	1324	CC[C@]1(OC)C(=O)NC2=CC=CC=C12
334	1180	CC1=C2C(C)=C(CO)OC(=O)C2=C(O)C=C1O
335	1129	CC1=CC(=O)CC(C)(C)[C@]11CCC(=O)C=C1
336	1085	C[C@H]1CC[C@@H]2[C@@]3(C)CCC(=O)C(C)(C)[C@@H]3CC[C@@]2(C)[C@]11CC2=C(O1)C(C)=CC(OC(C)=O)=C2OC(C)=O
337	891	C[C@@]1(Cl)C[C@@](C)(\C=C\Cl)[C@H](Cl)C[C@H]1Cl
338	1345	C[C@H](CCC=C(C)C)[C@H]1[C@H](O)C[C@@H](C)C[C@@H]2C[C@@H]2C2=C1COC2=O
339	1288	CC1=CC[C@@H](O[C@H]1C(Br)Br)[C@@](C)(Cl)CCl
340	411	CC1(C)O[C@](C)([C@@H](O)C[C@H]1Br)[C@H]1CC[C@@](C)(Cl)[C@H](Br)C1
341	1227	CC(=O)OC[C@H](Br)[C@@]1(C)CC=C2[C@H](C1)[C@@H](O)C[C@@H]1[C@]3(CO)C[C@@H]3[C@H](C[C@@]21C)OC(C)=O
342	901	CC1(C)[C@@H](Br)C[C@H](Cl)\C(=C/CCl)[C@@H]1Cl
343	1002	COC1=C(OC)C(=O)OC(CCCCCCCCCCSC)=C1C
344	1238	O[C@H]1CCC(=O)[C@H]2C[C@@]34SS[C@]5(C[C@H]6[C@H]([C@@H](O)CCC6=O)N5C3=O)C(=O)N4[C@@H]12
345	1344	CC(=O)O[C@]1(C[C@@H]2CC[C@]34[C@H](O)O[C@](C)(CC[C@@H]3Br)[C@@H]24)CC[C@H](Br)C(C)(C)C1
346	645	CC(=O)OC[C@H](Br)[C@@]1(C)CC=C2[C@H](C1)[C@@H](O)C[C@@H]1[C@]3(O)CC[C@@H]3[C@H](C[C@@]21C)OC(C)=O
347	1479	CC1(C)C=CC(=O)C(=C1)[C@@H](Br)CBr

348	747	CC1(C)CCC[C@@]2(C)[C@H]1CCC1=CC3=C(C=C21)C(=O)C=CC3=O
349	944	C[C@@H](CCC=C(C)C)[C@@H]1CC=C(C=O)[C@@H]2CC=C(C)[C@H]2[C@@H]1O
350	865	CC\C=C\C\C=C/C[C@@H](OC(C)=O)[C@@H](Cl)C\C=C/C#C
351	314	C[C@@H]1CC[C@H]2[C@@H]1[C@@H]1[C@@H](C[C@@H](O)[C@]21C)C(=C)[C@@H]1O[C@@H]1[C@@H]1OC1(C)C
352	1331	COC1=CC=CC2=C1[C@H](O)[C@@]1(O)C=CC3=C([C@@H]1[C@H]2O)C(=O)C[C@H](C)C3
353	1319	C[C@H](CCC=C(C)C)[C@@H]1[C@H](O)CC(=C)[C@@H]2CC=C(C)[C@@H]2[C@@H]1O
354	971	CC(C)C1=C2[C@H](O)C[C@]3(C)[C@H](O)CCC(=C)[C@@]3(O)C[C@@]2(C)CC1=O
355	1559	[O-][N+](=O)C1=C(NC(Cl)=C1Cl)[C@@H]1OCOC2=C1C=C(Cl)C=C2Cl
356	449	CC(=O)\C=C\[C@H]1C(=C)CC[C@@H](Br)C1(C)C
357	410	O[C@H]1CO[C@@]2(O)[C@@H]1OC(=O)[C@]2(O)CC1=C(Br)C(Br)=C(O)C(O)=C1
358	17	CN[C@H]1[C@H](C[C@@H](N)[C@H](O[C@@H]2OC(CN)=CC[C@H]2N)[C@@H]1O)OC
359	800	C[C@H]1[C@@H](O)C(=O)C(C)=C2CCC(C)=CC[C@]12C
360	438	CC[C@@H]1O[C@@]2(O[C@@H]3C[C@H](O[C@@H]3[C@H]2Br)C=C=CBr)[C@H]2C[C@@H]12
361	1574	C[C@@H]1[C@H]2C[C@H](O)\C=C/C(/C)=C\C[C@H](O)\C=C/C(/C)=C\[C@H](NC(=O)C(C)=O)[C@](C)(C(=O)O2)C1=O
362	1360	C[C@H](O)[C@H]1[C@@H]2CC(SCCNC(=O)CCNC(=O)[C@@H](O)C(C)(C)CO)=C(N2C1=O)C([O-])=O
363	705	CC(=O)O[C@@H]1C[C@]2(C)O[C@@H](O)C(=O)[C@]2(O)C(C)(C)C1
364	130	COC(=O)CC[C@@H]1C(=O)CC[C@@H]2[C@@](C)(CC3=C(O)C(C)=CC(O)=C3)[C@@H](C)CC[C@@]12C
365	1224	CC1(C)[C@H](Br)[C@H](O)CC(=C)[C@@]11CC[C@](C)(Br)[C@@H](Cl)C1
366	666	CC(=O)\C=C\C1=C(Br)C=C(O)C(O)=C1
367	443	CCC[C@@H](O)C1=C(Br)\C(OC1=O)=C\I
368	769	CC1(C)C=CN2[C@@H](C[C@@]11NC3=CC=CC=C3C1=O)C(=O)N1CCC[C@]1(O)C2=O
369	791	C[C@H]1[C@H](O)C(=O)C(C)=C2CCC(C)=CC[C@]12C
370	1682	COC1[C@@H](O)[C@H](O)C(NC(=O)C(\C)=C/C2=CC(O)=C(O[C@@H]3O[C@H]([C@H](O)[C@H]3O)C(C)=O)C=C2)[C@H](O)[C@@H]1O
371	1166	C[C@]12C[C@H](O)[C@@H]3C[C@H]1O[C@]1(C[C@@H](Br)C(C)(C)O[C@]31C)O2
372	1189	COC1=C(OC)C(C)=C(CCCCCCCCCCSC(C)=O)OC1=O
373	1029	C[C@@]12O[C@@H]3C[C@]1(CC[C@@]3(C)Cl)C(C)(C)C(Br)=C[C@H]2O
374	1263	NCC1=C(Br)C(Br)=C(O)C(O)=C1
375	1124	CCOC(=O)O[C@H](C)OC(=O)[C@@H]1N2[C@H](SC1(C)C)[C@@H](NC(=O)[C@H](N)C1=CC=CC=C1)C2=O
376	1450	CCC(C)(C)[C@H](C)CC[C@@H](C)[C@H]1CC[C@H]2[C@@H]3C[C@H](OS([O-])(=O)=O)[C@H]4C[C@H](OS([O-])(=O)=O)[C@H](C[C@]4(C)[C@H]3CC[C@]12C)OS([O-])(=O)=O
377	1068	OCC1=CC(O)=C(O)C(Br)=C1Br
378	1485	CC(C)=C(Br)CC\C(=C\Br)C(Cl)=C
379	138	CC(=C)[C@H]1CC[C@]2(C)C[C@@H]3[C@](C)(O)CC[C@H](O)[C@@]3(C)CC[C@@H]12
380	1411	NC1=N[C@@]23[C@@H](N1)[C@H](CO)N=C(N)N2CCC3(O)O
381	200	CC(C)[C@H](N(C)C(=O)[C@@H](C)CCCCC#C)C(=O)N(C)[C@@H](C(C)C)C(=O)N(C)[C@@H](C(C)C)C(=O)N(C)[C@@H](CC1=CC=CC=C1)C(N)=O
382	1638	COC(=O)[C@]1(O)[C@H](Cl)C(=O)C(Cl)=C1\C=C\C
383	1715	C[C@@H]([C@H]1O[C@@]2(CC[C@@H](C)[C@@H](CC3=NC4=C(O3)C=CC(O)=C4C([O-])=O)O2)CC[C@@H]1C)C(=O)C1=CC=CN1

384	111	C[C@@]12C[C@@H](O)CC(C)(C)[C@@]1(O)CC(=O)O2
385	986	C[C@H]1C(=O)C=C(C)[C@@]2(C)C[C@H](Br)[C@]3(C)CC[C@]123
386	1469	C[C@@H]1[C@H](O)C(=O)[C@@H](C)[C@]23CC[C@H](C[C@@]12C)C3=C
387	1404	C\C(C(=O)CBr)=C1/C[C@@H]2[C@](C)(CC[C@H](Br)C2(C)C)O1
388	1071	COC(=O)[C@@H]1CCC(=O)N1CC1=C(Br)C(Br)=C(O)C(O)=C1
389	669	CS(=O)(=O)CC1=CC(O)=C(O)C(Br)=C1Br
390	708	CC1(C)[C@H](Cl)C=C\C(=C/CCl)[C@@H]1Cl
391	1476	C[C@@](Cl)(C=C)[C@H](Cl)\C=C\C(=C\Cl)\C(Cl)Cl
392	387	CC(=C)[C@H](O)COC1=CC=C(CCO)C=C1
393	1020	COC1=CC(=O)C2=C(C(O)=C3O[C@@]4(OC5=C(O)C6=C(C=C(C)OC6=O)C=C5C[C@@H]4O)[C@@H](O)C 3=C2O)C1=O
394	216	CC\C=C\C\C=C/C[C@H]1O[C@H](C[C@H]1OC(C)=O)C=C=CBr
395	555	OC1=CC(O)=CC(O)=C1
396	174	CC1(C)S[C@H]2[C@H](NC(=O)CCCCO)C(=O)N2[C@H]1C([O-])=O
397	253	CCCCCCCC\C=C(/Br)[C@H](O)C(Br)Br
398	1579	CC[C@]1(SC(=O)C(CC(N)=O)=C1O)\C=C(\C)C=C
399	140	CC1(C)[C@@]2(Br)O[C@H]3C[C@](C)(Cl)[C@@H](Br)C[C@@]13[C@](C)(O)C=C2
400	578	CC[C@H]1O[C@H](C[C@@H](O)[C@H](OC(C)=O)\C=C/1)[C@H](Cl)C\C=C/C#C
401	21	C[C@@]12CC[C@H](Br)[C@]3(CC[C@@H](C[C@@]4(O)CC[C@H](Br)C(C)(C)C4)[C@H]13)[C@@H](O)O2
402	368	CCCCCCCC1=CC(=O)C2=C(N1)C=CC=C2
403	1080	CC1(C)C=CC(=O)C(=C1)[C@@H](Cl)CCl
404	1716	CO[C@@H]1O[C@@H](OC)\C2=C\C\C=C(C)/CC[C@H]([C@@H](C)CCC=C(C)C)[C@@H]12
405	1274	CC1(C)C=CC(=O)C(=C1)[C@@H](Cl)CO
406	1127	CC(C)[C@@]1(O)CC[C@@]2(C)C[C@H]3[C@](C)(O)C=C[C@H](O)[C@]3(C)C(=O)C[C@H]12
407	362	CC(=C)[C@H]1CC[C@]2(C)C[C@@H]3C(=C)CC[C@H](O)[C@@]3(C)CC[C@@H]12
408	1672	CO[C@@H]1O[C@@H](OC)\C2=C\C[C@@H]3O[C@]3(C)CC[C@H]([C@@H](C)CCC=C(C)C)[C@@H]12
409	379	COC1=CC(C\C=C(/C)CC2=C[C@]3(C)CCC[C@]3(C)[C@@]3(C[C@@H](O)C(C)(C)O3)O2)=C(O)C(C)=C1
410	744	C[C@H](CCC=C(C)C)[C@H]1CC\C(C)=C/C\C=C(C=O)/[C@@H]1C=O
411	556	C\C=C\C1=CC(=O)[C@H](Cl)[C@@]1(O)C([O-])=O
412	1606	CN[C@H]1[C@@H](O)[C@H](O[C@H]2[C@H](N)C[C@@H](N)[C@H](O[C@@H]3O[C@@H]([C@H](C)N)[C@H](O)[C@@H](O)[C@@H]3N)[C@H]2O)OC[C@]1(C)O
413	974	CC(C)[C@H](O)COC1=CC=C(CC([O-])=O)C=C1
414	389	CCCCCCCCCCCCCCCC(=O)N[C@H]1CCOC1=O
415	180	CC(C)[C@@H]1CC=C[C@H]2[C@@H]3[C@](C)(O)C(=O)C=C[C@@]3(C)CC[C@]12CBr
416	1468	CC(C)=C(Br)CC\C(=C\Cl)C(Cl)=C
417	828	CN1C(Br)=C(Br)C2=CC(Br)=C(Br)C=C12
418	1092	OCC1=CC(O)=C(O)C(Br)=C1
419	547	CC(C)CC1=CC2=C(CC=C2C)C(C)=C1
420	739	COC1=C(C)C(=O)C2=C([C@@H](C)CC[C@H]2[C@@H](C)\C=C\C=C(C)C)C1=O
421	182	C[C@]1(Cl)CC[C@]2(C[C@@H]1Br)C(=C)CC[C@@H](Br)C2(C)C
422	759	CC[C@H]1O[C@@H](C[C@@H](Cl)[C@H](O)[C@H]1CBr)[C@H](Cl)C\C=C/C#C
423	712	CC(C)(Cl)[C@@H](Br)CC\C(=C\Br)C(Cl)=C
424	967	C[C@H](CCC=C(C)C)[C@@H]1[C@H](O)C\C(C)=C/C\C=C(C=O)/[C@H]1C=O

425	697	COCC1=C(CC2=C(Br)C(Br)=C(O)C(O)=C2)C(O)=C(O)C(Br)=C1Br
426	503	OC1=CC(Br)=C(C=O)C=C1O
427	356	CC(C)[C@H](N)C(=O)NC[C@H]([C@H](O)C([O-])=O)[C@@H]1CN2[C@@H](CC2=O)O1
428	652	[O-]C(=O)[C@@H]1N=C1\C=C\CCCCCCCCCC=C(Br)Br
429	500	CC1(C)C=CC(=O)C(=C1)[C@H](Cl)CBr
430	455	CCC[C@@H](O)C1=C(Br)\C(OC1=O)=C\Cl
431	1448	OC1=CC(C=O)=CC(Br)=C1O
432	311	CC(C)(Cl)[C@H](Br)CC[C@@](Cl)(CBr)C=C
433	777	C[C@H](O)[C@H](NC(=O)[C@H]1CCCN1)[C@H]1O[C@@H](SCCOC(=O)C2=C(O)C=CC=C2)[C@@H](O)[C@@H](O)[C@@H]1O
434	414	CO\N=C(\C(=O)N[C@H]1[C@@H]2SCC=C(N2C1=O)C([O-])=O)C1=CSC(N)=N1
435	1582	N[C@H]1C[C@@H](N)[C@@H](O[C@@H]2O[C@H](CO)[C@H](O)[C@H](N)[C@H]2O)[C@@H](O)[C@@H]1O
436	516	C[C@]12C[C@@H](O)CC(C)(C)C1=CC(=O)O2
437	412	C1C[C@H]2CCCCCC[C@@H]3CCN4CCC[C@H](CCCCCC[C@@H]5CCN(C1)[C@H]2O5)[C@@H]4O3
438	525	CCCCCCC1=C(O)C=C(O)C=C1
439	261	CCCCCCC(=O)C(Br)=C(Br)Br
440	1176	COC(=O)CC1=CC(Br)=C(OC)C(O)=C1
441	720	COC1=CC2=C(C(=O)O[C@H](C)C2)C(O)=C1
442	921	C[C@@H]1CC2=C(C(O)=CC=C2C)C(=O)O1
443	980	C[C@H](O)[C@H]1[C@@H]2C[C@H](SCCNC(=O)CCNC(=O)[C@@H](O)C(C)(C)CO)[C@@H](N2C1=O)C([O-])=O
444	145	CC1=CC2=C(Cl)C(O)=C(Cl)C(O)=C2C(=O)O1
445	576	COC1=C(C\C=C(/C)CC2=C[C@@]3(C)CCC[C@@]3(C)[C@]3(C[C@H](O)C(C)(C)O3)O2)C=C(O)C=C1C
446	683	CC(C)C[C@H](NC(=O)[C@@H](O)[C@@H](O)[C@@H]1CC(=O)O[C@@](C)(O)[C@H](C)N1)[C@@H]1CC2=C(C(=O)O1)C(O)=CC=C2
447	871	C[C@@]12C[C@@H](O)CC(C)(C)C1=CC(=O)O2
448	71	CC(=O)OC[C@H](Br)[C@@]1(C)CC=C2[C@@H](C1)[C@H](O)C[C@@H]1[C@@]3(COC(C)=O)C[C@H]3[C@@H](C[C@@]21C)OC(C)=O
449	1657	CN([C@@H]1[C@@H](O)[C@@H](OC(N)=O)[C@H](CO)O[C@@H]1NC1=N[C@@H]2[C@@H](N1)C(=O)N(C)C[C@@H]2O)C(=O)C\N=C\N
450	1513	[O-]C(=O)C\C=C1/CCC(=C1)[N+]#C
451	605	[O-]C(=O)C1=C(CSC2=NN=CS2)CS[C@H]2[C@H](NC(=O)CN3C=NN=N3)C(=O)N12
452	1362	CC(=C)[C@H](O)COC1=CC=C(CC([O-])=O)C=C1
453	884	CC1(C)C=CC(=O)C(\C=C/Br)=C1
454	137	C[C@]1(O)CC2=C(C(O)=CC=C2)C(=O)O1
455	616	COC1=CC(=O)N(C1)C(=O)[C@@H]1CSC(=N1)[C@@H](CO)NC(=O)C1=CSC(\C=N/O)=N1
456	337	CC[C@H](O)CCCCC1=C(C)C(=O)OC1
457	1109	OC1=C(Br)C=C(C=C1)C([O-])=O
458	544	C[C@H](O)CCCCCC1=C(C)C(=O)OC1
459	1365	CC(C)=CCOC1=CC=C(CCO)C=C1
460	535	C[C@@]12O[C@@H]1C=C(Br)C(C)(C)[C@@]21C[C@H](Br)[C@@](C)(Cl)C[C@@H]1O
461	380	CC1=CC(=O)C[C@]2(O)C3=C(C=C[C@@]12O)C(=O)C1=C(O)C=CC=C1C3=O

462	1302	CC1(C)C=CC(=O)C(\C=C/Cl)=C1
463	3	OC1=C(O)C(Br)=C(C=O)C=C1Br
464	745	C[C@@]12O[C@@H]1[C@H]1O[C@@]1(Br)C(C)(C)[C@@]21C[C@H](Br)[C@@](C)(Cl)C[C@@H]1O
465	940	COC1=C(C)C(O)=C2CO[C@H](O)C2=C1C([O-])=O
466	1378	CCCCCCCCCCCC\C=C(/Cl)C=O
467	295	CC(C)(Cl)[C@@H](Cl)C[C@H](Cl)[C@](C)(Cl)C=C
468	514	CN1N=NN=C1SCC1=C(N2[C@@H](SC1)[C@H](NC(=O)CSC(F)(F)F)C2=O)C([O-])=O
469	540	OCC[C@H]1CN2[C@H](CC2=O)O1
470	341	CC1=C(O)C=C2C(=O)OCC2=C1O
471	18	OC1=C(O)C=C(C=O)C=C1
472	1030	COC1=CC2=C(C=C1O)C1=CC3=C(C=C)C=NC=C3C(=O)N1CC2
473	1425	CSC1=C(C2=C(NC3=CC(Br)=CC(C)=C23)S(C)=O)C2=C(Br)C=C(Br)C=C2N1
474	1306	OC1=C(C(=O)\C=C\C=C)C(=O)N[C@H]1CCCNC(=O)\C=C\C=C
475	1547	CO[C@@H]1O[C@H]2CO[C@@H](O[C@@H]2[C@@H](NC(C)=O)[C@@H]1O)C1=CC=CC=C1
476	1495	[O-]C(=O)[C@H]1CS[C@@H]2[C@H](NC(=O)COC3=CC=CC=C3)C(=O)N12
477	1254	CC(C)[C@@H](C)CC[C@@H](C)[C@H]1CC[C@H]2[C@@H]3C[C@H](OS([O-])(=O)=O)[C@H]4C[C@H](OS([O-])(=O)=O)[C@H](C[C@]4(C)[C@H]3CC[C@]12C)OS([O-])(=O)=O
478	888	C[C@@](Cl)(C=C)[C@H](Cl)\C=C\C(=C)C(Cl)Cl
479	928	O[C@@H]1CC(=O)C2=C(C=C(O)C=C2O)[C@H]1O
480	1520	COC1=CC2=C(C(=O)O[C@H](C)[C@@H]2O)C(O)=C1OC
481	1339	O\N=C1\CC2=CC=C(OC3=CC(C\C(=N\O)C(=O)N\C=C/C4=CC=C(OC5=CC(CCNC1=O)=CC(Br)=C5O)C(Br)=C4)=CC(Br)=C3O)C(Br)=C2
482	526	O=C1N[C@H]2CC(=O)NCCCC[C@H]1NC2=O
483	642	CC(C)(O)[C@H]1CC[C@@](C)(O1)[C@@H](O)CC[C@](C)(O)[C@H]1CC[C@@]2(C)O[C@](C)(CC[C@]2(C)O1)[C@]1(C)CC[C@@H](Br)C(C)(C)O1
484	1402	CC(C)(O)[C@H]1CC[C@@](C)(O1)[C@@H]1CC[C@](C)(O1)[C@H]1CC[C@H](O1)[C@@]1(C)CC[C@@H]2OC(C)(C)[C@H](Br)CC[C@@]2(C)O1
485	367	C[C@@]12O[C@@H]1[C@H](O)[C@]1(Br)O[C@H]3C[C@](C)(Cl)[C@@H](Br)C[C@]23C1(C)C
486	1205	CC(C)(O)[C@@H]1CC[C@@](C)(O1)[C@@H]1CC[C@@](C)(O1)[C@@H]1CC[C@H](O1)[C@@]1(C)CC[C@@H]2OC(C)(C)[C@H](Br)CC[C@@]2(C)O1
487	947	O[C@H]1CC[C@@H](O)C1(Cl)Cl
488	409	O[C@@H](C(=O)N[C@H]1[C@@H]2SCC(CSC3=NN=NN3CS([O-])(=O)=O)=C(N2C1=O)C([O-])=O)C1=CC=CC=C1
489	728	CC1=C(O)C(C=O)=C(C=O)C=C1O
490	1646	C[C@H]1[C@H]2O[C@H]2[C@]2(C)N1C(NC(=O)C=C(C)C)=CC2=O
491	1408	COC1=CC(O)=C2C(=O)C3=C([C@@H](O)[C@](C)(O)[C@H](O)C3)C(=O)C2=C1
492	205	CO[C@@H]1CC(=N[C@]11O[C@@H](C)C[C@@H]2CCCCCCCC3=CC=C(N3)[C@@H]12)C1=CC=CN1
493	494	CC(C)=CCC[C@@](Cl)(CBr)C(Cl)=C
494	1191	COC1=C(O)C=CC(C=O)=C1
495	1609	NC1=N[C@@H](O)[C@@]2(N1)[C@@H](Cl)[C@H](CNC(=O)C1=CC(Br)=C(Br)N1)[C@H]1CN3C(=O)C4=CC(Br)=C(Br)N4[C@@H]4N=C(N)N[C@]34[C@@H]21
496	120	CN(CC[C@@H](N)CC(=O)N[C@@H]1C=C[C@@H](O[C@H]1C([O-])=O)N1C=CC(N)=NC1=O)C(N)=N

497	1713	CO[C@H](C)[C@H]1C[C@H](C)[C@@H](O1)[C@@H](C)\C=C\[C@@H]1CC[C@H](C)[C@H](O1)C(\CO)=C/[C@H]1CCC[C@@H](C)[C@H]1[C@H](C)C(\O)=C1\C(=O)COC1=O
498	1607	NC1=N[C@H](O)[C@]2(N1)[C@H](Cl)[C@@H](CNC(=O)C1=CC(Br)=C(Br)N1)[C@@H]1CN3C(=O)C4=C([C@H]5N=C(N)N[C@@]35[C@@H]21)C(Br)=C(Br)N4
499	1178	N[C@@H]1C[C@@H](N)[C@@H](O)C(O[C@@H]2O[C@H](CO)[C@@H](O)[C@H](O)[C@H]2O)[C@H]1O
500	1301	OC[C@]1(O)[C@@H](O)C=CC1=O
501	715	OC[C@@H]1NC[C@@H](O)[C@H]1O
502	860	CC(C)(O)[C@@H]1CC[C@]2(C)O[C@H]2CC[C@]2(C)[C@@H](Br)CCC(=C)[C@H]2CC2=CC(=CC=C2O)C(=O)O1
503	134	OC1=C(C=C(C=O)C=C1)N=O
504	1463	CC[C@@H]1O[C@]2(C[C@@H]3CC[C@@H]4[C@H](C(=O)OCCCCCCCCCCCCCCCCC(=O)N(CCCN)C[C@@H](O)CCN)[C@]5(CCC[C@@H](C)O5)NC(=N2)N34)CCC=C1
505	466	CO[C@H](CNC(=O)C(\C)=C\C)C1=CC(=O)C2=C3C(=CC=N2)C2=CC=CC=C2N=C13
506	12	CO\N=C(\C(=O)N[C@H]1[C@@H]2SCC(COC(C)=O)=C(N2C1=O)C([O-])=O)C1=CSC(N)=N1
507	2	CO\N=C(\C(=O)N[C@H]1[C@@H]2SCC(COC(N)=O)=C(N2C1=O)C([O-])=O)C1=COC=C1
508	805	CC1=C(Br)C=C(C(O)=C1C1=C(O)C(=CC(Br)=C1C)[C@]1(C)CC[C@@H]2C[C@]12C)[C@@]1(C)CC[C@H]2C[C@@]12C
509	788	CO[C@H]1O[C@H]2CO[C@H](O[C@H]2[C@@H](NC(C)=O)[C@@H]1OC(C)=O)C1=CC=CC=C1
510	840	CO[C@H](\C=C\C(C)(C)O)C(\C)=C\C=C\C(\C)=C1/C(=O)C[C@@H]2[C@]1(C)CC[C@@H]1[C@]2(C)CC[C@@H](OC(C)=O)[C@]1(C)C([O-])=O
511	1454	CCCCCCCCC\C=C\C(=O)OC\C(=C/C=C/C(/C)=C/C=C/C=C(\C)/C=C/C=C(\C)/C=C/[C@H]1C(C)=C[C@H](O)CC1(C)C)C(=O)CC1=C(C)C[C@@H](O)CC1(C)C
512	1050	O[C@]1(CC(NC=O)=CC1=O)C=C
513	859	CC(C)=CC(=O)NC[C@H](O)C1=CC(=O)C2=C3C(=CC=N2)C2=CC=CC=C2N=C13
514	1486	CO\N=C(/C(=O)N[C@H]1[C@H]2SCC(C[N+]3(C)CCCC3)=C(N2C1=O)C([O-])=O)C1=CSC(N)=N1
515	753	C[C@H]1[C@@H](Br)[C@H](OC(C)=O)C(C)=C2CC[C@](C)(Cl)[C@@H](Br)C[C@]12C
516	522	COC1=CC(=O)C=CC1=[N+]=[N-]
517	646	CC(C)(O)[C@H]1CC[C@@](C)(O1)[C@@H](O)CCC(=C)[C@H]1CC[C@H]2O[C@H](CC[C@]2(C)O1)[C@]1(C)CC[C@H](O1)C(C)(C)O
518	244	C[N+]1=C(C=CC=C1)C([O-])=O
519	925	C[C@H]1[C@@H](O)OC[C@H]1C(C)=O
520	732	C[C@@H](CCC=C(C)C)[C@H]1CCC(=C)C2CC=C(C=O)[C@@H]1C2OC(C)=O
521	339	NC(=O)C1=C(O)N(C=N1)[C@@H]1O[C@@H](CO)[C@H](O)[C@H]1O
522	1403	C[C@H]1C[C@H]2C[C@@H](C)[C@@](C)([N+]#C)[C@H]3CC[C@H]4[C@@H]([C@@H]1CC[C@]4(C)NC=O)[C@H]23
523	1113	CCC[C@H]1CC2=C(C[C@@H](CC)CC3=C1C(=O)OC3=O)C(=O)OC2=O
524	127	C[C@H](O)C1=CC(=O)[C@@H](O)[C@@H]1O
525	1065	CC(=O)OC(C)(C)[C@H]1CC[C@@](C)(O1)[C@@H](O)CC[C@](C)(O)[C@H]1CC[C@H]2O[C@H](CC[C@]2(C)O1)[C@]1(C)CC[C@@H](Br)C(C)(C)O1
526	1033	O\N=C1/CC2=CC(Br)=C(OC3=CC(C\C(=N/O)C(=O)NCCC4=CC(Br)=C(O)C(OC5=CC=C(CCNC1=O)C=C5Br)=C4)=CC(Br)=C3O)C(Br)=C2
527	1155	CC[C@@H](Br)[C@H]1C[C@@H](Br)[C@H]2C[C@H](O2)[C@H](C\C=C/C#C)O1

528	472	CC(=O)O[C@H](CC[C@](C)(O)[C@H]1CC[C@@H]2O[C@@H](CC[C@@]2(C)O1)[C@@]1(C)CC[C@@H](Br)C(C)(C)O1)[C@@]1(C)CC[C@@H](O1)C(C)(C)O
529	283	CC(C)(Br)[C@@H]1CC[C@]2(C)O[C@H]2CC[C@]2(C)[C@@H](Br)CCC(=C)[C@H]2CC2=CC(=CC=C2O)C(=O)O1
530	1375	CC(C)(O)[C@H]1CC[C@](C)(O1)[C@H](O)CC[C@](C)(O)[C@H]1CC[C@H]2O[C@H](CC[C@]2(C)O1)[C@]1(C)CC[C@@H](Br)C(C)(C)O1
531	166	OCC1(O)C(=O)C=CC1=O
532	1206	C\C(\C=C\C=C(/C)\C=C\C1=C(C)CCCC1(C)C)=C/C=C/C=C(\C)/C=C/C=C(\C)/C=C/C1=C(C)C(=O)CCC1(C)C
533	358	O\N=C(\CC1=CC(Br)=C(OC2=CC(C\C(=N\O)C(=O)NCCC3=CC(Br)=C(O)C=C3)=CC(Br)=C2O)C=C1)C(=O)NCCC1=CC(Br)=C(O)C=C1
534	1545	CC(=O)O[C@@H]1C[C@]2(C)O[C@@H]3C[C@H](Br)C(C)(C)O[C@]3(C)[C@H]1C[C@H]2O
535	327	CC(C)(O)[C@H]1CC[C@@](C)(O1)[C@H](O)CC[C@](C)(O)[C@H]1CC[C@@H]2O[C@@H](CC[C@@]2(C)O1)[C@@]1(C)CC[C@@H](Br)C(C)(C)O1
536	546	CC[C@@H](Br)[C@H]1C[C@@H](Br)[C@H]2C[C@@H](O1)[C@H](C\C=C\C#C)O2
537	181	CO\N=C(/C(=O)N[C@H]1[C@@H]2SCC(CSC3=CN=NS3)=C(N2C1=O)C([O-])=O)C1=CSC(N)=N1
538	1571	CC(=C)[C@@H]1CC[C@@]2(C)O[C@H]2CC[C@]2(C)[C@@H](Br)CCC(=C)[C@H]2CC2=CC(=CC=C2O)C(=O)O1
539	1625	CO[C@H]1CC[C@H](O[C@@H]2[C@@H](C)[C@]3(O[C@]2(C)[C@H]2CC[C@@H](O2)[C@H]2CC[C@@H](O2)[C@H]2O[C@](C)(O)[C@@H](C)[C@@H](O[C@@H]4CC[C@H](OC)[C@H](C)O4)[C@H]2C)O[C@H](C[C@H]2O[C@](O)([C@@H](O)C([O-])=O)[C@@H](C)[C@H](OC)[C@@]2(C)OC)C[C@@H](OC)[C@@H]3C)O[C@@H]1C
540	22	CN[C@H]1[C@H](C[C@@H](N)[C@H](O[C@@H]2OC(=CC[C@H]2N)[C@@H](C)N)[C@H]1O)OC
541	1368	C\C(=C/C[C@H](O)[C@@]1(C)CC[C@@H](O1)C(C)(C)O)[C@H]1CC[C@H]2O[C@H](CC[C@]2(C)O1)[C@]1(C)CC[C@@H](Br)C(C)(C)O1
542	1596	O\N=C1\CC2=CC(Br)=C(OC3=CC(C\C(=N\O)C(=O)NCCC4=CC(Br)=C(OC5=CC(CCNC1=O)=CC(Br)=C5O)C(Br)=C4)=CC(Br)=C3O)C(Br)=C2
543	1648	CCCC[C@@H]1CCCC[C@H](C)[C@@H](OC(N)=O)C2=CC(O)=C([C@@H](CCCCCl)CCCC[C@H](C)[C@@H](OC(N)=O)C3=CC(O)=C1C(O)=C3)C(O)=C2
544	520	CC(=O)OC(C)(C)[C@H]1CC[C@@](C)(O1)[C@H](O)CC[C@](C)(O)[C@H]1CC[C@@H]2O[C@@H](CC[C@@]2(C)O1)[C@@]1(C)CC[C@H](Br)C(C)(C)O1
545	581	C[C@H](O)[C@@H](NC(=O)[C@H](C)N)C([O-])=O
546	1487	C\C=C(/CC[C@@H](C)[C@@H]1CC[C@H]2[C@H]3CC(=O)C4=CC(=O)CC[C@]4(C)[C@H]3CC[C@]12C)C(C)C
547	1652	CN\C(NCCC\C=C\CCC[C@H](C)[C@H]1OC(=O)\C(C)=C/C=C\[C@H](C)[C@H](O)C[C@@H](O)[C@@H](C)[C@H](O)CC[C@@H](C)[C@H](O)C[C@]2(O)O[C@H](C[C@H](O)[C@H]2O)C[C@H](O)C[C@@H](C[C@H](O)C[C@H](O)\C(C)=C/C=C\[C@H]1C)OC(=O)CC([O-])=O)=N/C
548	1499	C[C@@H]1OC(=O)C(C(=O)CCC([O-])=O)=C1O
549	1231	O\N=C1\CC2=CC(Br)=C(OC3=CC(C\C(=N/O)C(=O)NCCC4=CC(Br)=C(O)C(OC5=CC=C(CCNC1=O)C=C5Br)=C4)=CC(Br)=C3O)C(Br)=C2
550	287	CC1=C2CC3=CC(=CC=C3O)C(=O)O[C@@H](CC[C@](C)(O)[C@H](Br)CC[C@]2(C)[C@@H](Br)CC1)C(C)(C)O

551	90	CO[C@H]1CC\C=C(\C)/C=C/C[C@H](OC)\C=C(/C)\C=C\[C@@H](C)\C=C(C)\C(=O)O[C@@H]([C@@H](C)\C=C\C=C\1)C(\C)=C\C=C(/C)CNC(=O)[C@H](CO)NC=O
552	1692	CO[C@H]1\C=C/C=C(C)/C[C@@H](C)[C@H](O)[C@H](C)\C=C(/C)\C=C(OC)/C(=O)O[C@@H]1[C@H](C)[C@H](O)[C@H](C)[C@]1(C[C@@H](OC(=O)\C=C\C(=O)NC2=C(O)CCC2=O)[C@H](C)[C@@H](O1)C(C)C)OC
553	225	CC1(C)[C@H](Br)C[C@H](O)[C@]1(C)[C@@]1(O)CC[C@]2(C)[C@@H](Br)CC[C@@](C)(O)[C@@]2(O)C1
554	899	COC(=O)CCC(=O)NCCCCCN(O)C(=O)CC(C)C
555	1683	CO[C@@H]1[C@@H](C)[C@]2(O[C@]1(C)[C@H]1CC[C@@H](O1)[C@H]1CC[C@@H](O1)[C@H]1O[C@@](C)(O)[C@H](C)[C@@H](O[C@@H]3CC[C@@H](OC)[C@@H](C)O3)[C@@H]1C)O[C@H](C[C@H]1O[C@](O)([C@@H](O)C([O-])=O)[C@@H](C)[C@H](OC)[C@@]1(C)OC)C[C@@H](OC)[C@@H]2C
556	444	C[C@]12C[C@H]1CC[C@]1(C)[C@@H]2CC[C@@H]2C[C@](C)(CC=C12)[C@@H](Br)CO
557	530	CCO[C@H]1OC(=O)C2=C(OC)C(C)=C(OCC=C(C)C)C(C=O)=C12
558	474	CC\C=C/C[C@H](O)[C@H]1C=C[C@H](C\C=C/C\C=C/CCCC(=O)C2=C(O)C=C(O)C=C2O)N2N1C(=O)N(C2=O)C1=CC=CC=C1
559	597	CC[C@@H](Br)[C@H]1C[C@H](Br)[C@@H]2C[C@H](O1)[C@@H](C\C=C\C#C)O2
560	1507	CC[C@@H]1O[C@@H]2C[C@H](Br)[C@H](C\C=C/C#C)O[C@@H]2C[C@@H]1Br
561	1561	C[C@@H]1CCC\C=C/[C@@H]2O[C@@H](C[C@@H](O)[C@H]2O)C\C=C/C=C\[C@H](O)C\C=C/C=C\C(=O)O1
562	1572	CO[C@@H]1[C@@H](C)[C@]2(O[C@]1(C)[C@H]1CC[C@H](O1)[C@H]1CC[C@@H](O1)[C@H]1O[C@](C)(O)[C@H](C)C[C@@H]1C)O[C@H](C[C@@H]1O[C@](O)(CC([O-])=O)[C@H](C)[C@H](OC)[C@@]1(C)O[C@@H]1CC[C@@H](OC)[C@H](C)O1)C[C@@H](OC)[C@@H]2C
563	815	CC1=C(Br)C=C(C(O)=C1C1=C(O)C(=CC(Br)=C1C)[C@@]1(C)CC[C@@H]2C[C@]12C)[C@@]1(C)CC[C@@H]2C[C@]12C
564	1714	CC[C@]1(CC[C@@H](O1)[C@@]1(C)CC[C@@]2(C[C@@H](O)[C@@H](C)[C@H](O2)[C@@H](C)[C@@H](OC)[C@H](C)C([O-])=O)O1)[C@@H]1O[C@H](C[C@@H]1C)[C@@H]1O[C@@](O)(COC(=O)NCCC2=CC=CC=C2)[C@H](C)C[C@H]1C
565	1247	CO[C@]1(C)[C@H]2C[C@H]2[C@@]2(C)CC[C@@H](Br)[C@@](C)(O)CC[C@H](OC(=O)C3=CC=C(O)C(C[C@H]12)=C3)C(C)(C)Br
566	968	CN1N=NN=C1SCC1=C(N2[C@@H](SC1)[C@H](NC1=CC=[N+](CC3=CC=CC=C3)C=C1)C2=O)C([O-])=O
567	648	O[C@H](\C=C\CCCCCCCCC\C=C/CCCC\C=C/CCCCCCC\C=C\[C@@H](O)C#C)C#C
568	1696	C[C@@H]([C@@H](O)C(\C)=C/C=C/C(/C)=C/C1=COC(C)=N1)[C@@H]1C[C@H](O)\C(C)=C\C=C/[C@H](C)[C@H]2C[C@H](CC(=O)O2)C\C=C\C(=O)O1
569	637	CC1=C2CC3=CC(=CC=C3O)C(=O)O[C@H]3CC[C@@](C)(OC3(C)C)[C@H](Br)CC[C@]2(C)[C@@H](Br)CC1
570	42	[O-]C(=O)C1=CC=CC=C1
571	863	CC(=C)[C@@H]1CC[C@](C)(O)[C@H](Br)CC[C@]2(C)[C@@H](Br)CCC(=C)[C@H]2CC2=CC(=CC=C2O)C(=O)O1
572	689	CC(=O)OC[C@]1(C)CCC[C@@]2(C)[C@H]1CC[C@]1(C)[C@@H]2CC[C@]2(C)[C@@H]3[C@H](O)OC(=O)C3=CC[C@@H]12
573	1509	CC1(C)[C@H]2CC[C@](C)(C=C)[C@@]3([N+]#C)[C@H]4O[C@H]4C(C)(C)C4=NC5=CC=CC1=C5[C@]4(O)[C@]23O

574	1012	CC(C)=CCC[C@](C)(O)[C@H]1CC[C@](C)(O1)[C@H]1CC[C@H](O1)[C@@]1(C)CC[C@H](O1)[C@@]1(C)CC[C@H](O1)C(C)(C)O
575	271	C[C@]12C[C@H]1[C@H](O)C[C@]1(C)[C@@H]2C[C@@H](O)[C@@H]2C[C@](C)(CC=C12)[C@@H](Br)CO
576	1359	CO[C@@H]1C[C@@]2(OC1(C)C)OC(C\C(C)=C\CC1=CC(=O)C=C(C)C1=O)=C[C@]1(C)CCC[C@]21C
577	1063	C\C(\C=C\C=C(/C)\C=C\[C@H]1C(C)=C[C@H](O)CC1(C)C)=C/C=C/C=C(\C)/C=C/C=C(\CO)C(=O)CC1=C(C)C[C@@H](O)CC1(C)C
578	1165	C[C@H](OS([O-])(=O)=O)[C@H]1[C@@H]2CC(=C(N2C1=O)C([O-])=O)[S@@](=O)CCNC(C)=O
579	938	CC1(C)[C@@H](Br)CC[C@@]2(C)[C@H]1CC[C@@]1(C)O[C@H](CCl)[C@](C)(O)CC[C@H]21
580	918	COC1=C2C[C@@H]3[C@](C)(CC[C@H]4[C@]3(C)CC[C@H]3C(C)(C)[C@@H](O)CC[C@@]43C)C2=C(O)C=C1C
581	1578	CC[C@@H](C)[C@H]1[C@@](C)(O)C(=O)[C@@H]2C[C@](C)(O)C[C@@H](C)[C@H]2[C@@]1(C)C(C)=O
582	1325	CC[C@@H]1O[C@@H]2C[C@H](Cl)[C@H](C\C=C/C#C)O[C@@H]2C[C@@H]1Br
583	1642	CC(C)[C@@H](C)\C=C\[C@@H](C)[C@H]1CC[C@@H]2C3=CC=C4C[C@@H](O)CC[C@]4(C)[C@@H]3CC[C@]12C
584	57	C[C@]12C[C@H]1CC[C@]1(C)[C@@H]2C[C@@H](O)[C@@H]2C[C@](C)(CC=C12)[C@@H](Br)CO
585	1435	CCCCCCCCCCCCCCCCCCCCCCC[C@@H](O)C(=O)N[C@H](CO)[C@H](O)[C@H](O)C\C=C\CCCC[C@@H]1C[C@H]1CCCC
586	477	CC1=C2CC3=CC(=CC=C3O)C(=O)O[C@@H](CC[C@](C)(O)[C@H](Br)CC[C@]2(C)[C@@H](Br)CC1)C(C)(C)Br
587	252	CC1=CC[C@H]2[C@@](C)(CC[C@H]3C(C)(C)CCC[C@]23C)[C@@H]1CC1=C(O)C=CC(=C1)C([O-])=O
588	60	CC1=C(O)C=C(C(Br)=C1)[C@]1(C)CC[C@H]2C[C@@]12C
589	229	CC(C)CCC[C@@H](C)[C@H]1CC[C@H]2[C@@H]3[C@H](O)C=C4C[C@@H](O)CC[C@]4(C)[C@H]3CC[C@]12C
590	892	CC\C=C/C\C=C/C\C=C/C\C=C/C\C=C/CCCC(=O)C1=C(O)C=C(O)C=C1O
591	635	CC(C)(O)[C@@H]1O[C@](C)(C[C@@H]1O)[C@@H](O)CC[C@](C)(O)[C@H]1CC[C@H]2O[C@H](CC[C@]2(C)O1)[C@]1(C)CC[C@@H](Br)C(C)(C)O1
592	83	CC[C@@H]1O[C@@H]2C[C@@H](O[C@@H]2C\C=C\C#C)[C@@H](O)C[C@H]1Br
593	1070	CC1=CC[C@H](Br)[C@@]2(C)CC[C@@H](Br)[C@@]3(C)CC[C@H](OC(=O)C4=CC=C(O)C(C[C@H]12)=C4)C(C)(C)O3
594	1076	CC(C)(Br)[C@@H]1CC[C@](C)(O)[C@H](Br)CC[C@]2(C)[C@@H](Br)CCC(=C)[C@H]2CC2=CC(=CC=C2O)C(=O)O1
595	1635	COC(=O)[C@H]1C2=CC3=C(C(O)=C2[C@H](C[C@]1(C)O)O[C@@H]1C[C@H]([C@H](O[C@H]2C[C@@H](O)[C@H](O[C@@H]4CC[C@H](O)[C@@H](C)O4)[C@@H](C)O2)[C@H](C)O1)N(C)C)C(=O)C1=C(O)C=CC=C1C3=O
596	1366	C[C@H](CCC=C(C)C)[C@@H]1[C@H](O)C[C@@H](C)[C@@H]2C[C@@H]2C2=C1C(=O)O[C@H]2[C@H](O)[C@H]1[C@H]([C@H](C)CCC=C(C)C)[C@H](O)C\C(C)=C/C\C=C1\C=O
597	1598	CC[C@H]1OC(=O)[C@@H](C[C@H]2C=C[C@@H]3[C@@H]4O[C@]2([C@H]3[C@@H](O)[C@H](C)[C@H]4OC(=O)C2=CC=CN2)C(C)=C[C@@H]1C)OC
598	537	C[C@H]1OC(=O)C=C1O
599	1675	CC[C@H](C(=O)NC\C=C/C=C(\C)[C@@H](OC)[C@H](C)[C@H]1C[C@H](O)[C@H](O1)\C=C\C=C\C=C\C([O-])=O)[C@]1(O)C[C@@H](O)[C@@](C)(O)[C@@H](O1)\C=C\C=C/C
600	1570	CC[C@@H]1O[C@@H]2C[C@@H](O[C@@H]2C\C=C\C#C)[C@@H](O)C[C@H]1O

601	66	CC(C)(Cl)[C@@H](Br)CC[C@@]1(C)[C@H](Br)CC[C@@]2(C)[C@@H](CC3=C(O)C(Br)=CC(Br)=C3)C(=C)CC[C@H]12
602	1466	C\C(CCC1=C(C)CCCC1(C)C)=C/CC\C(CO)=C/C=C/C1=CC(=O)O[C@@H]1O
603	1533	O\N=C(\CC1=CC(Br)=C(O)C(=C1)C1=C(O)C(Br)=CC(C\C(=N\O)C(=O)NCCC2=CC(Br)=C(O)C=C2)=C1)C(=O)NCCC1=CC(Br)=C(O)C=C1
604	27	CCC[C@@H]([C@@H](OC)[C@H](C)[C@H]1O[C@]2(CC[C@@](C)(O2)[C@@H]2O[C@](CCC)(C[C@@H]2O[C@H]2CC[C@H](OC)[C@H](C)O2)[C@H]2O[C@H](C[C@@H]2C)[C@@H]2O[C@@](O)(CO)[C@@H](C)C[C@H]2C)C[C@@H](OC)[C@@H]1C)C([O-])=O
605	195	CC[C@@H](Br)[C@@H]1C\C=C/C[C@H](Cl)[C@H](C\C=C\C#C)O1
606	1219	CO[C@H](CCCCCCC[C@@H]1NCCC2=C1C=C(O)C(O)=C2)\C=C\CCCCCCCCCCC[C@@H](O[C@@H]1O[C@H](CO)[C@H](O)[C@H](O)[C@H]1O)[C@@H](C)N
607	94	CC1(C)[C@@H](O)CC[C@@]2(C)[C@H]1CC[C@@]1(C)[C@@H]3CCC4=C(C(=O)OC4)[C@@]3(C)[C@H](O)C[C@H]21
608	1667	CC[C@@H](O)[C@@]1(C)C[C@@H](C)[C@H](O1)[C@H]1C[C@@H](C)[C@@H](O1)[C@@]1(C)O[C@H](C[C@H]1OC)[C@]1(C)CC[C@]2(C[C@H](O)[C@@H](C)[C@H](O2)[C@@H](C)[C@@H](OC(=O)C2=C(O)C=C(OC)C=C2C)[C@@](C)(O)C([O-])=O)O1
609	63	C[C@H](CC[C@@H](OO)C(C)=C)[C@@H]1CC[C@@H]2[C@H]3C[C@H](O)C4=CC(=O)CC[C@@]4(C)[C@@H]3CC[C@]12C
610	163	CCCCCC[C@@H](C)[C@H]1CC(=O)N[C@@H](CC2=CC=CC=C2)C(=O)N[C@H](CC2=CC=CC=C2)C(=O)N[C@H]([C@@H](C)CC)C(=O)O1
611	459	C[C@H](CCC=C(C)C)[C@@H]1CC[C@@H]2[C@H]3CC=C4C[C@H](O)CC[C@@]4(C)[C@@H]3CC[C@]12C
612	1615	CO[C@@H]1C[C@@H](C[C@@H](O)CC\C(C)=C\[C@H](C)C([O-])=O)O[C@@]2(O[C@@](C)(C[C@H]2C)[C@H]2CC[C@@](C)(O2)[C@@H]2O[C@H](C[C@@H]2C)[C@@H]2O[C@](O)(CO)[C@@H](C)C[C@@H]2C)[C@@H]1C
613	1420	CCCCCCCCCCCCCCCCCCCCCC[C@@H](O)[C@@H](O)[C@@H](CO)NC(=O)[C@H](O)CCCCCCCC[C@H]1C[C@@H]1CCCC
614	1693	CC[C@@H](C(=O)NC\C=C\C=C(/C)[C@H](OC)[C@H](C)[C@H](O)[C@@H](O)\C=C/C=C/C=C/C=C(\C)C(=O)C1=C(O)C=CN(C)C1=O)[C@]1(O)C[C@H](O)C(C)(C)[C@H](O1)\C=C\C=C\C
615	1419	O[C@H](\C=C\CCCCCCCCC\C=C/CCCC\C=C/CCCCCCCCC\C=C\[C@@H](O)C#C)C#C
616	335	CC1(C)[C@@H]2CC[C@@](C)(O2)[C@@]11CC[C@@](C)(Cl)[C@H](Br)C1
617	770	C[C@]12C[C@H]1CC[C@@]1(C)[C@H]3CC[C@@](C)(CC3=CC[C@H]21)[C@@H](Br)CO
618	156	CC1=C2CC[C@](C)(O)[C@@H](O)C[C@H]2[C@@](C)(C1)[C@@H]1CC[C@](C)(O)[C@H](Br)C1
619	136	CC1(C)[C@@H]2CC[C@](C)(O)[C@@H]2[C@@H](Br)[C@@H]1C(=C)[C@@H]1CC[C@](C)(O)[C@H](Br)C1
620	607	CC1=CC(O)=C(C=C1Br)[C@]1(C)CC[C@@H]2C[C@]12C
621	966	CC(C)[C@H]1NC(=O)[C@@H](NC(=O)C2=C3N=C4C(OC3=CC=C2)=CC(=O)C(N)=C4C(=O)N[C@H]2[C@@H](C)OC(=O)[C@H](C(C)C)N(C)C(=O)CN(C)C(=O)[C@@H]3CCCN3C(=O)[C@@H](NC2=O)C(C)C)[C@@H](C)OC(=O)[C@H](C(C)C)N(C)C(=O)CN(C)C(=O)[C@@H]2CCCN2C1=O
622	1117	CCCCCC[C@H]1C(=O)N[C@@]([C@@H](O)[C@H]2CCCC=C2)(C([O-])=O)[C@]1(C)O
623	932	COC1=C2C[C@@H]3[C@](C)(CC[C@H]4[C@]3(C)CC[C@H]3C(C)(C)[C@H](CC[C@@]43C)OC(C)=C)C2=C(OC(C)=O)C=C1C

624	1661	CO[C@@H]1[C@@H](O)[C@@H](C)O[C@@H](OC[C@@H](C)[C@H]2OC(=O)[C@H](C)[C@@H](O[C@H]3C[C@@](C)(O)[C@H](O)[C@@H](C)O3)[C@H](C)[C@@H](O)[C@H](C)C[C@@](C)(O)C(=O)[C@@H](C)[C@H](OC(=O)[C@@H](O)C(C)C)[C@H]2C)[C@H]1OC
625	1480	CC(C)=CCC\C(=C\CC\C(C)=C\CC\C(C)=C\CC1=C(O)C(C)=CC(O)=C1)C([O-])=O
626	1634	CO[C@H]1\C=C/C=C(C)/C[C@@H](C)[C@H](O)[C@H](C)\C=C(/C)\C=C(OC)/C(=O)O[C@@H]1[C@H](C)[C@H](O)[C@H](C)[C@@]1(C[C@@H](O)[C@H](C)[C@H](O1)C(C)C)OC
627	1162	CC(C)[C@H]1[C@@H]2C[C@@]3(C)[C@H]([C@H]12)[C@](C)(O)CC[C@@H]3Br
628	1681	CO[C@H](C)[C@H](NC(=O)[C@H]1CCCN1C)[C@H]1O[C@@H](SCCOC(C)=O)[C@H](O)[C@@H](O)[C@@H]1O
629	1268	C[C@@]1(CO)CCC[C@@]2(C)[C@H]1CC[C@@]1(C)[C@@H]3CCC4=C(C(=O)OC4)[C@@]3(C)[C@H](O)C[C@H]21
630	1310	CC1(C)[C@@H]2CC[C@@](C)(O)[C@@H]2[C@@H](Br)[C@@H]1C(=C)[C@@H]1CC[C@](C)(O)[C@H](Br)C1
631	143	CCCCCC[C@@H](C)[C@H]1CC(=O)N[C@H](CC2=CNC3=CC=CC=C23)C(=O)N[C@H](CC2=CC=CC=C2)C(=O)N[C@H]([C@@H](C)CC)C(=O)O1
632	461	CC1=CC(O)=C(C=C1Br)[C@]1(C)CC[C@@](C)(O)C1=C
633	672	C[C@]1(O)CC[C@H](Br)C(C)(C)[C@@H]1CC[C@]1(C)[C@@H](Br)CC=C2COC3=CC=C(C=C3C[C@@H]12)C([O-])=O
634	1320	COC1=C(Br)C=C(Br)C(Br)=C1OC1=CC(Br)=CC(Br)=C1O
635	276	CC(C)(Br)[C@H](Cl)CC[C@@]1(C)[C@H](Br)CC[C@@]2(C)[C@@H](CC3=C(O)C(Br)=CC(Br)=C3)C(=C)CC[C@H]12
636	116	C\C=C(\CC[C@H](C)[C@H]1CC[C@H]2[C@H]3CC=C4C[C@@H](O)CC[C@]4(C)[C@H]3CC[C@]12C)C(C)C
637	825	C[C@H]1CCC\C=C/[C@H]2CC[C@H](O)[C@@H](CC(=O)O1)O2
638	748	CC[C@H](C)[C@H]1NC(=O)[C@H](NC(=O)C2=C3N=C4C(OC3=CC=C2)=C(C)C(=O)C(N)=C4C(=O)N[C@H]2[C@@H](C)OC(=O)[C@@H](C(C)C)N(C)C(=O)CN(C)C(=O)[C@H]3CCCN3C(=O)[C@@H](NC2=O)[C@H](C)CC)[C@@H](C)OC(=O)[C@H](C(C)C)N(C)C(=O)CN(C)C(=O)[C@H]2CCCN2C1=O
639	836	C[C@](O)(CCCCCCCC\C=C\C=C\C1=CC=CC=C1)C[C@@]1(C)OC(=O)C[C@H]1O
640	344	CC(C)=CC[C@@H](O)C(\CO)=C\CC\C(C)=C\CC\C(C)=C\CC1=C(O)C(C)=CC(O)=C1
641	1700	CO[C@@H]1CC(=O)NC2=CC(O)=CC(CC\C=C(C)\[C@H](O)[C@H](C)[C@@H](C\C=C\C=C\C=C\1)OC(=O)[C@@H](C)NC(=O)CCC(C)C)=C2O
642	148	CC1(C)[C@@H]2CC[C@](C)(C=C)[C@@]3([N+]#C)[C@H]4O[C@H]4C(C)(C)C4=C(C5=C1C=CC=C5N4)[C@]23O
643	874	C[C@]1(O)CC[C@H](Br)C(C)(C)[C@@H]1CC[C@]1(C)[C@@H](Br)CCC2=COC3=CC=C(C=C3C[C@@H]12)C([O-])=O
644	661	CC1=C2CC3=CC(=CC=C3O)C(=O)O[C@@H](CC[C@](C)(O)[C@H](Br)CC[C@H]2C(=C)CC1)C(C)(C)O
645	757	CC[C@@H](Br)[C@H]1C[C@@H](OC(C)=O)[C@@H](Cl)C[C@H](Cl)[C@H](C\C=C\C#C)O1
646	668	C[C@@H]1CC[C@H]2C(C)(C)[C@H](O)CC[C@]2(C)[C@H]1CCC(=C)C=C
647	912	CC[C@H]1O[C@@H](C[C@@H]1Br)[C@H]1C[C@H](Br)[C@@H](C\C=C/C#C)O1
648	1674	CC[C@@H]([C@H](O)[C@@H](C)C[C@@H](C)[C@H]1OC(=O)C[C@@]2(O)CC=C(C)[C@@H](O2)\C(C)=C\CCC[C@H](O[C@H]2C[C@@H](OC(N)=O)[C@H](O)[C@@H](C)O2)\C=C\[C@@H](C)[C@H](O)[C@H]1C)C(=O)CC

649	1537	CCCCCC[C@@H](C)[C@H]1CC(=O)N[C@@H](CC2=CC=CC=C2)C(=O)N[C@H](CC2=CC=CC=C2)C(=O)N[C@@H]([C@H](C)CC)C(=O)O1
650	98	C[C@]1(CO)CCC[C@@]2(C)[C@H]1CC[C@@]1(C)[C@@H]3CCC4=C(C(=O)OC4)[C@@]3(C)[C@H](O)C[C@H]21
651	779	CC(C)[C@@H]1C=CC[C@H]2[C@@H]3[C@](C)(O)CC[C@H](Br)[C@@]3(C)CC[C@]12CBr
652	102	C[C@]1(CCCCCCCC\C=C\C=C\C2=CC=CC=C2)C[C@]2(C)OC(=O)C[C@@H]2O1
653	5	C[C@@H]1CCC[C@@H]2O[C@H]2\C=C\[C@H](O)C[C@@H](O)C\C=C/C=C/[C@@H](O)C\C=C\C=C/C(=O)O1
654	1243	CC(=C)[C@@H]1CC[C@](C)(O)[C@H](Br)CC[C@]2(C)[C@@H](Br)CCC(C)=C2CC2=CC(=CC=C2O)C(=O)O1
655	480	O\N=C1/CC2=CC(Br)=C(OC3=CC(C\C(=N/O)C(=O)NCCC4=CC=C(Br)C(OC5=CC=C(CCNC1=O)C(Br)=C5O)=C4)=CC(Br)=C3O)C(Br)=C2
656	467	C[C@@H](CC[C@H]1[C@](C)(O)CC[C@H]2C(C)(C)C(=O)CC[C@]12C)C=C
657	259	CC1=CC2=C(C=C1Br)[C@]1(C)CC[C@H](Br)[C@]1(CO)O2
658	227	C[C@]12CC[C@H]3[C@@](C)(CC[C@H]4C(C)(C)[C@@H](Br)CC[C@]34C)C[C@H]1O2
659	1656	CC1(C)C[C@@H]2[C@@H](O)[C@@]34O[C@@H]3C(=O)[C@@]3(CO3)[C@@]4(C)[C@@H]2[C@@H]1O
660	126	CC[C@H](Br)[C@H]1C[C@H](OC(C)=O)[C@@H](Cl)C[C@H](Cl)[C@H](C\C=C\C#C)O1
661	1294	CC(=O)O[C@H]1CC(C)(C)C(=C=C\C(C)=C\C=C\C(\C)=C\C=C\C=C(/C)\C=C\C=C(/C)C(=O)C[C@@]23O[C@]2(C)C[C@@H](O)CC3(C)C)[C@](C)(O)C1
662	821	CC(C)C[C@@H](N)[C@@H](O)C(=O)N[C@H](C(C)C)C(=O)N[C@@H](C(C)C)C(=O)N[C@@H](CC([O-])=O)C(N)=O
663	1354	COC1=C(Br)[C@H](O)[C@]2(CC(=NO2)C(=O)NCCCCNC(=O)C2=NO[C@]3(C2)C=C(Br)C(=O)[C@H](Br)[C@H]3O)C=C1Br
664	1564	C\C(=C\C[C@@H]1C(=C)CC[C@H](Br)C1(C)C)[C@@H](O)CBr
665	505	CC(C)=CCC\C(=C\CC\C(C)=C\CC\C(C)=C\CC1=CC(=O)C=C(C)C1=O)C([O-])=O
666	267	CC1(C)[C@H](Br)[C@H](O)CC(=C)[C@@]11CC[C@@](O)(C=C1)C(Br)Br
667	937	CC(C)C1=CC[C@]2(C)[C@@H]1CC[C@H](OC(C)=O)\C(C)=C1\CCC(C=O)=C[C@@H]21
668	658	C\C(=C/C=C/C(C)(C)O)[C@H]1CC[C@]2(C)[C@@H](Br)CCC(=C)[C@@]2(O)C1
669	674	CC1=C(Br)C=C(C(O)=C1Br)[C@]1(C)CC[C@@H]2C[C@]12C
670	213	C[C@H]1CCC\C=C/[C@H]2CC[C@H](O)[C@H](CC(=O)O1)O2
671	1212	CC[C@H]1OC(=O)C[C@@H](O)[C@H](C)[C@@H](O[C@@H]2O[C@H](C)[C@@H](O[C@H]3CC[C@H](O)[C@@H](C)O3)[C@@H]([C@H]2O)N(C)C)[C@@H](CC=O)C[C@@H](C)C(=O)\C=C/C(/C)=C/[C@@H]1CO[C@@H]1O[C@H](C)[C@@H](O)[C@H](OC)[C@@H]1OC
672	1040	CC1(C)O[C@]2(C)CC[C@@H]1OC(=O)C1=CC=C(O)C(C[C@@H]3C(=C)CC[C@H](Br)[C@@]3(C)CC[C@H]2Br)=C1
673	843	O[C@H](\C=C\CCCCCCCCC\C=C/CCCCCCCCC\C=C\[C@@H](O)C#C)C#C
674	1222	OC1=C(OC2=C(Br)C=C(Br)C=C2)C(Br)=C(Br)C(Br)=C1
675	1423	COC1=C(OC)C(=O)OC(CCCCCCCCCCSSCCCCCCCCCCC2=C(C)C(OC)=C(OC)C(=O)O2)=C1C
676	436	C[C@H](CC[C@@H](O)C(C)=C)[C@@H]1CC[C@@H]2[C@H]3C[C@@H](O)C4=CC(=O)CC[C@@]4(C)[C@@H]3CC[C@@]12C
677	1358	C[C@@H](Br)[C@]1(C)CC[C@@H](C)[C@H](C\C=C\C=C)O1
678	851	O[C@H](\C=C\CCCCCCCCCCC\C=C/CCCCCCCCC\C=C\[C@@H](O)C#C)C#C

679	873	CO[C@H]1CC\C=C(\C)/C=C/C[C@H](OC)\C=C(/C)\C=C\[C@@H](C)\C=C\C(=O)O[C@@H]([C@@H](C)\C=C\C=C\1)C(\C)=C\C=C(/C)CNC(=O)[C@H](CO)NC=O
680	1498	OC\C=C/C=O
681	1069	CC(=O)O[C@@H]1C[C@@H]2[C@@]3(C)CCCC(C)(C)[C@@H]3CC[C@@]2(C)[C@@H]2C[C@H](O)C3=CC(=O)O[C@@H]3[C@@]12C
682	1383	CC1(C)[C@@H](Br)[C@H](O)CC(=C)[C@]11CC[C@](C)(Br)[C@@H](Cl)C1
683	1270	OCC1=CC(O)=C(O)C(Br)=C1CC1=C(Br)C(Br)=C(O)C(O)=C1
684	795	CC1(C)[C@@H](Br)CC[C@]2(C)O[C@@H](CBr)C(CO)=CC[C@@H]12
685	952	CC(C)[C@@]1(O)CC[C@@]2(C)C\C=C(C)/[C@H](O)C\C=C(C)/C(=O)C[C@H]12
686	1484	CC1=CC(=O)C2=C(C[C@@H]3[C@]2(C)CC[C@H]2[C@]3(C)CC[C@H]3C(C)(C)[C@@H](O)CC[C@@]23C)C1=O
687	915	C[C@H]1C[C@H](C)C[C@H](C)[C@@H](O)C(=C\C=C\C[C@@H](OC(=O)C[C@H](O)[C@H](C)C1)[C@@H]1CCC[C@H]1C([O-])=O)C#N
688	1013	CC(C)C[C@@H](N)[C@@H](O)C(=O)N[C@@H](C(C)C)C(=O)N[C@@H](C(C)C)C(=O)N[C@H](CCC(N)=O)C([O-])=O
689	1427	CCCCCCCCCCCCCCCCCCCCCCC[C@@H](O)C(=O)N[C@H](CO[C@H]1O[C@H](CO)[C@@H](O)[C@@H](O)[C@@H]1O)[C@H](O)[C@H](O)C\C=C\CCCC[C@@H]1C[C@H]1CCCC
690	168	C[C@]12CC[C@@H](C[C@@]3(O)CC[C@H](Br)C(C)(C)C3)[C@@H]1[C@](C)(O)CC[C@@H]2Br
691	703	C[C@H]1CC[C@@H]2[C@@]3(C)CC[C@H](O)C(C)(C)[C@@H]3CC[C@@]2(C)[C@@]11CC2=C(O1)C(C)=CC(O)=C2
692	924	CC(C)C1=C2CC\C(C)=C\[C@@H](O)C\C(C)=C\C[C@]2(C)CC1
693	281	CC1=CC(O)=C(C=C1)[C@]1(C)CC[C@@](C)(O)C1=C
694	1140	CC1=CC[C@](C)(O)C(=C)[C@@]11CC[C@@](C)(O)C=C1
695	343	CC(=C)[C@H]1CC[C@](C)(O)[C@@H](O)[C@@H]1CC(=C)[C@@H]1CC[C@](C)(O)[C@H](Br)C1
696	552	CC(C)C1=CC[C@]2(C)[C@@H]1CC[C@H](OC(C)=O)\C(C)=C1\CCC(=C[C@@H]21)C([O-])=O
697	909	OC1=CC([C@@H]2OCC3=CC(O)=C(O)C(Br)=C23)=C(Br)C(Br)=C1O
698	1167	CC1=CC(=CC(=C1N)S([O-])(=O)=O)C(C1=CC=C(NC2=CC=C(C=C2)S([O-])(=O)=O)C=C1)=C1C=CC(C=C1)=NC1=CC=C(C=C1)S([O-])(=O)=O
699	1075	O\N=C1/CC2=CC(Br)=C(OC3=CC(C\C(=N/O)C(=O)NC=CC4=CC=C(Br)C(OC5=CC=C(CCNC1=O)C(Br)=C5O)=C4)=CC(Br)=C3O)C(Br)=C2
700	574	CC[C@@H](Br)[C@H]1C[C@H](Br)[C@@H]2C[C@H](O1)[C@H](C\C=C\C#C)O2
701	852	CC\C=C/C[C@H](O)\C=C\C=C/C\C=C/C\C=C/CCCC(=O)C1=C(O)C=C(O)C=C1O
702	422	C[C@H]1CC[C@@]2(C)[C@H](CCCC2=C)[C@]1(C)CC1=C(OS([O-])(=O)=O)C(O)=CC(C=O)=C1
703	664	CCCCCCCCCC\C=C/[C@H](O)CC[C@@H](O)[C@H](N)CO
704	1110	CC(C)[C@@H]1CC[C@@](C)(O)[C@@H]2CCC(C)=C[C@@H]12
705	58	O[C@H](\C=C\CCCCCCCCC\C=C/CCCC\C=C/CCCCC\C=C\[C@@H](O)C#C)C#C
706	1597	CC[C@@H](C)[C@H]1[C@@](C)(O)C(=O)[C@@H]2C[C@@](O)(CO)C[C@@H](C)[C@H]2[C@@]1(C)C(=O)CCO
707	26	CC(C)=CCC[C@]1(C)[C@@H](Br)CC[C@@](C)(O)[C@H]1CC[C@@](C)(O)C=C
708	1560	CC[C@@H](Br)[C@@H]1C[C@H](OC(C)=O)[C@@H](Cl)C[C@H](Cl)[C@H](C\C=C/C#C)O1
709	73	CC(=C)[C@@H]1CC[C@](C)(O)[C@H](Br)CC[C@]2(C)[C@@H](Br)CC=C(C)[C@H]2CC2=CC(=CC=C2O)C(=O)O1
710	373	CC(C)=CC\C=C(/C)\C=C\C\C(C)=C\CC\C(C)=C\CO

711	324	CC(C)=CCC\C(C)=C\CC\C(C)=C\CC\C(C)=C\CC1=CC(=O)C=C(C)C1=O
712	1298	CC[C@@H]1O[C@H]([C@H](Br)[C@H]2O[C@H](C[C@H]2O)[C@@H](Br)C#C)[C@@H](Br)C[C@H]1Br
713	1504	CC(=C)[C@H]1CC[C@@](C)(O)[C@H](O)[C@@H]1CC(=C)[C@@H]1CC[C@](C)(O)[C@H](Br)C1
714	1552	COC1=CC(C\C=C(/C)CC2=C(CC(C)(C)O)C(=O)[C@]3(C)CCC[C@]3(C)C2)=C(OC)C(C)=C1
715	1628	CCCCCC\C=C/CC(=O)N[C@@H](CC1=CC=CC=C1)[C@@H](O)CC(=O)N[C@@H](C(C)C)C(=O)N[C@@H](CO)[C@H](O)CC(=O)N[C@@H](C(C)C)C(=O)N[C@@H]1COC(=O)[C@H](CC2=CC=C(O)C=C2)N(C)C(=O)[C@@H](NC(=O)\C=C/1)C(C)C
716	56	CC[C@H]1O[C@@H]2C[C@@](O)(O[C@H]2C[C@H]1Br)[C@H](Br)C\C=C\C#C
717	1381	C[C@H]1\C(CC[C@@]1(C)C1=C(O)C=C(C)C(Br)=C1)=C\Br
718	686	O\N=C1/CC2=CC(Br)=C(OC3=CC(C\C(=N/O)C(=O)NCCC4=CC=C(Br)C(OC5=CC=C(CCNC1=O)C(Br)=C5O)=C4Br)=CC(Br)=C3O)C(Br)=C2
719	481	CC1(C)[C@@H](Br)CCC(=C)[C@H]1CC[C@]1(C)[C@@H](Br)CCC2=COC3=CC=C(C=C3C[C@@H]12)C([O-])=O
720	1025	CC1=CC[C@H]2C(C)(C)[C@@H](Br)CC[C@]2(C)O[C@H]1CBr
721	772	CC(C)[C@@H]1C=C[C@H](O)[C@H]2[C@@H]3[C@](C)(O)CC[C@H](Br)[C@@]3(C)CC[C@]12CBr
722	1213	CC1(C)C[C@@H](CC[C@@H]1Br)[C@]12CC[C@](CO)(OO1)C=C2
723	290	OCC1=C(CC2=CC(Br)=C(O)C=C2)C(O)=C(O)C(Br)=C1CC1=C(CC2=CC(Br)=C(O)C=C2)C(O)=C(O)C(Br)=C1
724	462	O[C@@H](CCCCCCCCCCC\C=C/CCCCCCCCC\C=C\[C@@H](O)C#C)\C=C/C#C
725	786	C[C@@]1(CC[C@@]2(O)C(=C1)C(=O)C(O)=C1[C@](C)(CO)CC[C@@H](O)[C@]21C)C=C
726	1067	CC1=C2CC3=CC(=CC=C3O)C(=O)O[C@@H](CC[C@]3(C)O[C@H]3CC=C(C)[C@@H]2CC1)C(C)(C)O
727	1704	CCC[C@@]1(C[C@@H](O[C@H]2CC[C@@H](OC)[C@H](C)O2)[C@H](O1)[C@]1(C)CC[C@]2(C[C@H](OC)[C@@H](C)[C@H](O2)[C@@H](C)[C@@H](OC)[C@H](CC)C([O-])=O)O1)[C@H]1O[C@H](C[C@@H]1C)[C@H]1O[C@@](O)(CO)[C@H](C)C[C@@H]1C
728	209	CC(C)=CCC[C@](C)(O)[C@H]1CC[C@]2(C)O[C@H](CC[C@H]2O1)C(=C)CC[C@@H](OC(C)=O)[C@]1(C)CC[C@@H](O1)C(C)(C)O
729	489	CC1=C2CC3=CC(=CC=C3O)C(=O)OC(C)(C)[C@H](Br)CC[C@](C)(O)[C@H](Br)CC[C@]2(C)[C@@H](Br)CC1
730	1430	CC(C)[C@H](NC(=O)[C@H](C)NC(=O)[C@H](CCCC1=CC=C(O)C=C1)NC(=O)[C@H](O)CO)C(=O)N[C@H]1[C@@H](C)OC(=O)[C@@H](NC(=O)[C@H](CC2=CC(Br)=C(O)C=C2)N(C)C(=O)[C@H]([C@H](C)O)N2[C@H](O)CC[C@H](NC(=O)[C@H](CCC3=CC=C(O)C=C3)NC1=O)C2=O)C(C)C
731	604	CC1=C(Br)C=C(C(O)=C1I)[C@]1(C)CC[C@@H]2C[C@]12C
732	1567	COC1=CC=C(C[C@H](N(C)C(=O)[C@H](C)N(C)C(=O)[C@H](C)NC(=O)[C@H](CC2=CC=CC=C2)N(C)C(=O)[C@@H](C)CCCCC#C)C(N)=O)C=C1
733	765	CC(C)[C@@H]1C=C[C@H](OO)[C@H]2[C@@H]3[C@](C)(O)[C@@H](O)C[C@H](Br)[C@@]3(C)CC[C@]12CBr
734	101	CC1(C)[C@@H](Br)[C@@H](O)CC(=C)[C@]11CC[C@](C)(Br)[C@@H](Cl)C1
735	398	C[C@@H]1CCC\C=C/[C@@H]2O[C@H](C[C@@H](O)C\C=C/C=C\[C@@H](O)C\C=C/C=C\C(=O)O1)[C@H]2O
736	1107	CC[C@H](Br)[C@H]1C[C@H](OC(C)=O)[C@@H](Cl)C[C@H](Cl)[C@H](C\C=C/C#C)O1
737	1456	CCCC(=O)N[C@@H](C(C)C)C(=O)N[C@H]1[C@@H](C)OC(=O)[C@@H](NC(=O)[C@H](CC2=CC(Br)=C(OC)C=C2)N(C)C(=O)[C@H]([C@H](C)CC)N2[C@H](O)CC[C@@H](NC(=O)[C@H](CCCCN)NC1=O)C2=O)C(C)C

738	1580	CC[C@@H]1C[C@@H]2[C@@H]3O[C@@H]3[C@H]3[C@H](C[C@H]4\C=C\C(\O)=C5\C(=O)N[C@@H](CCCNC(=O)\C=C/C[C@H]34)C5=O)[C@H]2[C@H]1C
739	1488	C[C@H]1CC[C@@H]2[C@@]3(C)CC[C@H](O)C(C)(C)[C@@H]3CC[C@@]2(C)[C@]11CC2=C(O1)C(=CC(O)=C2O)C(C)=O
740	1583	CC[C@H]1C[C@@H]2CC[C@@H](O2)[C@H](C)C(=O)O[C@@H](CC)C[C@H]2CC[C@H](O2)[C@@H](C)C(=O)O1
741	1343	CC1=CC[C@@H](Br)C(C)(C)[C@]11CC[C@](C)(Cl)[C@@H](Br)C1
742	142	CC(C)[C@@]1(O)CC[C@@]2(C)C\C=C(C)/CC\C=C(C)/C(=O)C[C@H]12
743	1602	C[C@@H]1O[C@H](OC2=CC=C(\C=C\C(=O)NCCCNCCCCN)C=C2)[C@H](NC(N)=N)[C@@H](O)[C@@H]1NC(=O)N[C@@H]1OC[C@H](O[C@@H]2OC[C@H](O)[C@@H](N)[C@H]2NC(N)=O)[C@@H](O)[C@@H]1NC(N)=O
744	1443	CC1=C2CC3=CC(=CC=C3O)C(=O)OC(C)(C)[C@H](Br)CC[C@]3(C)O[C@H]3CC[C@]2(C)[C@@H](Br)CC1
745	1620	CC[C@@H](C)[C@H]1[C@@](C)(O)C(=O)[C@H]2C[C@](C)(O)C[C@@H](C)[C@@H]2[C@@]1(C)C(=O)CCO
746	254	CC1=CC[C@@H](Br)C(C)(C)[C@@]11CC[C@](C)(Cl)[C@@H](Br)C1
747	595	C[C@@H]1CC[C@@](C)(C1=C)C1=C(O)C=C(C)C(Br)=C1
748	425	CC(C)(O)[C@H]1CC[C@@](C)(O1)[C@@H](O)CC[C@@](O)(CO)C(=O)CC[C@H]1O[C@H](CC[C@]1(C)O)[C@]1(C)CC[C@@H](Br)C(C)(C)O1
749	1175	CC[C@@H](Br)[C@H]1C[C@H](OC(C)=O)[C@@H](Cl)C[C@H](Cl)[C@H](C\C=C/C#C)O1
750	321	C\C=C\C=C\C(=O)C1=C(O)[C@@]2(C)[C@H]3C(C(=O)\C=C\C=C\C)=C(O)C(C)=C(O)[C@@]3(C)O[C@@]2(O)[C@](C)(O)C1
751	1539	CC[C@@H](Br)[C@H]1C[C@@H](Br)[C@H]2C[C@@H](O1)[C@@H](C\C=C/C#C)O2
752	103	C\C(CC\C=C(/C)CCC(=O)C(C)(C)O)=C/CC\C(C)=C\CC1=C(O)C(C)=CC(O)=C1
753	782	COC1=CC(=O)C=C(C[C@@H]2[C@@](C)(O)CC[C@H]3[C@@]2(C)CC[C@H]2[C@]4(C)CCC[C@]32COC4=O)C1=O
754	1308	CCCCCCCC\C=C/CCCCCCCC(=O)OC1=CC(C)=C(O)C(C\C=C(/C)CC\C=C(/C)CC\C=C(/O)CC[C@@H](O)C(C)(C)O)=C1
755	9	CC1=CC[C@H](Br)C(C)(C)[C@@]11CC[C@@](C)(O)[C@H](Br)C1
756	587	COC1=CC=C(C[C@H](N(C)C(=O)[C@H](C)N(C)C(=O)[C@H](C)NC(=O)[C@H](CC2=CC=CC=C2)N(C)C(=O)[C@H](C)C[C@@H](C)CCCCC#C)C(N)=O)C=C1
757	1079	CC1=CC(=O)C2=C(C[C@H]3[C@@]2(C)CC[C@@H]2[C@@]3(C)CC[C@H]3C(C)(C)C(=O)CC[C@]23C)C1=O
758	1016	CC1=CC[C@@H](Br)C(C)(C)[C@]11CC[C@@](C)(Cl)[C@@H](Br)C1
759	377	C\C=C\C[C@](C)(O)C1=CC(=O)C2=C(C)C=C3C(=O)C4=C(C=C([C@H]5C[C@](C)([C@H](O)[C@@H](C)O5)N(C)C)C(O)=C4C(=O)C3=C2O1)[C@H]1C[C@H]([C@H](O)[C@@H](C)O1)N(C)C
760	1475	CC1=CC(=O)C2=C(C[C@H]3[C@@]2(C)CC[C@@H]2[C@]3(C)CC[C@H]3C(C)(C)C(=O)CC[C@]23C)C1=O
761	1451	CC(=C)[C@@H]1CC[C@](C)(O)[C@H](Br)CC[C@@H]2C(C)=CCC(C)=C2CC2=CC(=CC=C2O)C(=O)O1
762	1171	CC(C)[C@@H]1CC=C[C@H]2[C@@H]3[C@](C)(O)CC[C@H](Br)[C@@]3(C)CC[C@]12CBr
763	87	NC1=N[C@H]2N3C(Br)=C(Br)C=C3C(=O)N3CCC[C@]23N1
764	1523	[O-]P([O-])(=O)C(=O)CCl
765	1153	CO[C@@H]1C[C@@]2(OC1(C)C)OC(C\C(C)=C\CC1=C(OC)C(C)=CC(OC)=C1)=C[C@]1(C)CCC[C@]21C
766	364	C[C@](O)(CC[C@H](Br)[C@@]1(C)CC[C@H](Br)C(C)(C)O1)C=C

767	1555	C[C@H](CC[C@@H](O)C(C)=C)[C@@H]1CC[C@@]2(C)[C@H](O)CC=C(C=O)[C@@]12C=O
768	1664	CC(=O)OC[C@]12C[C@H]1[C@@H](C[C@]1(C)[C@@H]2C[C@@H](O)[C@@H]2C[C@](C)(CC=C12)[C@H]1CO1)OC(C)=O
769	527	C[C@H]1CC[C@@](C)(O)[C@@]2(CC[C@@](C)(Cl)[C@H](Br)C2)C1=C
770	1066	C[C@@]12C[C@](C)(CCCCCC\C=C\C=C\C3=CC=CC=C3)OO[C@@H]1CC(=O)O2
771	233	CC(=O)N(O)C[C@H](O)CP([O-])([O-])=O
772	328	C[C@H](CCCC1=COC=C1)\C=C\C=C(/C)CCC[C@@H](C)\C=C1\OC(=O)C(C)=C1O
773	15	CC[C@H](C)[C@@H]1NC(=O)[C@H](C)NC(=O)[C@H](CC[S@@](C)=O)NC(=O)[C@H](C)NC(=O)[C@H](CC(=O)OC)NC(=O)[C@H](CC2=CC=CC=C2)NC(=O)[C@@H]2CCCN2C1=O
774	723	CC1=C2O[C@]3(C)CC[C@@H]4[C@@]5(C)CC[C@H](O)C(C)(C)[C@@H]5CC[C@@]4(C)[C@H]3CC2=CC(O)=C1
775	1290	CC1=C2O[C@@]3(C)CC[C@H]4[C@@]5(C)CC[C@H](O)C(C)(C)[C@@H]5CC[C@]4(C)[C@@H]3CC2=CC(O)=C1
776	1527	BrC1=C(Br)C(Br)=C(N1)C1=C(Br)C(Br)=C(Br)N1
777	713	COC(=O)C[C@H](NC(=O)[C@@H](NC(=O)[C@H](N\C1=N/CC(=O)N2CCC[C@H]2C(=O)N[C@@H](C(C)C)C(=O)N[C@H]1C(C)(C)C)C(C)(C)C)[C@H](C)C1=CC=CC=C1)C1=NC=CS1
778	457	CC(=O)OC[C@]12C[C@H]1[C@@H](C[C@]1(C)[C@@H]2C[C@@H](O)[C@@H]2C[C@](C)(CC=C12)[C@@H](Br)CO)OC(C)=O
779	1131	CC(C)[C@@]1(O)CC[C@@]2(C)C\C=C(C)/[C@@H](C\C=C(C)/C(=O)C[C@H]12)OC(C)=O
780	1202	C[C@H]1CCC\C=C/[C@@H]2O[C@@H](CC(=O)O1)[C@@H](O)C=C2
781	1623	CO[C@H]1C[C@](O)(O[C@@H](C(C)C)[C@@H]1C)[C@@H](C)[C@H](O)[C@H](C)[C@H]1OC(=O)\C(OC)=C\C(\C)=C\[C@H](C)[C@H](O)[C@@H](C)C\C(C)=C/C=C/[C@@H]1OC
782	736	CC(C)[C@H]1CC[C@H](C)[C@@]2(CCC(C)=C2)[C@H]1O
783	545	COC1=C(C)C(=O)[C@]23[C@@H]([C@@H](COC(N)=O)[C@@]4(OC)[C@@H]5[C@H](CN24)N35)C1=O
784	1508	CC(C)=CC[C@@H](O)C(\CO)=C\CC\C(C)=C\CC\C(C)=C\CC1=CC(=O)C=C(C)C1=O
785	588	CC(C)[C@@H]1C=C[C@H](OO)[C@H]2[C@@H]3[C@](C)(O)[C@H](O)C[C@H](Br)[C@@]3(C)CC[C@]12CBr
786	152	CC[C@@H]1O[C@@]2(C)CC[C@H](Br)C(C)(C)[C@@H]2CC=C1C
787	1442	CC\C=C\[C@@H](CC)CCC[C@@]1(CC)C[C@H](CC)[C@H](CC([O-])=O)OO1
788	289	CC[C@H](CO)\C=C(\C)[C@H]1C[C@@H](C[C@@H](O)C(C)(C)[C@]2(O)O[C@@H](C[C@H](OC)[C@@H]2O)C[C@H](OC)[C@@H](O)C(=O)O1)OC
789	359	CC1(C)[C@@H](Br)CC[C@]2(C)O[C@](C)(CC[C@@H]12)C=C
790	1493	CCCCC[C@@H](O)[C@H]1C(=O)N[C@@]([C@@H](O)[C@H]2CCCC=C2)(C([O-])=O)[C@]1(C)O
791	336	CC1=C2O[C@]3(C)CC[C@@H]4[C@@]5(C)CC[C@H](O)C(C)(C)[C@@H]5CC[C@@]4(C)[C@@H]3CC2=CC(O)=C1
792	982	CCCCC[C@H]1C[C@@H]2CCC[C@@H](C[C@H](C[C@@H]3C[C@H](C[C@H](CC(=O)O1)O3)OC(=O)\C=C/CCC1=COC(\C=C/CNC(=O)OC)=N1)OC)O2
793	512	CC(=O)O[C@@H]1C=C(C)[C@@H]2[C@H]3C\C(C)=C/CC[C@](C)(OC(C)=O)[C@H](O3)[C@@H]2[C@@H]1C(C)(C)OC(C)=O
794	1413	CSC1=C(NC2=CC(Br)=C(Br)C(Br)=C12)[S@](C)=O
795	1236	CC1=CC[C@H](Br)[C@@]2(C)CC[C@@H](Br)[C@@](C)(O)CC[C@H](OC(=O)C3=CC=C(O)C(C[C@H]12)=C3)C(C)(C)Br
796	868	OC1=CC(CC2=CC(O)=C(O)C(Br)=C2Br)=C(Br)C(Br)=C1O

797	1718	CC[C@@]1(O)C[C@H](O[C@@H]2C[C@H]([C@@H](O[C@H]3C[C@H](O)[C@@H](O[C@@H]4CCC(=O)[C@H](C)O4)[C@@H](C)O3)[C@@H](C)O2)N(C)C)C2=C(O)C3=C(C=C2[C@H]1C(=O)OC)C(=O)C1=CC=CC(O)=C1C3=O
798	347	CCCC[C@@H](C)[C@H]1CC(=O)N[C@@H](CC2=CC=CC=C2)C(=O)N[C@H](CC2=CC=CC=C2)C(=O)N[C@H]([C@@H](C)CC)C(=O)O1
799	1004	CC[C@H](C)[C@H](NC(C)=O)C(=O)N[C@H](CC1=CC=C(O)C=C1)C(=O)N[C@@H](CC1=CC=C(O)C=C1)P([O-])([O-])=O
800	418	C[C@H]1[C@](C)(O)CC[C@@]1(C)C1=C(O)C=C(C)C=C1
801	606	C[C@@H]1CC[C@@]2(CC1=O)C(=C)CC=CC2(C)C
802	1203	C[C@]12CC[C@H]3[C@@](C)(CC[C@H]4C(C)(C)CCC[C@]34C)C[C@H]1O2
803	329	CC1=C2O[C@]3(C)CC[C@H]4[C@@]5(C)CC[C@H](O)C(C)(C)[C@@H]5CC[C@]4(C)[C@H]3CC2=CC(O)=C1
804	218	CC1(C)[C@@H](Br)CC[C@]2(C)O[C@@H](CBr)C(=C)[C@@H](O)C[C@@H]12
805	129	CC1(C)C[C@H](CC[C@@H]1Br)[C@@]12CC[C@@](CO)(OO1)C=C2
806	1283	C\C(CC\C=C(/C)CC[C@@H](O)C(C)(C)O)=C/CC\C(C)=C\CC1=C(O)C(C)=CC(O)=C1
807	1407	CC1(C)[C@@H](Br)CC[C@]2(C)O[C@@H](CBr)C(=C)[C@H](O)C[C@@H]12
808	382	[O-][N+](=O)C1=C(C=CC=C1Br)C1=CNC(Br)=C1Br
809	1614	CO[C@H]1\C=C/C=C(C)/C[C@@H](C)[C@H](OC(C)=O)[C@H](C)\C=C(/C)\C=C(OC)/C(=O)O[C@@H]1[C@H](C)[C@H](O)[C@H](C)[C@]1(C[C@H](OC(C)=O)[C@@H](C)[C@@H](O1)C(C)C)OC
810	1666	C[C@H]1CCCC[C@H](CCCC(Cl)Cl)C2=C(O)C=C(C=C2O)[C@H](OC(N)=O)[C@@H](C)CCCC[C@@H](CCCC(Cl)Cl)C2=C(O)C=C(C=C2O)[C@@H]1OC(N)=O
811	397	CC1(C)O[C@@](C)(CC[C@H]1Br)[C@H]1CC[C@@](C)(Br)[C@H](Cl)C1
812	476	C[C@](O)(CCCCCCCC\C=C\C=C\C1=CC=CC=C1)C[C@]1(C)OC(=O)C[C@@H]1O
813	1261	C[C@]12C[C@](C)(CCCCCCCC\C=C\C=C\C3=CC=CC=C3)OO[C@H]1CC(=O)O2
814	110	C[C@](Br)(CCl)[C@H]1C[C@H](O)[C@@](C)(O1)C(\Cl)=C/Br
815	439	COC1=C(O)C(C)=C(C\C=C(/C)CCCCCCC2=CC=CC=C2)OC1=O
816	741	CCCC[C@@H](C)[C@H]1CC(=O)N[C@H](CC2=CC=CC=C2)C(=O)N[C@@H](C(C)C)C(=O)N[C@H](C(C)C)C(=O)O1
817	251	CC1(C)C[C@H](CC[C@@H]1Br)C1=CC=C(CO)C=C1
818	906	NC[C@H](O)C[C@@H]1NC(=O)[C@@H](N)CC2=CC(=CC=C2O)C2=CC=C(O)C(C[C@@H](NC1=O)C([O-])=O)=C2
819	1540	CC(=O)O[C@H]1CC(C)(C)C(=C=C\C(C)=C\C=C\C(\C)=C\C=C\C=C(/C)\C=C\C=C(/C)C(=O)C[C@]23O[C@@]2(C)C[C@H](O)CC3(C)C)[C@@](C)(O)C1
820	154	C[C@H](CCC=C(C)C)[C@@H]1CC[C@](C)(O)[C@@H]2CC=C(C)[C@@H]2[C@@H]1O
821	1149	COC(=O)CC[C@]1(C)[C@@H]2CC3=C(O[C@@]2(C)C[C@@H](O)[C@H]1C(C)(C)O)C(C)=C1COC(=O)C1=C3OC
822	107	CC(C)[C@H]1CC(C)(C)[C@H](O)C2=C1CC[C@H]2C
823	304	C[C@@]12C[C@](C)(CCCCCCCC\C=C\C=C\C3=CC=CC=C3)OO[C@@H]1CC(=O)O2
824	1556	C[C@@H]1CC=C(C)[C@]2(CC[C@@](C)(O)[C@@H](Br)C2)C1=C
825	1047	CC(C)C[C@@H]1NC(=O)[C@@H](NC(=O)[C@@H](NC(=O)[C@H](C)NC(=O)[C@H](CCCC2=CC=C(O)C=C2)NC(=O)[C@H](O)CO)C(C)C)[C@@H](C)OC(=O)[C@@H](NC(=O)[C@H](CC2=CC(Br)=C(O)C=C2)N(C)C(=O)[C@H]([C@H](C)O)N2[C@H](O)CC[C@H](NC1=O)C2=O)C(C)C
826	273	CC[C@H]1CCC[C@@](C)(COC(C)=O)[C@H]2C=C[C@H](C)[C@@H]12

827	677	COC1=C(Br)C=C(C=C1O)[C@@H](CC1=C(Br)C(Br)=C(O)C(O)=C1)C([O-])=O
828	849	CCCCCCCCCCCCCC\C=C(/CCCCCCCCCCCCC)C=O
829	1566	CC(C)[C@H]1CC[C@@]2(C)[C@H](Br)CC=C(C)[C@@H]2[C@H]1O
830	493	C[C@H]1CC[C@@H]2[C@@]3(C)CC[C@H](O)C(C)(C)[C@@H]3CC[C@@]2(C)[C@]11CC2=C(O1)C(C)=CC(O)=C2
831	147	CC[C@H]1O[C@@H]2C[C@H](O[C@@H]2C[C@@H](O)[C@H](O)C[C@@H]1Br)C=C=CBr
832	942	COC1=C(C)C(=O)C2=C([C@@H](COC(N)=O)[C@@]3(NC[C@H]4[C@@H]3N24)OC)C1=O
833	1617	CC1=CC(O)=C2C(=O)OCC3=C2C1=C1[C@H]2[C@@H](O)[C@@]4(COC(=O)C5=C(O)C=C(C)C(C2=O)=C45)C1=C3O
834	1193	CC(C)C\C=C\[C@@H](C)[C@H]1CC[C@@H]2[C@]1(C)CC[C@@H]1[C@]34CO[C@]21C[C@@H](O)[C@@]3(O)C[C@H](CC4)OS([O-])(=O)=O
835	275	C[C@](O)(CCCCCCCCCCCCC1=CC=CC=C1)C[C@@]1(C)OC(=O)C[C@H]1O
836	23	CN1C=C(Br)C2=CC(Br)=CC=C12
837	1641	C[C@H](CCC=C(C)C)[C@@H]1CC[C@]2(C)O[C@H]2C\C=C2\CO[C@H](O)[C@@H]12
838	1313	CC\C(Br)=C/C[C@H]1O[C@@H](C\C=C/C#C)[C@@H](Cl)C[C@H]1Br
839	332	CC(C)(C=C)C1=C2[C@@H]3[C@H](CC[C@](C)(C=C)[C@@H]3[N+]#C)C(C)(C)C3=C2C(N1)=CC=C3
840	667	C[C@@]1(CC=C2[C@@H](C1)[C@H](O)C[C@@H]1[C@@]3(O)CC[C@H]3[C@H](O)C[C@@]21C)[C@@H](Br)CO
841	754	[O-][N+](=O)C1=C(C=C(Br)C=C1)C1=CNC(Br)=C1Br
842	333	CCCCCCCCCCCCCCCCCC(=O)OC1=CC(C)=C(O)C(C\C=C(/C)CC\C=C(/C)CC\C=C(/O)CC[C@@H](O)C(C)(C)O)=C1
843	799	COC1=C(Br)C=C(C=O)C=C1O
844	92	COC1=C(Br)C=C(C=C1O)[C@H](C([O-])=O)C1=C(Br)C(Br)=C(O)C(O)=C1
845	702	C\C(CC\C=C(\CC[C@H](Cl)C(C)=C)C([O-])=O)=C/CC[C@@]1(C)OC2=C(C)C=C(O)C=C2C=C1
846	1096	CC1=C2O[C@@]3(C)CC[C@@H]4[C@@]5(C)CC[C@H](O)C(C)(C)[C@@H]5CC[C@@]4(C)[C@@H]3CC2=CC(O)=C1
847	46	COC1=C(O)C(C)=C(C\C=C(/C)CCCCCCCC2=CC=CC=C2)OC1=O
848	1611	CS[C@@H]1N(C)C(=O)[C@@](CC2=CC=C(O)C=C2)(SC)N(C)C1=O
849	973	CC[C@@H](Br)[C@@H]1C[C@H](OC(C)=O)[C@@H](Cl)C[C@@H](OC(C)=O)[C@H](C\C=C/C#C)O1
850	837	CCC[C@@H](OC(C)=O)C1=C(Br)[C@](C)(OC)OC1=O
851	262	C[C@@]12O[C@@H]1C[C@@H](Br)C(C)(C)[C@]21CC[C@@](C)(Cl)[C@@H](Br)C1
852	1226	O[C@H]1CC[C@H](O)[C@@H]2[C@@H]1C[C@@]13SS[C@]4(C[C@H]5[C@@H]([C@@H](O)CC[C@@H]5O)N4C1=O)C(=O)N23
853	118	C[C@@H]1CC[C@H]2[C@@H]1[C@@H]1[C@H](CC(=O)[C@]21C)C(=C)[C@@H](O)\C=C\[C@@](C)(O)CO
854	665	CC1=C2CC3=CC(=CC=C3O)C(=O)OC(C)(C)[C@@H](O)CC[C@](C)(O)[C@H](Br)CC[C@]2(C)[C@@H](Br)CC1
855	192	CC[C@@H](Br)[C@H]1C[C@H](OC(C)=O)[C@@H](Cl)C[C@@H](OC(C)=O)[C@H](C\C=C/C#C)O1
856	711	COCC1=CC(O)=C(O)C(Br)=C1CC1=C(Br)C(Br)=C(O)C(O)=C1
857	338	C[C@@H]1CC[C@H]2[C@@H]1[C@@H]1[C@H](C[C@@H](O)[C@]21C)C(=C)CC[C@@H](O)C(C)=C
858	737	C[C@]1(O)CC[C@H](Br)[C@@]2(C)CC[C@]3(C[C@@H]12)C(=C)CC[C@@H](Br)C3(C)C
859	173	CC(C)[C@@H]1CC=C[C@H]2[C@@H]3[C@](C)(O)[C@H](O)C[C@H](Br)[C@@]3(C)CC[C@]12CBr
860	789	CC1=CC2=C(C=C1Br)[C@]1(C)CC[C@@](C)(O)[C@]1(C)O2

861	610	CC1=CC=C(Br)C(C)(C)[C@@]11CC[C@@](C)(Cl)[C@H](Br)C1
862	1187	CC1=C[C@H](O)[C@H](Br)C(C)(C)[C@@]11CC[C@](C)(Br)[C@@H](Cl)C1
863	247	CC1=C(Br)C[C@@]2(CC1)C(C)=C[C@H](O)[C@H](Br)C2(C)C
864	248	CC(C)[C@H](NC(=O)[C@H](C)NC(=O)[C@H](CCCC1=CC=C(O)C=C1)NC(=O)[C@H](O)CO)C(=O)N[C@H]1[C@@H](C)OC(=O)[C@@H](NC(=O)[C@H](CC2=CC(Br)=C(O)C=C2)N(C)C(=O)[C@H]([C@H](C)O)N2[C@H](O)CC[C@H](NC(=O)[C@H](CC3=CC=C(C)C=C3)NC1=O)C2=O)C(C)C
865	963	C[C@H](O)[C@@H](NC(=O)[C@H](N)CCCNC(N)=N)C(=O)N[C@@H](CC1=CC=CC=C1)C([O-])=O
866	563	CC1=C(Cl)C[C@]2(CC1)C(=C)CC[C@H](Br)C2(C)C
867	749	CC[C@H]1O[C@H](C[C@H](Br)[C@@H](C[C@@H]1Br)OC(C)=O)[C@H](Cl)C\C=C/C#C
868	877	C[C@]12CC[C@@H](Br)[C@@](C)(O)CC[C@@H](Br)C(C)(C)OC(=O)C3=CC=C(O)C(C[C@@H]1C(=C)CC[C@@H]2Br)=C3
869	232	CCC[C@@H](OC(C)=O)C1=C(Br)[C@@](C)(OC)OC1=O
870	1388	CC1(C)O[C@](C)(CC[C@@H]1Br)[C@H]1CC[C@@](C)(Cl)[C@H](Br)C1
871	867	CC(C)=CCC\C(C)=C\CC[C@]1(C)[C@@H](Br)CC=C2COC3=CC=C(C=C3C[C@@H]12)C([O-])=O
872	1072	C[C@]1(CCCCCCCCCCCCC2=CC=C(O)C=C2)C[C@]2(C)OC(=O)C[C@@H]2O1
873	416	CC1(C)O[C@@](C)(CC[C@H]1Br)[C@H]1CC[C@@](C)(Cl)[C@H](Br)C1
874	144	C\C(=C/C[C@H]1C(=C)CC=CC1(C)C)[C@@H](O)CBr
875	388	CC(C)=CC[C@H](O)C(\C)=C\CC\C(C)=C\CC\C(C)=C\CO
876	1626	CN([C@H]1[C@@H](O)[C@H](OC(N)=O)[C@@H](CO)O[C@@H]1NC1=N[C@@H]2[C@H](N1)C(=O)NC[C@@H]2O)C(=O)C\N=C\N
877	880	CC(C)=CCC\C(C)=C\[C@@H](O)[C@@H](O)C(\C)=C\CC\C(C)=C\CC1=CC(=O)C=C(C)C1=O
878	484	C[C@]12CC[C@@H](Br)[C@@](C)(O)CC[C@H](O)C(C)(C)OC(=O)C3=CC=C(O)C(C[C@@H]1C(=C)CC[C@@H]2Br)=C3
879	278	C\C=C\C=C/[C@H]1O[C@@H](C[C@@H]1OC(C)=O)[C@@H](Cl)C\C=C/C#C
880	1673	C[C@@H]1O[C@H](C[C@H]([C@H]1O)N(C)C)OC1=C2C(=CC(C)=C1[C@H]1C[C@@H]([C@H](O)[C@@H](C)O1)N(C)C)C=C(O)C1=C2C(=O)C2=CC=CC(O)=C2C1=O
881	1184	CC1(C)O[C@](C)([C@@H](O)C[C@H]1Br)[C@H]1CC[C@](C)(Br)[C@H](Cl)C1
882	523	CC[C@@H]1O[C@H](C[C@@H](OC(C)=O)[C@@H](Cl)C\C=C\C#C)[C@H](Cl)C[C@H]1Br
883	807	COC1=CC(C=O)=CC(Br)=C1O
884	1588	C[C@H]1[C@]2(C)CC[C@@]1(C)C1=CC(Br)=C(C)C=C1O2
885	927	COC(=O)CC[C@@H]1C(=O)CC[C@@H]2[C@@](C)(CC3=C(O)C(C)=CC(OC)=C3)[C@@H](C)CC[C@@]12C
886	993	CC1=C[C@H](O)[C@@H](Br)C(C)(C)[C@]11CC[C@@](C)(Cl)[C@@H](Br)C1
887	1538	CC1(C)[C@H](Br)CC(=O)[C@@]2(C)[C@@H]3CC[C@@](C)(O[C@@]3(C)C[C@H](O)[C@H]12)C=C
888	1015	C[C@H](NC(=O)[C@@H](N)CO)C(=O)N[C@@H](CO)C(=O)N[C@@H](CCCNC(N)=N)[C@@H](O)CC(=O)OCC1=C(N2[C@@H](SC1)[C@](NC=O)(NC(=O)CCC[C@@H](N)C([O-])=O)C2=O)C([O-])=O
889	1250	CC1=C(C=C(C(O)=C1)[C@]1(C)CC[C@@H]2C[C@]12C)C1=C(C)C=C(O)C(=C1)[C@]1(C)CC[C@@H]2C[C@]12C
890	357	C[C@H](CCC=C(C)C)[C@@H]1CC\C(C)=C/C\C=C(C=O)/[C@H]1C=O
891	1550	C[C@]1(Br)CC[C@]2(C[C@@H]1Cl)C(=C)CC[C@@H](Br)C2(C)C
892	204	CC(=C)[C@@H](O)CCC(=C)[C@@]1(O)CC[C@](C)(Br)[C@@H](Cl)C1
893	300	C[C@@H]1C[C@@]2(C)C[C@H]3C[C@@]2(CC[C@H]3C)[C@]1(C)O
894	1396	CC1=CC=C(Br)C(C)(C)[C@]11CC[C@](C)(Cl)[C@@H](Br)C1

895	1613	NCC[C@H](O)C(=O)N[C@@H]1C[C@@H](N)[C@@H](O[C@H]2O[C@@H](CN)[C@@H](O)[C@@H](O)[C@@H]2N)[C@H](O[C@@H]2O[C@H](CO)[C@H](O)[C@H]2O)[C@H]1O
896	1447	COCC1=C(CC2=CC(Br)=C(O)C(CC3=C(CC4=CC(Br)=C(O)C=C4)C(O)=C(O)C(Br)=C3)=C2)C(O)=C(O)C(Br)=C1
897	487	OC1=CC(C[C@@H](C([O-])=O)C2=CC=CC=C2)=C(Br)C(Br)=C1O
898	282	C[C@]1(CCCCCCCCCCCCC2=CC=CC=C2)C[C@]2(C)OC(=O)C[C@@H]2O1
899	77	CC(C)=CCC\C(C)=C\CC\C(C)=C\CCC1=CCC2=C(OC1)C=CC(=C2)C([O-])=O
900	1150	CC\C=C/C\C=C/C\C=C/C\C=C\C#C
901	1530	CC1=C(C)[C@@]2(CC[C@](C)(Cl)[C@@H](Br)C2)C(C)=CC1
902	237	CCC[C@@H](OC(C)=O)C1=C(Br)[C@](OC)(OC1=O)C(Br)Br
903	230	CN1C(Br)=C(Br)C2=C(Br)C=C(Br)C=C12
904	1406	C[C@@H]1CC[C@]2(CC1=O)C(=C)CC[C@H](Br)C2(C)C
905	1161	NC1=[N+]2C[C@@H](Cl)C[C@]2(O)C=C1
906	1027	C[C@H]1CC=C(C2=C(O)C=C(C)C(Br)=C2)C1(C)C
907	96	C[C@](Br)(CCl)[C@H]1C[C@H](O)[C@](C)(O1)C(\Cl)=C/Br
908	954	CC(C)=CC[C@@H](O)C(\C)=C\CCC(=C)[C@H](O)CC\C(C)=C\CO
909	623	CC1=C[C@H](O)[C@H](Br)C(C)(C)[C@]11CC[C@@](C)(Cl)[C@@H](Br)C1
910	1595	CCN(C)[C@@H]1[C@@H]2[C@@H](O)[C@@H]3C(=C(O)[C@]2(O)C(=O)C(C(N)=O)=C1O)C(=O)C1=C(O)C=CC=C1[C@@]3(C)O
911	833	CC[C@H]1[C@@H](OC1=O)C1=C(Br)\C(OC1=O)=C\Br
912	746	CC1=CC[C@]2(CC1)C(=C)C[C@@H](O)[C@H](Br)C2(C)C
913	1192	C[C@]1(O)OC(=O)C=C1[C@H]1CC[C@](C)(Cl)[C@@H](Br)C1
914	1441	CC1=CC(=O)CC(C)(C)[C@]11CC[C@](C)(Cl)[C@@H](Br)C1
915	1315	CCCCCC[C@@H]1C(=O)N[C@]([C@@H](O)[C@H]2CCCC=C2)(C(=O)SC[C@H](NC(C)=O)C(=O)OC)[C@]1(C)O
916	1701	C[C@H]1[C@]2(C)CC[C@@]1(C)C1=CC=C(C)C=C1O2
917	185	C[C@H]1CC(=O)C(C)=C2CCC(C)=CC[C@]12C
918	1500	CC[C@H]1O[C@H](CC(=O)CC\C=C/C#C)[C@H](O)C[C@@H]1Br
919	900	OC1=CC(=CC(Br)=C1O)C([O-])=O
920	1341	CC(C)[C@@]1(O)CC[C@@]2(C)CC=C(C)CCC=C(C)[C@H](C[C@H]12)OC(C)=O
921	49	CC1(C)[C@@H](Br)CC[C@]2(C)O[C@H]3COC(=O)C3=CC[C@@H]12
922	288	OC1=CC(CC2=C(CC3=CC(O)=C(O)C(Br)=C3Br)C=C(O)C(O)=C2Br)=C(Br)C(Br)=C1O
923	704	C[C@@](Br)(CCl)[C@@H]1C[C@H](O)[C@@](C)(O1)C(\Cl)=C\Br
924	641	CC(=O)OC[C@H](Br)[C@]1(C)CC=C2[C@H](C1)[C@H](C[C@H]1[C@@]3(CO)C[C@H]3[C@H](C[C@@]21C)OC(C)=O)OC(C)=O
925	1497	C[C@@H]1CC[C@H]2[C@@H]1[C@@H]1[C@H](C[C@H](O)[C@]21C)C(=C)[C@@H](O)\C=C\[C@@](C)(O)CO
926	430	C[C@@]1(Br)CC[C@]2(C[C@@H]1Cl)C(=C)CC[C@@H](Br)C2(C)C
927	1481	COC1=C(O)C=C(CO)C(CC2=C(Br)C(Br)=C(O)C(O)=C2)=C1O
928	1412	CCCCC1=C(Br)C(OC1=O)=C(Br)Br
929	953	C[C@@H]1CC[C@@H]2[C@]3(C)C[C@@H]3CC(C)(C)C[C@@]12O
930	1519	CC(=O)OCC1=CC=C(C=C1)[C@@H]1CC[C@H](Br)C(C)(C)C1
931	177	CC(C)[C@@H]1CC=C[C@H]2[C@@H]3[C@](C)(O)[C@@H](O)C[C@H](Br)[C@@]3(C)CC[C@]12CBr

932	222	CO[C@@H]1[C@@H](O)[C@@H](O)[C@@H](O[C@H]1CO[C@H]1C[C@@H](O)[C@@H](N)C(C)O1)N1C2=C(C=CC=C2)C2=C1C1=C(C3=CC=CC=C3N1)C1=C2C(=O)N(C)C1=O
933	7	CO[C@@]1(NC(=O)CC2=CC=CS2)[C@@H]2SCC(COC(N)=O)=C(N2C1=O)C([O-])=O
934	959	COC1=CC(C)=C2O[C@@](C)(CC3=C[C@]4(C)CCC[C@]4(C)[C@]4(OC(C)(C)C=C4)O3)CCC2=C1
935	1010	CN(C)CCN1N=NN=C1SCC1=C(N2[C@@H](SC1)[C@H](NC(=O)CC1=CSC(N)=N1)C2=O)C([O-])=O
936	994	CC1(C)[C@@H](Br)[C@@H](O)CC(=C)[C@@]11CC\C(=C\Br)C=C1
937	1616	CC\C=C\C=C\[C@]1(C)OC(=O)C(C(=O)\C=C\C)=C1OC
938	1505	C#[N+]\C=C\C1=CNC2=CC=CC=C12
939	1650	CCC[C@@H]1C[C@H](C[C@H](C)C[C@@H]2C[C@H](C[C@H](CC(=O)O1)O2)OC(=O)\C=C/CCC1=COC(\C=C/CNC(=O)OC)=N1)OC
940	236	COC1=CC2=C(C=C1)C(=O)C=C(N2C)C1=CC2=C(OCO2)C=C1
941	1008	C[C@@H]1CC[C@@H]2C(C)(C)CCC[C@@]2(C)[C@]11CC2=C(O1)C(C=O)=C(O)C(O)=C2
942	1659	CCN1CCN(C(=O)N[C@@H]([C@H](C)O)C(=O)N[C@]2(OC)[C@@H]3SCC(CSC4=NN=NN4C)=C(N3C2=O)C([O-])=O)C(=O)C1=O
943	365	OC[C@@H]1CN2[C@@H](CC2=O)O1
944	1651	CC[C@@H](O[C@@H]1C[C@](C)(O)[C@@H](O[C@@H]2CC[C@@H](OC(=O)[C@H](C)[C@@H](O)C3=CC4=C(C=C3)C(=O)C(C)=CC4=O)[C@H](C)O2)[C@@H](C)O1)[C@@H](C)[C@H](O)[C@H](C)[C@H](O)[C@@H](C)[C@@H](O)[C@H](C)[C@H]1OC(=O)C[C@H](O)[C@@H](C)[C@H](O)C[C@H](O)CCC[C@H](O)CC[C@@H](C)[C@@H](O)[C@@]2(O)C(=O)C[C@@H](C[C@H](O)C[C@@H](O)C[C@@H](O)C[C@@H](O)CC[C@@H](O)[C@@H]1O)OC2=O
945	221	C[C@@H]1CCC[C@]2(O)C(=O)C[C@@H]3[C@@H](C3(C)C)[C@@]12C
946	1323	CC(C)C1=C2[C@H](C[C@@]3(C)[C@@H](O)CCC(=C)[C@]3(O)C[C@]2(C)CC1)OC(C)=O
947	48	CC[C@H](Br)[C@H]1C[C@H]2O\C(=C\C\C=C/C#C)[C@@H](Br)[C@@H]2O1
948	551	C\C(=C\C[C@H]1C(=C)CC=CC1(C)C)[C@H](O)CBr
949	1328	OC1=CC(CC2=C(CN3C=NC4=C3C(=O)NC(=O)N4)C=C(O)C(O)=C2Br)=C(Br)C(Br)=C1O
950	586	CC1=CC=C2C(O[C@@]3(C)[C@@](C)(O)CC[C@@]23C)=C1
951	305	CC(C)=CCC\C(C)=C\CC\C(C)=C\CC[C@]1(C)OC2=C(C)C=C(OC(C)=O)C=C2C=C1
952	1125	CC(=O)O[C@H](CBr)C(\C)=C/C[C@@H]1C(=C)CC[C@H](Br)C1(C)C
953	385	CN([C@@H]1[C@@H](O)[C@@H](O)[C@H](COC(N)=O)O[C@@H]1NC1=N[C@H]2[C@H](N1)C(=O)N(C)C[C@H]2O)C(=O)CN
954	740	C[C@H](CCC=C(C)C)[C@@H]1CC[C@]2(C)[C@H]3CC=C(C)[C@@H]2[C@@H]13
955	830	CCC[C@@H](O)C1=C(Br)\C(OC1=O)=C\Br
956	1211	CCC[C@@H](O)C1=C(Br)\C(OC1=O)=C/I
957	864	COC(=O)C1=CC(Br)=C(O)C(O)=C1
958	1073	CC(C)=CCC\C(C)=C\CC[C@]1(C)[C@@H](Br)CCC2=COC3=CC=C(C=C3C[C@@H]12)C([O-])=O
959	882	CC1(C)[C@@H](Br)C(=O)C=C2C[C@]3(C)CC[C@]12C[C@@H]3Cl
960	308	CC1(C)[C@@H](Br)[C@@H](O)CC2=C[C@]3(C)CC[C@]12C[C@@H]3Cl
961	39	CCC[C@@H](O)C1=C(Br)C(OC1=O)=C(Br)Br
962	72	CO[C@@H](CC[C@H](C)[C@H]1O[C@@]23C[C@H](OC(=O)C[C@@H](OC(=O)C[C@](O)(O2)[C@H](C)CC3(C)C)[C@@H](C)O)[C@@H]1C)C1=C(Br)C=C(Br)C(O)=C1
963	198	CC1=C(Br)C[C@@]2([C@@H](O)C1)[C@]1(C)O[C@@H]1C=C(Br)C2(C)C
964	785	CC(=O)O[C@@H]1C[C@](C)(Cl)[C@@H](Br)C[C@H]1[C@@]1(C)OC(C)(C)[C@H](Br)C[C@@H]1O
965	1053	CC1=CC=C2C(O[C@@]3(CO)[C@@H](Br)CC[C@@]23C)=C1

966	1198	CC1(C)[C@@H](Br)CC[C@]2(C)O[C@H]3CO[C@H](O)C3=CC[C@@H]12
967	659	C[C@@]1(CC=C2[C@@H](C1)[C@H](O)C[C@@H]1[C@@]3(CO)C[C@H]3C(=O)C[C@@]21C)[C@@H](Br)CO
968	1522	CC1=CC[C@@]2(CC1)C(=C)C[C@@H](O)[C@@H](Br)C2(C)C
969	776	CC(C)=CC(=O)C\C(C)=C\CC\C(C)=C\CC\C(C)=C\C=O
970	24	C[C@@H]1CCC=C2C=C[C@@H]3[C@@H](C3(C)C)[C@@]12C
971	1061	CC[C@H](OC)\C=C/[C@H]1O[C@@H](C[C@H]1Br)[C@@H](Cl)C\C=C/C#C
972	309	COC1=CC(C)=C2O[C@](C)(CC\C=C(/C)[C@@H](O)[C@H](O)\C=C(/C)CCC=C(C)C)C=CC2=C1
973	560	CC\C=C/C\C=C/C\C=C/C\C=C/C#C
974	1677	C[C@H]1O[C@H](OC2=CC=C(\C=C\C(=O)NCCCNCCCCN)C=C2)[C@@H](NC(N)=N)[C@@H](O)[C@H]1NC(=O)N[C@H]1OC[C@@H](O[C@@H]2OC[C@@H]3OC(=O)N[C@H]3[C@H]2NC(N)=O)[C@@H](O)[C@@H]1NC(N)=O
975	370	CC1=CC(O)=C2C(=O)OCC3=C2C1=C1O[C@@]2(O)CC(=O)C4=C5C(C(=O)OC[C@]25C1=C3O)=C(O)C=C4C
976	1433	CC(=O)O[C@@H]1C[C@]2(C)[C@H](C[C@@H](O)[C@@H]3C[C@](C)(CC=C23)[C@H]2CO2)[C@@]2(CO)C[C@@H]12
977	1525	BrC1=CC=C2C(NC=C2\C=C2/NC(=O)[C@@H]3CCCN3C2=O)=C1
978	1159	CC(=O)O[C@@H]1C[C@@H]2[C@@]3(C)CCCC(C)(C)[C@@H]3CC[C@@]2(C)[C@@H]2CC=C3[C@H]([C@H](O)OC3=O)[C@@]12C
979	458	CC(C)C[C@@H]1NC(=O)[C@@H](NC(=O)[C@@H](NC(=O)[C@H](C)NC(=O)[C@H](CCCC2=CC=C(O)C=C2)NC(=O)[C@H](O)CO)C(C)C)[C@@H](C)OC(=O)[C@@H](NC(=O)[C@H](CC2=CC(Cl)=C(O)C=C2)N(C)C(=O)[C@H]([C@H](C)O)N2[C@H](O)CC[C@H](NC1=O)C2=O)C(C)C
980	1680	CO[C@@H]1CC(=O)NC2=CC(=O)C=C(CC\C=C(C)\[C@H](O)[C@H](C)[C@@H](C\C=C\C=C\C=C\1)OC(=O)[C@@H](C)NC(=O)CCC(C)C)C2=O
981	1512	CC1(C)[C@H](Br)[C@H](O)CC(=C)[C@@]11CC\C(=C/Br)C=C1
982	1116	C[C@H]1[C@]2(C)CC[C@@](C)(O2)[C@@]11CC[C@@](C)(Cl)[C@H](Br)C1
983	619	CC(C)[C@H]1OC(=O)[C@H](C)NC(=O)C(=C)NC(=O)[C@H](CCCC2=CC=C(O)C=C2)NC1=O
984	1028	CC1(C)C[C@@H](CC[C@H]1Br)C1=CC=C(CO)C=C1
985	13	CC1=C[C@@H](O)[C@@H](Br)C(C)(C)[C@]11CC[C@](C)(Cl)[C@@H](Br)C1
986	714	CO[C@H]1O[C@H](OC)\C2=C\C\C=C(C)/C[C@@H](O)[C@@H]([C@H](C)CCC=C(C)C)[C@@H]12
987	1691	CO[C@H]1\C=C/C=C(C)/C[C@@H](C)[C@H](O)[C@H](C)\C=C(/C)\C=C(OC)/C(=O)O[C@@H]1[C@H](C)[C@H](O)[C@H](C)[C@@]1(O)C[C@@H](O)[C@H](C)[C@@H](O1)C(C)C
988	171	CC1=CC=C(Br)C(C)(C)[C@@]11C[C@H](Br)[C@](C)(Cl)C[C@@H]1O
989	1060	COC(=O)[C@@H]1CC2=C(CN1)C(Br)=C(OC)C(O)=C2
990	89	CC1(C)[C@@H](Br)C(=O)CC2=C[C@]3(C)CC[C@]12C[C@@H]3Cl
991	1330	C[C@@H](CCC=C(C)C)[C@@H]1CC\C(C)=C/C\C=C2\CO[C@@H](O)[C@H]12
992	188	CO[C@@]1(C)OC(=O)C=C1[C@@H]1CC[C@@](C)(Cl)[C@H](Br)C1
993	1594	CO[C@@H]1\C=C/C=C(C)/C[C@@H](C)[C@H](O)[C@H](C)\C=C(/C)\C=C(OC)/C(=O)O[C@H]1[C@@H](C)[C@@H](O)[C@@H](C)C(=O)C[C@@H](OC(=O)\C=C\C(=O)NC1=C(O)CCC1=O)[C@@H](C)[C@H](O)C(C)C
994	1440	COC(=O)[C@@H]1CC2=C(CN1)C(O)=C(OC)C(Br)=C2
995	255	CC1=C(Cl)C[C@]2(CC1)C(=C)C[C@H](O)[C@H](Br)C2(C)C
996	1108	O=C1N[C@H](CC2=CC=CC=C2)C(=O)N[C@@H]1CC1=CC=CC=C1

997	263	CC(=O)OC[C@]12C[C@H]1[C@@H](C[C@]1(C)[C@@H]2C[C@@H](OC(C)=O)[C@@H]2C[C@](C)(CC=C12)[C@@H](Br)CO)OC(C)=O
998	1379	C[C@@]12O[C@@H]1C=C(Br)C(C)(C)[C@]21CC[C@](C)(Cl)[C@@H](Br)C1
999	231	CC[C@@H](C)\C=C(/C)\C=C/[C@@H](O)[C@](C)(O)C(=O)NCC(=O)N[C@H]([C@@H](C)O)C(=O)N[C@@H]([C@H](C)N)C(=O)N[C@@H]([C@@H](C)[C@@H](C)C(N)=O)C(=O)N[C@@H]1[C@H](OC(=O)[C@@H]2CCCCN2C(=O)[C@H](NC(=O)[C@@H](NC(=O)[C@H](C)NC(=O)CNC(=O)[C@@H](COC)NC1=O)[C@@H](C)O)[C@H](OC)C1=CC=C(O)C=C1)C(C)C
1000	81	CC1(C)[C@@H](Br)CC[C@]2(C)OC3=CC(Br)=C(O)C=C3C[C@@H]12
1001	86	C[C@@]1(Cl)C[C@@](C)(\C=C\Cl)[C@H](Cl)C[C@@H]1Cl
1002	733	CC[C@H](N)[C@H](O)[C@@H](C)C(=O)N[C@@H](CC(C)C)[C@@H]1CC2=C(C(O)=CC=C2)C(=O)O1
1003	132	C\C(C=O)=C/C1=C(Br)C(Br)=C(O)C(O)=C1
1004	401	CC1(C)[C@@H](Br)[C@@H](O)CC(=C)[C@@]11CCC(=C)C=C1
1005	844	CCCCC1=C(Br)\C(OC1=O)=C\Br
1006	533	CC(C)=CCC[C@@H](COC(C)=O)[C@@H]1CC\C(C)=C/C\C=C(C=O)/[C@H]1C=O
1007	206	C[C@H]1C(=C)CC[C@@]1(C)C1=C(O)C=C(C)C=C1
1008	902	C\C(C=C)=C\C[C@@H]1C(=C)CC[C@H](Br)C1(C)C
1009	611	CO[C@]1(C)OC(=O)C=C1[C@H]1CC[C@@](C)(Cl)[C@H](Br)C1
1010	408	BrC1=C(Br)C2=CC(Br)=C(Br)C=C2N1
1011	1237	CC(=O)OC[C@]12C[C@H]1[C@@H](C[C@]1(C)[C@@H]2C[C@@H](OC(C)=O)[C@@H]2C[C@](C)(CC=C12)[C@H]1CO1)OC(C)=O
1012	228	NC(=O)C1=CC=[N+](CC2=C(N3[C@H](SC2)[C@@H](NC(=O)[C@H](C2=CC=CC=C2)S([O-])(=O)=O)C3=O)C([O-])=O)C=C1
1013	822	CCC[C@@H](OC(C)=O)C1=C(Br)[C@@](OC)(OC1=O)C(Br)Br
1014	633	CCC[C@@H](O)C1=C(Br)\C(OC1=O)=C/Br
1015	1104	C[C@H]1CC[C@@H]2[C@@]3(C)CC[C@H](O)C(C)(C)[C@@H]3CC[C@@]2(C)[C@]11CC2=C(O1)C(C)=CC(=O)C2=O
1016	193	CC(=O)O[C@H]1C[C@@H]2[C@@]3(C)CCCC(C)(C)[C@@H]3CC[C@@]2(C)[C@@H]2CC=C3[C@H]([C@H](O)OC3=O)[C@@]12C
1017	726	COC(=O)C1=CC(=O)C=C(OC)[C@@]11OC2=C(C1=O)C(O)=CC(C)=C2
1018	999	CN[C@@H]1[C@@H](C[C@H](NC(N)=O)[C@@H](O[C@@H]2O[C@H](CC[C@@H]2N)[C@H](C)N)[C@H]1O)OC
1019	349	COC1=C(OC2=C(Br)C=C(Br)C(Br)=C2O)C=C(Br)C=C1Br
1020	363	CCCCCC[C@@H]1CC(=O)N[C@@H](CC2=CC=CC=C2)C(=O)N[C@@H](C)C(=O)N[C@H](CC(C)C)C(=O)O1
1021	1431	C[C@H]1CO[C@@H](C1)C1=C(Br)\C(OC1=O)=C\Br
1022	239	CC(=O)OCC1=CC=C(C=C1)[C@H]1CC[C@H](Br)C(C)(C)C1
1023	698	CC1=CC=C2OC=CC2=C1C
1024	1326	CC(=O)O[C@H]1CC[C@@]2(C)[C@@H](CC[C@@]3(C)[C@H]2CC[C@@]2(C)OC4=C(C)C=C(OC(C)=O)C=C4C[C@H]32)C1(C)C
1025	1655	COC[C@H]1O[C@@H](O[C@H]2OC[C@@H]3O[C@@]4(O[C@H]3[C@H]2OC(=O)C(C)C)O[C@H](C)[C@@](O)([C@@H](C)O)[C@@H]2OCO[C@H]42)[C@@H](OC)[C@@H](O)[C@@H]1O[C@H]1O[C@H](C)[C@H](OC)[C@H](O[C@@H]2C[C@@]3(C)O[C@]4(C[C@@H](O)[C@@H](O[C@H]5C[C@@H](O)[C@H](OC(=O)C6=C(C)C(Cl)=C(O)C(Cl)=C6OC)[C@@H](C)O5)[C@H](C)O4)O[C@@H]3[C@@H](C)O2)[C@H]1O

1026	1126	COC(=O)[C@H]1CC2=C(CN1)C(O)=C(OC)C(Br)=C2
1027	226	COC1=CC(O)=C2C(=O)[C@H]3[C@@H](O)C[C@H](C)[C@H](O)[C@@]3(C)OC2=C1
1028	249	CC1=CC2=C(C=C1Br)[C@]1(C)CC[C@H](O2)C1(C)C
1029	913	CC1=C(O)C(O)=C(C(O)=C1)C1=C(O)C(O)=C(C)C=C1O
1030	1122	C\C=C\C=C\C1=NC[C@@H](C)C1
1031	1253	CC[C@H](Br)\C=C/[C@H]1O[C@@H](C[C@H]1Br)[C@@H](Cl)C\C=C/C#C
1032	1573	CC(C)[C@H]1N(C)C(=O)[C@]23CS[C@@](CS2)(N(C)C(=O)[C@@H](C)NC(=O)[C@@H](COC1=O)NC(=O)CN1C=NC2=CC=CC=C2C1=O)C(=O)N(C)[C@@H](C(C)C)C(=O)OC[C@@H](NC(=O)CN1C=NC2=CC=CC=C2C1=O)C(=O)N[C@@H](C)C(=O)N3C
1033	1196	C[C@H]1CC=C(C2=CC=C(C)C=C2)C1(C)C
1034	1660	CC1(C)S[C@@H]2[C@H](NC(=O)C34C[C@H]5C[C@H](CC(N)(C5)C3)C4)C(=O)N2[C@H]1C([O-])=O
1035	150	COC1=C(C)C(=O)C(C)=C(O1)[C@H]1C\C(CO1)=C\C(\C)=C\C1=CC=C(C=C1)[N+]([O-])=O
1036	1337	CC1(C)CC(=O)C[C@@H]2C(=O)O[C@H]3C[C@@]12C=C[C@@]3(C)O
1037	1106	OC1=CC=CC2=C1N1[C@@H](C2)C(=O)N2[C@@H](CC3=CC=CC(O)=C23)C1=O
1038	870	C[C@@]12C[C@](C)(CCCCCCCCCCCCC3=CC=C(O)C=C3)OO[C@@H]1CC(=O)O2
1039	572	C[C@H](CCC=C(C)C)[C@H]1[C@@H](O)C\C(C)=C/C\C=C(C=O)/[C@@H]1COC(C)=O
1040	654	CN1C(Br)=C(Br)C2=CC(Br)=CC=C12
1041	509	C[C@]1(Cl)C[C@](C)(\C=C\Cl)[C@H](Cl)C[C@@H]1Cl
1042	908	NC1=C(Br)C(=O)[C@@](O)(C1)C=C
1043	478	C[C@@]1(Cl)C[C@@](C)(Br)[C@@H](C[C@@H]1Br)\C=C\Cl
1044	565	CC(C)[C@@H]1NC(=O)[C@@H]2CCCN2C1=O
1045	1511	COC(=O)[C@]1(CC2=CC(C[C@@H]3OC3(C)C)=C(O)C=C2)OC(=O)C(O)=C1C1=CC=C(O)C=C1
1046	1705	CCCCCCCCCCCCCCCCCC(=O)OC[C@H](CO[C@@H]1O[C@H](CN)[C@H](O)[C@H](O)[C@H]1O)OC(=O)CCCCCCCCCCCCCCCCC
1047	1023	C[C@@H]1C[C@H](OC1=O)C1=C(Br)\C(OC1=O)=C\Br
1048	25	C[C@@H](C[C@H](O)[C@H]1O[C@@H]2CC[C@@]3(CC[C@@H](O3)\C=C\[C@@H](C)[C@@H]3CC(C)=C[C@@]4(O[C@H](C[C@@](C)(O)C([O-])=O)CC[C@H]4O)O3)O[C@H]2[C@H](O)C1=C)[C@H]1O[C@@]2(CCCCO2)CC[C@H]1C
1049	315	C[C@@]1(Cl)C[C@](C)(\C=C\Cl)[C@@H](Br)C[C@H]1Cl
1050	322	CC(=O)O[C@H]1CC[C@@]2(C)[C@@H](CC[C@]3(C)[C@@H]2CC[C@@]2(C)OC4=C(C)C=C(OC(C)=O)C=C4C[C@H]32)C1(C)C
1051	485	CCOCC1=CC(O)=C(O)C(Br)=C1CC1=C(Br)C(Br)=C(O)C(O)=C1
1052	97	CC(C)=C[C@H](O)C\C(C)=C1/CC[C@](C)(Br)[C@@H](Cl)C1
1053	1141	CC1(C)[C@@H](Br)[C@@H](O)CC2=C[C@]3(C)CC[C@]12CC3=O
1054	44	CC1=CC(=O)CC(C)(C)[C@]11CC\C(=C/Br)C=C1
1055	1528	C[C@H](CCC=C(C)C)[C@@H]1CC[C@]2(C)CC=C(C=O)[C@@]1(C=O)[C@H]2OC(C)=O
1056	392	C[C@H](CC[C@H](O)C(C)=C)[C@@H]1CC[C@@]2(C)[C@@H](O)CC=C(C=O)[C@@]12C=O
1057	1095	CC1=COC2=C1C[C@@H]1[C@H](CCC1=C)C(C)=C2
1058	486	CC1(C)[C@@H](Br)CC[C@@]2(C)OC3=CC(Br)=C(O)C=C3C[C@@H]12
1059	562	C[C@H](CCCC[C@H]1OC(=O)[C@H]1CO)C\C(C)=C\C(\C)=C\C([O-])=O
1060	1418	CC(=O)O[C@]1(C[C@@H]2CC[C@]34[C@@H](O)O[C@](C)(CC[C@@H]3Br)[C@@H]24)CC[C@H](Br)C(C)(C)C1
1061	1146	C[C@@]1(O)[C@H](O)C[C@H](Br)[C@@]2(C)CC[C@]3(C[C@H]12)C(=C)CC[C@H](Br)C3(C)C

1062	1541	COC(=O)[C@H]1C2=CC3=C(C(O)=C2[C@H](C[C@]1(C)O)O[C@@H]1C[C@@H]([C@H](O)[C@@H](C)O1)N(C)C)C(=O)C1=C(O)C=CC=C1C3=O
1063	584	C[C@H](CCC=C(C)C)[C@H]1CC\C(C)=C/C\C=C2\CO[C@@H](O)[C@H]12
1064	11	C[C@H]1C=C[C@@H](Br)C(C)(C)[C@@H]1CC[C@@](C)(O)[C@H](O)CBr
1065	1639	CC[C@@H](O)[C@@H](C)[C@@H](O)[C@@H](C)[C@H](O)[C@@H](C)[C@H](O)[C@@H](C)[C@H]1OC(=O)C[C@H](O)[C@@H](C)[C@@H](O)C[C@@H](O)CCC[C@@H](O)CC[C@@H](C)[C@H](O)[C@@]2(O)C(=O)C[C@@H](C[C@H](O)C[C@H](O)C[C@H](O)C[C@H](O)CCC[C@H](O)[C@@H]1O)OC2=O
1066	1062	BrC1=CC=C2C(NC=C2[C@H]2CNC(=O)C(=N2)C2=CNC3=CC(Br)=CC=C23)=C1
1067	1128	C[C@@]1(O)CC[C@]2(C[C@@H]12)[C@H]1CC[C@H](Br)C(C)(C)C1
1068	1026	C[C@H]1[C@@H]2[C@H](OC(C)=O)C(=O)[C@@]3(C)[C@@H](CC[C@H]4\C([C@H](C[C@]34C)OC(C)=O)=C(/CCCC(C)(C)O)C([O-])=O)[C@@]2(C)C=CC1=O
1069	119	C[C@@]1(Cl)C[C@](C)(\C=C\Cl)[C@H](Cl)C[C@H]1Cl
1070	372	[O-][N+](=O)C1=C(C=CC=C1Cl)C1=CNC(Cl)=C1Cl
1071	1392	OC1=C(O)C(Br)=CC(C=C)=C1
1072	88	COC1=C(O)C=C(CO)C=C1Br
1073	402	C[C@H]1CC[C@@]2(C)[C@@H](CCCC2=C)[C@]1(C)CC1=C(O)C(=O)C=C(NCCC([O-])=O)C1=O
1074	701	CC(C)[C@@H]1N(C)C2=CC=CC3=C2[C@@](O)(C[C@H](CO)NC1=O)C(=O)N3
1075	752	CC1(C)S[C@@H]2[C@@H](NC(=O)[C@@H](NC(=O)N3CCNC3=O)C3=CC=CC=C3)C(=O)N2[C@H]1C([O-])=O
1076	806	C[C@H]([C@@H]1O[C@@]2(CCC[C@@H](CC3=NC4=C(O3)C=CC(O)=C4C([O-])=O)O2)CC[C@H]1C)C(=O)C1=CC=CN1
1077	501	CC[C@@H](C)[C@H](N(C)C(=O)[C@H](CC(N)=O)N(C)C(=O)[C@H](C(C)C)N(C)C(=O)[C@@H](CC1=CC=CC=C1)NC(=O)COC)C(=O)N(C)[C@@H](C(C)C)C(=O)N(C)[C@@H](CC(C)C)C(=O)N[C@@H](CC(N)=O)C(=O)N[C@H](CC1=CC=CC=C1)C(=O)N1CCC[C@H]1C(=O)N1CCC[C@H]1C(=O)N[C@@H](CCCNC(N)=N)C([O-])=O
1078	291	COCC1=CC(O)=C(O)C(Br)=C1Br
1079	989	C[C@@](O)(CC[C@H]1C(=C)CC[C@@H](Br)C1(C)C)C=C
1080	803	CC1(C)O[C@](C)([C@@H]2CC[C@](C)(Cl)[C@@H](Br)C2)C(=O)C=C1
1081	75	CC1=CC=C2C(O[C@@]3(C)[C@@H](Br)CC[C@@]23C)=C1
1082	19	CC\C=C\C\C=C/C[C@H](OC(C)=O)[C@H](C\C=C\C#C)OC(C)=O
1083	396	CC(=O)O[C@H]1C[C@]2(OC1(C)C)OC(C\C(C)=C\CC1=C(OC(C)=O)C(C)=CC(OC(C)=O)=C1)=C[C@@]1(C)CCC[C@@]21C
1084	82	OC1=CC(C=O)=C(Br)C(Br)=C1O
1085	1083	CC1(C)[C@@H](Cl)C[C@H](Br)\C(=C/CCl)[C@H]1Cl
1086	1558	CC(C)C1=C2[C@H](O)C[C@]3(COC(C)=O)[C@H](O)CCC(=C)[C@@]3(O)C[C@@]2(C)CC1=O
1087	196	OCCC1=CC(O)=C(O)C(Br)=C1Br
1088	1356	OC\C=C1\O[C@@H]2CC(=O)N2[C@@H]1C([O-])=O
1089	622	C[C@@]1(O)CC[C@@]2(C[C@H]1Br)C(=C)CC[C@H](Br)C2(C)C
1090	1434	CO[C@H]1CC(=O)[C@H]2C[C@@]34SS[C@]5(C[C@H]6[C@H]([C@@H](O)[C@H](CC6=O)OC)N5C3=O)C(=O)N4[C@H]2[C@H]1O
1091	1297	C\C(=C/Br)\C=C\[C@@H](Cl)[C@](C)(Cl)C=C
1092	1183	CC1=C(Cl)C[C@]2(CC1)C(C)=C[C@H](O)[C@H](Br)C2(C)C
1093	187	CC(C)=CC[C@H](O)C(\C)=C\CC\C(C)=C\CC\C(C)=C\C([O-])=O

1094	320	C[C@@]1(CC(=C)[C@H](Cl)C[C@H]1Cl)\C=C\Cl
1095	634	C[C@@]1(Cl)CC[C@@]2(C[C@H]1Br)C(=C)C(=O)C=CC2(C)C
1096	1445	CCCCCCCCCCCCCCCC(=O)O[C@@H]1CC(=C)[C@H]2C[C@@H](O)[C@@H]3C[C@](C)(CC=C3[C@]2(C)C[C@H]1OC(C)=O)[C@H](Br)CO
1097	1093	CC[C@H](C)[C@@H](N)C(=O)N[C@@H]([C@@H](C)CC)C(=O)N[C@@H](COP([O-])([O-])=O)C(=O)N[C@H](CCC(N)=O)C(=O)N[C@H](CCC([O-])=O)C([O-])=O
1098	826	CCC[C@@H](O)C1=C(Br)\C(OC1=O)=C/Cl
1099	1078	CC(C)C[C@@H]1NC(=O)[C@@H](NC(=O)[C@@H](NC(=O)[C@@H]2CCCN2C(C)=O)[C@@H](C)O)[C@@H](C)OC(=O)[C@@H](NC(=O)[C@H](CC2=CC=C(O)C=C2)N(C)C(=O)[C@H](CC2=CC=CC=C2)N2[C@H](O)CC[C@H](NC1=O)C2=O)C(C)C
1100	879	C[C@@H]1CC[C@H]2C(C)(C)CCC[C@]2(C)C2=C1OC1=C(C=O)C(O)=C(O)C=C21
1101	1699	CC[C@@H]1OC(=O)C[C@@H](OC(C)=O)[C@H](C)[C@@H](O[C@@H]2O[C@@H](C)[C@@H](O[C@@H]3C[C@](C)(O)[C@@H](OC(=O)CC(C)C)[C@@H](C)O3)[C@H]([C@H]2O)N(C)C)[C@@H](CC=O)C[C@@H](C)C(=O)\C=C/C(/C)=C/[C@H]1CO
1102	106	CC(=C)[C@H](Br)C[C@@H](Br)C(\C)=C\C=O
1103	1587	CN[C@H]1[C@@H](C[C@@H](N)[C@H](O[C@H]2O[C@@H](CC[C@@H]2NC(N)=O)[C@H](C)N)[C@H]1O)OC
1104	1630	C[C@](O)(CO)[C@H](O)[C@@]12NC(=O)[C@@](O)(NC1=O)C(=C)CCO2
1105	519	COC(=O)C1=CC=C(C=C1)[N+](\[O-])=N/C#N
1106	447	CC[C@@H]1CO[C@H]1C1=C(Br)\C(OC1=O)=C\Br
1107	215	OCC1=C(Br)C(O)=C(O)C(Br)=C1
1108	721	CC(C)[C@]1(O)[C@@H](O)C[C@]2(C)CC=C(C)CC[C@@H]12
1109	1005	C[C@@]12O[C@@H]1C=C(Br)C(C)(C)[C@]21CC[C@@](C)(Cl)[C@@H](Br)C1
1110	1536	C[C@H](CCC=C(C)C)[C@@H]1CC=C(C)[C@H]2CC=C(C)[C@@H]2[C@@H]1O
1111	453	CC(=O)O[C@@](C)(CC[C@@H]1C(C)=CC[C@H](Br)C1(C)C)C=C
1112	681	COC1=CC(C=O)=C(Br)C(Br)=C1OC
1113	241	CCC[C@@H](OC(C)=O)C1=C(Br)[C@@](Cl)(OC)OC1=O
1114	1055	COCC1=C(CC2=CC(Br)=C(O)C=C2)C(O)=C(O)C(Br)=C1
1115	1142	CN[C@H]1C(O)[C@@H](NC)[C@H](O)C(O[C@@H]2C[C@@H](O)C[C@H](C)O2)[C@@H]1O
1116	647	CC[C@@H](Cl)\C=C/[C@H]1O[C@@H](C[C@@H]1OC(C)=O)[C@@H](Cl)C\C=C/C#C
1117	958	C\C=C\C\C=C\CCC(=O)[C@H]1O[C@H]1C(N)=O
1118	1637	CC[C@@H](C)[C@H]1NC(=O)[C@H](NC(=O)C2=CC=CC3=C2N=C2C(O3)=C(C)C(=O)C(N)=C2C(=O)N[C@H]2[C@@H](C)OC(=O)[C@@H](C(C)C)N(C)C(=O)CN(C)C(=O)[C@H]3CCCN3C(=O)[C@@H](NC2=O)C(C)C)[C@@H](C)OC(=O)[C@H](C(C)C)N(C)C(=O)CN(C)C(=O)[C@@H]2CCCN2C1=O
1119	887	CC(C)=CCC\C(C)=C\CC\C(C)=C\CC\C(C)=C\CC1=CC(OC(C)=O)=CC(C)=C1OC(C)=O
1120	1307	CC(=O)O[C@@H]1C[C@@](C)(Cl)[C@H](Br)C[C@@]11C(C)(C)[C@@H]2CC[C@@]1(C)O2
1121	115	NC[C@H](O)C[C@@H]1NC(=O)[C@@H](N)CC2=CC(=CC=C2O)C2=CC=C(O)C(=C2)[C@@H](O)[C@@H](NC1=O)C([O-])=O
1122	407	CC(C)=CCC\C(C)=C\C\C=C(/C)[C@H](O)CC1=CNC(=C1)[N+]([O-])=O
1123	729	CC(C)C1=C2[C@H](C[C@@]3(C)CCCC(=C)[C@]3(O)C[C@]2(C)CC1)OC(C)=O
1124	1502	COC1=CC(O)=C2OC(C)=CC2=C1Cl
1125	1355	COC1=CC(C\C=C(/C)CC2=C[C@]3(C)CCC[C@]3(C)[C@]3(OC(C)(C)C=C3)O2)=C(OC)C(C)=C1
1126	1432	CC1(C)C[C@H](CC[C@@H]1Br)[C@@]1(Cl)CC[C@@](O)(CO)[C@@H]2O[C@H]12

1127	628	C[C@@H]1CCC=C2C(=O)C[C@@H]3[C@@H](C3(C)C)[C@@]12C
1128	649	CCC[C@@H](OC(C)=O)C1=C(Br)\C(OC1=O)=C\I
1129	650	CC(=O)OC[C@H](Br)[C@@]1(C)CC=C2[C@@H](C1)[C@H](O)C[C@@H]1[C@@]3(C)C[C@H]3[C@@H](C[C@@]21C)OC(C)=O
1130	894	CC(=C)[C@H](Br)C[C@@H](Br)C(\C)=C\C(Cl)Cl
1131	1160	C[C@H](CCC=C(C)C)[C@@H]1[C@@H](C[C@@H](C)[C@@H]2C[C@@H]2C2=C1C(=O)OC2)OC(C)=O
1132	123	CCCCCCC[C@@]1(O)C(=O)NC2=C(C=CC=C2)C1=O
1133	905	C[C@@](Cl)(\C=C\Cl)[C@@H](Cl)\C=C\[C@@](Cl)(CCl)CBr
1134	1057	C[C@]1(Cl)CC[C@]2(C[C@@H]1Br)C(=C)C[C@@H](O)[C@@H](Br)C2(C)C
1135	1446	CCCCCCCCCCCCCCC\C(O)=C1/C(=O)[C@@H](C)N(C)C1=O
1136	435	CC1=CC(C([O-])=O)=C(C(=O)C2=C(O)C=CC=C2O)C(O)=C1
1137	217	COC1=CC=CC2=C1C(=O)C1=C(O)C3=C(O[C@]4(C)CCC[C@H]3O4)C=C1O2
1138	268	CCCCCC\C=C/CCCCCCCCCCCCC#C[C@@H](OS([O-])(=O)=O)C([O-])=O
1139	67	COC1=C(Br)C2=C(C[C@H](NC2)C([O-])=O)C=C1O
1140	1147	C[C@@H](CO)NC(=O)[C@H]1CN(C)[C@@H]2CC3=CNC4=CC=CC(=C34)C2=C1
1141	1590	C[C@H]1CC[C@H]2C(C)(C)CCC[C@]2(C)\C1=C\C1=CC(O)=C(O)C(\C=N/CCS([O-])(=O)=O)=C1OS([O-])(=O)=O
1142	1046	CC(=O)O[C@@H]1C[C@]2(C)[C@H](C[C@@H](OC(C)=O)[C@@H]3C[C@](C)(CC=C23)[C@H]2CO2)[C@@]2(CO)C[C@@H]12
1143	1115	CO[C@@]12OC3=CC(CO)=C(Br)C(Br)=C3O[C@@]1(O)CC(CO)=CC2=O
1144	856	CC(C)=CCC[C@@]1(C)[C@H](Br)CC[C@@]2(C)[C@@H](CC3=C(O)C=CC(=C3)C([O-])=O)C(=C)CC[C@H]12
1145	890	CC[C@H](O)[C@H](C)\C=C\C[C@H]1CO[C@@H]([C@H](O)C(\C)=C\C(=O)OCCCCCCCC(=O)NC2=C3SSC=C3NC2=O)[C@H](O)[C@@H]1O
1146	1182	C[C@H]1CCC=C(C)[C@@H](O)\C=C\[C@@](C)(O)C=CC(=O)O[C@H]1C
1147	1321	COC1=C2C(O[C@H]3OC=C[C@@]23O)=CC2=C1C(=O)C1=C(O)C=CC=C1O2
1148	627	CC1=C(Cl)C[C@@]2(CC1)C(=C)C[C@@H](O)[C@@H](Br)C2(C)C
1149	517	OC1=CC(OC2=C3OC4=C(O)C=C(O)C(=C4OC3=C(O)C=C2O)C2=C3OC4=C(O)C=C(O)C(OC5=CC(O)=CC(O)=C5)=C4OC3=C(O)C=C2O)=CC(O)=C1
1150	1377	NCCCC[C@@H](NC(=O)[C@@H](CO)NC(=O)[C@H](N)CO)[C@H](O)CC(=O)OCC1=C(N2[C@@H](SC1)[C@](NC=O)(NC(=O)CCC[C@H](N)C([O-])=O)C2=O)C([O-])=O
1151	415	COC1=CC(C=O)=C(Br)C(Br)=C1O
1152	1151	COC1=CC=CC2=[N+]([O-])C3=C(O)C=CC=C3[N+]([O-])=C12
1153	1199	C[C@H](N)C(=O)N[C@@H](C)C(=O)N[C@H](C)C(=O)N[C@H](CCCNC(N)=N)[C@H](O)CC(=O)OCC1=C(N2[C@@H](SC1)[C@](NC=O)(NC(=O)CCC[C@H](N)C([O-])=O)C2=O)C([O-])=O
1154	1465	OC1=CC(CC2=C(CCOS([O-])(=O)=O)C=C(O)C(O)=C2Br)=C(Br)C(Br)=C1O
1155	573	CC(C)[C@H](N)C(=O)N[C@H]1CC[C@@]1(O)CC([O-])=O
1156	600	BrC1=C(Br)C2=CC=C(Br)C=C2N1
1157	1042	CC(=O)OC[C@]12C[C@H]1[C@@H](C[C@]1(C)[C@@H]2C[C@@H](OC(C)=O)[C@@H]2C[C@](C)(CC=C12)[C@@H](O)CO)OC(C)=O
1158	778	C[C@H]1CC[C@@]2(C)C3=CC=C(C)C=C3O[C@@]12C
1159	724	CCCCCC[C@@H]1C(=O)N[C@@]2([C@H](O)[C@H]3CCCC=C3)C(=O)O[C@@]12C
1160	1022	CC(C)=C[C@H]1CC(=C)[C@]2(CC[C@](C)(Cl)[C@@H](Br)C2)O1

1161	54	C[C@H]1C[C@H](O)[C@@H]2C(=O)C3=C(O)C=CC=C3O[C@]2(C)[C@H]1O
1162	760	OC1=CC=CC2=NC3=CC=CC=C3N=C12
1163	301	CC(=O)O[C@@H]1C[C@@H]2[C@@]3(C)CCCC(C)(C)[C@@H]3CC[C@@]2(C)[C@@H]2CC=C(C[C@@]12C)C=O
1164	679	C[C@]12C[C@](C)(CCCCCCCCCCCCC3=CC=C(O)C=C3)OO[C@H]1CC(=O)O2
1165	725	CO[C@H]1O[C@H](O)[C@@H]2[C@H]([C@@H](C)CCC=C(C)C)[C@H](C\C(C)=C/C\C=C1/2)OC(C)=O
1166	286	OC1=CC(O)=C(OC2=CC(O)=C3OC4=C(OC5=CC(O)=CC(O)=C5)C(O)=CC(O)=C4OC3=C2)C(O)=C1
1167	427	CO[C@H]1[C@H](O)[C@H](O)[C@H](O[C@@H]1CO[C@H]1C[C@H](O)[C@@H](N)CO1)N1C2=C(C3=C1C(Cl)=CC=C3)C1=C(C(=O)N(C)C1=O)C1=C2NC2=CC=CC=C12
1168	504	C[C@@](Cl)(C=C)[C@@H](Cl)\C=C\C(=C\Cl)\C(Cl)Cl
1169	264	CC(C)C[C@@H]1NC(=O)[C@H](CC2=CC=CC=C2)NC(=O)[C@H](CC2=CC=CC=C2)N(C)C(=O)[C@H](CC2=CC=CC=C2)NC(=O)C(C)(C)NC(=O)[C@H](CC(C)C)N(C)C(=O)[C@@H]2[C@@H](C)CCN2C1=O
1170	1157	C\C(CC[C@@H](O)C(C)(C)O)=C\CC1=C(O)C(C=O)=C(C)C(Cl)=C1O
1171	1706	C[C@H]1O[C@@H](O[C@@H]2[C@H](O)[C@@H](O)[C@H](OC(N)=O)[C@@H](O)[C@@H]2NC(N)=N)[C@@H](O[C@]2(C)O[C@H](CO)[C@H](O)[C@@H](O)[C@@H]2N)[C@@]1(O)CO
1172	386	CC1=CC[C@]2(CC1)C(=C)C[C@H](O)[C@H](Br)C2(C)C
1173	817	CO[C@H]1[C@H](O)[C@H](O)[C@H](O[C@@H]1CO[C@@H]1C[C@H](O)[C@H](N)C(C)O1)N1C2=C(C3=C1C(Cl)=CC=C3)C1=C(C(=O)N(C)C1=O)C1=C2NC2=CC=CC=C12
1174	662	C[C@H](CC1=C(Br)C(Br)=C(O)C(O)=C1)C=O
1175	351	CC(=O)OCC1=CC=C(CC1)[C@H]1CC[C@H](Br)C(C)(C)C1
1176	1105	C\C(\C=C\[C@H](Cl)[C@](C)(Cl)CBr)=C/C=O
1177	62	CO[C@@H](CC[C@H](C)[C@H]1O[C@@]23C[C@H](OC(=O)C[C@@H](OC(=O)C[C@](O)(O2)[C@H](C)CC3(C)C)[C@@H](C)O)[C@@H]1C)C1=CC(O)=CC=C1
1178	1405	CCCC\C=C1/C[C@@H]1C1=C(C)C(=O)C2=C(N1)C=CC=C2
1179	987	CCOC(=O)C[C@@H](O)C[C@H](O)CC[C@@]1(O)[C@H](C)C=CC2=C[C@@H](C)CC[C@H]12
1180	862	CSC1=NC=C(C[C@@H](C([O-])=O)[N+](C)(C)C)N1
1181	100	C\C(CCl)=C/C[C@H](Br)C(\C)=C\C=O
1182	29	COC1=C(OC)C(=NC(CO)=C1)C1=NC=CC=C1
1183	41	C[C@]12CC[C@H](Br)C(C)(C)[C@H]1C[C@@H](O2)C1=C(O)C(=O)OC1
1184	1568	CCCCCC\C=C/CC(=O)N[C@@H](CO)C(=O)N[C@@H](C(C)C)C(=O)N[C@@H](CO)[C@H](O)CC(=O)N[C@@H](C(C)C)C(=O)N[C@@H]1COC(=O)[C@H](CC2=CC=C(O)C=C2)N(C)C(=O)[C@@H](NC(=O)\C=C/1)C(C)C
1185	1424	CC(=O)OC[C@H](Br)[C@@]1(C)CC=C2[C@H](C1)[C@H](C[C@@H]1[C@]3(O)CC[C@@H]3[C@@H](C[C@]21C)OC(C)=O)OC(C)=O
1186	1293	C[C@@](Cl)(C=C)[C@H](Cl)\C=C\C(=C\Cl)\C=O
1187	1524	C[C@H](CCC=C(C)C)[C@H]1[C@@H](C\C(C)=C/C\C=C(C=O)/[C@@H]1C=O)OC(C)=O
1188	1164	COC1=CC(C\C=C(/C)CC2=C[C@]3(C)CCC[C@]3(C)[C@]3(OC(C)(C)C=C3)O2)=C(O)C(C)=C1
1189	1346	OC1=CC=CC2=[N+]([O-])C3=C(O)C=CC=C3[N+]([O-])=C12
1190	1516	OC[C@@H]1NC[C@H](O)[C@H]1O
1191	307	C[C@@]1(C[C@](Cl)(CBr)[C@@H](Cl)C[C@H]1Cl)\C=C\Cl
1192	743	COC1=C(Br)C=C(C[C@H](N)C([O-])=O)C=C1O
1193	1100	CC(=O)OCC1=C(N2[C@@H](SC1)[C@H](NC(=O)CSC1=CC=NC=C1)C2=O)C([O-])=O
1194	1393	CC(=O)O[C@@H]1C[C@@](C)(Cl)[C@H](Br)C[C@H]1[C@]1(C)CC[C@@H](Br)C(C)(C)O1

1195	1282	C[C@H]1CC[C@@H]2[C@@]3(C)CC[C@H](OC(C)=O)C(C)(C)[C@@H]3CC[C@@]2(C)[C@]11CC2=C(O1)C(C)=CC(OC(C)=O)=C2OC(C)=O
1196	38	O[C@H]1CC(=O)C2=C3[C@@H]1[C@]1(O)C=CC(=O)C4=C(O)C=CC(C3=CC=C2O)=C14
1197	1694	CO[C@H]1\C=C/C=C(C)/C[C@@H](C)[C@H](OC(C)=O)[C@H](C)\C=C(/C)\C=C(OC)/C(=O)O[C@@H]1[C@H](C)[C@H](O)[C@H](C)[C@@]1(O)C[C@@H](OC(C)=O)[C@@H](C)[C@@H](O1)C(C)C
1198	1632	CC(C)=CCC[C@@](C)(O)[C@@H]1CC[C@](C)(O1)[C@H]1CC[C@H](O1)[C@@]1(C)CC[C@@H](O1)[C@@](C)(O)CCC=C(C)C
1199	64	CC[C@@H](O)\C=C/[C@H]1O[C@@H](C[C@@H]1O)[C@@H](Cl)C\C=C/C#C
1200	303	C\C(=C\C[C@@H](O)[C@](C)(Br)CCl)\C(\Cl)=C\Br
1201	256	OC1=CC(O)=C(OC2=CC(O)=CC(O)=C2OC2=CC(O)=CC(O)=C2C2=C(O)C=C(O)C=C2O)C(O)=C1
1202	1136	CC(C)C[C@@H](NC(=O)[C@H](O)[C@H](N)CC1=CC=CC=C1)C([O-])=O
1203	808	CC(=O)O[C@H]1C[C@@H](Br)C(C)(C)O[C@]1(C)[C@H]1CC[C@@](C)(Cl)[C@H](Br)C1
1204	832	OC1=CC=C2C3=C4C(C(=O)CC[C@@]4(O)[C@@H]4[C@H]5O[C@H]5C(=O)C1=C24)=C(O)C=C3
1205	442	COC1=CC2=C(C=C1O)C1=CC3=C(C=NC=C3C(=O)N1CC2)[C@@H](C)O
1206	1217	CC1(C)C[C@H](CC[C@@H]1Br)[C@@]1(O)CCC(=O)C=C1
1207	700	CC(=C)[C@](Cl)(CBr)CC[C@@H](Br)C(C)(C)Cl
1208	52	CS(=O)C1=C(C2=C(Br)C=C(Br)C=C2N1)S(C)=O
1209	1385	CCCC[C@H](C)[C@@H]1CC(=O)NCC(=O)N[C@@H](C(C)C)C(=O)N[C@@H](CC(C)C)C(=O)N[C@@H](C)C(=O)N[C@@H](CC2=CC=CC=C2)C(=O)O1
1210	510	C[C@H]1CC[C@@H]2[C@@]3(C)CC[C@H](OC(C)=O)C(C)(C)[C@@H]3CC[C@@]2(C)[C@]11CC2=C(O1)C(C)=CC(OC(C)=O)=C2
1211	615	CC(C)=C[C@@H]1CC(=C)[C@]2(CC[C@](C)(Cl)[C@@H](Br)C2)O1
1212	771	CC\C=C\C=C\[C@]1(C)OC(OC)=C(C(C)=O)C1=O
1213	1246	O[C@@H]1[C@H]2[C@H](C[C@]34SS[C@]5(C[C@H]6[C@H]([C@@H](O)[C@H](S)CC6=O)N5C3=O)C(=O)N24)C(=O)C[C@H]1S
1214	814	CC(C)CCC[C@@H](C)[C@H]1CC[C@@H]2[C@]1(C)CC[C@@H]1[C@]34CO[C@]21C[C@@H](O)[C@@]3(O)C[C@H](CC4)OS([O-])(=O)=O
1215	1336	CCCC[C@@H](C)[C@H]1CC(=O)N[C@H](C(C)C)C(=O)N[C@H](CC2=CC=CC=C2)C(=O)N[C@H]([C@H](C)CC)C(=O)O1
1216	809	COC1=C(Br)C=C(C\C(=N/O)C(=O)NCCC2=CNC=N2)C=C1
1217	1489	CC(=O)OC[C@H](Cl)C1=CC(C)(C)C=CC1=O
1218	1148	CC(C)C1=C2CC[C@@]3(C)[C@H](CCC(=C)[C@]3(O)C[C@]2(C)CC1)OC(C)=O
1219	1627	CN[C@H]1[C@@H](O)C[C@@H](N)[C@H](O[C@@H]2O[C@H](CO)[C@H](O)[C@H](O)[C@@H]2N)[C@H]1O
1220	105	CN(C)C1CSSC1
1221	1043	C[C@@H](O)[C@H](C)\C=C\C[C@]1(O)CO[C@H](C\C(C)=C\C(=O)OCCCCCCCCC(=O)NC2=C3SSC=C3NC2=O)C[C@@H]1O
1222	521	C[C@@H]1CC[C@H]2[C@@H]1[C@@H]1[C@H](C[C@H](O)[C@]21C)C(=C)[C@@H](O)[C@H](O)\C=C(/C)COC(C)=O
1223	1276	C[C@]12C[C@](C)(CCCCCCCCCCCCC3=CC=CC=C3)OO[C@H]1CC(=O)O2
1224	1592	NCC[C@H](O)CN[C@@H]1C[C@H](N)[C@H](O[C@H]2O[C@@H](CN)[C@@H](O)[C@@H](O)[C@@H]2O)[C@@H](O)[C@@H]1O[C@@H]1O[C@H](CO)[C@@H](O)[C@H](N)[C@H]1O
1225	285	C[C@]1(Cl)CC[C@@]2(C[C@@H]1Br)C(=C)C[C@H](O)[C@H](Br)C2(C)C

1226	1244	OC1=CC(CCOS([O-])(=O)=O)=CC(Br)=C1O
1227	536	C[C@H](CCC=C(C)C)[C@@H]1CC[C@@]2(C)[C@H](O)CC=C(C=O)[C@@]12C=O
1228	1255	CCCCC\C=C/C[C@@H](O)[C@@H](O[C@H](C\C=C/CCC)[C@H](O)\C=C\[C@H](O)CCCCC([O-])=O)\C=C\[C@H](C\C=C/CCCC([O-])=O)OC[C@H]1O[C@@H](OC[C@@H](O)CO)[C@@H](O)[C@@H](O)[C@H]1O
1229	961	COC1=CC=C2OC3=CC=CC(O)=C3C(=O)C2=C1C([O-])=O
1230	465	CC(=O)O[C@@H]1C[C@]2(C)[C@H](C[C@@H](O)[C@@H]3C[C@](C)(CC=C23)[C@@H](Br)CO)[C@@]2(C)C[C@@H]12
1231	1185	OCCC1=CC=C(O)C=C1
1232	960	CO[C@@H]1[C@H](C)[C@@]2(C)CC=C(C)CCC2=C(C)C1=O
1233	1264	CC(C)[C@H]1CC(C)(C)C[C@@]23O[C@@]12C[C@@H](Cl)[C@]3(C)O
1234	801	CO[C@H](C)[C@H](NC(=O)[C@H]1CCCN1C)[C@H]1O[C@@H](SCCOC(=O)C(C)C)[C@H](O)[C@@H](O)[C@@H]1O
1235	599	CCCCCC1=CC=CC(O)=C1C1=N[C@H](CS1)[C@@H]1SC[C@@H]([C@@H](O)C(C)(C)C([O-])=O)N1C
1236	1529	C\C=C\C=C\C=C/C1=C(C=O)C(O)=C(CC=C(C)C)C=C1O
1237	1252	CCOCC1=CC(O)=C(O)C(Br)=C1Br
1238	804	CC[N+](C)(C)C1=CC(O)=CC=C1
1239	437	CN1N=NN=C1SCC1=C(N2[C@@H](SC1)[C@H](NC(=O)[C@H](NC(=O)C1=C(O)C=C(C)N=C1)C1=CC=C(O)C=C1)C2=O)C([O-])=O
1240	591	C[C@@]12O[C@@H]1C[C@@H]1[C@](C)(CC[C@H](Br)C1(C)C)O\C2=C\Br
1241	266	C[C@@H](CC1=C(Br)C(Br)=C(O)C(O)=C1)C=O
1242	452	CC1(C)C[C@H](CC[C@H]1Br)[C@]12CC[C@]3(CO)O[C@@H]3[C@H]1O2
1243	1340	C[C@H]1CC(=O)[C@H](C)[C@]23CC[C@@]2(C)[C@@H](Br)C[C@]13C
1244	694	CC(C)(Cl)[C@H](Br)CC[C@@](Cl)(CBr)C(Cl)=C
1245	169	CC1=CC(O)=C2C(OC3=CC=C(O)C(C([O-])=O)=C3C2=O)=C1
1246	985	CC(=O)\C=C\C1=CC(Br)=C(O)C(O)=C1
1247	280	OC1=CC(CCOS([O-])(=O)=O)=C(Br)C(Br)=C1O
1248	184	COC1=CC(C)=C2O[C@@](C)(CC3=C[C@]4(C)CCC[C@]4(C)[C@@]4(C[C@@H](O)C(C)(C)O4)O3)CCC2=C1
1249	1577	CO[C@@H]1[C@@H](O)[C@@H](O)[C@@H](O[C@H]1CO[C@@H]1C[C@H](O)[C@H](N)CO1)N1C2=C(C=CC=C2)C2=C1C1=C(C3=CC=CC=C3N1)C1=C2C(=O)N(C)C1=O
1250	30	CC(=O)O[C@H]1C=C(C)[C@H](CBr)O[C@@]2(C)CC[C@H](Br)C(C)(C)[C@H]12
1251	1314	CC(C)C1=CC[C@@]2(C)C[C@@]3(O)C(=C)CC[C@H](OC(C)=O)[C@]3(C)CC=C12
1252	1000	CC1(C)[C@@H](Br)C[C@H](Cl)\C(=C/CCl)[C@H]1Cl
1253	350	C[C@@H](CCC=C(C)C)[C@@H]1[C@H](O)CC(=C)[C@@H]2CC=C(C)[C@H]2[C@@H]1O
1254	602	CC(=O)O[C@H]1C[C@@H](Br)C(C)(C)O[C@]1(C)[C@@H]1C[C@H](Br)[C@@](C)(Cl)C[C@H]1O
1255	475	OC[C@@H](O)COCC1=CC(O)=C(O)C(Br)=C1Br
1256	1239	CC[C@@H](CCC(=O)C(Br)=C(Br)Br)OC(C)=O
1257	1671	CC(C)[C@H]1NC(=O)[C@H](NC(=O)C2=CC=C(C)C3=C2N=C2C(O3)=C(C)C(=O)C(N)=C2C(=O)N[C@H]2[C@@H](C)OC(=O)[C@@H](C(C)C)N(C)C(=O)CN(C)C(=O)[C@@H]3CCCN3C(=O)[C@H](NC2=O)C(C)C)[C@@H](C)OC(=O)[C@@H](C(C)C)N(C)C(=O)CN(C)C(=O)[C@H]2CCN2C1=O
1258	1234	CC(=O)O[C@@H]1C[C@]2(C)[C@H](C[C@@H](O)[C@@H]3C[C@](C)(CC=C23)[C@@H](Br)CO)[C@@]2(CO)C[C@@H]12

1259	125	C[C@@]1(Cl)C[C@](C)(\C=C\Cl)[C@@H](Br)C[C@@H]1Br
1260	498	C[C@H](O)[C@H](C)\C=C\C[C@H]1CO[C@@H]([C@H](O)C(\C)=C\C(=O)OCCCCCCCCCC(=O)NC2=C3SSC=C3NC2=O)[C@H](O)[C@@H]1O
1261	764	C[C@H]1CCCCCC(=O)C2=C(O)C=C(O)C=C2CC(=O)O1
1262	878	OC1=CC(CC([O-])=O)=C(Br)C(Br)=C1O
1263	1548	CC[C@H]1O[C@H](C[C@@H](OC(C)=O)[C@H](OC(C)=O)\C=C/1)[C@H](Cl)C\C=C/C#C
1264	1305	O[C@H]1C[C@@H](O)C2=C(C=CC=C2O)C1=O
1265	780	CC[C@@]1(O)CCC2=C(O)C3=C(C=C2[C@@H]1C(=O)OC)C(=O)C1=CC=CC(O)=C1C3=O
1266	949	COC1=C2C(=O)OCC2=C(C)C2=C1C[C@@H]1[C@](C)(C[C@@H](O)[C@]34O[C@@](CC[C@]13C)(OC)OC4(C)C)O2
1267	663	CC(=O)OC[C@H](Br)[C@@]1(C)CC=C2[C@@H](C1)[C@@H](C[C@@H]1[C@@]3(O)CC[C@H]3[C@@H](C[C@@]21C)OC(C)=O)OC(C)=O
1268	1494	C[C@@](Cl)(\C=C\Br)[C@H](Cl)\C=C\C(=C\Cl)\C(Cl)Cl
1269	139	COC(=O)[C@]1(CC2=CC(CC=C(C)C)=C(O)C=C2)OC(=O)C(O)=C1C1=CC=C(O)C=C1
1270	31	CCCCC1=CN=C(C=C1)C(=O)OC
1271	1589	C[C@H]1CC[C@H]2C(C)(C)CCC[C@]2(C)\C1=C\C1=CC(OS([O-])(=O)=O)=C(O)C(C=O)=C1OS([O-])(=O)=O
1272	518	C[C@@]1(Cl)C[C@@](C)(Br)[C@@H](C[C@@H]1Cl)\C=C\Cl
1273	492	CO[C@@H](CC[C@H](C)[C@H]1O[C@@]23C[C@H](OC(=O)C[C@@H](OC(=O)C[C@](O)(O2)[C@H](C)CC3(C)C)[C@@H](C)O)[C@@H]1C)C1=C(Br)C=CC(O)=C1
1274	376	CC(C)[C@@H]1CC=C[C@H]2[C@@H]3[C@](C)(O)C(=O)C[C@@H](O)[C@@]3(C)CC[C@]12CBr
1275	160	CCCCCCCC[C@@H]1CC(=O)N[C@@H](CC2=CC=CC=C2)C(=O)N[C@@H](C)C(=O)N[C@H](CC(C)C)C(=O)O1
1276	1260	COC1=C(Br)C=C(C=C1O)C([O-])=O
1277	371	CC[C@H]1O[C@H](C[C@@H](Br)[C@H](OC(C)=O)\C=C/1)[C@H](Cl)C\C=C/C#C
1278	762	CC(=O)O[C@H]1C[C@@H](Br)C(C)(C)[C@H]2[C@@H](O)C[C@]3(C)O[C@](C)(CC[C@H]3[C@]12C)C=C
1279	128	OC1=CC(OC2=C3OC4=C(O)C=C(O)C=C4OC3=C(O)C=C2O)=CC(O)=C1
1280	1258	C[C@H]1CC(=O)O[C@H]1[C@@]1(C)CC(=O)C2=C(O)C(=CC=C2O1)C1=C2O[C@](C)(CC(=O)C2=C(O)C=C1C)[C@@H]1OC(=O)C[C@H]1O
1281	750	CC\C=C\C=C/[C@]1(C)OC(OC)=C(C(=O)C[C@H](C)O)C1=O
1282	1417	C[S@@](=O)C1=C(C2=C(NC3=CC(Br)=CC(Br)=C23)[S@@](C)=O)C2=C(Br)C=C(Br)C=C2N1
1283	499	CC(C)CCC[C@H](C)CCC[C@@H](C)CCC\C(C)=C\CC1=CC(OC(C)=O)=CC(C)=C1OC(C)=O
1284	43	CCC[C@H](OC(C)=O)C1=C(Br)\C(OC1=O)=C/Br
1285	575	NC(=O)OC[C@@H]1N=C(N)N2CCC(O)(O)[C@@]22N=C(N)N[C@@H]12
1286	1210	CCCCCC[C@H](C)[C@@H]1CC(=O)NCC(=O)N[C@@H](C(C)C)C(=O)N[C@@H](CC(C)C)C(=O)N[C@@H](C)C(=O)N[C@@H](CC2=CC=CC=C2)C(=O)O1
1287	429	CO[C@]12CCN(CC3=C(Br)C(Br)=C(O)C(O)=C3)[C@H]1NC(=O)N2
1288	391	CN(\C=C\CC=C)C(=O)CCCCCCCC\C=C\[C@H](CSSC[C@H](NC(C)=O)\C=C\CCCCCCCCC(=O)N(C)\C=C\CC=C)NC(C)=O
1289	592	CO[C@]1(NC(=O)[C@H](NC(N)=O)C2=CC=CS2)[C@@H]2SCC(CSC3=NN=NN3C)=C(N2C1=O)C([O-])=O
1290	1248	COC1=C(Br)C=C(CC([O-])=O)C=C1O
1291	731	C[C@@H]1CC2=C(C(=O)O1)C(O)=C(C=C2)C([O-])=O

1292	775	CC(C)=CCC\C(C)=C\CC[C@](C)(O)[C@H](O)CC1=CNC(=C1)[N+]([O-])=O
1293	707	COC1=CC(CCN(C)C)=C2SC3=C(OC)C(OC)=C(SC)C(CCN(C)C)=C3SSC2=C1O
1294	1044	CCC[C@@H](OC(C)=O)C1=C(Br)\C(OC1=O)=C\Br
1295	59	CC(=O)O[C@@H]1C[C@@]2(C)[C@H](C[C@H](O)[C@H]3C[C@](C)(CC=C23)[C@@H](Br)CO)[C@]2(O)CC[C@H]12
1296	784	CO[C@H]1C[C@H](C)[C@H](OC)C2=CC(O)=CC(NC(=O)\C(C)=C/C=C/[C@H](OC)[C@@H](OC(N)=O)\C(C)=C\[C@H](C)[C@@H]1O)=C2O
1297	483	OC1=CC=C(CC([O-])=O)C=C1
1298	117	COC1=CC(CCN(C)C)=C2SC3=C(O)C(OC)=CC(CCN(C)C)=C3SSC2=C1O
1299	507	C\C(=C\Br)\C=C\[C@@H](Cl)[C@](C)(Cl)\C=C\Br
1300	76	COC1=CC(\C=N\O)=NC(=C1)C1=CC=CC=C1
1301	1279	COC1=CC(C)=C2O[C@](C)(CC\C=C(/C)C=O)C=CC2=C1
1302	898	C[C@](Cl)(\C=C/Br)[C@@H](Cl)\C=C\C(=C\Cl)\C(Cl)Cl
1303	946	C[C@@H]1C(=O)[C@@H](Br)[C@H](C)[C@@]2(C)C[C@H](Br)[C@]3(C)CC[C@]123
1304	831	CC(=O)O[C@H]1C[C@@](C)(Cl)[C@H](Br)C[C@@]11C(=C)C(=O)C=CC1(C)C
1305	1472	C[C@@](Cl)(CBr)\C=C\[C@H](Br)[C@](C)(Cl)\C=C\Br
1306	471	CCC\C=C/C[C@@H](O[C@@H](\C=C\[C@H](C\C=C/CCCC(=O)OC)OC[C@H]1O[C@@H](OC[C@@H](O)CO)[C@@H](O)[C@@H](O)[C@H]1O)[C@H](O)C\C=C/C\C=C/CC)[C@H](O)\C=C\[C@H](O)CCCCC(=O)OC
1307	1241	CC(=O)OCC[C@]1(C)CC=C2[C@@H](C1)[C@@H](C[C@@H]1[C@]3(COC(C)=O)C[C@H]3[C@@H](C[C@]21C)OC(C)=O)OC(C)=O
1308	969	CO[C@@H]1[C@H](C)[C@@]2(C)C[C@H](Br)[C@]3(C)CC[C@]23[C@@H](C)C1=O
1309	948	OC1=CC(OC2=C(O)C=C(O)C3=C2OC2=C3C3=C(OC4=C(O3)C(O)=CC(O)=C4OC3=CC(O)=CC(O)=C3)C(O)=C2)=CC(O)=C1
1310	990	C[C@]1(CC(=O)[C@]2(O)C(=C1)C(=O)C(O)=C1[C@]22C[C@H]2CC[C@@]1(C)C([O-])=O)C=C
1311	260	CNC1=NC2=C(CC3=CC(Br)=C(OC)C(Br)=C3)C(=O)NC=CC2=N1
1312	1348	COC1=CC(C\C=C(/C)CC2=C[C@]3(C)CCC[C@]3(C)[C@@]3(C[C@@H](O)C(C)(C)O3)O2)=C(OC)C(C)=C1
1313	936	NC1=C2N=CN(CC3=CC(O)=C(O)C(Br)=C3CC3=C(Br)C(Br)=C(O)C(O)=C3)C2=NC=N1
1314	1401	CC(=O)\C=C\C1=C(Br)C(Br)=C(O)C(O)=C1
1315	497	C[C@@](Cl)(\C=C\Br)[C@@H](Cl)\C=C\C(=C\Cl)\C(Cl)Cl
1316	108	C[C@H]1CC[C@@H]2[C@@]3(C)CC[C@H](OC(C)=O)C(C)(C)[C@@H]3CC[C@@]2(C)[C@]11CC2=C(O1)C(C)=C(Cl)C(OC(C)=O)=C2OC(C)=O
1317	608	COC1=CC(=NC(CO)=C1)C1=NC=CC=C1
1318	141	COC1=C2C(=O)C3=C(O)C=CC=C3OC2=C([C@@H](CO)[C@@H](O)CO)C(O)=C1
1319	692	BrCCBr
1320	470	CCCCCCCC\C=C\CCCCCCCC(=O)O[C@@H](CNC(=O)C(\C)=C\C)C1=CC(=O)C2=C3C(=CC=N2)C2=CC=CC=C2N=C13
1321	676	CCC\C=C/C[C@@H](O[C@@H](\C=C\[C@H](C\C=C/CCCC([O-])=O)OC[C@H]1O[C@@H](OC[C@@H](O)CO)[C@@H](O)[C@@H](O)[C@H]1O)[C@H](O)C\C=C/C\C=C/CC)[C@H](O)\C=C\[C@H](O)CCCCC([O-])=O
1322	1200	CC(C)=CCC\C(=C/Br)C=C
1323	428	CC(C)=CCCC(=C)[C@]1(O)CCC(C)=CC1

1324	1712	C[C@@H](C[C@@H](C)C(=O)C(\C)=C\[C@H](C)[C@H]1O[C@@]2(CC[C@](C)(O2)[C@@H]2CC[C@H](C)[C@@]3(O[C@H](C[C@H]3C)[C@@H]3O[C@@](O)(CO)[C@H](C)C[C@H]3C)O2)C[C@@H](O)[C@H]1C)C([O-])=O
1325	549	CC(C)C1=C2[C@H](C[C@@]3(C)[C@H](CCC(=C)[C@]3(O)C[C@]2(C)CC1)OC(C)=O)OC(C)=O
1326	1685	CC(=O)O[C@@H](C[C@H]1C(=C)C[C@H](OC(C)=O)[C@@H]2C(C)(C)[C@H](Br)CC[C@@]12C)[C@@](C)(O)C=C
1327	1711	CCCCCCCCCCC[C@@H]1CC(=O)N[C@@H](CC([O-])=O)C(=O)N[C@H](CC2=CC=C(O)C=C2)C(=O)N[C@H](CC(N)=O)C(=O)N[C@@H](CO)C(=O)N[C@H](CCC([O-])=O)C(=O)N[C@@H](CO)C(=O)N[C@@H]([C@H](C)O)C(=O)N1
1328	112	CC(C)CC(=O)N(O)CCCCCNC(=O)CCC(=O)N(O)CCCCCNC(=O)CCC(=O)N(O)CCCCCN
1329	361	CC(C)C[C@@H]1NC(=O)[C@@H]2CCCN2C1=O
1330	1084	C[C@H](O)[C@H](C)\C=C\C[C@H]1CO[C@@H](C\C(C)=C\C(=O)OCCCCCCCC(=O)NC2=C3SSC=C3NC2=O)[C@H](O)[C@@H]1O
1331	1295	OC1=CC(O)=C2OC3=CC(O)=C4OC5=CC(O)=CC(O)=C5OC4=C3OC2=C1
1332	413	C[C@H]1[C@]2(CBr)CC[C@@]1(C)C1=CC(Br)=C(C)C(Br)=C1O2
1333	1086	C\C(=C\Br)\C=C\[C@H](Cl)[C@](C)(Cl)\C=C\Br
1334	1482	CC(C)=CCC\C(=C\Br)C=C
1335	1668	OC1=C(C(=O)[C@@H]2O[C@@]2(C1=O)C1=CC=CC=C1)C1=CC=CC=C1
1336	1216	CC(=O)O[C@@H]1C[C@@]2(C)[C@@H](C[C@H](O)[C@H]3C[C@](C)(CC=C23)[C@@H](Br)CO)[C@@]2(C)C[C@@H]12
1337	1257	OCC1=CC=C(O1)C=O
1338	997	COC1=C2NC3=C(C=CN=C3C)C2=C(Br)C=C1
1339	709	N[C@@H](C(=O)N[C@H]1[C@@H]2SCC(CSC3=CNN=N3)=C(N2C1=O)C([O-])=O)C1=CC=C(O)C=C1
1340	941	CC(C)C1=C2[C@H](C[C@@]3(C)[C@@H](O)CCC(=C)[C@]3(O)C[C@]2(C)C[C@H]1OC(C)=O)OC(C)=O
1341	1619	CC(C)CCCCCCCCC[C@H]1CC(=O)N[C@@H](CC(N)=O)C(=O)N[C@@H](CC2=CC=C(O)C=C2)C(=O)N[C@H](CC(N)=O)C(=O)N2CCC[C@H]2C(=O)N[C@@H](CCC([O-])=O)C(=O)N[C@H](CO)C(=O)N[C@@H]([C@@H](C)O)C(=O)N1
1342	1622	CCCCCCCC[C@H]1OC(=O)CNC(=O)[C@H](NC(=O)[C@H](CO)NC(=O)[C@@H](NC(=O)[C@H](CC(C)C)N(C)C(=O)[C@H]1C)C(C)C)[C@H](C)O
1343	1082	CSC
1344	1531	CCCCCCCCCCCCCCCC[C@H](O)C([O-])=O
1345	513	CC(=C)[C@H](Cl)CC\C(=C\Br)C=C
1346	34	CCC[C@@H](OC(C)=O)C1=C(Br)C(OC1=O)=C(Br)Br
1347	675	COC1=CC(OC)=C2C(O)=C3C(=O)C[C@@](C)(O)OC3=CC2=C1
1348	1585	C[C@@H]1[C@]2(C)CC[C@@]1(CO)OC1=CC(C)=C(Br)C=C21
1349	626	N[C@@H](CCCC(=O)N[C@@]1(NC=O)[C@@H]2SCC(COC(=O)C[C@@H](O)[C@@H](CCCNC(N)=N)NC(=O)[C@H](CO)NC(=O)[C@@H](N)CO)=C(N2C1=O)C([O-])=O)C([O-])=O
1350	1575	CC(=O)N[C@@H]1[C@@H](O)[C@H](OS([O-])(=O)=O)[C@@H](CO)O[C@H]1O[C@H]1C[C@H](N[C@H]1CO)C([O-])=O
1351	1332	CC(C)CCCCCCCCC[C@H]1CC(=O)N[C@H](CC(N)=O)C(=O)N[C@@H](CC2=CC=C(O)C=C2)C(=O)N[C@H](CC(N)=O)C(=O)N[C@H](CCC([O-])=O)C(=O)N2CCC[C@H]2C(=O)N[C@H](CC(N)=O)C(=O)N[C@H]([C@H](C)O)C(=O)N1
1352	161	CC[C@H]1O[C@H](C[C@@H](OC(C)=O)[C@H](O)\C=C/1)[C@H](Cl)C\C=C/C#C

1353	992	C[C@@H](N)C(=O)N[C@@H](C)C(=O)N[C@H](CCCNC(N)=N)[C@H](O)CC(=O)OCC1=C(N2[C@@H](SC1)[C@H](NC(=O)CCC[C@@H](N)C([O-])=O)C2=O)C([O-])=O
1354	1391	C[C@H](N)C(=O)N[C@H](CCCNC(N)=N)[C@H](O)CC(=O)OCC1=C(N2[C@@H](SC1)[C@H](NC(=O)CCC[C@@H](N)C([O-])=O)C2=O)C([O-])=O
1355	970	[O-]C(=O)CC1=CC=CC=C1
1356	366	CC(C)C1=CC[C@@]2(C)CC3=C(CCC=C3[C@H](C[C@H]12)OC(C)=O)C=O
1357	1173	COC1=C2C(O)=C3C(=O)C[C@@](C)(O)OC3=CC2=CC(O)=C1
1358	781	CC(C)=C[C@@H](O)CC(=C)[C@@]1(O)CC[C@](C)(Br)[C@@H](Cl)C1
1359	384	C[C@H](CCC=C(C)C)[C@H]1[C@@H](O)C\C(C)=C/C\C=C2\C(=O)O[C@H](O)[C@]12C
1360	1138	OC1=C2N=C3C=CC(Cl)=C(O)C3=NC2=CC=C1
1361	695	C[C@H](O)[C@H](C)\C=C\C[C@H]1CO[C@@H]([C@H](O)C(\C)=C\C(=O)OCCCCCCCC(=O)NC2=C3SSC=C3NC2=O)[C@H](O)[C@@H]1O
1362	146	C[C@H](CCC=C(C)C)[C@@H]1CC[C@]2(C)CC=C(C=O)[C@@]1(C=O)[C@H]2O
1363	596	NC1=NC(=CS1)C(=N/OCC([O-])=O)\C(=O)N[C@H]1[C@@H]2SCC(C=C)=C(N2C1=O)C([O-])=O
1364	460	CC(=O)OC[C@H](Br)[C@@]1(C)CC=C2[C@@H](C1)[C@@H](C[C@@H]1[C@@]3(COC(C)=O)C[C@H]3[C@@H](C[C@@]21C)OC(C)=O)OC(C)=O
1365	1460	BrCBr
1366	1695	C[C@@]12CC[C@@]3(C[C@]1(Cl)O2)C(=C)C[C@H](O)[C@H](Br)C3(C)C
1367	240	CNC1=NC2=C(CC3=CC(Br)=C(OC)C(Br)=C3)C(=O)N(C)C=CC2=N1
1368	153	COC1=CC(C)=CC(C([O-])=O)=C1C1=CC(=O)C2=C(C=CC=C2O)C1=O
1369	451	CC(=O)O[C@@H]1C[C@@]2(C)[C@H](C[C@H](OC(C)=O)[C@H]3C[C@](C)(CC=C23)[C@@H](Br)CO)[C@@]2(C)C[C@@H]12
1370	1312	CC1=C(O)C2=C(C[C@@](C)(O)OC2=O)C(C)=C1O
1371	1134	CC(=O)C1=C(O)C(C)=C(O)C(C)=C1
1372	758	COC(=O)\C=C(/C)CC\C=C(/C)CC\C=C(/C)[C@@H](O)CC=C(C)C
1373	792	CC(=O)OCC1=C(N2[C@@H](SC1)[C@H](NC(=O)C1=C(C)ON=C1C1=C(Cl)C=CC=C1)C2=O)C([O-])=O
1374	202	CCC(=O)N[C@@H](CO)[C@H](OC(C)=O)C1=CC=C(C=C1)[N+]([O-])=O
1375	1543	C[C@H](C[C@H](C)C=C(C)C)\C=C\C(=O)NC1=C[C@](O)(CCCCC([O-])=O)[C@@H]2O[C@@H]2C1=O
1376	1603	N[C@@H](CCCNC(N)=N)C(=O)N[C@H]1[C@@H](O)[C@@H](O)[C@@H](CO)O[C@@H]1O[C@@H]1[C@H](O)[C@@H](O)[C@@H](O[C@H]2[C@H](O)[C@@H](NC(=O)[C@@H]3CCC(N)=N3)[C@H](O)[C@@H](O)[C@@H]2OC(N)=O)O[C@@H]1CO
1377	1272	COC1=CC(=NC(=C1)C#N)C1=NC=CC=C1
1378	207	[O-]C(=O)C1=C(NC=N1)C(=O)N[C@@H](C(=O)N[C@H]1[C@H]2SCC(C[N+]3=CC=C(CCS([O-])(=O)=O)C=C3)=C(N2C1=O)C([O-])=O)C1=CC=CC=C1
1379	375	CC1=C(O)C(O)=C2COCC2=C1O
1380	1586	C[C@@H](NC(=O)[C@@H](N)CO)C(=O)N[C@@H](CO)C(=O)N[C@@H](CCCCN)[C@@H](O)CC(=O)OCC1=C(N2[C@@H](SC1)[C@](NC=O)(NC(=O)CCC[C@@H](N)C([O-])=O)C2=O)C([O-])=O
1381	1130	OC1=CC(CN2C=NC3=C2C(=O)NC(=O)N3)=C(Br)C(Br)=C1O
1382	1535	OC[C@H](NC(=O)C(Cl)Cl)[C@@H](O)C1=CC=C(C=C1)[N+]([O-])=O
1383	323	CCCC1=C(Cl)C(OC)=C(Cl)C(O)=C1C([O-])=O
1384	569	CC(=O)NCCSC1=C(N2[C@H](C1)\C(=C(\C)CO)C2=O)C([O-])=O
1385	1503	COC1=C(OC)C(=NC(\C=N\O)=C1)C1=NC=CC=C1

1386	978	CC[C@H](C)CCC(=O)N[C@H](C(C)C)C(=O)N[C@@H]([C@@H](C)O)C(=O)N[C@@H](C(C)C)C(=O)N[C@H](C(C)C)C(=O)N1CCC[C@@H]1C(=O)N[C@@H](CCCN)C(=O)N[C@H]([C@@H](C)CC)C(=O)N[C@@H]1[C@@H](C)OC(=O)[C@@H](NC(=O)\C(NC(=O)[C@H](CC2=CC=CC=C2)NC(=O)[C@H](NC(=O)[C@H](NC1=O)[C@@H](C)CC)C(C)C)=C\C)C(C)C
1387	1269	CCCCC\C=C/C[C@@H](O)[C@@H](O[C@H](C\C=C/CCC)[C@H](O)\C=C\[C@H](O)CCCCC(=O)OC)\C=C\[C@H](C\C=C/CCCC(=O)OC)OC[C@H]1O[C@@H](OC[C@@H](O)CO)[C@H](O)[C@@H](O)[C@@H]1O
1388	1103	CCCCC[C@H](O)[C@@H]1C(=O)N[C@@]2([C@@H](O)[C@H]3CCCC=C3)C(=O)O[C@@]12C
1389	1367	CC(=O)NCCSC1=C(N2[C@H](C1)[C@H](C2=O)C(C)(C)O)C([O-])=O
1390	636	CONC(=O)C1=CC(OC)=CC(=N1)C1=NC=CC=C1
1391	179	C[C@H]1NC(=O)[C@@H](CC2=CNC3=CC=CC=C23)NC1=O
1392	131	COC1=CC2=C([C@H]3C=CO[C@@H]3O2)C2=C1C1=C(C(=O)[C@H](O)C1)C(=O)O2
1393	718	CC1(C)[C@@H]2CCC(=C)[C@]11C[C@H]3[C@@](C)(O)CC[C@@H](Br)[C@]3(C)C[C@@H]1O2
1394	716	CC(C)=CCCC(=C)C(Cl)=C
1395	699	CO[C@H]1C2=C(O)C(O)=C(Br)C(Br)=C2[C@@H](OC)C2=C(O)C(O)=C(Br)C(Br)=C12
1396	1278	C[C@H](O)[C@H](C)\C=C\[C@H]1CO[C@@H]([C@H](O)C(\C)=C\C(=O)OCCCCCCCC(=O)NC2=C3SS(=O)(=O)C=C3NC2=O)[C@H](O)[C@@H]1O
1397	589	CC(=O)O[C@@H]1C[C@](C)(Cl)[C@@H](Br)C[C@H]1[C@@]1(C)OC(C)(C)[C@H](Br)C[C@@H]1OC(C)=O
1398	299	C\C(CCl)=C/C=C(\Cl)/C(/C)=C/C=O
1399	1459	C[C@@H]1CC(=O)[C@@]23[C@@H](C)C(=C[C@]2(C)CC[C@@H]13)C([O-])=O
1400	124	OC1=CC(OC2=C3OC4=C(O)C=C(OC5=C(O)C=C(OC6=C7OC8=C(O)C=C(O)C=C8OC7=C(O)C=C6O)C=C5O)C=C4OC3=C(O)C=C2O)=CC(O)=C1
1401	876	CBr
1402	1670	CC(C)CCCCCCCCCC[C@@H]1CC(=O)N[C@@H](CC([O-])=O)C(=O)N[C@H](CC2=CC=C(O)C=C2)C(=O)N[C@@H](CC(N)=O)C(=O)N[C@@H](CO)C(=O)N[C@H](CCC([O-])=O)C(=O)N[C@@H](CO)C(=O)N[C@@H]([C@H](C)O)C(=O)N1
1403	811	C[C@](O)(\C=C\[C@@H]1CC=CC(=O)O1)[C@H](C[C@@H](O)\C=C/C=C\C=C\CO)OP([O-])([O-])=O
1404	473	C\C=C(/C)C(=O)NC[C@H](O)C1=CC(=O)C2=C3C(=CC=N2)C2=CC=CC=C2N=C13
1405	735	COC1=C(C)C(=O)C2=CN(C)C=C2C1=O
1406	172	C\C=C\CCC(=O)C[C@@H](O)CCO
1407	773	[O-]C(=O)CCCCCC1=C(\C=C\C=C)C=CC=C1
1408	400	OC1=C(O)C(Br)=C(Br)C(Br)=C1Br
1409	531	COC1=CC(=O)C2=C(C(O)=C3C[C@](C)(O)[C@H](O)[C@@H](O)C3=C2O)C1=O
1410	624	O\N=C\C1=CC(O)=CC(=N1)C1=NC=CC=C1
1411	768	CC1(C)O[C@]2(C)[C@@H]3CC[C@](C)(O[C@@H]2C[C@H]1Br)[C@H](O)C3
1412	334	CNC1=C(C([O-])=O)C2=C(OC(C[C@H]3O[C@@]4(CC[C@@H]3C)O[C@@H]([C@H](C)C(=O)C3=CC=CN3)[C@H](C)C[C@@H]4C)=N2)C=C1
1413	189	C[C@@H]1OC2=C(C(=O)C(=N)C3=C(O)C(O)=CC(C=O)=C23)C1(C)C
1414	1438	[O-]C(=O)CNC(=O)\C=C\CCCCCCCCCC=C(Br)Br
1415	1389	COC1=CC=CC2=C1C(=O)C1=C(O)C3=C(O[C@@]4(C)C[C@H](O)C[C@@H]3O4)C=C1O2
1416	583	CC(C)C[C@@H](NC(=O)C1=C(C)NC(=O)C(=N1)C(C)C)C(=O)N[C@@H](C)C([O-])=O

1417	61	CC(=O)O[C@@H]1C[C@]2(C)[C@H](C[C@@H](OC(C)=O)[C@@H]3C[C@](C)(CC=C23)[C@@H](Br)CO)[C@@]2(CO)C[C@@H]12
1418	1163	C[C@@H]1OC2=C3C4=C(NC(=O)C4=C(O)C=C3C)C(O)=C2C1(C)C
1419	632	COC1=CC(=NC(\C=N/O)=C1)C1=NC=CC=C1
1420	717	[O-]C(=O)C#CC([O-])=O
1421	424	CC(=O)N(O)CCCP([O-])([O-])=O
1422	417	COC1=CC(=NC(=C1)C(N)=O)C1=NC=CC=C1
1423	1119	CC(C)(SCC1=C(N2[C@H](SC1)[C@H](NC(=O)CCC[C@H](N)C([O-])=O)C2=O)C([O-])=O)[C@@H](N)C([O-])=O
1424	886	COC1=CC(OC)=C2C(O)=C3C(=O)C=C(C)OC3=CC2=C1
1425	534	CCO[C@H]1OC(=O)C2=C(OC)C(C)=C(O)C(CO)=C12
1426	1689	CO[C@H]1[C@H](C)O[C@@H]2OC3=C(O[C@]2(OC)[C@@H]1O)C=C(\C=N\O)N=C3C1=NC=CC=C1
1427	345	OC1=CC2=NC3=C(O)C=CC=C3N=C2C(=C1)C([O-])=O
1428	1374	CC(=O)N[C@H]1CON(C(C)=O)C1=O
1429	1624	CO[C@@]1(NC(=O)C2SC(S2)=C(C(N)=O)C([O-])=O)[C@@H]2SCC(CSC3=NN=NN3C)=C(N2C1=O)C([O-])=O
1430	313	CC(C)CC(=O)N(O)CCCCCNC(=O)CCC(=O)N(O)CCCCCNC(=O)CCC([O-])=O
1431	1132	COC1=C2C(=O)C3=C(C(O)=C4C[C@](O)(C[C@@H](O[C@@H]5C[C@H](N)[C@@H](O[C@@H](C[C@@H](C)O)O[C@@H](C)CO)[C@H](C)O5)C4=C3O)C(C)=O)C(=O)C2=CC=C1
1432	543	CC(C)=CCC[C@H]1CO[C@@H]2O[C@@H](O)\C3=C\C\C=C(C)/CC[C@@H]1[C@H]23
1433	135	C[C@@H]1CC[C@H]2[C@@H]1[C@@H]1[C@@H](C[C@@H](OC(C)=O)[C@]21C)C(=C)C[C@H](OC(C)=O)C=C(C)C
1434	113	CCOCC1=CC(OC2=C(Br)C(O)=C(O)C=C2COCC)=C(O)C(Br)=C1Br
1435	1398	CC(=O)NCC1=CC(O)=CC(=N1)C1=NC=CC=C1
1436	1644	CN[C@@H]1[C@H](O)[C@H](CO)O[C@@H](O[C@H]2[C@@H](N)C[C@@H](N)[C@H](O[C@@H]3OC(CN)=CC[C@@H]3N)[C@@H]2O)[C@@H]1O
1437	911	CCCCC1=CN=C(C=C1)C(N)=O
1438	585	C[C@@H]1[C@H](CC(=O)[C@H](C)[C@@]1(C)\C=C\C(\C)=C/CC1=C(O)C(Cl)=C(C)C(C=O)=C1O)OC(C)=O
1439	183	CCC1=C(N=C(C(C)C)C(=O)N1)C(=O)N[C@H](CC(C)C)C(=O)N[C@@H](C)C([O-])=O
1440	33	NC1=NC2=C(N1)C(=NC=C2)C1=CC(Br)=C(Br)N1
1441	965	CO[C@H]1CC(=N[C@@]11O[C@@H](C)C[C@@H]2CCCCCCCC3=CC=C(N3)[C@@H]12)C1=CC=CN1
1442	691	CC(C)=CCCC(=C)C=C
1443	1458	OC[C@H]1O[C@H]([C@H](O)[C@H]1O)N1C=NC2=CN=C(NCC3=C(Br)C(Br)=C(O)C(O)=C3)N=C12
1444	1470	C[C@H](\C=C\C[C@H]1CO[C@@H]([C@H](O)C(\C)=C\C(=O)OCCCCCCCC(=O)NC2=C3SSC=C3NC2=O)[C@H](O)[C@@H]1O)C(C)=O
1445	1296	C[C@H]1CC2=C(C=C3C(C)=CC=C13)C(C)=CO2
1446	919	COC1=CC(=NC(\C=N\O)=C1)C1=NC=CC=C1
1447	1054	COC1=CC(O)=C2C(=O)C3=C(O)C=C(C)C=C3C(=O)C2=C1
1448	431	COC1=C2C(=O)C3=C(O)C([C@H]4CCC[C@@H](C)O4)=C(O)C(Cl)=C3OC2=CC=C1
1449	1719	C[C@H](CCC=C(C)C)[C@@H]1[C@H](O)C[C@]2(C)O[C@H]2C\C=C(C=O)/[C@H]1COC(C)=O
1450	450	CC(C)C[C@@H]1NC(=O)[C@H](CC2=CC=CC=C2)NC(=O)[C@H](CC2=CC=CC=C2)N(C)C(=O)[C@H](CC2=CC=CC=C2)NC(=O)C(C)(C)NC(=O)[C@H](CC(C)C)N(C)C(=O)[C@@H]2CCCN2C1=O

1451	1654	CCCCCCCCCCCCCC[C@@H]1CC(=O)N[C@@H](CC([O-])=O)C(=O)N[C@H](CC2=CC=C(O)C=C2)C(=O)N[C@@H](CC(N)=O)C(=O)N[C@@H](CO)C(=O)N[C@H](CCC([O-])=O)C(=O)N[C@@H](CO)C(=O)N[C@@H]([C@H](C)O)C(=O)N1
1452	1292	CC(C)CC(=O)N(O)CCCCCNC(=O)CCC([O-])=O
1453	1721	CCCCCCCC[C@H](O)[C@@H](O)CCCCCCCCCCCCCCCC1=CC(OS([O-])(=O)=O)=CC(OS([O-])(=O)=O)=C1
1454	979	C[C@H](CCC=C(C)C)[C@@H]1[C@H](O)C\C(C)=C/C\C=C(C=O)/[C@H]1COC(C)=O
1455	612	COC1=CC(O)=C2C(=O)C3=C(C[C@@](C)(O)[C@H](O)C3)C(=O)C2=C1
1456	1335	CC1=CC(O)=C2C(=O)C3=C(O)C(O)=CC=C3C(=O)C2=C1C1=C(C)C=C2C(=O)C3=CC=C(O)C(O)=C3C(=O)C2=C1O
1457	1717	NC[C@H]1O[C@H](O[C@@H]2[C@@H](N)C[C@@H](N)[C@@H](O[C@@H]3O[C@H](CO)[C@@H](O)[C@H](O)[C@H]3O)[C@H]2O)[C@@H](O)[C@H](O)[C@H]1O
1458	1316	CCC1=CC=C2C(O)=C3C(=O)C4=C(O)C=CC=C4C(=O)C3=CC2=C1C(=O)OC
1459	1576	CC(C)CCCCCCCCC[C@@H]1CC(=O)N[C@@H](CC([O-])=O)C(=O)N[C@H](CC2=CC=C(O)C=C2)C(=O)N[C@@H](CC(N)=O)C(=O)N[C@@H](CO)C(=O)N[C@H](CCC([O-])=O)C(=O)N[C@@H](CO)C(=O)N[C@@H]([C@H](C)O)C(=O)N1
1460	550	CNCC(=O)N[C@@H](CNC(N)=N)C(=O)N[C@@H]1[C@H](O)[C@H](O)[C@H](O[C@H]1C(=O)NCCCNCCCCN)N1C=CC(N)=NC1=O
1461	883	CCC(C)(C)[C@H](NC(=O)[C@H](NC(=O)[C@@H]1CCCN1C(=O)[C@H](NC(=O)[C@@H](C)NC=O)[C@H](O)C1=CC=C(Br)C=C1)C(C)(C)C)C(=O)N[C@H](CC1=CNC2=CC=CC=C12)C(=O)N[C@@H](CCCNC(N)=N)C(=O)N[C@H](CS([O-])(=O)=O)C(=O)N[C@H]1[C@@H](C)OC(=O)[C@@H](CC([O-])=O)NC(=O)[C@@H]2CCCN2C(=O)[C@H](NC(=O)[C@H](CCC(N)=O)N(C)C1=O)C(C)C
1462	219	CO\N=C(\C(=O)N[C@H]1[C@@H]2SCC(CSC3=NC(C)=C(CC([O-])=O)S3)=C(N2C1=O)C([O-])=O)C1=CSC(N)=N1
1463	1347	COC1=C2C(=O)C3=C(C(O)=C4C[C@](O)(C[C@@H](O[C@@H]5C[C@H](NC=O)[C@@H](O)[C@H](C)O5)C4=C3O)[C@@H](C)O)C(=O)C2=CC=C1
1464	1286	CCCCCCCCCCCCCCCC(=O)C1=C(OC(C)=O)C=C(OC(C)=O)C=C1OC(C)=O
1465	35	CO\N=C(\C(=O)N[C@H]1[C@@H]2SCC(CSC3=NC(=O)C(O)=NN3C)=C(N2C1=O)C([O-])=O)C1=CSC(N)=N1
1466	316	COC1=C(O)C(=NC(\C=N\O)=C1)C1=NC=CC=C1
1467	1640	C[C@H](O[C@H]1O[C@@H](CO)[C@H](O)[C@H](O)[C@@H]1NC(C)=O)[C@@H](NC(=O)[C@@H](CCCCN)NC(=O)[C@H]1C[C@H](O[C@H]2O[C@@H](CO)[C@@H](OS([O-])(=O)=O)[C@H](O)[C@@H]2NC(C)=O)[C@@H](CO)N1)C(=O)N[C@@H](CO)C([O-])=O
1468	238	CC(=O)O[C@H]([C@@H]1CC[C@@]23[C@@H]1[C@@](C)(CC[C@@H]2Br)OC3=O)[C@@]1(O)CC[C@H](Br)C(C)(C)C1
1469	930	CC(C)=CCC1=CC=C(O)C2=C1OC1=CC3=C(C(O)=C1C2=O)[C@]1(O)C=CO[C@@H]1O3
1470	158	NC1=C2N=CN([C@H]3O[C@@H](CO)[C@H](O)[C@@H]3O)C2=NC(Cl)=N1
1471	1702	CSC1=C2NC(=O)\C(C)=C/C=C\[C@@H](C)[C@H](O)[C@@H](C)[C@H](O)[C@@H](C)[C@@H](O)[C@@H](C)[C@@H](O)[C@H](C)\C=C(C)/C(=O)C3=C(O)C(C)=C(O)C(C2=O)=C3C1=O
1472	404	CC(=O)N[C@H](CO)[C@H](OC(C)=O)C1=CC=C(C=C1)[N+]([O-])=O
1473	186	C[C@]12CC[C@H]3C[C@@]33[C@H]1[C@H](OC2=O)[C@H](O)C1=C[C@](C)(CC(=O)[C@]31O)C=C
1474	1676	C[C@H](O[C@H]1O[C@@H](CO)[C@@H](O)[C@H](O)[C@@H]1NC(C)=O)[C@@H](NC(=O)[C@@H](CCCCN)NC(=O)[C@H]1C[C@H](O[C@H]2O[C@@H](CO)[C@@H](OS([O-

		])(=O)=O)[C@@H](O)[C@@H]2NC(C)=O)[C@@H](CO)N1)C(=O)N[C@H](CO)C(=O)N[C@H](CCC([O-])=O)C(=O)N[C@@H](CO)C([O-])=O
1475	590	C[C@@H]1OC2=C(C(=O)C(=O)C3=CC(O)=CC(C)=C23)C1(C)C
1476	1662	CC\C(Br)=C1\O[C@@H]2C[C@H]3O[C@H]2[C@@H]1[C@H]3[C@@H](Cl)\C=C/C#C
1477	766	CC1(C)[C@H](NC(=O)C(=N/OCC([O-])=O)\C2=CSC(N)=N2)C(=O)N1OS([O-])(=O)=O
1478	155	C[C@H]1[C@H](NC(=O)C(=N/OC(C)(C)C([O-])=O)\C2=CSC(N)=N2)C(=O)N1S([O-])(=O)=O
1479	1563	CCCCC\C=C/C[C@]1(OC(C)=O)C=C(Cl)C(=O)[C@@H]1[C@@H](OC(C)=O)[C@H](OC(C)=O)[C@H](CCCC(=O)OC)OC(C)=O
1480	1636	NCC[C@@H](O)C(=O)N[C@@H]1[C@H](O)[C@@H](N)[C@@H](O[C@H]2O[C@@H](CN)[C@H](O)[C@@H](O)[C@@H]2N)[C@H](O[C@@H]2O[C@@H](CO)[C@H](O)[C@H]2O)[C@@H]1O
1481	819	CCCC[C@@H]1[C@H](OC(C)=O)[C@H](C)OC(=O)[C@@H](NC(=O)C2=C(O)C(NC=O)=CC=C2)[C@@H](C)OC1=O
1482	644	CCCCCCCC\C=C\CCCCCCCC(=O)O[C@@H](CNC(=O)C=C(C)C)C1=CC(=O)C2=C3C(=CC=N2)C2=CC=CC=C2N=C13
1483	1687	COC(=O)C[C@H](NC(=O)[C@@H](NC(=O)C\N=C(/N)[C@H]1CCCN1C(=O)[C@H](NC(=O)[C@H](NC(=O)C(C)(C)C)C(C)(C)C)C(C)C)[C@@H](C)C1=CC=CC=C1)C1=NC=CS1
1484	1633	CC(=O)N[C@@H]1[C@@H](O)[C@H](OS([O-])(=O)=O)[C@@H](CO)O[C@H]1O[C@H]1C[C@H](N[C@H]1CO)C(=O)NCCS([O-])(=O)=O
1485	1710	COC1=C2C(=O)C3=C(C(O)=C4C[C@](O)(C[C@@H](O[C@@H]5C[C@H](N)[C@H](O[C@@H](C[C@@H](C)O)O[C@@H](C)C([O-])=O)[C@H](C)O5)C4=C3O)C(C)=O)C(=O)C2=CC=C1
1486	1720	NC(=O)C1=C(N)[C@H](O)[C@H]2O[C@@H]2C1=O
1487	1220	CC(C)C[C@H]1NC(=O)[C@@H](CC(C)C)N(C)C(=O)[C@@H](CC(C)C)NC(=O)[C@@H](CC(C)C)N(C)C(=O)[C@@H](CC(C)C)NC1=O
1488	951	NC1=C2N=CN([C@H]3O[C@@H](COS(N)(=O)=O)[C@H](O)[C@@H]3O)C2=NC(Cl)=N1
1489	1600	C[C@H](NC(C)=O)C(=O)N[C@@H](C)C(=O)N[C@@H](C)C(=O)N[C@@H](CCCNC(N)=N)[C@H](O)CC(=O)OCC1=C(N2[C@@H](SC1)[C@](NC=O)(NC(=O)CCC[C@@H](NC(C)=O)C([O-])=O)C2=O)C([O-])=O
1490	330	N[C@H](CCC(=O)N[C@@H](CS)C(=O)N[C@@H](CCC(=O)N[C@H](CS)C(=O)NCC([O-])=O)C([O-])=O)C([O-])=O
1491	1707	CCCCCCC[C@@H]1CC(=O)N[C@@H](C(C)C)C(=O)N[C@H]([C@@H](O)C(C)C)C(=O)N[C@@H](C)C(=O)N[C@@H]([C@H](O)C(C)C)C(=O)N[C@@H](CCC(N)=O)C(=O)N(C)[C@@H]([C@@H](C)CC)C(=O)N[C@@H]([C@@H](O)C(N)=O)C(=O)N[C@@H]([C@@H](C)O)C(=O)N2CCC[C@H]2C(=O)N[C@H](CC(C)C)C(=O)N[C@H]([C@H](C)O)C(=O)N1
1492	660	CO[C@H](CNC(=O)C=C(C)C)C1=CC(=O)C2=C3C(=CC=N2)C2=CC=CC=C2N=C13
1493	1631	CCCCCCC[C@H]1CC(=O)N[C@H](C(C)C)C(=O)N[C@H]([C@@H](O)C(C)C)C(=O)N[C@H](C)C(=O)N[C@@H](CC(C)C)C(=O)N[C@@H](CCC(N)=O)C(=O)N(C)[C@@H]([C@@H](C)CC)C(=O)N[C@H]([C@@H](O)C(N)=O)C(=O)N[C@@H]([C@@H](C)O)C(=O)N2CCC[C@H]2C(=O)N[C@@H](CC(C)C)C(=O)N[C@H]([C@H](C)O)C(=O)N1
1494	838	C[C@H]1O[C@H]1[C@@H]1C[C@@H]1C1=C(Br)[C@H]2O[C@@H](C[C@H]2O1)C=C=CBr
1495	1618	CC(=O)O[C@H]1[C@@H]2C3=C4C(C)=CC(O)=C5C(=O)OCC(C(O)=C3[C@]11COC(=O)C3=C(O)C=C(C)C(C2=O)=C13)=C45
1496	1554	CCCCC\C=C\C1=C(CO)[C@@H]2OC(C)(C)[C@@H](O)C[C@@]22O[C@H]2[C@@H]1O
1497	1593	NCC[C@H](O)C(=O)N[C@@H]1CC[C@@H](CN)O[C@H]1O[C@@H]1[C@@H](N)C[C@@H](NC(=O)[C@@H](O)CCN)[C@H](O[C@H]2O[C@@H](CO)[C@@H](O)[C@H](N)[C@H]2O)[C@H]1O

1498	1658	N[C@H](CCOC1=CC=C(C=C1)[C@@H](NC=O)C(=O)N[C@H]1CN(C(C([O-])=O)C2=CC=C(O[C@@H]3O[C@@H]([C@@H](O)[C@@H](O)[C@@H]3O)C([O-])=O)C=C2)C1=O)C([O-])=O
1499	1518	NC(=O)NCCC[C@@H](NC(=O)[C@H](CCO)NC(=O)[C@H](CO)NC(=O)[C@H](CCO)NC(=O)[C@H](CC([O-])=O)NC(=O)[C@H]1CCN2C(=O)NC3=C2N1C1=CC(=O)C(O)=CC1=C3)C(=O)N[C@@H](CO)C(=O)NCC(=O)N[C@H]([C@@H](O)C([O-])=O)C([O-])=O
1500	1357	CO[C@H]1O[C@@H](COC(C)=O)[C@@H](OC(C)=O)[C@@H](NC(C)=O)[C@@H]1OC(C)=O
1501	68	CCCCCCCCC[C@H](CCCCCCCC(C)=O)O[C@@H]1O[C@H](CO)[C@@H](O)[C@H](O)[C@H]1O[C@@H]1O[C@H](CO[C@@H]2O[C@H](C)[C@H](O)[C@H](O)[C@@H]2O)[C@@H](OC(C)=O)[C@H](OC(=O)CCC)[C@H]1O[C@@H]1O[C@@H](C)[C@H](O)[C@@H](O)[C@@H]1O
1502	559	CC1=C2C=CC=C(O)C2=C(O)C2=C(O)C3=C(O)C(C(N)=O)=C(O)C(O)=C3C=C12
1503	1599	C[C@]12C[C@@H]3O[C@H]1C[C@@H]3[C@@]1(C)OC(C)(C)[C@H](Br)C[C@H]1O2
1504	1007	CO[C@H]1[C@@H](C[C@H](O)CN)O[C@H]2C[C@H]3O[C@H](C[C@@H](C)C3=C)CC[C@@H]3O[C@H](CC3=C)CC[C@]34C[C@@H]5[C@H](O[C@@H]6[C@@H]5O[C@H]5CC[C@H](CC(=O)C[C@H]12)O[C@@H]5[C@@H]6O3)O4
1505	1610	CC(=O)N[C@@H]1[C@@H](O)[C@H](OS([O-])(=O)=O)[C@@H](CO)O[C@H]1O[C@H]1C[C@H](N[C@H]1CO)C(=O)NCCC([O-])=O
1506	1690	C[C@@H](O)[C@H](NC(=O)[C@@H](C)[C@H](O)[C@H](C)NC(=O)[C@@H](NC(=O)C1=C(C)C(N)=NC(=N1)[C@H](CC(N)=O)NC[C@H](N)C(N)=O)[C@@H](O[C@H]1O[C@H](CO)[C@@H](O)[C@@H](O)[C@@H]1O[C@@H]1O[C@H](CO)[C@@H](O)[C@H](OC(N)=O)[C@H]1O)C1=CNC=N1)C(=O)NCCC1=NC(=CS1)C1=NC(=CS1)C(=O)NCCCNCCCCN
1507	1669	C[C@@H](O)[C@H](NC(=O)[C@@H](C)[C@H](O)[C@@H](C)NC(=O)[C@@H](NC(=O)C1=C(C)C(N)=NC(=N1)[C@H](CC(N)=O)NC[C@H](N)C(N)=O)[C@@H](O[C@@H]1O[C@H](CO)[C@@H](O)[C@H](O)[C@@H]1O[C@@H]1O[C@H](CO)[C@@H](O)[C@H](OC(N)=O)[C@@H]1O)C1=CNC=N1)C(=O)NCCC1=NC(=CS1)C1=NC(=CS1)C(=O)NCCCCNC(N)=N
1508	1621	CN[C@H](CCCNC(=O)C1=CSC(=N1)[C@H]1CSC(CCNC(=O)[C@@H](NC(=O)[C@@H](C)[C@H](O)[C@@H](C)NC(=O)[C@H](NC(=O)C2=C(C)C(N)=NC(=N2)[C@H](CC(N)=O)NC[C@H](N)C(N)=O)[C@@H](O[C@@H]2O[C@H](CO)[C@H](O)[C@H](O)[C@@H]2O[C@@H]2O[C@H](CO)[C@H](O)[C@@H](OC(N)=O)[C@@H]2O)C2=CNC=N2)[C@H](C)O)=N1)CC(=N)NO
1509	1709	C[C@@H](O)[C@H](NC(=O)[C@@H](C)[C@@H](O)[C@@H](C)NC(=O)[C@@H](NC(=O)C1=C(C)C(N)=NC(=N1)[C@H](CC(N)=O)NC[C@H](N)C(N)=O)[C@@H](O[C@@H]1O[C@H](CO)[C@@H](O)[C@H](O)[C@H]1O[C@@H]1O[C@@H](CO)[C@@H](O)[C@@H](OC(N)=O)[C@@H]1O)C1=CNC=N1)C(=O)NCCC1=NC(=CS1)C1=NC(=CS1)C(=O)NCCCCN
1510	1608	C[C@@H](O)[C@H](NC(=O)[C@@H](C)[C@H](O)[C@@H](C)NC(=O)[C@H](NC(=O)C1=NC(=NC(N)=C1C)[C@@H](CC(N)=O)NC[C@H](N)C(N)=O)[C@@H](O[C@H]1O[C@@H](CO)[C@H](O)[C@H](O)[C@@H]1O[C@@H]1O[C@H](CO)[C@@H](O)[C@H](OC(N)=O)[C@@H]1O)C1=CNC=N1)C(=O)NCCC1=NC(=CS1)C1=NC(=CS1)C(N)=O
1511	1665	C[C@@H](O)[C@H](NC(=O)[C@@H](C)[C@H](O)[C@@H](C)NC(=O)[C@H](NC(=O)C1=NC(=NC(N)=C1C)[C@H](CC(N)=O)NC[C@H](N)C(N)=O)[C@@H](O[C@@H]1O[C@H](CO)[C@@H](O)[C@H](O)[C@H]1O[C@H]1O[C@H](CO)[C@H](O)[C@H](OC(N)=O)[C@@H]1O)C1=CNC=N1)C(=O)NCCC1=NC(=CS1)C1=NC(=CS1)C(=O)NCCC[S@@](C)=O

1512	1647	C[C@@H](O)[C@H](NC(=O)[C@@H](C)[C@H](O)[C@@H](C)NC(=O)[C@@H](NC(=O)C1=C(C)C(N)=NC(=N1)[C@H](CC(N)=O)NC[C@@H](N)C(N)=O)[C@@H](O[C@@H]1O[C@H](CO)[C@@H](O)[C@@H](O)[C@@H]1O[C@@H]1O[C@H](CO)[C@@H](O)[C@H](OC(N)=O)[C@@H]1O)C1=CNC=N1)C(=O)NCCC1=NC(=CS1)C1=NC(=CS1)C(=O)NCCCC\N=C(/N)NCCCCNC(N)=N
1513	1649	C[C@@H](O)[C@H](NC(=O)[C@H](C)[C@H](O)[C@@H](C)NC(=O)[C@@H](NC(=O)C1=C(C)C(N)=NC(=N1)[C@H](CC(N)=O)NC[C@H](N)C(N)=O)[C@@H](O[C@@H]1O[C@@H](CO)[C@H](O)[C@@H](O)[C@H]1O[C@H]1O[C@@H](CO)[C@H](O)[C@@H](OC(N)=O)[C@@H]1O)C1=CNC=N1)C(=O)NCCC1=NC(=CS1)C1=NC(=CS1)C(=O)NCCCN
1514	1686	C[C@@H](O)[C@H](NC(=O)[C@@H](C)[C@H](O)[C@H](C)NC(=O)[C@H](NC(=O)C1=NC(=NC(N)=C1C)[C@@H](CC(N)=O)NC[C@H](N)C(N)=O)[C@@H](O[C@@H]1O[C@H](CO)[C@H](O)[C@H](O)[C@H]1O[C@H]1O[C@H](CO)[C@@H](O)[C@@H](OC(N)=O)[C@@H]1O)C1=CNC=N1)C(=O)NCCC1=NC(=CS1)C1=NC(=CS1)C(=O)NCCC1=CNC=N1
1515	1708	CSCCCNC(=O)C1=CSC(=N1)C1=CSC(CCNC(=O)[C@@H](NC(=O)[C@@H](C)[C@H](O)[C@@H](C)NC(=O)[C@@H](NC(=O)C2=NC(=NC(N)=C2C)[C@H](CC(N)=O)NC[C@H](N)C(N)=O)[C@@H](O[C@@H]2O[C@H](CO)[C@H](O)[C@H](O)[C@H]2O[C@@H]2O[C@H](CO)[C@H](O)[C@H](OC(N)=O)[C@@H]2O)C2=CNC=N2)[C@@H](C)O)=N1
1516	191	C[C@H](O)[C@H](NC(=O)[C@@H](C)[C@H](O)[C@@H](C)NC(=O)[C@H](NC(=O)C1=C(C)C(N)=NC(=N1)[C@H](CC(N)=O)NC[C@H](N)C(N)=O)[C@@H](O[C@H]1O[C@@H](CO)[C@H](O)[C@H](O)[C@H]1O[C@@H]1O[C@@H](CO)[C@H](O)[C@H](OC(N)=O)[C@@H]1O)C1=CNC=N1)C(=O)NCCC1=N[C@H](CS1)C1=NC(=CS1)C(=O)NCCC(N)=N
1517	1688	C[C@@H](O)[C@H](NC(=O)[C@@H](C)[C@H](O)[C@H](C)NC(=O)[C@@H](NC(=O)C1=C(C)C(N)=NC(=N1)[C@H](CC(N)=O)NC[C@H](N)C(N)=O)[C@@H](O[C@H]1O[C@H](CO)[C@@H](O)[C@H](O)[C@@H]1O[C@@H]1O[C@@H](CO)[C@H](O)[C@@H](OC(N)=O)[C@@H]1O)C1=CNC=N1)C(=O)NCCC1=NC(=CS1)C1=NC(=CS1)C(=O)NCCCNCCCCNCCCN

Table S2. Complete results of the three filters by color code (green = good; red = bad).

BDB ID	2D p*K*i	2DApplicability	3D p*K*i	3D Applicability	Docking p*K*i	Mean	nM
1169	10.06	No	7.4	Excellent	10.26	9.24	0.6
28	9.90	No	7.2	Excellent	9.64	8.91	1.2
45	9.86	No	7.4	Excellent	9.42	8.89	1.3
1172	9.46	No	7.9	Excellent	9.31	8.89	1.3
1421	8.90	No	7.4	Excellent	9.79	8.70	2.0
246	10.59	No	7.3	Excellent	7.91	8.60	2.5
14	9.91	No	7.1	Excellent	8.71	8.57	2.7
298	9.73	No	7.8	Excellent	8.11	8.55	2.8
798	8.88	No	7.7	Excellent	9.05	8.54	2.9
984	8.25	No	7.3	Excellent	9.77	8.44	3.6
1179	9.53	No	7.3	Excellent	8.49	8.44	3.6
848	9.08	No	8.6	Excellent	7.62	8.43	3.7
1333	8.35	No	7.4	Excellent	9.45	8.40	4.0
420	10.15	No	7.5	Excellent	7.35	8.33	4.6
272	9.63	No	7	Excellent	8.19	8.27	5.3

84	7.70	No	8	Excellent	9.03	8.24
1048	9.03	No	7.1	Excellent	8.55	8.23
914	9.58	No	7.3	Excellent	7.75	8.21
1232	9.02	No	7.6	Excellent	7.98	8.20
1223	8.36	No	7.2	Excellent	8.95	8.17
302	8.90	No	7.6	Excellent	7.90	8.13
640	8.84	No	7.1	Excellent	8.43	8.12
981	7.88	No	7.9	Excellent	8.53	8.10
1273	9.76	No	7.5	Excellent	7.00	8.09
258	9.32	No	7	Excellent	7.93	8.08
1190	10.41	No	7	Excellent	6.77	8.06
670	8.32	No	8.3	Excellent	7.52	8.05
456	8.97	No	7.2	Excellent	7.93	8.04
279	7.51	No	7.4	Excellent	9.18	8.03
841	8.82	No	7.2	Excellent	8.07	8.03
903	8.74	No	7.5	Excellent	7.84	8.03
1158	9.01	No	7.3	Excellent	7.71	8.01
1197	9.32	No	7.9	Excellent	6.77	8.00
1087	8.55	No	7.6	Excellent	7.73	7.96
434	9.17	No	7.3	Excellent	7.40	7.96
99	9.63	No	7.5	Excellent	6.74	7.96
1051	7.86	No	7.1	Excellent	8.89	7.95
933	7.82	YES	8.1	Excellent	7.88	7.93
245	8.86	No	7.8	Excellent	7.13	7.93
524	7.22	No	7.2	Excellent	9.31	7.91
122	8.90	YES	7	Excellent	7.81	7.90
827	7.86	No	7.2	Excellent	8.63	7.90
306	6.68	No	7.4	Excellent	9.60	7.89
1114	8.05	No	7.2	Excellent	8.42	7.89
95	8.94	No	7.9	Excellent	6.65	7.83
1049	7.67	No	7	Excellent	8.81	7.83
1235	7.85	No	7	Excellent	8.61	7.82
170	7.10	No	7.5	Excellent	8.81	7.80
257	8.07	No	7.4	Excellent	7.93	7.80
820	8.28	YES	6.9	Excellent	8.21	7.80
1429	7.91	No	7.1	Excellent	8.36	7.79
1228	7.41	No	7	Excellent	8.91	7.77
151	8.66	No	7.1	Excellent	7.54	7.77
1474	8.68	No	7.7	Excellent	6.93	7.77
1299	9.61	No	7.2	Excellent	6.40	7.74
242	8.22	No	7.2	Excellent	7.79	7.73
348	8.62	No	7.7	Excellent	6.89	7.73
696	7.07	No	7.3	Excellent	8.83	7.73

1123	6.54	No	7.4	Excellent	9.23	7.72
734	8.08	No	7	Excellent	7.98	7.69
850	8.11	No	7	Excellent	7.93	7.68
625	8.12	No	7.8	Excellent	7.04	7.65
761	6.47	No	7.8	Excellent	8.64	7.64
671	6.89	No	7.3	Excellent	8.70	7.63
1386	7.96	No	7.5	Excellent	7.42	7.63
1400	7.52	No	7.4	Excellent	7.91	7.61
1009	7.97	No	7	Excellent	7.86	7.61
570	8.13	No	7.2	Excellent	7.47	7.60
55	7.89	No	8.2	Excellent	6.70	7.60
1471	8.77	No	7.1	Excellent	6.90	7.59
1546	7.54	No	7.7	Excellent	7.52	7.59
1036	8.29	No	7	Excellent	7.42	7.57
684	7.49	No	7.5	Excellent	7.67	7.55
1094	8.73	No	7.2	Excellent	6.71	7.55
975	7.38	No	7.5	Excellent	7.74	7.54
1034	7.49	No	8	Excellent	7.09	7.53
1496	8.16	No	7.3	Excellent	7.11	7.52
1262	7.06	YES	7.1	Excellent	8.41	7.52
916	8.59	No	7.2	Excellent	6.76	7.52
793	8.34	No	7.2	Excellent	7.02	7.52
854	8.38	No	7.1	Excellent	7.07	7.52
1112	9.19	No	7.2	Excellent	6.15	7.51
1215	9.10	No	7.2	Excellent	6.23	7.51
1300	8.25	No	7.1	Excellent	7.17	7.51
1329	7.36	No	7.1	Excellent	8.06	7.51
1225	7.57	YES	7.6	Excellent	7.34	7.50
1436	6.56	No	7.6	Excellent	8.34	7.50
85	7.06	YES	7.2	Excellent	8.22	7.49
594	7.80	No	7.3	Excellent	7.36	7.49
1037	8.01	No	7.2	Excellent	7.25	7.49
1245	8.01	YES	6.9	Good	7.51	7.47
1265	8.58	No	7.6	Excellent	6.23	7.47
1581	5.36	No	7.9	Excellent	9.14	7.47
1133	8.32	No	7	Excellent	7.07	7.46
1428	8.53	No	7.8	Excellent	6.06	7.46
939	7.63	No	7.2	Excellent	7.55	7.46
1455	7.74	YES	7.5	Bad	7.12	7.45
653	8.25	No	7.5	Excellent	6.58	7.44
352	7.21	No	7.6	Excellent	7.52	7.44
353	7.01	No	7.9	Excellent	7.39	7.43
538	6.41	No	7.4	Excellent	8.48	7.43

369	6.99	No	7.6	Excellent	7.69	7.43
796	6.10	No	7.9	Excellent	8.27	7.43
962	6.25	No	7.6	Excellent	8.41	7.42
1121	6.43	No	7.3	Excellent	8.51	7.41
1565	7.59	YES	7.2	Excellent	7.42	7.40
846	7.97	YES	6.9	Excellent	7.33	7.40
529	6.13	No	7.1	Excellent	8.95	7.39
1437	8.22	No	7.8	Excellent	6.14	7.39
1569	6.70	No	7.1	Excellent	8.35	7.38
1144	6.05	No	8.3	Excellent	7.80	7.38
20	7.89	No	7.1	Excellent	7.16	7.38
355	7.51	No	7.4	Excellent	7.22	7.38
293	7.01	No	7.6	Excellent	7.52	7.37
1152	7.30	YES	7.3	Excellent	7.51	7.37
621	5.61	No	10.2	Excellent	6.30	7.37
508	6.54	No	7.4	Excellent	8.15	7.36
488	6.20	No	7.3	Excellent	8.58	7.36
1154	7.81	No	7.1	Excellent	7.16	7.36
710	7.31	YES	7.4	Excellent	7.36	7.36
175	7.31	No	7.1	Excellent	7.65	7.35
929	6.30	No	7.3	Excellent	8.42	7.34
133	8.67	No	7.4	Excellent	5.94	7.34
1549	6.27	No	7.6	Excellent	8.13	7.33
1058	6.15	No	8	Excellent	7.85	7.33
265	6.87	YES	7.5	Excellent	7.61	7.33
354	7.15	No	7.4	Excellent	7.43	7.33
1137	9.06	No	7.2	Excellent	5.72	7.33
1018	6.54	No	8	Excellent	7.41	7.32
1318	7.11	No	7.1	Excellent	7.72	7.31
1118	7.49	No	7	Excellent	7.44	7.31
688	7.08	YES	7.1	Good	7.72	7.30
495	5.88	No	7.5	Excellent	8.52	7.30
580	6.68	No	7.9	Excellent	7.30	7.29
853	6.60	No	7.2	Excellent	8.08	7.29
790	6.81	No	7	Excellent	8.06	7.29
164	8.30	No	7.1	Excellent	6.47	7.29
1380	7.41	No	7.2	Excellent	7.25	7.29
956	7.28	No	7.2	Excellent	7.38	7.29
374	7.02	No	7	Excellent	7.84	7.28
1242	6.85	YES	7.5	Excellent	7.49	7.28
643	7.52	No	7	Excellent	7.29	7.27
1645	7.65	No	7.9	Excellent	6.25	7.27
1478	5.51	No	7.4	Excellent	8.88	7.26

1534	9.40	No	7.1	Excellent	5.28	7.26
722	6.70	No	7.5	Excellent	7.57	7.26
234	8.71	No	7	Excellent	6.01	7.24
167	5.97	YES	7.1	Excellent	8.63	7.23
79	8.20	YES	6.6	Excellent	6.89	7.23
1521	8.13	YES	7.2	Excellent	6.34	7.22
1453	8.30	No	8	Excellent	5.34	7.21
1259	7.27	No	7	Excellent	7.35	7.21
783	6.83	No	7.7	Excellent	7.08	7.20
395	6.92	YES	7.3	Excellent	7.39	7.20
1514	6.38	YES	7.1	Excellent	8.12	7.20
557	7.13	No	7.1	Excellent	7.37	7.20
639	6.93	No	7.1	Excellent	7.56	7.20
1612	4.71	No	9	Excellent	7.88	7.20
1350	7.85	No	7	Excellent	6.73	7.19
1267	7.01	YES	7.1	Excellent	7.43	7.18
1364	6.50	No	7.2	Excellent	7.81	7.17
682	6.76	No	7.6	Excellent	7.14	7.17
1449	7.03	No	7.1	Excellent	7.35	7.16
598	6.40	No	7.5	Excellent	7.58	7.16
1439	6.59	No	7.9	Excellent	6.99	7.16
292	6.66	No	7.3	Excellent	7.52	7.16
558	6.18	No	7.2	Excellent	8.09	7.16
875	7.18	YES	7.3	OK	6.98	7.15
995	6.86	YES	7	Excellent	7.58	7.15
1170	6.31	No	7.5	Excellent	7.63	7.15
1039	7.11	No	7.2	Excellent	7.10	7.14
1289	7.23	No	7	Excellent	7.18	7.14
1464	7.38	No	7.9	Excellent	6.11	7.13
1467	6.11	No	7.1	Excellent	8.18	7.13
839	6.99	No	7	Excellent	7.39	7.13
1098	6.81	No	7	Excellent	7.58	7.13
1370	6.41	No	7.2	Excellent	7.78	7.13
1542	6.90	No	7.4	Excellent	7.06	7.12
996	6.62	No	7.5	Excellent	7.24	7.12
1174	6.84	No	7	Excellent	7.49	7.11
1410	6.46	No	7.1	Excellent	7.76	7.11
515	7.59	No	7.2	Excellent	6.52	7.10
651	7.13	No	7.2	Excellent	6.97	7.10
390	5.98	No	7.3	Excellent	8.02	7.10
235	7.09	No	7.1	Excellent	7.10	7.10
1201	7.07	No	7.2	Excellent	7.01	7.09
1490	6.82	YES	7.2	Excellent	7.26	7.09

1414	6.80	No	7.1	Excellent	7.36	7.09
1473	6.72	No	7.2	Excellent	7.34	7.08
957	6.28	No	7	Excellent	7.97	7.08
190	6.29	No	7	Excellent	7.96	7.08
1678	6.56	No	7.7	Excellent	6.98	7.08
794	7.33	YES	6.6	Excellent	7.31	7.08
889	6.74	No	7	Excellent	7.48	7.08
917	8.13	No	7.8	Excellent	5.28	7.07
511	7.63	No	7.2	Excellent	6.38	7.07
1266	6.53	YES	7.3	Excellent	7.35	7.06
567	6.27	No	8	Excellent	6.87	7.05
923	7.43	YES	6.9	Good	6.81	7.05
885	6.80	YES	7.1	Excellent	7.22	7.04
802	7.34	YES	6.8	Good	6.98	7.04
1064	7.04	YES	7.1	Good	6.97	7.04
763	6.24	No	7.3	Excellent	7.55	7.03
496	6.39	No	7.2	Excellent	7.50	7.03
950	7.27	YES	6.6	Excellent	7.22	7.03
1544	6.61	No	7.4	Excellent	7.06	7.03
955	8.17	No	7	Excellent	5.90	7.02
977	6.99	No	7.1	Excellent	6.98	7.02
1145	8.25	No	7.2	Excellent	5.61	7.02
423	7.71	YES	7	Excellent	6.30	7.00
1001	6.54	No	7.1	Excellent	7.37	7.00
393	5.90	No	7.9	Excellent	7.20	7.00
1089	5.79	No	7.2	Excellent	8.01	7.00
810	7.08	YES	7	Good	6.89	6.99
1584	6.42	No	7.1	Excellent	7.45	6.99
1361	5.97	No	7.4	Excellent	7.59	6.99
1135	6.53	No	7	Excellent	7.43	6.99
1006	5.50	No	7	Excellent	8.43	6.98
1399	6.46	No	7.5	Excellent	6.96	6.97
1684	6.14	No	7.8	Excellent	6.98	6.97
1426	6.28	YES	8.1	Excellent	6.53	6.97
294	6.86	No	7.1	Excellent	6.92	6.96
1309	5.92	No	7.3	Excellent	7.66	6.96
1003	7.42	YES	6.3	Excellent	7.16	6.96
1384	7.13	No	7	Excellent	6.73	6.95
539	7.84	YES	7.3	Excellent	5.69	6.94
318	6.63	YES	8	Excellent	6.20	6.94
603	6.08	No	7.1	Excellent	7.65	6.94
211	6.19	YES	7	Excellent	7.61	6.93
50	6.31	YES	7.4	Excellent	7.07	6.93

4	6.96	No	7	Excellent	6.83	6.93
490	7.31	YES	6.6	OK	6.87	6.93
609	7.26	YES	7	Poor	6.52	6.93
787	6.50	YES	7	Excellent	7.28	6.92
157	6.01	No	7.3	Excellent	7.47	6.92
1382	4.80	No	7.9	Excellent	8.07	6.92
1457	6.07	No	7.1	Excellent	7.59	6.92
895	7.67	No	7.3	Excellent	5.79	6.92
657	7.95	No	7.3	Excellent	5.48	6.91
210	7.32	YES	6.1	Excellent	7.30	6.91
1045	5.78	No	7.2	Excellent	7.74	6.91
991	6.80	No	7	Excellent	6.91	6.90
554	5.80	No	7.2	Excellent	7.70	6.90
620	5.47	No	7.3	Excellent	7.91	6.89
553	7.09	YES	6.6	Good	6.98	6.89
149	8.15	YES	6.3	Excellent	6.21	6.89
65	6.73	No	7.4	Excellent	6.52	6.88
756	6.89	YES	7.1	Excellent	6.65	6.88
945	6.57	No	6.9	Excellent	7.15	6.87
1703	6.12	No	8.3	Excellent	6.20	6.87
1557	6.08	No	7.5	Excellent	7.04	6.87
1517	6.14	YES	7.1	Excellent	7.36	6.87
601	6.11	YES	7.2	Excellent	7.29	6.87
858	7.32	No	7.3	Excellent	5.97	6.86
1452	7.05	YES	6.7	Excellent	6.81	6.85
1230	6.46	YES	7	Excellent	7.08	6.85
1186	7.55	No	7	Excellent	5.99	6.85
727	6.04	YES	7.4	Excellent	7.08	6.84
445	6.24	No	7.1	Excellent	7.17	6.84
582	7.68	No	7.1	Excellent	5.73	6.84
78	6.50	No	7.1	Excellent	6.89	6.83
1697	6.46	No	7.2	Excellent	6.83	6.83
159	7.82	YES	7	Excellent	5.64	6.82
823	8.43	YES	6	Excellent	6.03	6.82
441	7.15	YES	6.5	Excellent	6.81	6.82
818	5.74	No	7	Excellent	7.72	6.82
972	5.87	No	8.2	Excellent	6.39	6.82
454	6.54	No	7.6	Excellent	6.32	6.82
1371	6.30	No	7.1	Excellent	7.04	6.81
630	7.29	YES	6.1	Excellent	7.05	6.81
532	5.99	YES	7.1	Excellent	7.34	6.81
751	5.45	No	7.3	Excellent	7.67	6.81
656	6.85	No	7.3	Excellent	6.26	6.80

1351	6.29	YES	7.5	Excellent	6.61	6.80
847	5.01	No	7.9	Excellent	7.47	6.80
1605	6.42	No	7.7	Excellent	6.26	6.79
1111	6.27	No	7.2	Excellent	6.90	6.79
1209	6.54	No	7	Excellent	6.83	6.79
998	7.25	YES	6.5	Excellent	6.61	6.79
1334	5.46	No	7	Excellent	7.90	6.79
1188	5.78	No	7.1	Excellent	7.47	6.78
855	6.03	YES	7.7	Excellent	6.61	6.78
47	6.64	No	7.3	Excellent	6.39	6.78
1014	6.42	No	7.1	Excellent	6.80	6.77
1081	5.54	No	7.2	Excellent	7.53	6.76
51	6.79	No	7.2	Excellent	6.29	6.76
1017	6.33	No	7	Excellent	6.93	6.75
1284	6.20	No	7.3	Excellent	6.76	6.75
1035	5.60	No	7.4	Excellent	7.26	6.75
194	5.70	No	7.3	Excellent	7.26	6.75
317	6.16	No	7.6	Excellent	6.49	6.75
310	5.49	No	7	Excellent	7.76	6.75
1303	6.03	No	7	Excellent	7.21	6.75
440	6.55	No	7.1	Excellent	6.58	6.74
976	6.56	No	7.5	Excellent	6.16	6.74
296	5.61	No	7.1	Excellent	7.51	6.74
593	6.17	No	7.4	Excellent	6.65	6.74
577	6.35	No	7.3	Excellent	6.55	6.73
1221	6.63	No	7	Excellent	6.56	6.73
223	5.56	No	7.7	Excellent	6.93	6.73
394	7.13	No	7.2	Excellent	5.85	6.73
1342	6.00	No	8.3	Excellent	5.86	6.72
1604	7.13	No	7.1	Excellent	5.92	6.72
1271	5.28	No	7.4	Excellent	7.45	6.71
121	6.04	No	7.2	Excellent	6.88	6.71
1277	6.07	No	7.2	Excellent	6.83	6.70
331	5.16	No	7	Excellent	7.93	6.70
564	5.82	No	7.1	Excellent	7.16	6.69
673	7.30	YES	6.2	Good	6.58	6.69
1275	5.70	No	7.2	Excellent	7.15	6.68
738	6.14	No	7	Excellent	6.91	6.68
566	4.77	No	7.3	Excellent	7.97	6.68
1338	6.73	No	7.3	Excellent	6.00	6.68
91	6.72	YES	7.2	Excellent	6.11	6.68
297	5.47	No	7.4	Excellent	7.16	6.67
1088	6.02	No	7.4	Excellent	6.59	6.67

270	5.93	No	7.1	Excellent	6.97	6.67
326	5.28	No	7.2	Excellent	7.52	6.67
6	5.65	No	7.2	Excellent	7.14	6.66
1409	6.84	YES	7	Excellent	6.15	6.66
1653	6.81	No	7.2	Excellent	5.97	6.66
468	5.41	YES	7.4	Excellent	7.17	6.66
1353	7.00	YES	6.2	Excellent	6.77	6.66
568	4.53	No	7.2	Excellent	8.22	6.65
1099	5.78	YES	7.2	Excellent	6.96	6.65
866	5.97	No	7	Excellent	6.97	6.65
74	6.35	YES	7	Excellent	6.56	6.64
383	5.26	No	7.3	Excellent	7.33	6.63
1349	5.13	No	7.1	Excellent	7.66	6.63
719	5.68	No	7.1	Excellent	7.09	6.62
687	6.16	No	7.3	Excellent	6.38	6.62
1194	5.97	No	7.4	Excellent	6.48	6.62
1324	6.69	YES	7.7	Excellent	5.45	6.61
1180	6.19	No	7.2	Excellent	6.45	6.61
1129	5.51	YES	7.4	Excellent	6.91	6.61
1085	6.10	No	7.3	Excellent	6.42	6.61
891	6.03	No	7	Excellent	6.79	6.61
1345	6.72	No	7.6	Excellent	5.47	6.60
1288	6.07	No	7	Excellent	6.71	6.59
411	6.51	No	7	Excellent	6.27	6.59
1227	5.94	No	7.1	Excellent	6.72	6.59
901	5.73	YES	7.2	Excellent	6.83	6.59
1002	6.22	No	7.3	Excellent	6.24	6.59
1238	6.20	No	7.6	Excellent	5.95	6.58
1344	5.93	No	7.4	Excellent	6.42	6.58
645	5.78	No	7.3	Excellent	6.64	6.57
1479	6.27	YES	7.2	Excellent	6.23	6.57
747	7.01	No	7.1	Excellent	5.58	6.56
944	5.68	No	7	Excellent	7.00	6.56
865	5.63	No	7	Excellent	7.03	6.56
314	6.70	No	7.1	Excellent	5.85	6.55
1331	5.73	No	7.4	Excellent	6.51	6.55
1319	5.80	No	7.4	Excellent	6.43	6.54
971	6.50	No	7.1	Excellent	6.01	6.54
1559	6.38	No	7	Excellent	6.23	6.54
449	5.73	No	7.3	Excellent	6.56	6.53
410	5.24	No	7.1	Excellent	7.22	6.52
17	6.72	No	7	Excellent	5.84	6.52
800	4.96	No	7.1	Excellent	7.47	6.51

438	5.28	No	7	Excellent	7.25	6.51
1574	6.01	No	7.3	Excellent	6.22	6.51
1360	4.57	No	8	Excellent	6.95	6.51
705	4.28	No	7.9	Excellent	7.31	6.50
130	6.01	No	7.2	Excellent	6.28	6.50
1224	7.27	No	7.1	Excellent	5.10	6.49
666	5.58	No	7.2	Excellent	6.68	6.49
443	6.43	No	7	Excellent	6.02	6.48
769	6.07	No	7.1	Excellent	6.25	6.47
791	4.94	No	7.1	Excellent	7.35	6.46
1682	3.41	No	7.1	Excellent	8.88	6.46
1166	4.77	No	7.2	Excellent	7.39	6.45
1189	5.64	No	7.4	Excellent	6.30	6.45
1029	5.12	No	7	Excellent	7.22	6.45
1263	6.24	YES	7.3	Excellent	5.79	6.44
1124	5.89	No	8	Excellent	5.42	6.44
1450	5.14	No	7	Excellent	7.15	6.43
1068	5.90	No	7.1	Excellent	6.29	6.43
1485	5.69	YES	7.3	Excellent	6.30	6.43
138	6.63	No	7.1	Excellent	5.55	6.43
1411	6.17	No	7	Excellent	6.10	6.42
200	8.58	YES	7.3	Good	3.38	6.42
1638	5.26	No	7.4	Excellent	6.60	6.42
1715	5.61	No	7	Excellent	6.65	6.42
111	6.03	No	7	Excellent	6.21	6.42
986	4.60	No	7.1	Excellent	7.54	6.41
1469	4.28	No	7.2	Excellent	7.75	6.41
1404	6.72	No	7	Excellent	5.51	6.41
1071	6.83	YES	7	Excellent	5.39	6.41
669	5.27	No	7.2	Excellent	6.73	6.40
708	5.87	No	7	Excellent	6.32	6.40
1476	6.03	No	7.1	Excellent	6.06	6.40
387	5.72	No	7.6	Excellent	5.86	6.39
1020	2.62	No	7.8	Excellent	8.76	6.39
216	4.98	No	7.2	Excellent	6.98	6.39
555	5.29	YES	6.9	Excellent	6.94	6.38
174	5.20	No	7.5	Excellent	6.43	6.38
253	5.46	YES	7.2	Excellent	6.46	6.37
1579	5.06	No	7.6	Excellent	6.44	6.37
140	5.33	No	7.4	Excellent	6.35	6.36
578	5.87	No	7.3	Excellent	5.91	6.36
21	5.43	No	7.4	Excellent	6.24	6.36
368	5.79	YES	7.1	Excellent	6.17	6.35

1080	5.87	YES	7.4	Excellent	5.79	6.35
1716	6.07	No	7	Excellent	5.97	6.35
1274	6.32	No	7	Excellent	5.71	6.34
1127	5.71	No	7.6	Excellent	5.72	6.34
362	6.34	No	7.1	Excellent	5.59	6.34
1672	5.38	No	7.2	Excellent	6.43	6.34
379	4.63	No	7	Excellent	7.37	6.33
744	6.47	No	7.1	Excellent	5.42	6.33
556	6.80	No	7	Excellent	5.19	6.33
1606	5.36	No	7.9	Excellent	5.70	6.32
974	5.50	No	7.8	Excellent	5.66	6.32
389	5.66	YES	7.4	Excellent	5.89	6.32
180	6.53	No	7	Excellent	5.42	6.32
1468	5.57	YES	7.2	Excellent	6.18	6.31
828	7.75	YES	6.2	Excellent	4.96	6.30
1092	6.27	No	7.2	Excellent	5.44	6.30
547	5.25	YES	7.2	Excellent	6.44	6.30
739	5.76	YES	7.2	Excellent	5.84	6.27
182	6.51	No	7	Excellent	5.28	6.26
759	5.86	No	7.6	Excellent	5.33	6.26
712	5.31	YES	7.2	Excellent	6.26	6.26
967	6.46	No	7.2	Excellent	5.10	6.25
697	5.35	YES	7.2	Excellent	6.20	6.25
503	6.29	No	7.2	Excellent	5.24	6.24
356	6.25	No	8	Excellent	4.48	6.24
652	5.18	No	7.2	Excellent	6.34	6.24
500	5.73	YES	7	Excellent	5.95	6.23
455	5.91	YES	7.1	Excellent	5.66	6.22
1448	6.36	No	7.2	Excellent	5.08	6.21
311	5.74	No	7.1	Excellent	5.78	6.21
777	4.83	No	7.4	Excellent	6.37	6.20
414	3.39	No	8.1	Excellent	7.10	6.20
1582	5.55	No	7.1	Excellent	5.91	6.19
516	5.25	No	7.1	Excellent	6.20	6.19
412	10.94	No	7.6	Good		6.18
525	5.66	YES	7	Excellent	5.87	6.18
261	5.40	YES	7.2	Excellent	5.92	6.17
1176	5.43	YES	7	Excellent	6.09	6.17
720	5.56	YES	7.1	Excellent	5.84	6.17
921	5.75	YES	7	Excellent	5.71	6.15
980	4.81	No	7.2	Excellent	6.45	6.15
145	5.38	No	7.1	Excellent	5.97	6.15
576	4.54	No	7.2	Excellent	6.71	6.15

683	4.76	No	7.6	Excellent	6.08	6.15
871	5.19	No	7.1	Excellent	6.15	6.15
71	4.99	No	7.1	Excellent	6.35	6.15
1657	5.32	No	7.5	Excellent	5.60	6.14
1513	5.50	No	7.1	Excellent	5.80	6.13
605	4.30	No	7.8	Excellent	6.29	6.13
1362	5.46	No	7	Excellent	5.92	6.13
884	5.34	YES	7.1	Excellent	5.92	6.12
137	5.40	YES	7.3	Excellent	5.60	6.10
616	4.42	No	7.7	Excellent	6.17	6.10
337	5.15	YES	7.5	Excellent	5.62	6.09
1109	6.41	YES	7.4	Excellent	4.46	6.09
544	4.97	No	7.5	Excellent	5.79	6.09
1365	5.12	No	7.1	Excellent	6.00	6.07
535	5.95	No	7.2	Excellent	5.01	6.05
380	4.96	No	7.2	Excellent	5.97	6.04
1302	4.95	YES	7.5	Excellent	5.68	6.04
3	5.49	No	7.2	Excellent	5.44	6.04
745	5.63	No	7.1	Excellent	5.39	6.04
940	5.02	No	7.1	Excellent	5.96	6.03
1378	4.79	No	7.2	Excellent	6.06	6.02
295	5.08	No	7	Excellent	5.95	6.01
514	5.03	No	7.7	Excellent	5.27	6.00
540	6.07	No	7.4	Excellent	4.51	5.99
341	5.54	YES	7.1	Excellent	5.32	5.98
18	5.85	No	7.6	Excellent	4.49	5.98
1030	5.05	YES	7.1	Excellent	5.78	5.98
1425	5.08	No	7	Excellent	5.80	5.96
1306	4.04	No	7	Excellent	6.84	5.96
1547	4.77	No	7.8	Excellent	5.30	5.96
1495	3.95	No	7.5	Excellent	6.40	5.95
1254	4.95	No	7.1	Excellent	5.80	5.95
888	5.10	No	7	Excellent	5.75	5.95
928	4.39	No	7.3	Excellent	6.14	5.94
1520	4.31	No	7.3	Excellent	6.19	5.93
1339	9.79	No	8	Poor		5.93
526	5.19	YES	7.2	Excellent	5.38	5.93
642	10.98	No	6.8	Excellent		5.93
1402	10.94	No	6.8	Good		5.91
367	5.08	No	7.3	Excellent	5.33	5.90
1205	10.89	No	6.8	Excellent		5.90
947	5.36	No	7.6	Excellent	4.72	5.89
409	4.73	No	7.1	Excellent	5.80	5.88

728	5.64	No	7.4	Excellent	4.58	5.87
1646	3.70	No	7	Excellent	6.86	5.85
1408	4.49	No	7.3	Excellent	5.75	5.85
205	4.21	No	7	Excellent	6.33	5.85
494	4.66	No	7	Excellent	5.84	5.83
1191	5.88	No	7.2	Excellent	4.40	5.83
1609	9.67	No	7.8	Good		5.82
120	4.63	No	8.1	Excellent	4.70	5.81
1713	10.78	No	6.6	Good		5.79
1607	8.97	No	8.4	Poor		5.79
1178	3.90	No	7.4	Excellent	6.00	5.77
1301	5.27	No	7.5	Excellent	4.48	5.75
715	5.39	No	7.2	Excellent	4.64	5.75
860	9.93	No	7.3	Good		5.74
134	5.52	No	7.2	Excellent	4.50	5.74
1463	9.61	No	7.6	Bad		5.74
466	3.90	No	7.1	Excellent	6.17	5.72
12	4.00	No	7.5	Excellent	5.65	5.72
2	4.85	No	7.4	Excellent	4.88	5.71
805	9.39	No	7.7	OK		5.70
788	3.71	No	7.2	Excellent	6.14	5.69
840	8.62	No	8.4	OK		5.67
1454	10.00	No	7	Bad		5.67
1050	5.03	No	7.1	Excellent	4.85	5.66
859	3.02	No	7.1	Excellent	6.85	5.66
1486	4.10	No	7.3	Excellent	5.55	5.65
753	5.03	No	7	Excellent	4.90	5.65
522	5.43	YES	7.1	Excellent	4.39	5.64
646	10.07	No	6.8	Excellent		5.62
244	6.10	YES	7.1	Excellent	3.67	5.62
925	5.11	No	7.4	Excellent	4.35	5.62
732	4.33	No	7.1	Excellent	5.41	5.61
339	3.88	No	7.3	Excellent	5.67	5.61
1403	9.53	No	7.3	Good		5.61
1113	4.50	YES	7.4	Excellent	4.89	5.60
127	5.01	No	7	Excellent	4.78	5.60
1065	9.48	No	7.3	Good		5.59
1033	8.77	No	8	Poor		5.59
1155	9.53	No	7.2	Good		5.58
472	8.62	No	8.1	Good		5.57
283	9.97	No	6.7	OK		5.56
1375	9.93	No	6.7	Good		5.54
166	4.93	YES	7.3	Excellent	4.38	5.54

1206	9.90	No	6.7	Bad		5.53
358	9.28	No	7.3	Poor		5.53
1545	3.73	No	7.6	Excellent	5.23	5.52
327	9.72	No	6.8	Excellent		5.51
546	9.77	No	6.7	Excellent		5.49
181	3.54	No	7.4	Excellent	5.52	5.49
1571	9.51	No	6.9	Poor		5.47
1625	8.81	No	7.6	Poor		5.47
22	7.36	No	9	Poor		5.45
1368	9.55	No	6.8	Excellent		5.45
1596	9.04	No	7.3	OK		5.45
1648	9.12	No	7.2	Poor		5.44
520	9.31	No	7	OK		5.44
581	4.85	No	7.1	Excellent	4.35	5.44
1487	8.89	No	7.4	Good		5.43
1652	8.57	No	7.7	Bad		5.42
1499	4.06	No	7.5	Excellent	4.69	5.42
1231	9.21	No	7	Poor		5.40
287	8.80	No	7.4	OK		5.40
90	8.12	No	8	Bad		5.37
1692	8.60	No	7.5	Poor		5.37
225	9.28	No	6.8	Good		5.36
899	3.44	YES	7	Excellent	5.62	5.35
1683	9.62	No	6.4	Good		5.34
444	9.44	No	6.5	Excellent		5.31
530	3.39	No	7.4	Excellent	5.12	5.30
474	9.39	No	6.5	OK		5.30
597	9.77	No	6.1	Excellent		5.29
1507	9.16	No	6.7	Excellent		5.29
1561	9.36	No	6.5	OK		5.29
1572	8.05	No	7.8	Poor		5.28
815	9.41	No	6.4	Good		5.27
1714	9.10	No	6.7	Bad		5.27
1247	9.16	No	6.6	Excellent		5.25
968	7.25	No	8.5	Good		5.25
648	8.14	No	7.6	Bad		5.25
1696	8.52	No	7.2	Good		5.24
637	9.11	No	6.6	Good		5.24
42	5.42	YES	7	Excellent	3.30	5.24
863	8.99	No	6.7	Good		5.23
689	8.78	No	6.9	Excellent		5.23
1509	8.46	No	7.2	OK		5.22
1012	9.45	No	6.2	Excellent		5.22

271	8.83	No	6.8	Good		5.21
1359	7.58	No	8	Good		5.19
1063	9.28	No	6.3	OK		5.19
1165	3.85	No	7.1	Excellent	4.59	5.18
938	8.91	No	6.6	Excellent		5.17
918	8.81	No	6.7	Excellent		5.17
1578	8.80	No	6.7	Excellent		5.17
1325	8.69	No	6.8	Excellent		5.16
1642	8.58	No	6.9	Excellent		5.16
57	9.08	No	6.4	Excellent		5.16
1435	8.36	No	7.1	Poor		5.15
477	8.95	No	6.5	Good		5.15
252	8.94	No	6.5	Excellent		5.15
60	8.53	No	6.9	Excellent		5.14
229	8.82	No	6.6	Excellent		5.14
892	7.72	No	7.7	Good		5.14
635	8.90	No	6.5	Excellent		5.13
83	8.70	No	6.7	Excellent		5.13
1070	8.90	No	6.5	Good		5.13
1076	9.47	No	5.9	Poor		5.12
1635	7.83	No	7.5	Good		5.11
1366	8.51	No	6.8	Poor		5.10
1598	7.79	No	7.5	Good		5.10
537	5.11	YES	7	Excellent	3.17	5.09
1675	7.67	No	7.6	Bad		5.09
1570	8.66	No	6.6	Excellent		5.09
66	7.85	No	7.4	Good		5.08
1466	8.63	No	6.6	Excellent		5.08
1533	9.01	No	6.2	Poor		5.07
27	7.91	No	7.3	Poor		5.07
195	8.50	No	6.7	Excellent		5.07
1219	7.50	No	7.7	Bad		5.07
94	8.57	No	6.6	Excellent		5.06
1667	8.86	No	6.3	Good		5.05
63	8.36	No	6.8	Excellent		5.05
163	7.62	No	7.5	Poor		5.04
459	7.92	No	7.2	OK		5.04
1615	7.90	No	7.2	Poor		5.03
1420	7.77	No	7.3	Poor		5.02
1693	6.77	No	8.3	Poor		5.02
1419	8.27	No	6.8	Bad		5.02
335	8.15	No	6.9	Excellent		5.02
770	8.14	No	6.9	Excellent		5.01

156	8.33	No	6.7	Excellent		5.01
136	8.33	No	6.7	Excellent		5.01
607	8.43	No	6.6	Excellent		5.01
966	7.61	No	7.4	Poor		5.00
1117	8.10	No	6.9	Excellent		5.00
932	7.29	No	7.7	Good		5.00
1661	8.09	No	6.9	OK		5.00
1480	8.17	No	6.8	Excellent		4.99
1634	7.46	No	7.5	Good		4.99
1162	8.05	No	6.9	Excellent		4.98
1681	3.18	No	7.2	Excellent	4.57	4.98
1268	8.25	No	6.7	Excellent		4.98
1310	8.24	No	6.7	Excellent		4.98
143	7.04	No	7.9	Bad		4.98
461	8.11	No	6.8	Excellent		4.97
672	8.30	No	6.6	Excellent		4.97
1320	7.70	No	7.2	Good		4.97
276	8.20	No	6.7	Good		4.97
116	8.87	No	6	Excellent		4.96
825	8.07	No	6.8	Excellent		4.96
748	7.16	No	7.7	Bad		4.95
836	7.84	No	7	OK		4.95
344	8.02	No	6.8	Excellent		4.94
1700	7.09	No	7.7	OK		4.93
148	8.48	No	6.3	Good		4.93
874	8.17	No	6.6	Excellent		4.92
661	8.56	No	6.2	OK		4.92
757	8.06	No	6.7	Excellent		4.92
668	7.45	No	7.3	Good		4.92
912	8.24	No	6.5	Excellent		4.91
1674	7.73	No	7	Poor		4.91
1537	7.62	No	7.1	OK		4.91
98	8.32	No	6.4	Excellent		4.91
779	7.81	No	6.9	Good		4.90
102	8.19	No	6.5	Good		4.90
5	8.19	No	6.5	Excellent		4.90
1243	8.27	No	6.4	Good		4.89
480	8.06	No	6.6	Poor		4.89
467	7.45	No	7.2	Good		4.88
259	7.74	No	6.9	Excellent		4.88
227	8.13	No	6.5	Excellent		4.88
1656	7.42	No	7.2	Good		4.87
126	8.01	No	6.6	Good		4.87

1294	7.71	No	6.9	Bad		4.87
821	3.11	No	8	Excellent	3.50	4.87
1354	7.19	No	7.4	Good		4.86
1564	7.78	No	6.8	Excellent		4.86
505	7.78	No	6.8	Poor		4.86
267	7.87	No	6.7	Excellent		4.86
937	7.97	No	6.6	Excellent		4.86
658	7.67	No	6.9	Excellent		4.86
674	8.05	No	6.5	Excellent		4.85
213	8.05	No	6.5	Excellent		4.85
1212	6.83	No	7.7	Poor		4.84
1040	8.73	No	5.8	Good		4.84
843	7.63	No	6.9	Poor		4.84
1222	7.41	No	7.1	Good		4.84
1423	7.70	No	6.8	Bad		4.83
436	8.38	No	6.1	Excellent		4.83
1358	7.58	No	6.9	Excellent		4.83
851	7.76	No	6.7	Good		4.82
873	8.25	No	6.2	Good		4.82
1498	4.55	No	7.1	Excellent	2.80	4.82
1069	6.74	No	7.7	Poor		4.81
1383	7.14	No	7.3	OK		4.81
1270	7.14	No	7.3	OK		4.81
795	7.74	No	6.7	Excellent		4.81
952	7.74	No	6.7	Excellent		4.81
1484	8.03	No	6.4	Good		4.81
915	7.83	No	6.6	OK		4.81
1013	3.86	No	7.1	Excellent	3.47	4.81
1427	7.72	No	6.7	Poor		4.81
168	7.60	No	6.8	Excellent		4.80
703	7.39	No	7	Good		4.80
924	7.99	No	6.4	Good		4.80
281	7.88	No	6.5	Good		4.79
1140	7.47	No	6.9	Excellent		4.79
343	7.57	No	6.8	Excellent		4.79
552	7.77	No	6.6	Excellent		4.79
909	6.76	YES	7.6	Good		4.79
1167	9.36	No	5	Poor		4.79
1075	7.75	No	6.6	Bad		4.78
574	9.75	No	4.6	Bad		4.78
852	7.25	No	7.1	Good		4.78
422	7.13	No	7.2	Good		4.78
664	7.12	No	7.2	OK		4.77

1110	7.41	No	6.9	Excellent	4.77
58	8.00	No	6.3	OK	4.77
1597	7.89	No	6.4	Excellent	4.76
26	7.19	No	7.1	Good	4.76
1560	7.49	No	6.8	Excellent	4.76
73	8.89	No	5.4	OK	4.76
373	6.89	No	7.4	Poor	4.76
324	7.68	No	6.6	Excellent	4.76
1298	7.58	No	6.7	Excellent	4.76
1504	7.46	No	6.8	Excellent	4.75
1552	5.95	No	8.3	Good	4.75
1628	5.75	No	8.5	Poor	4.75
56	7.75	No	6.5	Excellent	4.75
1381	7.45	No	6.8	Excellent	4.75
686	7.64	No	6.6	Poor	4.75
481	8.24	No	6	Excellent	4.75
1025	7.64	No	6.6	Excellent	4.75
772	7.64	No	6.6	Excellent	4.75
1213	7.64	No	6.6	Excellent	4.75
290	6.93	No	7.3	OK	4.74
462	7.62	No	6.6	Poor	4.74
786	7.52	No	6.7	Excellent	4.74
1067	7.71	No	6.5	OK	4.74
1704	7.71	No	6.5	OK	4.74
209	7.50	No	6.7	Excellent	4.73
489	8.50	No	5.7	OK	4.73
1430	7.40	No	6.8	Poor	4.73
604	7.89	No	6.3	Good	4.73
1567	7.59	No	6.6	Good	4.73
765	7.09	No	7.1	Good	4.73
101	7.38	No	6.8	Excellent	4.73
398	8.47	No	5.7	Poor	4.72
1107	7.45	No	6.7	Excellent	4.72
1456	5.54	No	8.6	Poor	4.71
1580	6.74	No	7.4	Good	4.71
1488	7.23	No	6.9	Excellent	4.71
1583	8.12	No	6	Good	4.71
1343	7.42	No	6.7	Excellent	4.71
142	7.42	No	6.7	Excellent	4.71
1602	5.61	No	8.5	Bad	4.70
1443	8.20	No	5.9	OK	4.70
1620	7.96	No	6.1	Excellent	4.69
254	7.36	No	6.7	Excellent	4.69

595	7.36	YES	6.7	Excellent		4.69
425	7.95	No	6.1	Excellent		4.68
1175	7.25	No	6.8	Good		4.68
321	6.84	No	7.2	OK		4.68
1539	9.44	No	4.6	Bad		4.68
103	7.04	No	7	Poor		4.68
782	7.54	No	6.5	Excellent		4.68
1308	7.14	No	6.9	Bad		4.68
9	7.34	No	6.7	Good		4.68
587	7.54	No	6.5	OK		4.68
1079	7.63	No	6.4	Excellent		4.68
1016	7.33	No	6.7	Excellent		4.68
377	6.52	No	7.5	Poor		4.67
1475	7.62	No	6.4	Good		4.67
1451	8.01	No	6	OK		4.67
1171	7.50	No	6.5	Excellent		4.67
87	7.50	No	6.5	Excellent		4.67
1523	4.48	YES	7.2	Excellent	2.30	4.66
1153	7.68	No	6.3	OK		4.66
364	7.08	No	6.9	Excellent		4.66
1555	6.27	No	7.7	Good		4.66
1664	6.86	No	7.1	Good		4.65
527	7.15	No	6.8	Excellent		4.65
1066	6.85	No	7.1	Good		4.65
233	3.58	No	7.3	Excellent	3.06	4.65
328	7.24	No	6.7	Excellent		4.65
15	6.33	No	7.6	Good		4.64
723	7.52	No	6.4	Excellent		4.64
1290	7.52	No	6.4	Excellent		4.64
1527	7.51	No	6.4	Excellent		4.64
713	5.51	No	8.4	Good		4.64
457	7.00	No	6.9	Good		4.63
1131	7.10	No	6.8	Excellent		4.63
1202	7.30	No	6.6	Excellent		4.63
1623	7.70	No	6.2	Excellent		4.63
736	6.99	No	6.9	Excellent		4.63
545	6.98	No	6.9	Good		4.63
1508	7.78	No	6.1	Good		4.63
588	7.07	No	6.8	Excellent		4.62
152	7.06	No	6.8	Excellent		4.62
1442	7.05	No	6.8	Excellent		4.62
289	7.64	No	6.2	Good		4.61
359	7.74	No	6.1	Excellent		4.61

1493	8.34	No	5.5	Excellent	4.61
336	7.54	No	6.3	Excellent	4.61
982	7.13	No	6.7	Good	4.61
512	6.72	No	7.1	Poor	4.61
1413	7.21	No	6.6	Good	4.60
1236	8.19	No	5.6	Good	4.60
868	6.79	YES	7	Good	4.60
1718	6.99	No	6.8	Excellent	4.60
347	7.49	No	6.3	OK	4.60
1004	6.69	No	7.1	Poor	4.60
418	7.48	No	6.3	Excellent	4.59
606	6.68	No	7.1	Good	4.59
1203	7.28	No	6.5	Good	4.59
329	7.48	No	6.3	Excellent	4.59
218	6.87	No	6.9	Excellent	4.59
129	7.46	No	6.3	Excellent	4.59
1283	7.35	No	6.4	Excellent	4.58
1407	6.84	No	6.9	Excellent	4.58
382	6.84	YES	6.9	Excellent	4.58
1614	6.34	No	7.4	Good	4.58
1666	7.33	No	6.4	Good	4.58
397	7.23	No	6.5	Excellent	4.58
476	7.93	No	5.8	Excellent	4.58
1261	7.02	No	6.7	OK	4.57
110	6.92	No	6.8	Excellent	4.57
439	6.52	YES	7.2	Good	4.57
741	6.30	No	7.4	Poor	4.57
251	6.80	No	6.9	Excellent	4.57
906	6.00	No	7.7	Poor	4.57
1540	7.60	No	6.1	Poor	4.57
154	6.99	No	6.7	Excellent	4.56
1149	7.09	No	6.6	Excellent	4.56
107	6.78	YES	6.9	Excellent	4.56
304	6.98	No	6.7	Good	4.56
1556	6.88	No	6.8	Excellent	4.56
1047	5.88	No	7.8	Poor	4.56
273	7.07	No	6.6	Excellent	4.56
677	6.97	No	6.7	Excellent	4.56
849	6.16	No	7.5	OK	4.55
1566	6.76	No	6.9	Excellent	4.55
493	7.46	No	6.2	Excellent	4.55
147	7.05	No	6.6	Excellent	4.55
942	6.75	No	6.9	Good	4.55

1617	7.25	No	6.4	Poor	4.55
1193	7.74	No	5.9	Excellent	4.55
275	7.03	No	6.6	Good	4.54
23	6.72	YES	6.9	Excellent	4.54
1641	6.72	No	6.9	Excellent	4.54
1313	7.31	No	6.3	Excellent	4.54
332	7.30	No	6.3	Good	4.53
667	7.50	No	6.1	Excellent	4.53
754	6.70	YES	6.9	Excellent	4.53
333	6.89	No	6.7	Poor	4.53
799	6.18	No	7.4	OK	4.53
92	6.87	No	6.7	Excellent	4.52
702	7.07	No	6.5	Excellent	4.52
1096	7.55	No	6	Excellent	4.52
46	6.65	YES	6.9	Excellent	4.52
1611	6.94	No	6.6	Good	4.51
973	6.63	No	6.9	Good	4.51
837	6.12	No	7.4	Good	4.51
262	6.72	No	6.8	Excellent	4.51
1226	7.02	No	6.5	Excellent	4.51
118	6.72	No	6.8	Excellent	4.51
665	8.01	No	5.5	OK	4.50
192	6.60	No	6.9	Good	4.50
711	6.79	YES	6.7	Poor	4.50
338	6.68	No	6.8	Excellent	4.49
737	6.58	No	6.9	Excellent	4.49
173	6.87	No	6.6	OK	4.49
789	7.26	No	6.2	Excellent	4.49
610	6.76	No	6.7	Excellent	4.49
1187	7.05	No	6.4	Excellent	4.48
247	6.65	No	6.8	Excellent	4.48
248	6.84	No	6.6	Bad	4.48
963	6.14	No	7.3	OK	4.48
563	6.54	No	6.9	Excellent	4.48
749	6.84	No	6.6	Good	4.48
877	7.44	No	6	Poor	4.48
232	6.03	No	7.4	Good	4.48
1388	6.73	No	6.7	Excellent	4.48
867	7.23	No	6.2	Excellent	4.48
1072	7.13	No	6.3	OK	4.48
416	6.62	No	6.8	Excellent	4.47
144	6.52	YES	6.9	Excellent	4.47
388	6.82	No	6.6	Excellent	4.47

1626	5.61	No	7.8	OK	4.47
880	6.81	No	6.6	Good	4.47
484	6.90	No	6.5	Poor	4.47
278	6.00	No	7.4	Good	4.47
1673	6.30	No	7.1	Poor	4.47
1184	6.80	No	6.6	Excellent	4.47
523	6.99	No	6.4	OK	4.46
807	6.59	No	6.8	Excellent	4.46
1588	6.79	No	6.6	Excellent	4.46
927	6.19	No	7.2	OK	4.46
993	6.48	No	6.9	Excellent	4.46
1538	6.38	No	7	Good	4.46
1015	5.58	No	7.8	Bad	4.46
1250	8.78	No	4.6	Poor	4.46
357	6.47	No	6.9	Excellent	4.46
1550	6.77	No	6.6	Excellent	4.46
204	6.56	No	6.8	Excellent	4.45
300	6.95	No	6.4	Excellent	4.45
1396	6.94	No	6.4	Excellent	4.45
1613	3.84	No	9.5	Poor	4.45
1447	7.23	No	6.1	Good	4.44
487	6.43	YES	6.9	Good	4.44
282	7.31	No	6	Good	4.44
77	7.91	No	5.4	Excellent	4.44
1150	6.50	No	6.8	Excellent	4.43
1530	6.49	No	6.8	Excellent	4.43
237	6.09	No	7.2	Good	4.43
230	6.69	YES	6.6	Excellent	4.43
1406	7.07	No	6.2	Excellent	4.42
1161	7.07	No	6.2	Poor	4.42
1027	6.57	YES	6.7	Excellent	4.42
96	6.37	No	6.9	Excellent	4.42
954	6.36	No	6.9	Excellent	4.42
623	6.46	No	6.8	Excellent	4.42
1595	5.66	No	7.6	Poor	4.42
833	6.66	No	6.6	Excellent	4.42
746	6.35	No	6.9	Excellent	4.42
1192	5.85	No	7.4	Good	4.42
1441	6.44	No	6.8	Excellent	4.41
1315	6.44	No	6.8	Excellent	4.41
1701	6.74	No	6.5	Excellent	4.41
185	6.33	YES	6.9	Good	4.41
1500	6.73	No	6.5	Excellent	4.41

900	6.43	No	6.8	Excellent	4.41
1341	6.03	No	7.2	Good	4.41
49	7.03	No	6.2	Excellent	4.41
288	6.82	YES	6.4	Good	4.41
704	6.32	No	6.9	Excellent	4.41
641	5.72	No	7.5	Poor	4.41
1497	6.81	No	6.4	Excellent	4.40
430	6.71	No	6.5	Excellent	4.40
1481	6.11	No	7.1	Good	4.40
1412	6.50	YES	6.7	Excellent	4.40
953	6.40	No	6.8	Excellent	4.40
1519	6.30	No	6.9	Excellent	4.40
177	6.90	No	6.3	Excellent	4.40
222	5.39	No	7.8	OK	4.40
7	6.29	No	6.9	Excellent	4.40
959	6.68	No	6.5	Excellent	4.39
1010	5.68	No	7.5	OK	4.39
994	7.08	No	6.1	Excellent	4.39
1616	6.27	No	6.9	Excellent	4.39
1505	6.77	YES	6.4	Excellent	4.39
1650	6.57	No	6.6	Good	4.39
236	6.57	YES	6.6	Excellent	4.39
1008	6.97	No	6.2	Excellent	4.39
1659	5.77	No	7.4	Poor	4.39
365	6.36	No	6.8	Excellent	4.39
1651	6.95	No	6.2	Bad	4.38
221	6.25	No	6.9	Excellent	4.38
1323	5.95	No	7.2	OK	4.38
48	8.54	No	4.6	Poor	4.38
551	6.84	YES	6.3	Excellent	4.38
1328	5.84	YES	7.3	OK	4.38
586	6.33	No	6.8	Excellent	4.38
305	6.23	No	6.9	Good	4.38
1125	6.63	No	6.5	Excellent	4.38
385	5.32	No	7.8	Good	4.37
740	6.72	No	6.4	Excellent	4.37
830	6.22	YES	6.9	Excellent	4.37
1211	6.32	No	6.8	Excellent	4.37
864	6.52	YES	6.6	Good	4.37
1073	7.10	No	6	Excellent	4.37
882	6.40	No	6.7	Excellent	4.37
308	6.59	No	6.5	Excellent	4.36
39	6.39	YES	6.7	Excellent	4.36

72	6.19	No	6.9	OK	4.36
198	6.69	No	6.4	Excellent	4.36
785	5.98	No	7.1	Good	4.36
1053	6.18	No	6.9	Good	4.36
1198	6.78	No	6.3	Excellent	4.36
659	6.98	No	6.1	Excellent	4.36
1522	6.28	No	6.8	Excellent	4.36
776	6.17	No	6.9	Excellent	4.36
24	6.16	No	6.9	Excellent	4.35
1061	6.46	No	6.6	Excellent	4.35
309	5.46	No	7.6	Good	4.35
560	6.15	No	6.9	Excellent	4.35
1677	5.05	No	8	Bad	4.35
370	6.35	No	6.7	Excellent	4.35
1433	6.34	No	6.7	Excellent	4.35
1525	6.32	YES	6.7	Excellent	4.34
1159	6.42	No	6.6	OK	4.34
458	5.62	No	7.4	Poor	4.34
1680	6.52	No	6.5	Poor	4.34
1512	6.92	No	6.1	Excellent	4.34
1116	6.61	No	6.4	Excellent	4.34
619	6.60	YES	6.4	Excellent	4.33
1028	6.80	No	6.2	Excellent	4.33
13	6.59	No	6.4	Excellent	4.33
714	5.89	No	7.1	Good	4.33
1691	7.09	No	5.9	Good	4.33
171	6.08	No	6.9	Excellent	4.33
1060	6.18	YES	6.8	Poor	4.33
89	6.27	No	6.7	Excellent	4.32
1330	6.07	No	6.9	Excellent	4.32
188	6.37	No	6.6	Excellent	4.32
1594	6.97	No	6	Poor	4.32
1440	6.27	YES	6.7	Excellent	4.32
255	6.37	No	6.6	Excellent	4.32
1108	6.66	YES	6.3	Excellent	4.32
263	6.56	No	6.4	OK	4.32
1379	6.26	No	6.7	Excellent	4.32
231	5.56	No	7.4	Bad	4.32
81	6.95	No	6	Excellent	4.32
86	6.05	No	6.9	Excellent	4.32
733	6.54	No	6.4	Good	4.31
132	6.12	No	6.8	Excellent	4.31
401	6.12	No	6.8	Excellent	4.31

844	6.21	YES	6.7	Excellent	4.30
533	6.01	No	6.9	Good	4.30
206	6.81	YES	6.1	Excellent	4.30
902	6.81	YES	6.1	Excellent	4.30
611	6.51	No	6.4	Excellent	4.30
408	6.91	YES	6	OK	4.30
1237	6.31	No	6.6	OK	4.30
228	6.10	No	6.8	Excellent	4.30
822	6.00	No	6.9	Excellent	4.30
633	6.10	YES	6.8	Excellent	4.30
1104	7.00	No	5.9	Excellent	4.30
193	6.40	No	6.5	Poor	4.30
726	5.79	No	7.1	Poor	4.30
999	6.09	No	6.8	Excellent	4.30
349	6.78	No	6.1	Excellent	4.29
363	5.18	No	7.7	Poor	4.29
1431	6.28	YES	6.6	OK	4.29
239	6.28	No	6.6	Excellent	4.29
698	6.27	YES	6.6	Excellent	4.29
1326	6.27	No	6.6	Excellent	4.29
1655	5.86	No	7	Bad	4.29
1126	6.25	YES	6.6	Excellent	4.28
226	5.95	No	6.9	Excellent	4.28
249	6.55	No	6.3	Excellent	4.28
913	6.14	YES	6.7	Excellent	4.28
1122	6.14	No	6.7	Excellent	4.28
1253	6.64	No	6.2	Excellent	4.28
1573	6.43	No	6.4	Bad	4.28
1196	5.93	YES	6.9	Excellent	4.28
1660	6.03	No	6.8	Excellent	4.28
150	6.43	No	6.4	Excellent	4.28
1337	5.92	No	6.9	Excellent	4.27
1106	6.02	No	6.8	Excellent	4.27
870	6.01	No	6.8	Excellent	4.27
572	6.01	No	6.8	Excellent	4.27
654	6.71	YES	6.1	Excellent	4.27
509	6.21	No	6.6	Excellent	4.27
908	6.01	No	6.8	Excellent	4.27
478	5.90	No	6.9	Excellent	4.27
565	6.00	YES	6.8	Excellent	4.27
1511	5.10	No	7.7	Good	4.27
1705	6.00	No	6.8	Bad	4.27
1023	6.39	YES	6.4	OK	4.26

25	6.39	No	6.4	Poor	4.26
315	6.19	No	6.6	Excellent	4.26
322	6.28	No	6.5	Excellent	4.26
485	6.48	YES	6.3	Good	4.26
97	5.88	No	6.9	Excellent	4.26
1141	6.37	No	6.4	Excellent	4.26
44	6.07	YES	6.7	Excellent	4.26
1528	6.67	No	6.1	Good	4.26
392	6.27	No	6.5	Excellent	4.26
1095	6.37	YES	6.4	Excellent	4.26
486	6.85	No	5.9	Excellent	4.25
562	6.35	No	6.4	Excellent	4.25
1418	5.95	No	6.8	Good	4.25
1146	5.85	No	6.9	Excellent	4.25
1541	5.74	No	7	Good	4.25
584	6.03	No	6.7	Excellent	4.24
11	6.23	No	6.5	Excellent	4.24
1639	6.22	No	6.5	Bad	4.24
1062	6.12	No	6.6	Excellent	4.24
1128	6.72	No	6	Excellent	4.24
1026	5.92	No	6.8	Poor	4.24
119	6.12	No	6.6	Excellent	4.24
372	6.22	No	6.5	Excellent	4.24
1392	5.82	YES	6.9	Excellent	4.24
88	6.21	No	6.5	Excellent	4.24
402	6.41	No	6.3	Excellent	4.24
701	5.41	No	7.3	Good	4.24
752	6.61	No	6.1	Excellent	4.24
806	5.81	No	6.9	Excellent	4.24
501	6.60	No	6.1	Bad	4.23
291	5.79	YES	6.9	Excellent	4.23
989	6.59	No	6.1	Excellent	4.23
803	6.18	No	6.5	Excellent	4.23
75	6.07	No	6.6	Excellent	4.22
19	5.97	No	6.7	OK	4.22
396	4.87	No	7.8	Good	4.22
82	6.06	No	6.6	OK	4.22
1083	5.86	No	6.8	Good	4.22
1558	5.75	No	6.9	Excellent	4.22
196	5.85	No	6.8	Excellent	4.22
1356	6.15	No	6.5	Excellent	4.22
622	6.34	No	6.3	Excellent	4.21
1434	5.83	No	6.8	Excellent	4.21

1297	5.53	No	7.1	Good	4.21
1183	6.43	No	6.2	Excellent	4.21
187	6.62	No	6	Excellent	4.21
320	6.11	No	6.5	Excellent	4.20
634	5.81	No	6.8	Excellent	4.20
1445	5.41	No	7.2	Bad	4.20
1093	4.40	No	8.2	OK	4.20
826	5.80	YES	6.8	Excellent	4.20
1078	6.29	No	6.3	Poor	4.20
879	6.69	No	5.9	Excellent	4.20
1699	5.69	No	6.9	OK	4.20
106	5.88	No	6.7	Excellent	4.19
1587	6.18	No	6.4	Excellent	4.19
1630	5.78	No	6.8	Poor	4.19
519	6.27	No	6.3	Excellent	4.19
447	6.17	No	6.4	Excellent	4.19
215	5.77	No	6.8	Excellent	4.19
721	5.87	No	6.7	Excellent	4.19
1005	6.16	No	6.4	Excellent	4.19
1536	5.86	YES	6.7	Excellent	4.19
453	6.26	No	6.3	Excellent	4.19
681	5.95	No	6.6	Good	4.18
241	5.64	No	6.9	Good	4.18
1055	6.13	No	6.4	Good	4.18
1142	5.73	No	6.8	Excellent	4.18
647	5.43	No	7.1	Good	4.18
958	5.93	No	6.6	Excellent	4.18
1637	6.43	No	6.1	Bad	4.18
887	6.33	No	6.2	Good	4.18
1307	5.13	No	7.4	Good	4.18
115	5.93	No	6.6	Good	4.18
407	6.52	No	6	Excellent	4.17
729	5.82	No	6.7	Excellent	4.17
1502	6.22	No	6.3	Excellent	4.17
1355	5.61	No	6.9	Good	4.17
1432	6.11	No	6.4	Excellent	4.17
628	5.69	No	6.8	Excellent	4.16
649	5.39	No	7.1	Good	4.16
650	5.79	No	6.7	Excellent	4.16
894	5.59	YES	6.9	Excellent	4.16
1160	5.79	No	6.7	Excellent	4.16
123	5.58	No	6.9	Excellent	4.16
905	5.98	No	6.5	Excellent	4.16

1057	5.67	No	6.8	Excellent	4.16
1446	6.06	YES	6.4	Excellent	4.15
435	5.76	No	6.7	Excellent	4.15
217	5.66	No	6.8	Excellent	4.15
268	6.05	No	6.4	OK	4.15
67	5.95	No	6.5	Excellent	4.15
1147	5.75	No	6.7	Excellent	4.15
1590	6.05	No	6.4	Excellent	4.15
1046	5.74	No	6.7	Good	4.15
1115	6.34	No	6.1	Excellent	4.15
856	7.44	No	5	OK	4.15
890	5.24	No	7.2	Poor	4.15
1182	5.84	No	6.6	Excellent	4.15
1321	5.53	No	6.9	Excellent	4.14
627	6.31	No	6.1	Excellent	4.14
517	5.11	No	7.3	Poor	4.14
1377	6.11	No	6.3	Poor	4.14
415	5.60	No	6.8	Excellent	4.13
1151	5.90	No	6.5	Excellent	4.13
1199	4.60	No	7.8	Poor	4.13
1465	5.79	No	6.6	Good	4.13
573	5.89	No	6.5	Excellent	4.13
600	6.38	YES	6	Excellent	4.13
1042	6.18	No	6.2	OK	4.13
778	5.78	No	6.6	Excellent	4.13
724	5.57	No	6.8	Excellent	4.12
1022	5.47	No	6.9	Excellent	4.12
54	5.97	No	6.4	Excellent	4.12
760	5.97	YES	6.4	Excellent	4.12
301	6.56	No	5.8	Good	4.12
679	6.05	No	6.3	Excellent	4.12
725	5.15	No	7.2	Good	4.12
286	6.55	No	5.8	Excellent	4.12
427	5.05	No	7.3	OK	4.12
504	6.05	No	6.3	Excellent	4.12
264	4.45	No	7.9	Bad	4.12
1157	5.74	No	6.6	OK	4.11
1706	4.14	No	8.2	OK	4.11
386	6.33	No	6	Excellent	4.11
817	5.03	No	7.3	Good	4.11
662	5.63	No	6.7	Excellent	4.11
351	6.32	No	6	Excellent	4.11
1105	6.02	No	6.3	Excellent	4.11

62	5.02	No	7.3	Good	4.11
1405	5.62	No	6.7	Excellent	4.11
987	5.51	No	6.8	Excellent	4.10
862	5.51	No	6.8	Excellent	4.10
100	5.51	No	6.8	Excellent	4.10
29	5.31	No	7	Poor	4.10
41	5.50	No	6.8	Excellent	4.10
1568	5.29	No	7	Bad	4.10
1424	5.39	No	6.9	Good	4.10
1293	5.69	No	6.6	Excellent	4.10
1524	5.58	No	6.7	Excellent	4.09
1164	5.48	No	6.8	Good	4.09
1346	6.07	No	6.2	Excellent	4.09
1516	5.37	No	6.9	OK	4.09
307	5.77	No	6.5	Excellent	4.09
743	5.56	No	6.7	Excellent	4.09
1100	3.96	No	8.3	Good	4.09
1393	5.56	No	6.7	Good	4.09
1282	5.45	No	6.8	Good	4.08
38	6.05	No	6.2	Excellent	4.08
1694	5.45	No	6.8	Good	4.08
1632	7.64	No	4.6	OK	4.08
64	6.14	No	6.1	Excellent	4.08
303	5.54	No	6.7	Excellent	4.08
256	5.34	No	6.9	Poor	4.08
1136	5.53	No	6.7	Excellent	4.08
808	5.72	No	6.5	Excellent	4.07
832	6.02	No	6.2	OK	4.07
442	5.42	YES	6.8	Excellent	4.07
1217	5.91	No	6.3	Excellent	4.07
700	5.31	YES	6.9	Excellent	4.07
52	5.30	No	6.9	Excellent	4.07
1385	4.70	No	7.5	Poor	4.07
510	5.79	No	6.4	Excellent	4.06
615	5.49	No	6.7	Excellent	4.06
771	5.39	No	6.8	Excellent	4.06
1246	5.88	No	6.3	Excellent	4.06
814	7.58	No	4.6	Poor	4.06
1336	5.98	No	6.2	OK	4.06
809	5.37	No	6.8	Excellent	4.06
1489	5.57	No	6.6	Excellent	4.06
1148	5.87	No	6.3	Excellent	4.06
1627	5.06	No	7.1	OK	4.05

105	5.96	No	6.2	Excellent	4.05
1043	5.06	No	7.1	Poor	4.05
521	5.96	No	6.2	Excellent	4.05
1276	6.24	No	5.9	Excellent	4.05
1592	3.94	No	8.2	Poor	4.05
285	5.53	No	6.6	Excellent	4.04
1244	5.63	No	6.5	Excellent	4.04
536	5.72	No	6.4	Excellent	4.04
1255	5.02	No	7.1	Bad	4.04
961	5.62	YES	6.5	Excellent	4.04
465	5.32	No	6.8	Good	4.04
1185	5.61	No	6.5	Excellent	4.04
960	5.19	No	6.9	Excellent	4.03
1264	5.39	No	6.7	Excellent	4.03
801	3.99	No	8.1	OK	4.03
599	6.06	No	6	Excellent	4.02
1529	5.76	No	6.3	Excellent	4.02
1252	5.36	YES	6.7	Excellent	4.02
804	6.66	YES	5.4	Excellent	4.02
437	5.86	No	6.2	Excellent	4.02
591	6.15	No	5.9	Excellent	4.02
266	5.65	No	6.4	Excellent	4.02
452	5.25	No	6.8	Excellent	4.02
1340	5.24	No	6.8	Excellent	4.01
694	5.14	No	6.9	Excellent	4.01
169	5.53	YES	6.5	Excellent	4.01
985	5.62	No	6.4	Excellent	4.01
280	5.52	No	6.5	Excellent	4.01
184	5.31	No	6.7	Good	4.00
1577	5.61	No	6.4	Excellent	4.00
30	5.51	No	6.5	OK	4.00
1314	5.51	No	6.5	Excellent	4.00
1000	5.70	YES	6.3	Excellent	4.00
350	5.80	No	6.2	Excellent	4.00
602	5.20	No	6.8	Poor	4.00
475	5.39	No	6.6	Excellent	4.00
1239	5.09	YES	6.9	Excellent	4.00
1671	5.89	No	6.1	Poor	4.00
1234	5.48	No	6.5	Excellent	3.99
125	6.18	No	5.8	Excellent	3.99
498	4.58	No	7.4	Poor	3.99
764	5.57	YES	6.4	Excellent	3.99
878	5.35	No	6.6	Excellent	3.98

1548	5.15	No	6.8	OK	3.98
1305	5.25	No	6.7	Excellent	3.98
780	5.35	No	6.6	Good	3.98
949	5.24	No	6.7	Good	3.98
663	5.32	No	6.6	Good	3.97
1494	5.12	No	6.8	Excellent	3.97
139	4.91	No	7	Good	3.97
31	5.61	YES	6.3	Excellent	3.97
1589	6.00	No	5.9	Poor	3.97
518	5.70	No	6.2	Excellent	3.97
492	5.79	No	6.1	OK	3.96
376	5.99	No	5.9	Excellent	3.96
160	5.18	No	6.7	OK	3.96
1260	6.28	YES	5.6	Excellent	3.96
371	5.66	No	6.2	Excellent	3.95
762	5.36	No	6.5	OK	3.95
128	5.46	No	6.4	OK	3.95
1258	5.76	No	6.1	Excellent	3.95
750	4.96	No	6.9	Good	3.95
1417	5.05	No	6.8	Excellent	3.95
499	5.54	No	6.3	OK	3.95
43	5.04	No	6.8	Excellent	3.95
575	5.04	No	6.8	Poor	3.95
1210	4.84	No	7	OK	3.95
429	5.42	No	6.4	Excellent	3.94
391	4.92	No	6.9	Bad	3.94
592	5.71	No	6.1	Excellent	3.94
1248	5.10	No	6.7	Bad	3.93
731	5.19	No	6.6	Poor	3.93
775	6.29	No	5.5	Excellent	3.93
707	4.29	No	7.5	OK	3.93
1044	5.18	No	6.6	Good	3.93
59	5.27	No	6.5	Excellent	3.92
784	5.76	No	6	Good	3.92
483	5.06	No	6.7	Excellent	3.92
117	4.56	No	7.2	OK	3.92
507	5.15	No	6.6	Excellent	3.92
76	5.04	No	6.7	Excellent	3.91
1279	5.14	No	6.6	Excellent	3.91
898	5.03	No	6.7	Excellent	3.91
946	4.81	No	6.9	Excellent	3.90
831	4.71	No	7	Good	3.90
1472	4.80	No	6.9	Excellent	3.90

471	5.09	No	6.6	Bad	3.90
1241	4.58	No	7.1	Poor	3.89
969	4.98	No	6.7	Excellent	3.89
948	4.48	No	7.2	Good	3.89
990	7.07	No	4.6	Bad	3.89
260	5.07	YES	6.6	Poor	3.89
1348	4.76	No	6.9	Good	3.89
936	5.05	YES	6.6	Poor	3.88
1401	5.64	No	6	Excellent	3.88
497	5.14	No	6.5	Excellent	3.88
108	4.94	No	6.7	Good	3.88
608	5.24	No	6.4	Excellent	3.88
141	6.03	No	5.6	Excellent	3.88
692	5.32	YES	6.3	Excellent	3.87
470	4.42	No	7.2	Poor	3.87
676	5.40	No	6.2	Bad	3.87
1200	4.70	YES	6.9	Good	3.87
428	5.10	YES	6.5	Excellent	3.87
1712	4.99	No	6.6	Good	3.86
549	5.38	No	6.2	Good	3.86
1685	5.88	No	5.7	Good	3.86
1711	3.78	No	7.8	Poor	3.86
112	4.06	YES	7.5	Bad	3.85
361	4.76	YES	6.8	Excellent	3.85
1084	4.76	No	6.8	Bad	3.85
1295	4.75	No	6.8	Excellent	3.85
413	7.04	No	4.5	Bad	3.85
1086	5.13	No	6.4	Excellent	3.84
1482	4.82	YES	6.7	Excellent	3.84
1668	5.20	No	6.3	Excellent	3.83
1216	5.20	No	6.3	Excellent	3.83
1257	5.59	No	5.9	Excellent	3.83
997	4.68	YES	6.8	Excellent	3.83
709	5.48	No	6	Excellent	3.83
941	4.77	No	6.7	Excellent	3.82
1619	4.47	No	7	Bad	3.82
1622	4.47	No	7	OK	3.82
1082	5.45	YES	6	Excellent	3.82
1531	5.43	YES	6	Excellent	3.81
513	5.03	YES	6.4	Good	3.81
34	5.52	No	5.9	Good	3.81
675	5.01	No	6.4	Excellent	3.80
1585	6.90	No	4.5	Excellent	3.80

626	5.19	No	6.2	Bad	3.80
1575	4.19	No	7.2	OK	3.80
1332	4.29	No	7.1	Bad	3.80
161	5.38	No	6	Excellent	3.79
992	4.17	No	7.2	OK	3.79
1391	4.27	No	7.1	Poor	3.79
970	4.67	No	6.7	Excellent	3.79
366	4.66	No	6.7	Good	3.79
1173	4.85	No	6.5	Excellent	3.78
781	5.15	No	6.2	Excellent	3.78
384	6.84	No	4.5	OK	3.78
1138	4.74	YES	6.6	Excellent	3.78
695	4.44	No	6.9	OK	3.78
146	6.84	No	4.5	OK	3.78
596	3.32	No	8	Good	3.77
460	4.82	No	6.5	Good	3.77
1460	5.01	YES	6.3	Excellent	3.77
1695	4.50	No	6.8	Excellent	3.77
240	5.40	YES	5.9	OK	3.77
153	4.89	No	6.4	Excellent	3.76
451	4.48	No	6.8	Good	3.76
1312	4.58	No	6.7	Excellent	3.76
1134	4.56	YES	6.7	Excellent	3.75
758	5.06	No	6.2	Good	3.75
792	4.75	No	6.5	Excellent	3.75
202	4.65	No	6.6	OK	3.75
1543	4.75	No	6.5	Excellent	3.75
1603	2.65	No	8.6	OK	3.75
1272	5.14	No	6.1	OK	3.75
207	4.62	No	6.6	Excellent	3.74
375	4.30	YES	6.9	Poor	3.73
1586	5.89	No	5.3	Poor	3.73
1130	5.09	YES	6.1	Good	3.73
1535	5.08	No	6.1	Excellent	3.73
323	4.98	No	6.2	Excellent	3.73
569	4.17	No	7	Poor	3.72
1503	4.16	No	7	OK	3.72
978	4.06	No	7.1	Bad	3.72
1269	5.06	No	6.1	Bad	3.72
1103	5.05	No	6.1	Excellent	3.72
1367	4.44	No	6.7	Excellent	3.71
636	4.64	No	6.5	Excellent	3.71
179	5.14	YES	6	Excellent	3.71

131	5.34	No	5.8	Excellent	3.71
718	6.81	No	4.3	OK	3.70
716	4.31	YES	6.8	Excellent	3.70
699	4.41	No	6.7	Good	3.70
1278	4.20	No	6.9	Poor	3.70
589	4.50	No	6.6	OK	3.70
299	4.70	No	6.4	Excellent	3.70
1459	4.59	No	6.5	Poor	3.70
124	3.59	No	7.5	Poor	3.70
876	4.99	YES	6.1	Excellent	3.70
1670	3.97	No	7.1	Bad	3.69
811	5.05	No	6	Excellent	3.68
473	4.24	No	6.8	Excellent	3.68
735	4.83	YES	6.2	Excellent	3.68
172	4.40	No	6.6	Excellent	3.67
773	4.80	No	6.2	Excellent	3.67
400	4.87	YES	6.1	Excellent	3.66
531	4.06	No	6.9	Excellent	3.65
624	4.65	No	6.3	Excellent	3.65
768	6.64	No	4.3	Excellent	3.65
334	4.33	No	6.6	Excellent	3.64
189	4.73	No	6.2	Excellent	3.64
1438	4.43	No	6.5	Excellent	3.64
1389	4.82	No	6.1	Excellent	3.64
583	4.01	No	6.9	Bad	3.64
61	4.99	No	5.9	Good	3.63
1163	4.58	No	6.3	Excellent	3.63
632	4.27	No	6.6	Excellent	3.62
717	4.26	No	6.6	Bad	3.62
424	4.45	No	6.4	Excellent	3.62
417	4.65	YES	6.2	Good	3.62
1119	5.32	No	5.5	Excellent	3.61
886	4.59	YES	6.2	OK	3.60
534	4.08	No	6.7	Excellent	3.59
1689	4.18	No	6.6	OK	3.59
345	4.85	No	5.9	Excellent	3.58
1374	4.45	No	6.3	Excellent	3.58
1624	5.23	No	5.5	Excellent	3.58
313	3.20	No	7.5	Bad	3.57
1132	3.57	No	7.1	Good	3.56
543	6.45	No	4.2	Excellent	3.55
135	4.24	No	6.4	Good	3.55
113	4.24	No	6.4	OK	3.55

1398	3.71	No	6.9	Excellent	3.54
1644	6.40	No	4.2	Bad	3.53
911	5.18	YES	5.4	Excellent	3.53
585	6.37	No	4.2	Poor	3.52
183	3.74	No	6.8	Excellent	3.51
33	5.03	YES	5.5	Excellent	3.51
965	4.12	No	6.4	Excellent	3.51
691	4.02	YES	6.5	Good	3.51
1458	3.91	No	6.6	Excellent	3.50
1470	4.06	No	6.4	Bad	3.49
1296	4.31	YES	6.1	Excellent	3.47
919	4.31	No	6.1	Excellent	3.47
1054	3.98	YES	6.4	Excellent	3.46
431	3.95	No	6.4	Excellent	3.45
1719	6.10	No	4.2	OK	3.43
450	4.47	No	5.8	Poor	3.42
1654	3.93	No	6.3	Bad	3.41
1292	3.53	No	6.7	Excellent	3.41
1721	5.99	No	4.2	Excellent	3.40
979	5.99	No	4.2	Poor	3.40
612	3.86	No	6.3	Excellent	3.39
1335	2.44	No	7.7	Good	3.38
1717	3.42	No	6.7	Excellent	3.37
1316	4.11	YES	6	Excellent	3.37
1576	3.90	No	6.2	Bad	3.37
550	2.26	No	7.8	Good	3.35
883	3.76	No	6.3	Bad	3.35
219	3.61	No	6.4	Excellent	3.34
1347	3.10	No	6.9	Excellent	3.33
1286	3.28	No	6.7	Good	3.33
35	4.06	No	5.9	Excellent	3.32
316	3.96	No	6	Excellent	3.32
1640	2.91	No	7	OK	3.30
238	5.77	No	4.1	Excellent	3.29
930	3.22	No	6.6	Excellent	3.27
158	3.36	No	6.4	Excellent	3.25
1702	2.97	No	6.7	OK	3.22
404	4.25	No	5.4	Excellent	3.22
186	5.52	No	4.1	OK	3.21
1676	2.17	No	7.4	Bad	3.19
590	3.57	No	6	Excellent	3.19
1662	5.46	No	4.1	Excellent	3.19
766	3.24	No	6.3	Good	3.18

155	3.63	No	5.9	Excellent	3.18
1563	2.12	No	7.4	OK	3.17
1636	3.40	No	6.1	Poor	3.17
819	3.57	No	5.9	Good	3.16
644	3.24	No	6.2	OK	3.15
1687	2.88	No	6.4	Poor	3.09
1633	2.67	No	6.6	Good	3.09
1710	2.76	No	6.5	Poor	3.09
1720	5.11	No	4.1	OK	3.07
1220	2.08	No	7.1	OK	3.06
951	2.19	No	6.9	Excellent	3.03
1600	2.87	No	6.2	Bad	3.02
330	3.89	No	5.1	Excellent	3.00
1707	2.50	No	6.4	Poor	2.97
660	2.79	No	6.1	Excellent	2.96
1631	2.25	No	6.6	Poor	2.95
838	4.79	No	4	Excellent	2.93
1618	4.76	No	4	Poor	2.92
1554	4.74	No	4	OK	2.91
1593	4.69	No	4	Excellent	2.90
1658	3.31	No	5.2	Poor	2.84
1518	2.40	No	6.1	Poor	2.83
1357	1.77	No	6.7	Good	2.82
68	1.23	No	7.1	Bad	2.78
559	2.70	YES	5.6	Excellent	2.77
1599	4.17	No	3.9	Poor	2.69
1007	3.66	No	3.7	Poor	2.45
1610	2.66	No	4.6	Excellent	2.42
1690	-1.20	No	8.4	Bad	2.40
1669	-0.77	No	7.8	Bad	2.34
1621	-0.64	No	7.6	Bad	2.32
1709	-0.49	No	7	Bad	2.17
1608	-0.01	No	6.5	Bad	2.16
1665	-0.23	No	6.4	Bad	2.06
1647	-1.38	No	7.5	Bad	2.04
1649	-0.73	No	6.4	Bad	1.89
1686	-1.39	No	6.9	Bad	1.84
1708	-0.40	No	5.5	Poor	1.70
191	-0.60	No	4.9	Bad	1.43
1688	-1.68	No	5.6	Bad	1.31

Table S3. Experimental and calculated K_i by docking.

S2RSLDB ID	Exp. K_i (nM)	Calcd. K_i (nM)
2	1.50	0.68
6	3.00	3.84
7	11.60	0.79
13	5.50	4.64
19	4.37	1.30
20	9.60	3.07
23	0.88	1.15
28	14.60	4.51
29	5.00	10.08
32	4.24	1.05
35	10.00	2.22
36	10.20	2.91
46	26.80	5.17
51	12.90	5.52
52	9.24	2.17
54	12.20	5.97
56	12.60	3.96
58	5.34	0.56
61	15.00	10.75
62	8.68	5.14
65	94.00	5.78
68	8.00	1.07
69	32.00	12.61
79	4.84	1.23
82	20.10	2.84
86	2.40	4.35
92	43.00	1.38
94	20.00	9.62
96	3.56	10.70
103	4.90	1.55
104	1.67	13.37
106	8.68	10.08
107	5.74	2.13
112	29.60	0.82
113	11.00	4.37
121	6.30	7.54
124	20.00	21.71
130	16.00	3.18
134	12.30	0.65
136	26.80	19.81
139	11.00	3.18
144	11.00	1.18
150	33.00	1.52
154	39.00	24.54
155	5.90	2.90
158	12.00	8.93
161	9.90	11.06
162	50.90	5.91
165	13.00	5.28
167	6.72	5.49
171	20.00	11.12
172	2.90	2.05
173	8.90	7.69
175	5.90	5.55
177	46.60	7.77
179	25.20	7.54
182	25.20	8.07
183	75.00	1.92
184	8.00	37.68
185	2.57	12.94
186	52.00	2.01
189	11.50	32.03
190	67.50	21.67
191	7.65	15.89
192	12.60	10.43
193	27.50	4.08
198	4.40	7.30
199	5.45	1.45
204	5.92	13.55
208	10.50	15.15
210	50.00	5.32
211	30.60	12.24
212	85.90	36.31
219	32.00	2.50
223	18.80	23.96
224	62.00	36.33
225	183.90	36.15
226	2.15	4.70
227	8.94	27.42
228	7.71	17.33
230	26.80	2.36
239	94.00	8.45
241	6.00	3.15
248	19.00	4.82
259	27.00	2.02
266	21.20	1.84
273	3.24	14.20
274	12.70	7.40
275	22.90	15.99
278	100.00	10.30
280	40.70	1.81
282	3.78	23.95
283	25.60	2.52
287	12.60	54.50
289	1.96	11.47

295	55.00	39.33	481	2.44	14.59
296	27.30	5.34	483	2.66	7.44
297	44.00	9.75	485	10.10	26.41
299	150.00	2.02	490	28.30	5.10
306	734.01	35.31	493	4.02	13.37
308	7.04	8.35	494	3.05	9.55
310	29.10	7.03	495	48.13	14.67
311	2.79	5.98	496	23.20	29.69
312	4.95	22.42	498	16.00	14.11
316	10.00	7.07	499	3.73	5.65
319	39.40	4.32	501	5.89	55.35
321	0.77	52.63	505	41.10	71.39
329	3.10	1.14	506	4.47	40.95
331	9.30	2.74	508	26.10	22.95
334	14.90	12.39	509	1.90	11.14
341	21.00	7.08	513	90.60	17.06
345	105.00	25.42	518	11.44	69.15
348	76.00	18.40	520	8.40	54.55
354	2.46	18.92	521	113.00	23.21
356	30.00	3.42	522	11.28	9.74
357	1.49	42.64	526	26.40	55.16
367	0.57	4.64	529	3.06	14.48
372	21.26	37.65	530	42.30	44.64
373	19.00	10.97	533	29.29	6.19
377	6.34	34.85	535	34.50	11.19
381	10.41	45.47	536	161.00	6.53
382	6.89	45.16	540	14.90	8.30
387	25.00	63.46	547	12.40	7.18
389	35.20	19.24	548	8.80	8.38
398	18.80	4.02	551	309.00	33.75
399	4.72	26.07	552	9.72	62.71
403	30.10	5.37	554	45.90	37.26
415	1.64	17.90	555	59.30	11.83
416	4.85	14.70	559	16.43	22.23
423	8.49	32.98	562	32.70	51.26
427	3.15	3.68	565	21.20	17.63
428	9.00	4.38	568	10.90	28.12
430	8.52	14.44	570	117.00	95.00
441	10.10	12.53	571	14.60	116.35
443	46.90	58.19	575	15.00	40.97
454	2.22	6.62	576	21.90	97.61
457	6.30	12.07	578	9.90	89.90
458	2.12	20.06	579	207.00	169.20
459	4.90	40.45	583	14.00	139.81
462	2.31	4.90	586	29.60	77.76
464	15.36	15.96	587	13.80	206.46
469	2.90	27.66	588	57.00	51.96
472	16.50	10.49	594	26.10	76.99
478	12.10	11.80	600	56.60	207.87

603	18.80	57.21	628	17.78	218.83
611	28.10	140.61	648	34.00	336.55
616	69.80	150.87	650	23.00	877.30
626	97.72	316.24			

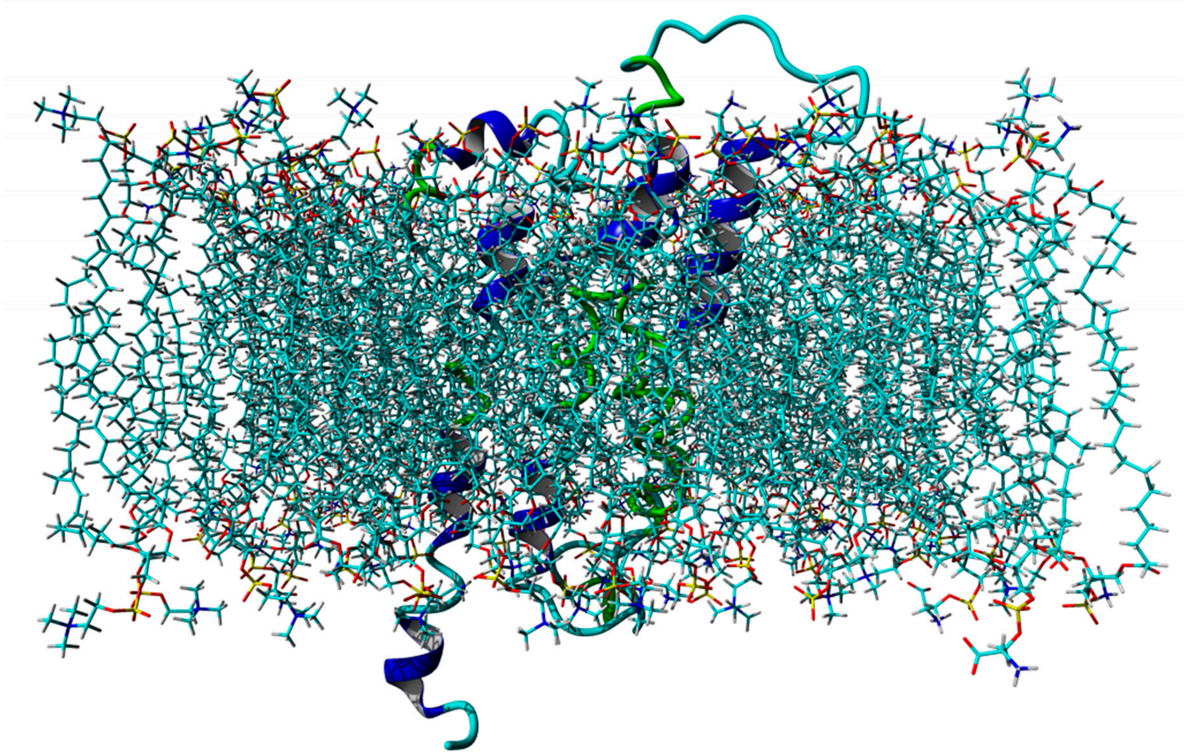

Fig. S3. Homology model of the σ_2-receptor immersed in the endoplasmic reticulum membrane.
